# An Orthogonal Conductance Pathway in Spiropyrans for Well‐Defined Electrosteric Switching Single‐Molecule Junctions

**DOI:** 10.1002/smll.202306334

**Published:** 2023-10-10

**Authors:** David Jago, Chongguang Liu, Abdalghani H. S. Daaoub, Emma Gaschk, Mark C. Walkey, Thea Pulbrook, Xiaohang Qiao, Alexandre N. Sobolev, Stephen A. Moggach, David Costa‐Milan, Simon J. Higgins, Matthew J. Piggott, Hatef Sadeghi, Richard J. Nichols, Sara Sangtarash, Andrea Vezzoli, George A. Koutsantonis

**Affiliations:** ^1^ School of Molecular Science The University of Western Australia 35 Stirling Highway Crawley Western Australia 6009 Australia; ^2^ Department of Chemistry University of Liverpool Crown St Liverpool L69 7ZD UK; ^3^ School of Engineering University of Warwick Coventry CV4 7AL UK; ^4^ Centre for Microscopy Characterisation and Analysis University of Western Australia Stirling Highway Crawley Western Australia 6009 Australia

**Keywords:** break‐junction, molecular devices, molecular electronics, spiropyran, switch

## Abstract

While a multitude of studies have appeared touting the use of molecules as electronic components, the design of molecular switches is crucial for the next steps in molecular electronics. In this work, single‐molecule devices incorporating spiropyrans, made using break junction techniques, are described. Linear spiropyrans with electrode‐contacting groups linked by alkynyl spacers to both the indoline and chromenone moieties have previously provided very low conductance values, and removing the alkynyl spacer has resulted in a total loss of conductance. An orthogonal T‐shaped approach to single‐molecule junctions incorporating spiropyran moieties in which the conducting pathway lies orthogonal to the molecule backbone is described and characterized. This approach has provided singlemolecule conductance features with good correlation to molecular length. Additional higher conducting states are accessible using switching induced by UV light or protonation. Theoretical modeling demonstrates that upon (photo)chemical isomerization to the merocyanine, two cooperating phenomena increase conductance: release of steric hindrance allows the conductance pathway to become more planar (raising the mid‐bandgap transmission) and a bound state introduces sharp interference near the Fermi level of the electrodes similarly responding to the change in state. This design step paves the way for future use of spiropyrans in single‐molecule devices and electrosteric switches.

## Graphical abstract

Reimagining molecular switches where the conducting pathway lies orthogonal to the molecular backbone provides a new class of electrosteric switches. Higher conducting states are accessible when the spiropyran switched to the merocyanine by light or protonation. Torsion of the backbone leads to increased orbital overlap between the anchor group and central functional core unit of the junction giving increased conduction.

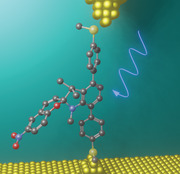

## 1 Introduction

Molecular electronics uses molecules contacted between electrodes as functional building blocks in electronic devices. Large‐area ensembles and single‐molecule junctions are used to probe the electrical properties and structure of molecular junctions.^[^ ^1^ ^]^ It is hoped that molecules will replace or become symbiotic with existing solid‐state circuitry and components such as wires, switches, and transistors in complementary metal‐oxide semiconductor (CMOS) technology. However, it has since evolved into a field beyond the silicon‐based paradigm.^[^ ^2^ ^]^ Currently, molecular electronics focuses on understanding the unique functions of single‐molecule junctions by increasing the library of molecules studied. Myriad discrete functions of molecular junctions have been explored including switching,^[^ ^3^ ^]^ rectification,^[^ ^4^ ^]^ thermoelectric energy conversion,^[^ ^5^ ^]^ electroluminescence,^[^ ^6^ ^]^ and spintronics.^[^ ^7^ ^]^

Controlling the transition between different conductance states of single‐molecule junctions is a coveted functionality.^[^ ^3^, ^8^ ^]^ Several stimuli, including chemical,^[^ ^9^ ^]^ electrochemical,^[^ ^10^ ^]^ mechanical,^[^ ^9^, ^11^ ^]^ and light,^[^ ^12^ ^]^ have been used to switch the conductance of single‐molecule junctions. In most studies, a change in the molecular backbone (i.e., length or shape) is associated with the conductance differences which require dynamic electrode separation between the conductance states. Conductance along the junction transport pathway can be imparted by an adjacent functionality's electronic or geometric properties. The demonstration and understanding of these effects usually require the comparison of a range of molecules whose properties are incrementally altered by structural variation, and therefore synthesis.^[^ ^13^ ^]^ The use of a stimulable side‐group, extrinsic to the junction electron transport pathway, to mediate the electron transport properties of single‐molecules is a much less explored phenomenon,^[^ ^14^ ^]^ and is a promising alternative to creating functional molecular devices that require static electrode positioning, which is likely required in realistic electronic devices. Most examples of side‐group conductance switching use chemical gating as the predominant stimulus. The use of light is advantageous due to greater spatial and temporal control of the stimulus.

Modeling and theory are important for understanding the conductance changes in switchable single‐molecule junctions, potentially allowing more rational design. Quantum interference plays a crucial role in the charge transport of single‐molecule junctions as the electrons traverse the molecules through the discrete orbitals.^[^ ^15^ ^]^ Constructive and destructive interference effects are observed when the electron wave functions of the transmittance pathways are in‐phase or out‐of‐phase, respectively. Switching the conductance of single‐molecule junctions by variation of constructive and destructive interference effects is a potential way to achieve functional molecular devices.^[^ ^16^ ^]^

**Figure 1:**
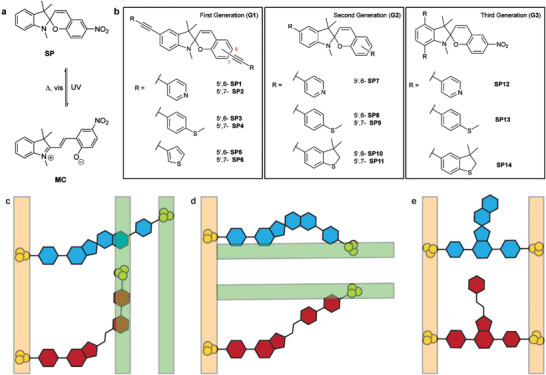
a) Scheme of the photoisomerization of the spiropyran to merocyanine. b) Spiropyrans studied in this work categorized into three generations (G1, G2, and G3). The isomerization of the spiropyran (blue) to merocyanine (red) in the linear type spiropyrans results in conformation changes within the single‐molecule device that require different electrode (represented as three gold circles) placements in both the c) cross‐conjugated and d) conjugated forms. This is represented by pinning one side of the electrode (yellow bars) and showing the displacement required for the second electrode (green bars). e) An orthogonal conductance pathway in the spiropyrans displays no electrode displacement (yellow bars can be pinned) upon isomerization from the spiropyran to the merocyanine.

Spiropyrans (SPs) are photochromic molecules that isomerize to a ring‐opened merocyanine (MC) upon exposure to UV light (**Figure** **1**a).^[^ ^17^ ^]^ This isomerization results in several changes in the properties of the molecules, the most significant being longer wavelength (visible light) absorption, increased conjugation, dipole moment, and length. Spiropyrans are putative molecular materials for optical filters, data storage, imaging, mechanosensing, and drug delivery applications.^[^ ^18^ ^]^

Single‐molecule junctions incorporating spiropyrans with weak binding electrode‐contact groups (nitro) showed switching under light and/or acid stimuli; however, the short junction lifetimes exhibited by these spiropyrans limited further junction characterization.^[^ ^19^ ^]^ Additionally, the short junction lengths in this survey exemplified the need for spiropyrans with efficacious anchoring groups. To this end, ethynyl and 4‐ethynylpyridyl functionalized spiropyrans have been considered.^[^ ^9b^ ^]^ The dative pyridyl binding groups enabled examination of the current‐voltage properties of these junctions in both the closed and protonated (open) merocyanine form, with rectification properties observed for the junctions containing the latter species. Spiropyran junctions with covalent Au‐C contacts ring‐opened under mechanical strain, leading to conductance spikes. Further evidence for switching spiropyrans under mechanical force has been shown in other break junction studies.^[^ ^20^ ^]^ Surface‐enhanced Raman scattering in addition to conductance and principal component analysis provided useful insights on the presence of the different spiropyran and merocyanine isomers in a single‐molecule junction.^[^ ^21^ ^]^

Inspired by the need to comprehensively study the isomerization of spiropyrans as potential molecular switches we have designed several generations of spiropyrans with contact groups suitable for studies in single‐molecule break junctions (Figure 1b). Here we compare three generations of molecules, two of which incorporate the switching moiety in the conduction path (**G1**, **G2**) and the other having it adjacent, namely, T‐shaped spiropyrans (**G3**). The first generations, **G1** and **G2**, were designed to access the conductance change upon isomerization to a conjugated or cross‐conjugated merocyanine (Figure 1c,d). Generation **G3** molecules have a conductance pathway orthogonal to the spiropyran to merocyanine isomerizing moiety. The geometry of generation **G3** molecules also mitigates the large conformational change inherent in **G1** and **G2** molecules on the spiropyran to merocyanine ring opening, potentially creating more stable break junctions (Figure 1e). We believe that this stabilization will be required to facilitate the development of future single‐molecule devices that require static electrode placements for CMOS compatibility.^[^ ^22^ ^]^

## 2 Results and Discussion

### 2.1 Design and Synthesis

Figure 1b shows the spiropyrans synthesized in this study. We have grouped them into three generations to make clear the evolution of our results and previous studies.^[^ ^9^, ^19^, ^20^, ^21^ ^]^ Three different conductance paths with the contacting (anchor) groups 4‐thiomethylphenyl, 3‐thienyl, 3,3‐dimethyl‐2,3‐dihydrobenzo[*b*]thiophen‐5‐yl (DMBT) and the 4‐pyridyl contacting groups were studied. Details of the synthesis, standard characterization, light‐ and acid‐induced switching studies, and single‐crystal X‐ray crystallography are detailed in the Supporting Information.

### 2.2 Single‐Molecule Conductance

The scanning tunneling microscope‐break junction (STM‐BJ)^[^ ^23^ ^]^ technique was used to fabricate junctions of **SP** and to measure their conductance. This method uses an STM apparatus to repeatedly form and rupture Au point contacts between the electrode tip and a substrate in the presence of a solution of the target molecule. Upon rupture of the point contact, the molecule can self‐assemble in the nanogap, forming a molecular junction and allowing the measurement of its charge transport properties under a DC bias. Transport through the junction is recorded as conductance $G\;=\frac{I}{V}$ in units of the quantum of conductance *G*_0_(≅ 77.48 µ*S*) during the tip withdrawal process, yielding traces of *G* versus electrode separation. This process is repeated thousands of times to ensure statistical robustness. The traces are then compiled into 1D statistical histograms that yield the most probable value of conductance, and 2D density maps, which show the correlation between conductance values and junction length. Details on the equipment used in this study and the data analysis process can be found in our previous studies^[^ ^10^, ^24^ ^]^ and in the Supporting Information.

**Figure 2:**
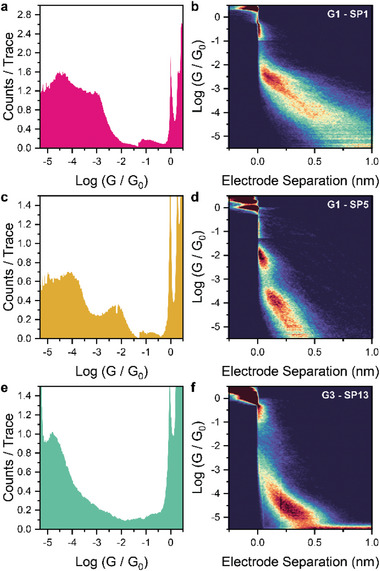
Selected histograms and 2D plots for a,b) SP1, c,d) SP5, and e,f) SP13. As discussed in the text, the G1 materials SP1 and SP5 do not show features in the 2D plot that can be attributed to transport through the extended molecular wire (i.e., at electrode separation > 0.75 nm). All histograms and 2D plots were compiled with 100 bins per conductance decade, 100 bins per nanometer, and are normalized to the number of scans acquired. SP1 and SP5 were measured at 200 mV bias. SP13 was measured at 500 mV bias. All measurements were performed in mesitylene (1,3,5‐trimethylbenzene), at 1 mm concentration of the target compound.

The 1D histograms and 2D maps of selected spiropyrans are shown in **Figure** **2** and additional conductance data, histograms, and maps are collected in Table S8, Supporting Information and in the Supporting Information. The conductance histograms of the first generation spiropyrans **SP1** (Figure 2a) and **SP5** (Figure 2c), show both a high and low conductance peak. The conductance histogram of **SP2** only displayed one conductance feature and no conductance features were observed for **SP3**. We suggest that the increase in molecular length of **SP3** compared to **SP1** results in there being no observable value in the conductance histogram. Further analysis of the junction heatmaps, Figure 2b,d, with further examples available in the supporting information and in Table S8, Supporting Information, demonstrate that these junctions cannot be extended to their full molecular length—that is, the electrode separation at which junctions break is considerably shorter than the molecular length as determined by single‐crystal X‐ray diffraction studies or DFT modeling. We attribute these short break‐off lengths to either incomplete junction stretching (i.e., the molecule lies tilted in the gap and cannot be lifted to a fully extended configuration) or a shorting of the junction (i.e., contact of one electrode at the midpoint of the molecule).^[^ ^20b^ ^]^ We have further shown using theory that this shorting of the molecular junctions leads to higher conductances in close agreement with values obtained for the junctions formed here for the first generation spiropyrans.

Upon the addition of a dilute solution of trifluoroacetic acid to **SP5** the high conductance feature disappeared, and the low conductance feature remained relatively unchanged (Figure S112, Supporting Information). These observations could be the consequence of a shorting of the junction as described above affecting the high conductance feature. The addition of a proton hinders the ability of the electrode to bind at the midpoint of the molecule, hence the disappearance of this high conductance feature. We therefore ascribe the low conductance feature of **SP5** to the spontaneous opening of the spiropyran to the merocyanine, which is further evinced by the similar feature observed upon the addition of TFA to single‐molecule junctions of **SP5**. These data also infer that the single‐molecule conductance of **SP5** in the spiropyran form lies beyond our visible conductance window. The original conductance plots measurements could not be regenerated upon neutralization with NEt_3_ but instead led to noisy histograms with no discernible features, a complication most probably arising from the formation of triethylammonium triflate which acted as an ionic conductor, and thus increased faradaic currents in the STM liquid cell. We are currently further investigating these observations in the hope of finding a definitive cause.

Having established that first‐generation materials are not suitable for robust studies, we focused our efforts on the second generation spiropyrans, **SP8** and **SP9**. These shorter versions of the first‐generation compounds lacked only the ethynyl spacer thus we expected higher conductance and the fabrication of junctions of length commensurate to the shorter molecular S–S or N–N distances. To our surprise, these compounds showed no clear conductance features in the histograms (Table S8, Figures S113, and S114, Supporting Information). Analysis of the individual traces reveals an elongated tunneling signal with a decay constant lower than

that obtained in pure solvent, which failed to settle on a plateau before drifting into the noise level of the instrument (≈10^−5.5^ *G*_0_ at the bias voltages employed in this study). Our data points toward efficient junction formation, but poor orbital overlap between the spiropyran backbone and the twisting of the installed contact groups, due to steric encumbrance, results in inefficient charge transport, with associated conductance values falling below the sensitivity of our instrumentation. The disappointing results of these pilot systems meant we did not pursue the other **G2** spiropyrans but instead turned our attention to an alternative molecular structure for single‐molecule junctions.

**Figure 3:**
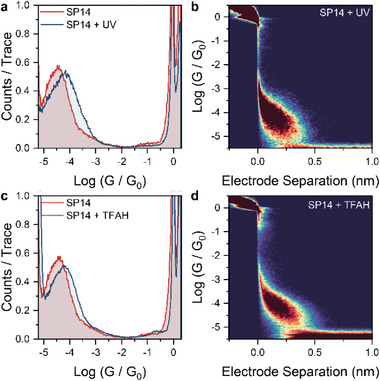
Switching experiments. a) Comparison of conductance histograms of SP14 before and after in situ irradiation with UV light. b) 2D density map for SP14 after irradiation. c) Comparison of conductance histograms of SP14 before and after in situ addition of trifluoroacetic acid (TFAH). d) 2D density map for SP14 in the presence of TFAH. All histograms and 2D maps were compiled with 100 bins per conductance decade, 100 bins per nanometer, and normalized to the number of scans acquired. All data were obtained at a DC bias of 500 mV, 1 mm concentration of SP14 in 1,2,4‐trichlorobenzene.

We therefore designed and synthesized the T‐shaped third generation spiropyran system, terminated either with 4‐pyridyl (**SP12**), 4‐thiomethylphenyl (**SP13**), or DMBT (**SP14**) contact groups. The histogram and density map for **SP12** suggest a conductance feature that moves into the noise floor of the conductance window (i.e., the single‐molecule conductance is below 10^−5.5^ G_0_). **SP13** and **SP14** showed well‐defined conductance histograms with break‐off distances in good agreement with fully extended molecular length (Figures 2e,f and **3**).

These compounds show significantly suppressed charge‐transport efficiency when compared to simple terphenyl analogs.^[^ ^25^ ^]^ The phenomenon can be ascribed to the bulky spiropyran moiety forcing the 4‐thiomethylphenyl (**SP13**) or DMBT (**SP14**) ring to twist out of plane, reducing the overall conjugation along the conductive pathway.^[^ ^26^ ^]^ Of the three T‐shaped third‐generation compounds synthesized, we focused our effort on **SP14** for further switching studies, as it was the material showing the highest conductance value, with a clear conductance peak in the histogram sitting above the noise level of our instrumentation. We designed an irradiation system for the STM‐BJ apparatus, which comprises a UV LED (385 nm peak wavelength, 1.2 W radiant flux @ 3.5 V) mounted on a freestanding Al beam and a power supply. The UV LED is put in close proximity (≈5 mm) to the STM liquid cell, and a wide dome (≈120°) lens is used to illuminate the 1 mm solution of **SP14** in situ.

After irradiating the solution for 20 min (to trigger full spiropyran → merocyanine conversion in the molecular junction) we observed an increase in conductance in the junction formed of approximately half an order of magnitude, from 10^−4.51^ *G*_0_ to 10^−4.08^ *G*_0_. Irradiating for a longer time did not produce any noticeable difference. The system was kept in strict darkness during data collection, and we limited the acquisition window to 40 min after irradiation (≈2500 conductance traces), to minimize the effect of the spontaneous merocyanine→ spiropyran thermally activated back‐conversion.

Similarly, a conductance increase was observed upon the addition of a dilute solution of trifluoroacetic acid (≈50 µL, ≈0.13 m). In this case, the change was more modest (from 10^−4.51^ *G*_0_ to 10^−4.22^ *G*_0_). Again, increasing the amount of acid added to the solution resulted in no change to the peak position, indicating full spiropyran → protonated merocyanine conversion.

A practical single‐molecule switch for building molecular devices requires a static connection between two nano‐electrodes. In addition to the on‐off ratio of the conductance, the performance of a molecular switch is also measured with switching frequency and stability or longevity. The STM‐BJ technique is unsuitable to test other performance factors. The results presented represent the first steps toward improving single‐molecule devices of spiropyrans. Additional techniques, such as the I(t) measurement and mechanically controlled break junctions should provide more valuable information on the performance of these and future similar devices. We are currently exploring these additional switching aspects along with improvements to the current systems but remain outside the scope of the central hypothesis presented in this work.

In both the photochemical and acid stimuli cases, switching from a less‐conductive spiropyran form of the molecular wire to a more efficient‐conducting merocyanine structure by cleavage of the C‐spiro‐O bond was observed. Junctions fabricated with the compound in its merocyanine form can be extended to lengths commensurate with the calculated S–S distance (1.5 nm) as evinced by the 2D density maps pictured in Figure 3b,d. In both cases the junctions extend to electrode separations of ≈0.5 nm, corresponding to junction size of ≈1 nm, accounting for electrode snapback.^[^ ^27^ ^]^ The change in charge‐transport efficiency in these T‐shaped compounds, however, is not in line with that observed in previous generation materials (albeit, as discussed earlier, most probably happening through a non‐extended, or shorted wire in the junction),^[^ ^9b^ ^]^ or with the observed changes in optical band gap monitored by UV–vis spectroscopy. The latter would suggest a dramatic change in the electronic structure of the two isomers upon conversion (spiropyran ⇌ merocyanine), which was not reflected in our single‐molecule conductance measurements. As simple electronic band structure cannot account for the observed behavior, we turned our attention to in silico modeling methods to shed light on the phenomena in place in the T‐shaped molecular system.

### 2.3 Theoretical Calculations

In order to gain insight into the charge‐transport properties of third generation spiropyran (**G3**) systems, we performed theoretical modeling with the SIESTA^[^ ^28^ ^]^ implementation of Density Functional Theory (DFT). The methodology is described in the Supporting Information. The ground‐state geometry and electronic structure of gas‐phase **SP14** terminated with DMBT contact groups were first computed. Subsequently, the electron transport properties of the compounds presented in Figure 1d (**SP14**, **SP14**+UV, **SP14**+TFAH) connected to two Au electrodes were calculated. The mean‐field Hamiltonian of the junctions from the ground‐state relaxed geometry was constructed, and the GOLLUM^[^ ^29^ ^]^ transport code was used to calculate the transmission coefficient *T*(*E*) of electrons with energy (*E*) passing from one electrode to the other through the molecule. By applying the Landauer formula, we then calculated the conductance of junctions (see the Supporting Information for further details).

**Figure 4:**
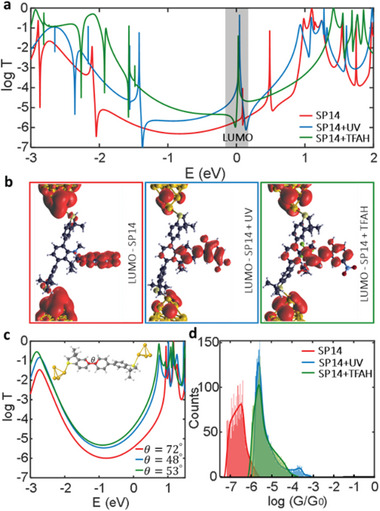
DFT conductance calculations. a) Transmission coefficients for (SP14, SP14+UV, SP14+TFAH). b) Local densities of states of LUMO resonances (grey area shown in transmission plot in (a)). c) Transmission coefficients of model backbone with no side group. d) Calculated room temperature conductance histograms over a range of molecule‐electrode conformations (see Supporting Information for more information) for SP14, SP14+UV, SP14+TFAH molecular junctions. *E* = 0 eV shows DFT Fermi energy (EF).

**Figure** **4**a shows the calculations of the transmission coefficient *T*(*E*) for **SP14** (red line), **SP14**+UV (blue line), and **SP14**+TFAH (green line). For all junctions, a sharp Fano resonance feature close to the DFT Fermi energy (*E* = *E*_F_ = 0 eV in Figure 4a) was obtained. A Fano resonance is a quantum interference effect and indicative of a localized bound state interacting weakly with the continuum of states.^[^ ^30^ ^]^ Local density calculations around the LUMO (grey area in Figure 4b) show that these are due to the localized states on the chromene side group. The wave function calculations represented in Figures S124–S126, Supporting Information, and Figures S128 and S129, Supporting Information also indicate that the LUMO is localized on the chromene side group. The width of these resonances is larger for the merocyanine and protonated merocyanine which leads to a greater effect on transmission coefficient. We postulate that the bound state can interact more with the transmission channel in the merocyanine form due to increased conjugation. It is evident that the amplitude of the red curve (**SP14**) is smaller than those for the other two molecules (**SP14**+UV and **SP14**+TFAH) for a wide range of Fermi energy levels [−1.4–0 eV]. We attribute this to the different dihedral angles between the anchor group (DMBT) and the backbone due to changes in the molecular structure upon UV radiation. To further support this idea, the transmission coefficient for model **SP14**‐based molecular junctions without side‐groups (i.e., terphenyl) was calculated but using the same geometry of molecules that have the chromene side groups. As shown in Figure 4c, the red curve (**SP14**) has a smaller amplitude than those for the other two molecules for all energies between the HOMO‐LUMO resonances. This is because the dihedral angle of the spiropyran form **SP14** (*θ* = 70°) is larger than that of the merocyanine form, generated with UV radiation (*θ* = 48°) or by the presence of TFAH (*θ* = 53°). We find that the shape of side groups has a significant impact on the dihedral angle between the anchor group and the central benzene moiety. For instance, the side group of an **SP14** molecule is vertically aligned with the backbone, whereas other molecules have a more planar shape, which leads to a decrease in the dihedral angle (see Figures S121–S123, Supporting Information for further details). We note that no Fano resonances are found when the side group is removed. This suggests that transport resonances due to the side groups do not have a significant effect on the conductance differences observed in our experiment and are far away from the Fermi energy. We note that the DFT Fermi energy is inaccurate^[^ ^31^ ^]^ and that is why *T*(*E*) is calculated for a wide range of energies and then we form calculated conductance histograms as discussed below. The major effect in the overall transmission is because of changes through the conducting backbone, and the side group influences the backbones’ structure.

To investigate the effect of junction conformations on single‐molecule transport, the conductance histograms as shown in Figure 4d were computed. This is to mimic an STMBJ experiment, where each crash/withdrawal cycle generates a fresh nanogap of an unknown atomic structure. A variety of junction configurations were explored by altering the angle between the molecules and the gold electrodes (further information, including the structures of the junctions, can be found in the Supporting Information), and the transmission function for each structure was calculated. From these, we calculated the conductance histograms for each compound, as shown in Figure 4d for a wide range of energies between HOMO–LUMO gap.^[^ ^32^ ^]^ The center of log‐normal distribution fitted to these histograms agreed with the experimental findings of G**_SP14_** < (G**_SP14_**_+UV_ and G**_SP14_**_+TFAH_) and G**_SP14_**_+UV_ ≈ G**_SP14_**_+TFAH_.

## 3 Conclusion

This work demonstrates that both UV and acid switching of spiropyran single‐molecule junctions produce well‐defined plateaus and conductance peaks. The single‐molecule junctions of the merocyanine and protonated merocyanine show increased conductance over the spiropyran. Theoretical assessment of the junctions shows that the conductance increase is due to torsional rearrangement between the terphenyl‐like backbone leading to increased orbital overlap between the anchor group and central functional core unit of the junction. These results highlight the need and potential for rational molecular engineering to enable switching molecular electronic devices. This approach appears to be a rich vein of research to explore in the future. Additional spiropyrans to compensate for the torsional ring structure and to improve the switching performance of these single‐molecule devices presented here are being explored.

## Conflict of Interest

The authors declare no conflict of interest.

## Author Contributions

G.A.K. conceived the project. D.J., E.G., M.C.W., and T.P. synthesized the compounds used in this study and performed their characterization. D.C.‐M., R.J.N., and A.V. designed the charge transport experiments. D.J., C.L., and X.Q. performed the single‐molecule experiments and analyzed the data with software written by A.V. A.H.S.D., H.S., and S.S. performed the computational studies. A.N.S. and S.M. performed single‐crystal X‐ray diffraction experiments and analyzed the data. M.J.P., S.S., A.V., and G.A.K. supervised the study. All authors contributed to the discussion of the results. D.J., S.S., A.V., and G.A.K. wrote the paper with contributions from all authors.

## Supporting information

Supporting Information

## Data Availability

The data that support the findings of this study are openly available in the University of Liverpool Data at https://10.17638/datacat.liverpool.ac.uk/2258, reference number 2258.
