# Supplementary material for: An Orthogonal Conductance Pathway in Spiropyrans for Well‐Defined Electrosteric Switching Single‐Molecule Junctions
Source: Small. 2023 Oct 10;20(8):2306334. doi: 10.1002/smll.202306334 (PMC11475379; doi:10.1002/smll.202306334)
Supplement: Supplementary file 1 — Supporting Information [file SMLL-20-2306334-s001.pdf]

NANO · MICRO
small

Supporting Information

for *Small*, DOI 10.1002/smll.202306334

An Orthogonal Conductance Pathway in Spiropyrans for Well-Defined Electrosteric Switching Single-Molecule Junctions

David Jago, Chongguang Liu, Abdalghani H. S. Daaoub, Emma Gaschk, Mark C. Walkey, Thea Pulbrook, Xiaohang Qiao, Alexandre N. Sobolev, Stephen A. Moggach, David Costa-Milan, Simon J. Higgins, Matthew J. Piggott, Hatef Sadeghi, Richard J. Nichols, Sara Sangtarash, Andrea Vezzoli* and George A. Koutsantonis**

Supporting Information for

An Orthogonal Conductance Pathway in Spiropyrans for Well-Defined Electrosteric Switching Single-Molecule Junctions

David Jago,[a] Chongguang Liu,[b] Abdalghani H. S. Daaoub,[c] Sara Sangtarash,[c] Emma Gaschk,[a] Mark C. Walkey,[a] Thea Pulbrook,[a] Xiaohang Qiao,[b] Alexandre N. Sobolev,[d] Stephen Moggach,[a] David Costa-Milan,[b] Simon J. Higgins,[b] Matthew J. Piggott,[a] Hatef Sadeghi,[c] Richard J. Nichols,[b] Andrea Vezzoli,*[b] and George A Koutsantonis*[a]

[a] D. Jago, E. Gaschk, M. C. Walkey, T. Pulbrook, S. Moggach, M. J. Piggott and G. A. Koutsantonis
School of Molecular Science
The University of Western Australia
Stirling Highway, Crawley, WA 6009 (Australia)
E-mail: george.koutsantonis@uwa.edu.au

[b] C. Liu, X. Qiao, D. Costa-Milan, S. J. Higgins, R. J. Nichols, A. Vezzoli
Department of Chemistry
University of Liverpool
Crown St, Liverpool L69 7ZD (UK)

[c] A. H. S. Daaoub, S. Sangtarash, H. Sadeghi
School of Engineering
University of Warwick
Coventry CV4 7AL (UK)

[d] A. N. Sobolev
Centre for Microscopy, Characterisation and Analysis
University of Western Australia
Stirling Highway, Crawley, WA 6009 (Australia)

Table of Contents

Synthesis and Characterisation 1

X-ray Crystallography 86

Switching 100

Single-molecule Conductance 109

Additional DFT-NEGF Details 115

References 122

Synthesis and Characterisation

Full Synthesis

Designing molecules for single-molecule junctions requires incorporation of functional groups that can form stable bonds to metal electrodes.[1] For this purpose, we synthesised several spiropyrans containing the 4-thiomethylphenyl, 3,3-dimethyl-2,3-dihydrobenzo[*b*]thiophen-

5-yl (DMBT) or pyridyl contacting groups. Thioether groups are well established anchor groups that support both hole and electron mediated transport in single-molecule junctions.[2] The related benzodihydro[*b*]thiophene (BT) contact group offers high junction formation probability in STM break junction experiments.[3] The addition of the 3,3-dimethyl groups enhances solubility and is synthetically more accessible.[4] Pyridyl contact groups also serve as a complementary anchor group.[3a, 3d, 5]

The TMS protected dialkynyl spiropyrans **4** and **5** were synthesised by condensing the indolinium salt **1** and either the salicylaldehyde **2** or **3** in the presence of piperidine.[6] Upon cooling of the reaction mixture, **4** precipitated out as a spectroscopically pure powder. The terminal alkynyl spiropyrans **6** and **7** were obtained by desilylation using *n*Bu_4NF in good yields. Subsequent Sonogashira coupling of the diethynyl spiropyrans with the appropriate aryl iodide capped the molecules with appropriate gold bind groups to give **SP1** to **SP6** (Scheme S1).

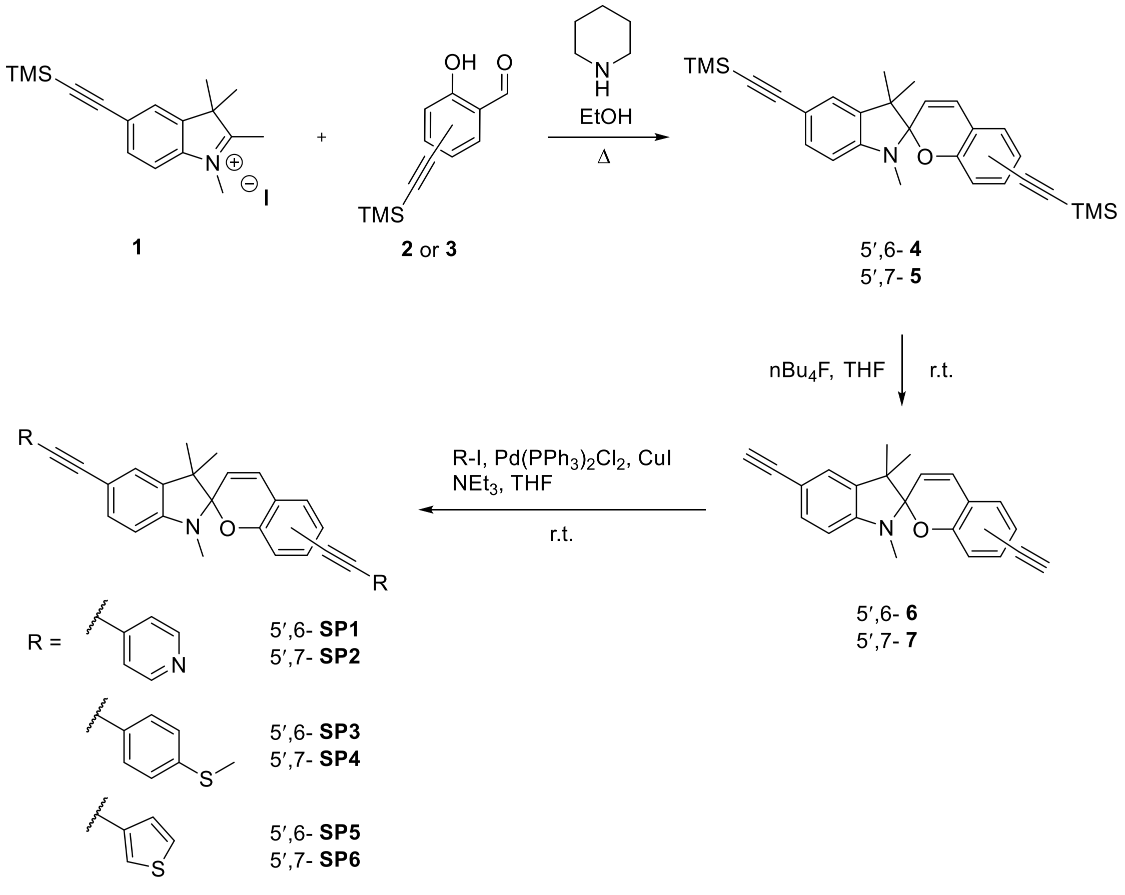

Scheme S1 Synthesis of first generation (G1) spiropyrans

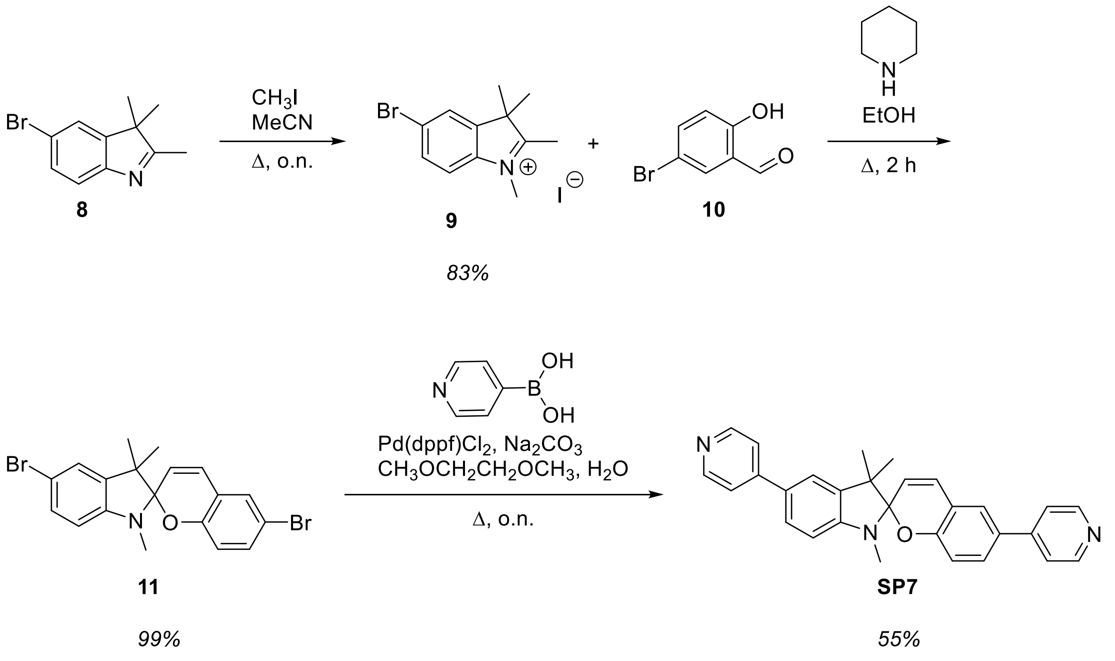

Scheme S2 Synthesis of the second generation (G2) spiropyran **SP7**

SP7 was synthesised by palladium-catalysed Suzuki coupling of 4-pyridylboronic acid and 5′,6-dibromo-spiropyran **11** (Scheme S2). During this study, Bhattacharyya *et al.* also reported the synthesis of **SP7**.[7] Our methodology provides a concise workup and purification procedure that gives a more pure product by comparison of ^{1}H NMR spectra and elemental analysis. **11** was prepared by the condensation of 5-bromosalicylaldehyde and 5-bromoindolium iodide. This method is higher yielding than the previously reported synthesis by brominating 6-bromospiropyran.[8] More extensive characterisation of **11** is also reported in this study.

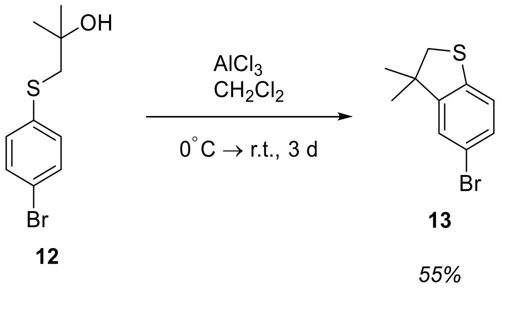

Scheme S3 Synthesis of bromo-DMBT

The cyclic thioether 5-bromo-3,3-dimethyl-2,3-dihydrobenzo[*b*]thiophene **13** was prepared by a modified literature procedure (Scheme S3). Meisner *et al.* first reported the synthesis of **13** in 2012.[9] Naher *et al.* reported a modified version more recently.[4] The discrepancies

between the procedures are the equivalents of $AlCl_3$ used and reaction time. We found that one equivalent of $AlCl_3$ and the pre-cursor alcohol exchanges the hydroxy group for a chloride group.[10] The additional improvement here relies on the controlled temperature and rate of addition of **12** to a dilute suspension of $AlCl_3$ in CH_2Cl_2. The product was isolated in a higher yield than previously reported with only column chromatography required for purification.

Both the acyclic and cyclic thioether (**13** and **14**) were converted to their boronic acid analogues (**15** and **16**) using lithium halogen exchange followed by quenching with triisopropyl borate (Scheme S4).[11]

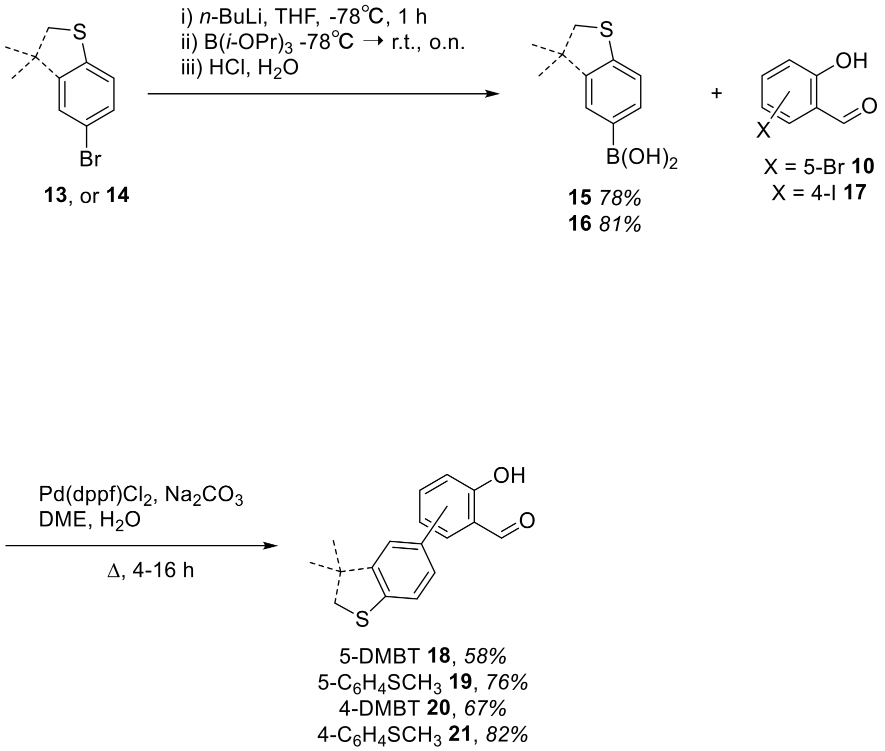

Scheme S4 Synthesis of salicylaldehyde **18**-**21**

All salicylaldehyde and indole derivatives were synthesised by Suzuki coupling of the thioether boronic acid (**15** or **16**) and respective halogenated moiety (**10**, **17**) (Scheme S4) or **8** (Scheme S5).[12] Spiropyrans containing the thioether contact groups, **SP8** – **SP11**, were prepared by

condensation an indolium iodide (**24** or **25**) and salicylaldehyde (**18**, **19**, **20** or **21**) (Scheme S5).[13]

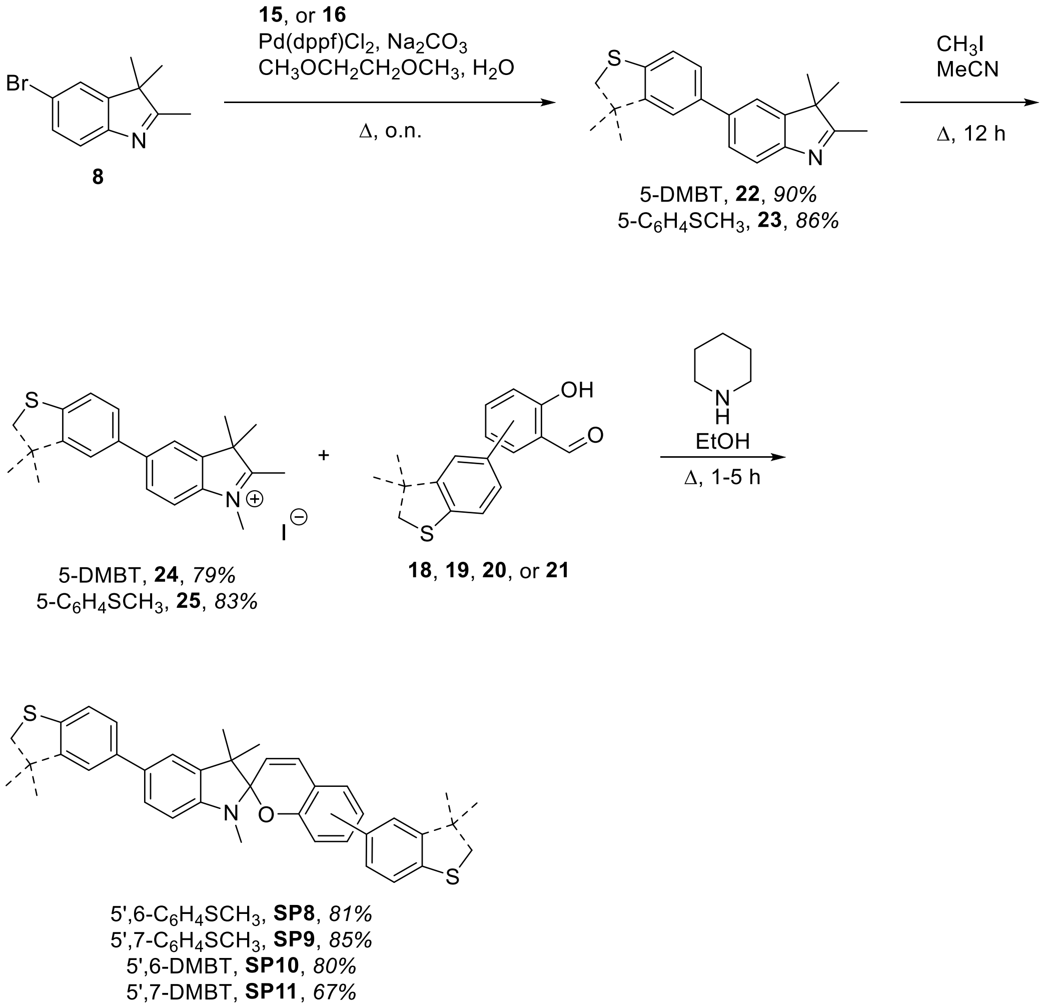

Scheme S5 Synthesis of second generation (G2) spiropyrans **SP8**-**SP11**

Scheme S6 and Scheme S7 show the synthesis of **SP12**, and **SP13** and **SP14**. Compounds **27** – **30** were synthesised by slightly modified literature procedures.[14] Methylation of **30** using trimethyloxonium tetrafluoroborate gave **31** which was further reacted with **32** to furnish **33**. Suzuki coupling of **33** with 4-pyridylboronic acid gave **SP12**. Compounds **34** and **35** were synthesised by Suzuki coupling of **15** or **16** to **30**, and subsequent methylation using iodomethane gave **36** and **37**. These indolinium salts reacted with 4-nitrosalicylaldehyde in the presence of piperidine to give **SP13** and **SP14**.

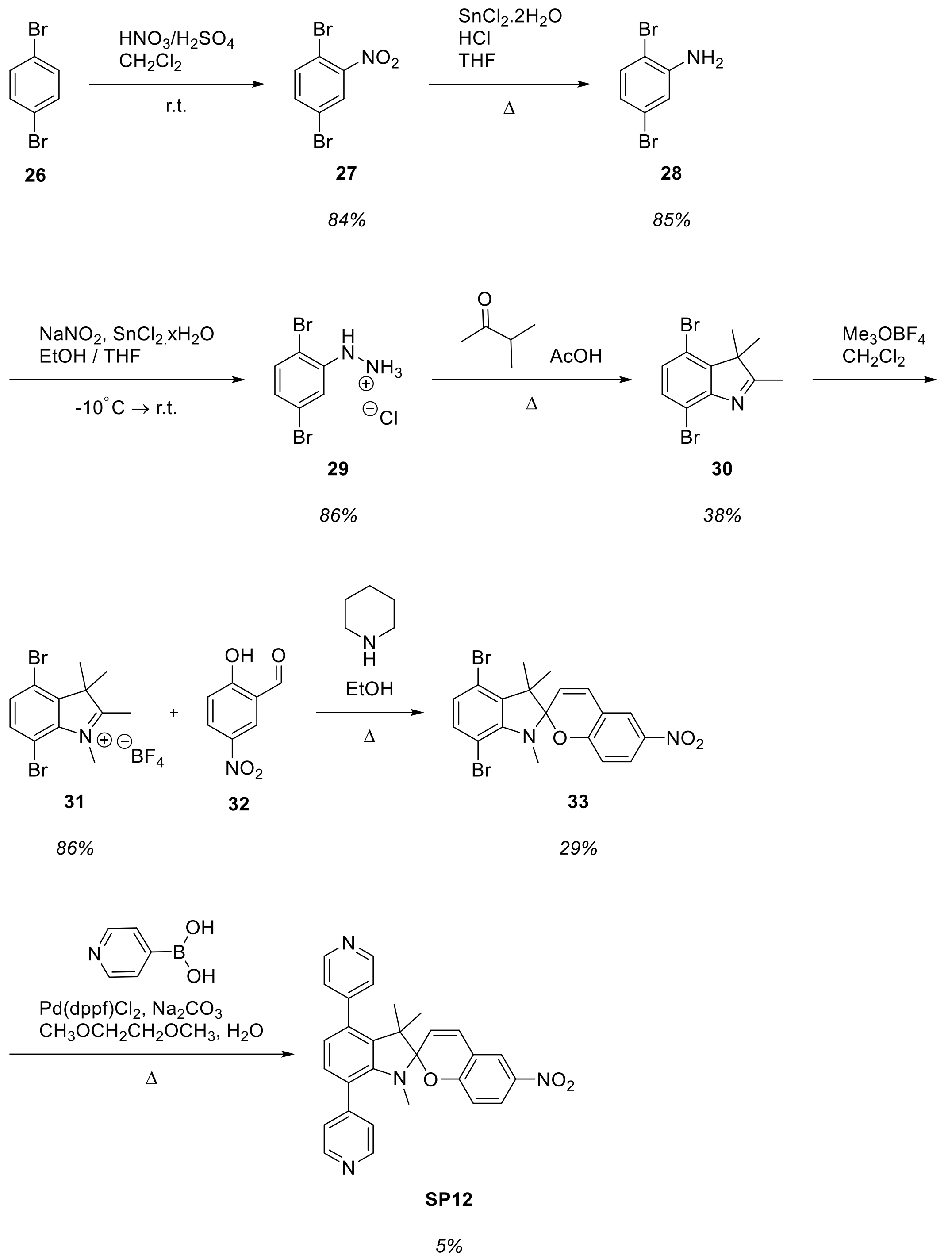

Scheme S6 Synthesis of the third generation (G3) spiropyran **SP12**

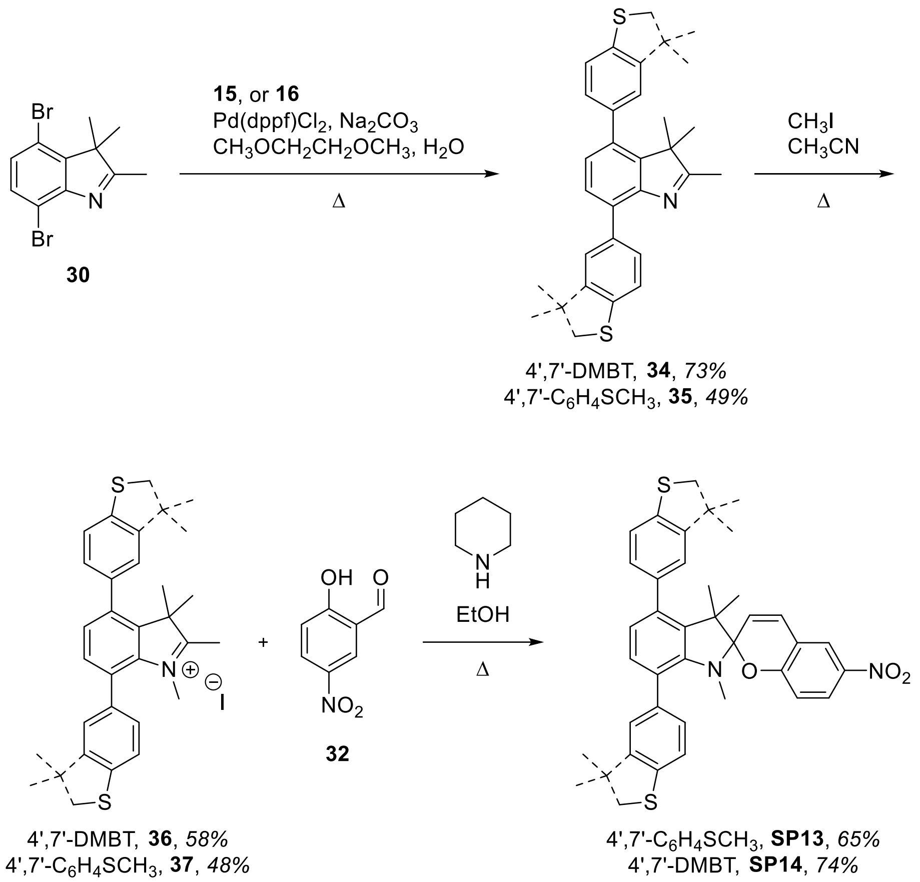

Scheme S7 Synthesis of the third generation (G3) spiropyrans **SP13** and **SP14**

General Experimental

All reactions were carried out under high purity argon or nitrogen using Schlenk techniques and oven-dried glassware. All solvents for reactions were dried by distillation under argon over an appropriate drying agent or by an Innovative technologies SPS and deoxygenated (freeze/pump/thaw or sparging) before use. Hexanes and EtOAc for column chromatography were distilled before use. All other solvents were of AR grade and used without further purification. Unless otherwise stated, no special precautions were taken to exclude air or moisture during work-up and purification. All other reagents were used as received from their supplier. Flash column chromatography was carried out using silica gel 60 (0.04 – 0.063) or alumina (basic, activity IV). Thin layer chromatography was carried out using Merck silica gel 60 F254 pre-coated aluminium sheets.

^{1}H and ^{13}C spectra were recorded at 25°C on a Bruker 600 MHz, 500 MHz, or 400 MHz spectrometer. Chemical shifts (δ) are referenced to the internal undeuterated residual

solvent signal (^1H) or deuterated solvent signal (^{13}C). First order multiplets are as follows: s (singlet), d (doublet), t (triplet), q (quartet), quin (quintet), sext (sextet), oct (octet), dd (doublet of doublets), ddd (doublet of doublet of doublets), dt (doublet of triplets), tt (triplet of triplets). Apparent multipets are labelled with the prefix 'app.'. Broad signals are labelled with the prefix 'br.'. Multiplets of higher order or undeterminable are labelled as multiplet (m). Coupling constants (*J*) are reported in hertz (Hz). Signal assignments were made with help from HSQC, HMBC and COSY experiments, spectrum prediction software and literature precedent.

Infrared spectroscopy was carried out as neat products using an ATR module fitted FTIR spectrometer. Electrospray ionisation (ESI) or atmospheric-pressure chemical ionisation (APCI) mass spectrometry was carried out on a Waters LCT Premier TOF spectrometer or Thermofisher Orbitrap Exploris spectrometer. In most cases, HPLC grade acetonitrile was used as the solvent. If needed, a couple of drops of dichloromethane was used to help solubilise the compound for analysis. Melting points were collected on a Buchi M-565. Elemental analyses were performed by London Metropolitan University, London, United Kingdom.

Procedures and Characterisation

The compounds **1**,[6b] **4**, **6** and **SP1**[6c] were synthesised using previous methodology.

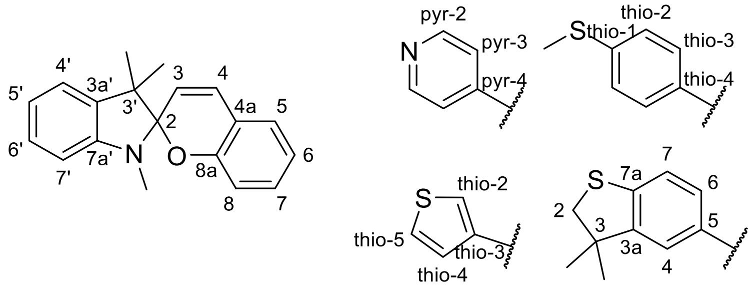

Figure S1 General numbering scheme for spiropyrans

2-hydroxy-5-((trimethylsilyl)ethynyl)benzaldehyde (**2**):

Synthesised by modified literature procedure.[15] IR and HRMS data collected and $^{13}C\{^{1}H\}$ NMR assigned. Anhydrous NEt3 (30 mL) was deoxygenated by sparging with argon for 20 min. Then 5-bromo-2-hydroxybenzaldehyde (2.03 g, 10.1 mmol), trimethylsilylacetylene (2.5 mL), CuI (130 mg, 0.684 mmol) and $Pd(PPh_3)_2Cl_2$ (220 mg, 0.314 mmol) were added and the solution was refluxed overnight. After cooling to room temperature, the solvent was removed under reduced pressure. The residue was taken up in 20% EtOAc:Hexanes and filtered through a small silica plug. The solvent and other volatiles were again removed under reduced pressure. The crude brown solid was recrystallised from hot heptane to give **2** as an off-white solid (1.41 g, 64%).

^{1}H NMR ($CDCl_3$, 600 MHz) δ /ppm: 11.09 (s, 1H, OH), 9.85 (s, 1H, CHO), 7.70 (d, *J* = 2.0 Hz, 1H, H6), 7.60 (dd, *J* = 2.0, 8.7 Hz, 1H, H4), 6.93 (d, *J* = 8.7 Hz, H3), 0.25 (s, 9H, $Si(CH_3)_3$).

$^{13}C\{^{1}H\}$ NMR ($CDCl_3$, 151 MHz) δ / ppm: 196.03 (CHO), 161.57 (C2), 140.14 (C4), 137.39 (C6), 120.40 (C1), 118.01 (C3), 115.15 (C5), 103.28 (Cα), 93.85 (Cβ), 0.01 ($Si(CH_3)_3$).

FTIR (ATR, neat) ν / cm^{-1}: 3203 (OH), 2956, 2879, 2149 (C≡C), 1665 (C=O), 1610, 1571.

HRMS (TOF AP(+)) *m/z*: $[M]^+$ Calcd for $C_{12}H_{14}O_2Si^+$ 218.0758. Found 218.0763.

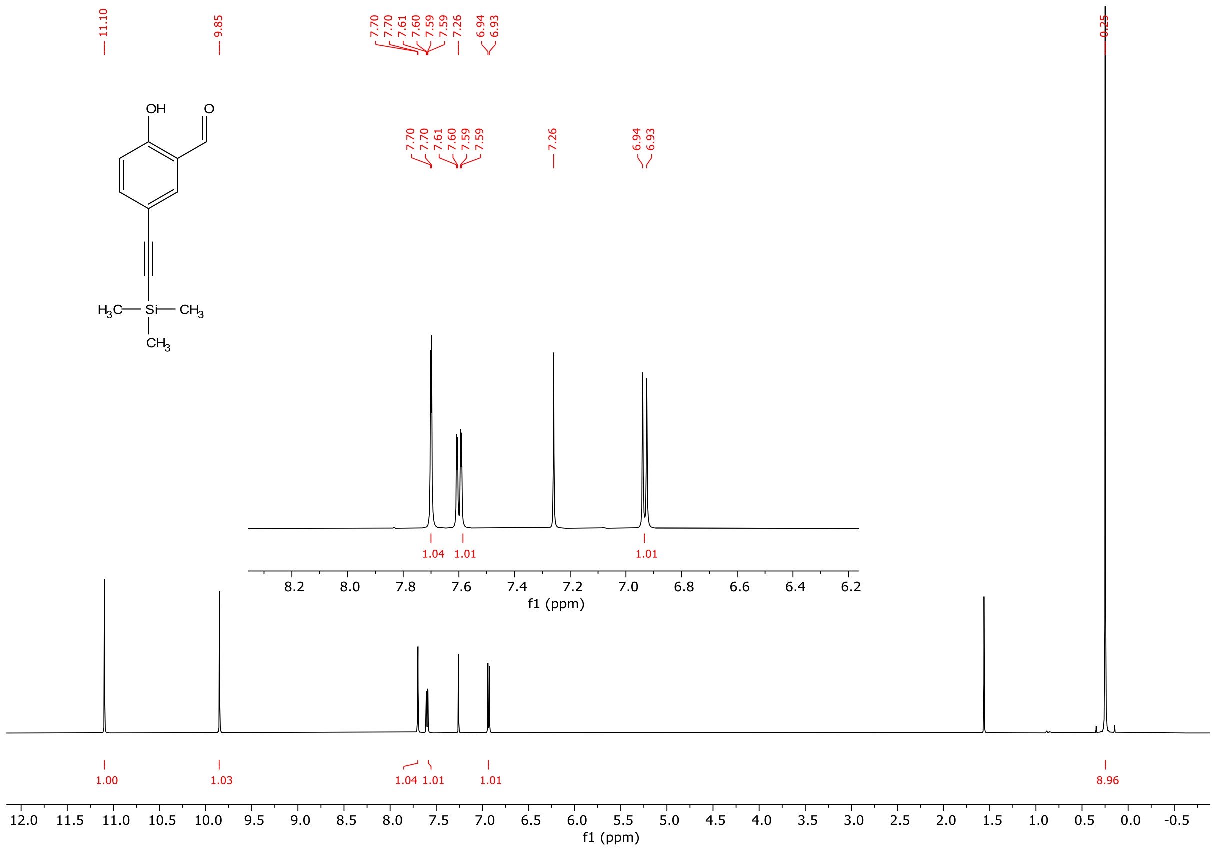

Figure S2 ^{1}H NMR Spectrum of **2** in $CDCl_3$.

2-hydroxy-4-((trimethylsilyl)ethynyl)benzaldehyde (**3**):

2-hydroxy-4-iodobenzaldehyde[16] (55 mg, 0.22 mmol), $Pd(PPh_3)_2Cl_2$ (10 mg, 0.014 mmol) and CuI (8 mg, 0.04 mmol) were charged to a Schlenk tube. Then THF (5 mL) , NEt_3 (0.1 mL) and trimethysilyacetylene (0.1 mL, 0.7 mmol) were added and the reaction was stirred overnight. The solvent as removed *in vacuo*. The residue was subject to flash column chromatography (silica, 10% EtOAc:Hexanes) to give **3** (42 mg, 87%) as a pale yellow powder. The ^{1}H, ^{13}C{^{1}H} NMR and IR spectra are consistent with the literature.[17] ^{1}H and ^{13}C{^{1}H} peaks NMR are assigned.

^{1}H NMR ($CDCl_3$, 500 MHz) δ /ppm: 10.99 (s, 1H, OH), 9.87 (s, 1H, CHO), 7.48 (d, *J* = 7.8 Hz, 1H, H6), 7.07 (dd, *J* = 1.4, 7.8 Hz, 1H, H5), 7.06 (d, *J* = 1.4 Hz, 1H, H3), 0.26 (s, 9H, $Si(CH_3)_3$).

^{13}C{^{1}H} NMR ($CDCl_3$, 126 MHz) δ / ppm: 195.96 (CHO), 161.38 (C2), 133.49 (C6), 131.80 (C4), 123.45 (C5), 120.94 (C3), 120.35 (C1), 103.65 (Cα), 99.82 (Cβ), -0.13 ($Si(CH_3)_3$).

FTIR (ATR, neat) ν / cm^{-1}: 3215 (OH), 2956, 2895, 2159 (C≡C), 1652 (C=O), 1618.

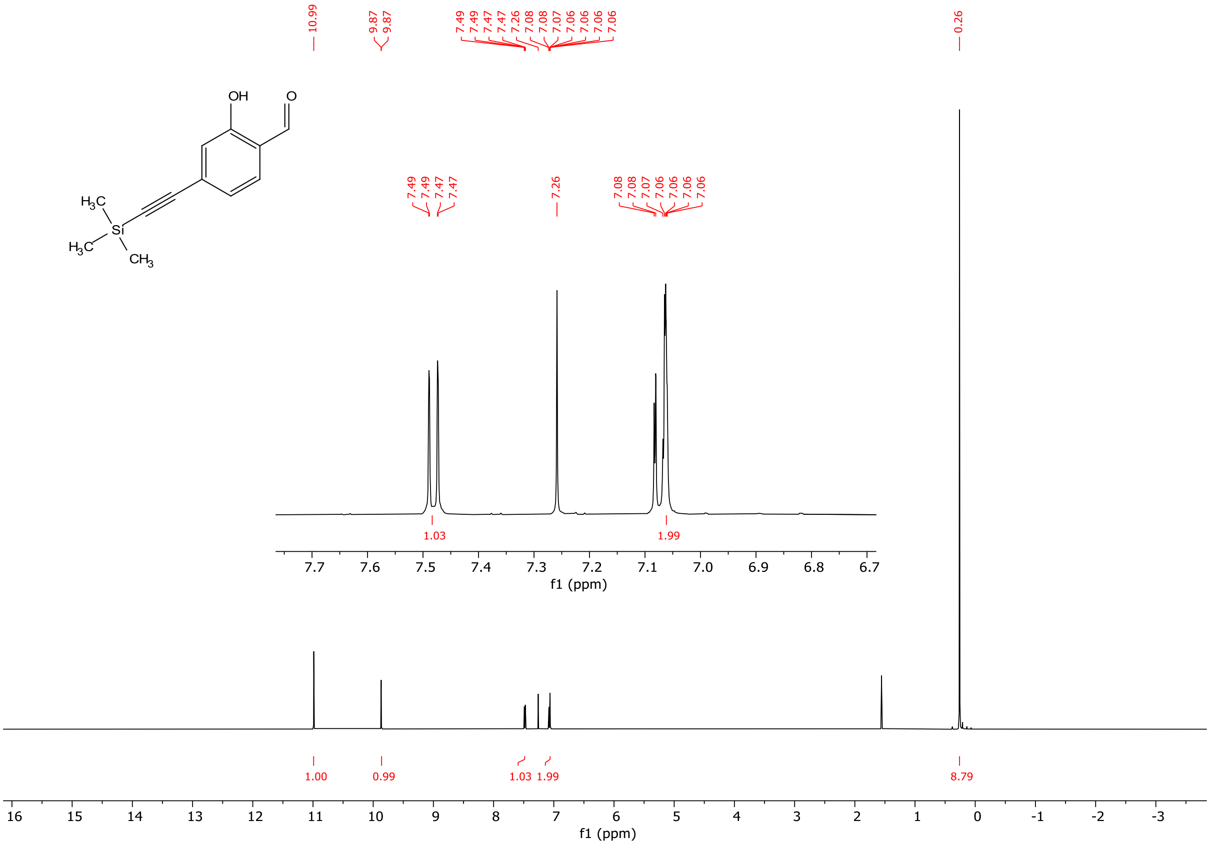

Figure S3 ^{1}H NMR Spectrum of **3** in $CDCl_3$.

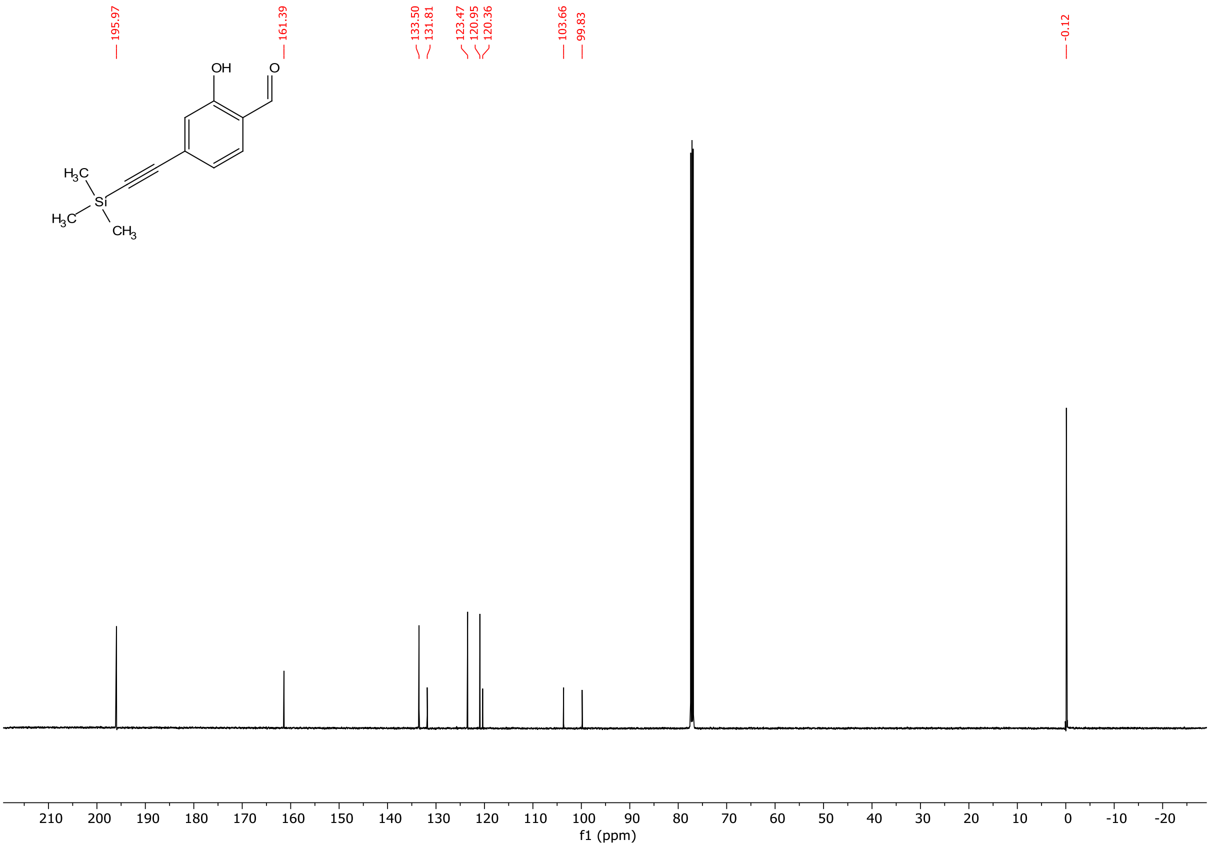

Figure S4 ^{13}C NMR Spectrum of **3** in $CDCl_3$.

1′,3′,3′-trimethyl-5′,7-bis((trimethylsilyl)ethynyl)spiro[chromene-2,2′-indoline] (**5**):

1,2,3,3-tetramethyl-5-((trimethylsilyl)ethynyl)-3*H*-indol-1-ium iodide (**1**) (742 mg, 1.87 mmol) and 2-hydroxy-4-((trimethylsilyl)ethynyl)benzaldehyde (**3**) (492 mg, 2.25 mmol) were charged into a reflux set-up. EtOH (20 mL, deoxygenated by argon sparging) and piperidine (0.5 mL, 5 mmol) were added and the solution was refluxed for 4 h. The solvent was removed under reduced pressure. And the residue was subject to flash column chromatography (alumina, 50% CH_2Cl_2/Hexanes) to give **5** as a pale-yellow powder (802 mg, 91%).

Anal. Calcd for $C_{29}H_{35}NOSi_2$: C, 74.15. H, 7.51. Found: C, 73.92. H, 7.60.

m.p.: 126 – 129 °C.

^{1}H NMR ($CDCl_3$, 500 MHz) δ /ppm: 7.31 (dd, *J* = 1.5, 8.1 Hz, 1H, H6′), 7.16 (d, *J* = 1.5 Hz, 1H, H4′), 6.96 (d, *J* = 7.8 Hz, 1H, H5), 6.93 (dd, *J* = 1.4, 7.8 Hz, 1H, H6), 6.83 (d, *J* = 10.3 Hz, 1H, H4) 6.80 (s, 1H, H8), 6.40 (d, *J* = 8.1 Hz, 1H, H7′), 5.66 (d, *J* = 10.2 Hz, 1H, H3), 2.70 (s, 3H, NCH_3), 1.26 (s, 3H, *gem*-CH_3), 1.13 (s, 3H, *gem*-CH_3) 0.24 (s, 9H, $Si(CH_3)_3$), 0.21 (s, 9H, $Si(CH_3)_3$).

^{13}C{^{1}H} NMR ($CDCl_3$, 125 MHz) δ / ppm: 153.88 (C8a), 148.52 (C7a′), 136.84 (C3a′), 132.55 (C6′), 129.28 (C4), 126.63 (C5), 125.50 (C4′), 124.27 (C7), 124.15 (C6), 119.81 (C3), 119.30 (C4a), 118.55 (C8), 113.22 (C5′), 106.85 (Cα′), 106.56 (C7′), 104.93 (Cα), 104.24 (C2), 94.95 (Cβ′), 91.25 (Cβ), 51.70 (C3), 28.88 (NCH_3), 25.84 (*gem*-CH_3), 20.20 (*gem*-CH_3), 0.35 $Si(CH_3)_3$), 0.04 ($Si(CH_3)_3$).

FTIR (ATR, neat) ν / cm^{-1}: 2958, 2149 (C≡C), 1648 (C=C), 1609, 1549, 1486, 1433, 1384, 1351, 1248, 1154, 1100, 1016, 982 (C-O).

HRMS (TOF ES(+)) *m/z*: $[M+H]^+$ Calcd for $C_{29}H_{36}NOSi_2^+$ 470.2330. Found 470.2335.

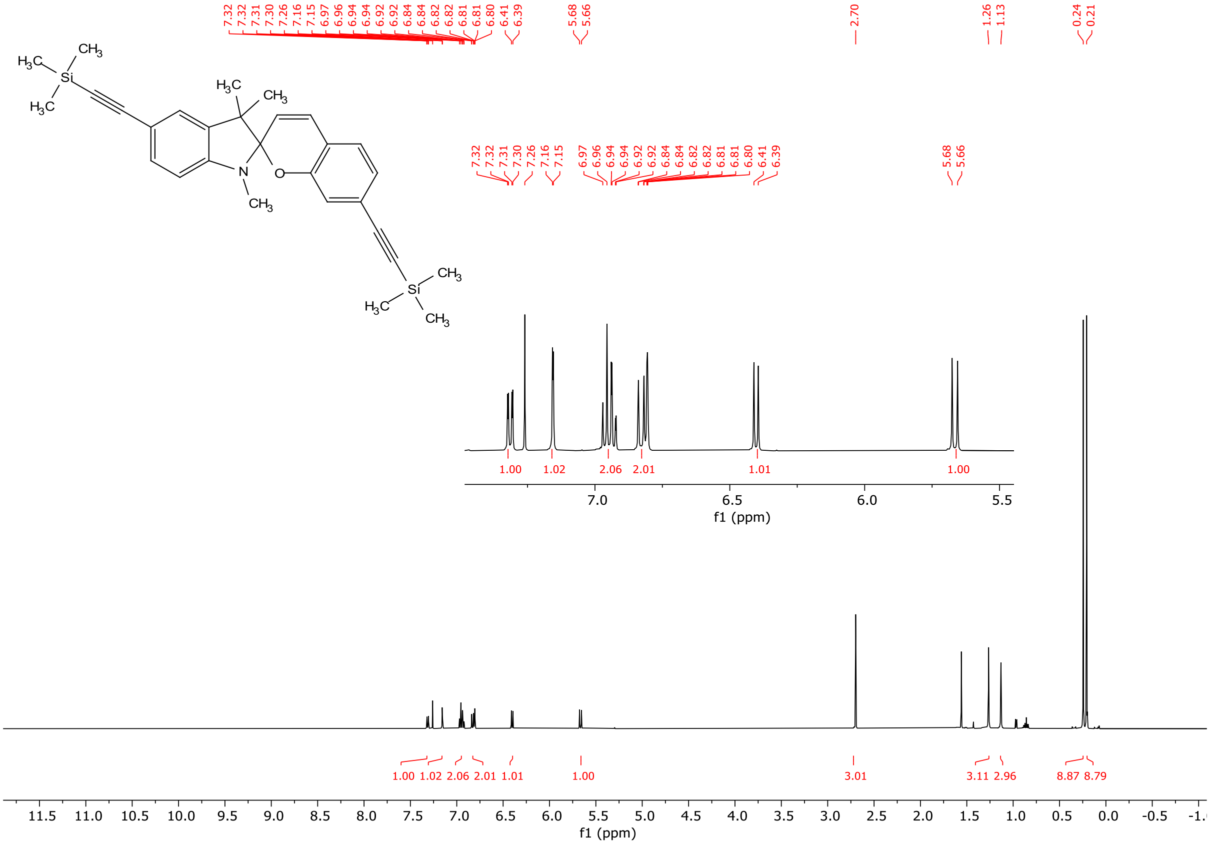

Figure S5 ^{1}H NMR Spectrum of **5** in $CDCl_3$.

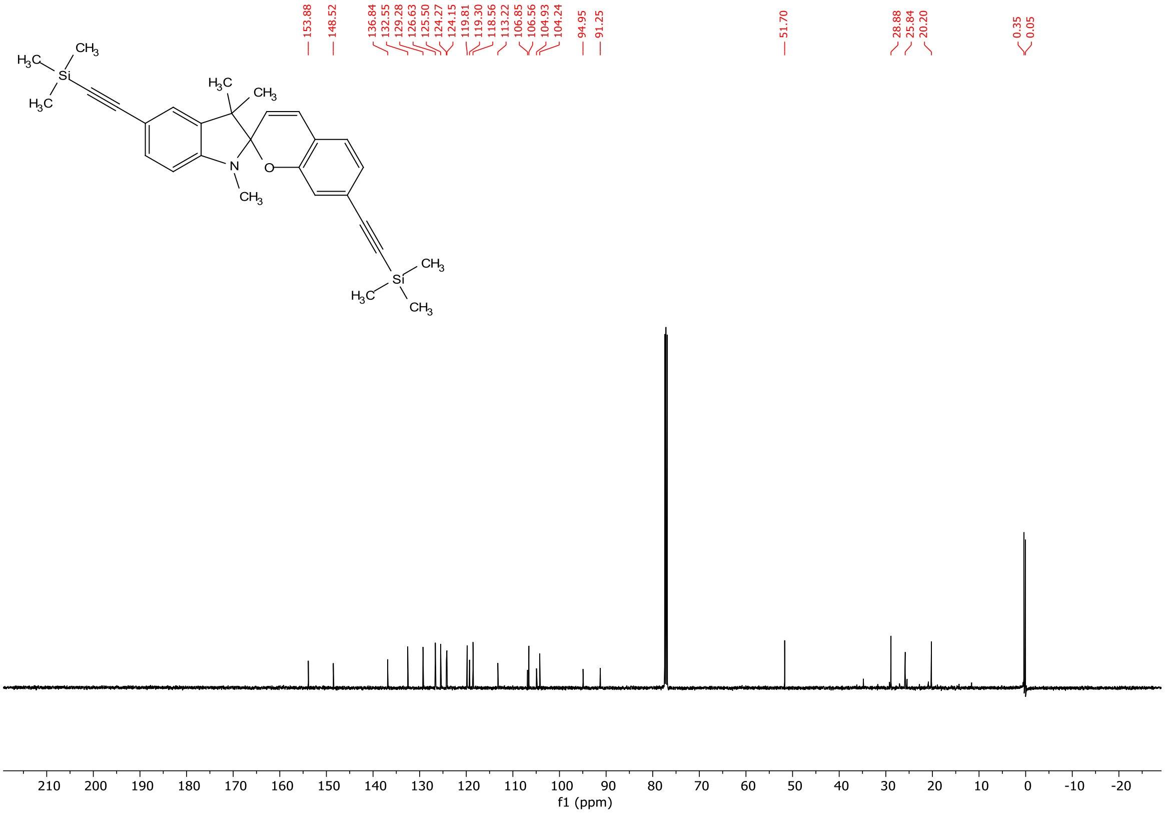

Figure S6 ^{13}C NMR Spectrum of **5** in $CDCl_3$.

5′,7-diethynyl-1′,3′,3′-trimethylspiro[chromene-2,2′-indoline] (**7**):

1′,3′,3′-trimethyl-5′,7-bis((trimethylsilyl)ethynyl)spiro[chromene-2,2′-indoline] (**5**) (430 mg, 0.915 mmol) was dissolved in THF (20 mL). Then 1M TBAF in THF (2.6 mL) was added and the solution was stirred for 90 min under argon. The solvent was removed *in vacuo*. The residue was dissolved in CH_2Cl_2 (50 mL) and washed with water (3 x 50 mL). The organic layer was dried over sodium sulfate, filtered, and evaporated to dryness. Flash column chromatography (50% CH_2Cl_2 in hexanes) gives a pale yellow powder of **7** after solvent evaporation (272 mg, 91%).

Anal. Calcd for $C_{23}H_{19}NO$: C, 84.89. H, 5.89. N, 4.30. Found: C, 84.71. H, 5.79. N, 4.13.

m.p.: 132-135 °C.

^{1}H NMR ($CDCl_3$, 600 MHz) δ /ppm: 7.35 (dd, *J* = 1.6, 8.0 Hz, 1H, H6′), 7.18 (d, *J* = 1.6 Hz, 1H, H4′), 6.99 (d, *J* = 7.7 Hz, 1H, H5), 6.96 (dd, *J* = 1.2, 7.7 Hz, 1H, H6), 6.85 (d, *J* = 10.2 Hz, 1H, H4), 6.84 (s (br.), 1H, H8), 6.44 (d, *J* = 8.0 Hz, 1H, H7′), 5.69 (d, *J* = 10.2 Hz, 1H, H3), 3.03 (s, 1H, CC-H), 2.99 (s, 1H, CC-H), 2.73 (s, 3H, NCH_3), 1.28 (s, 3H, *gem*-CH_3), 1.15 (s, 3H, *gem*-CH_3).

^{13}C{^{1}H} NMR ($CDCl_3$, 151 MHz) δ / ppm: 153.94 (C8a), 148.69 (C7a′), 136.92 (C3a), 132.69 (C6′), 129.26 (C4), 126.75 (C5), 125.61 (C4′), 124.41 (C6), 123.28 (C7), 120.02 (C4a), 119.54 (C3), 118.68 (C8), 112.18 (C5′), 106.64 (C7′), 104.35 (C2), 85.14 (Cα′), 83.50 (Cα), 77.72 (Cβ), 74.83 (Cβ′), 51.79 (C3′), 28.92 (NCH_3), 25.88 (*gem*-CH_3), 20.17 (*gem*-CH_3).

FTIR (ATR, neat) ν / cm^{-1}: 3272 (C≡C-H), 2962, 2099 (C≡C), 1643 (C=C), 1609, 1550, 1485, 1421, 1384, 1352, 1309, 1264, 1219, 1184, 1141, 1117, 1102, 1058, 1016, 977 (C-O).

HRMS (TOF ES(+)) *m/z*: $[M+H]^+$ Calcd for $C_{23}H_{20}NO^+$ 326.1540. Found 326.1546.

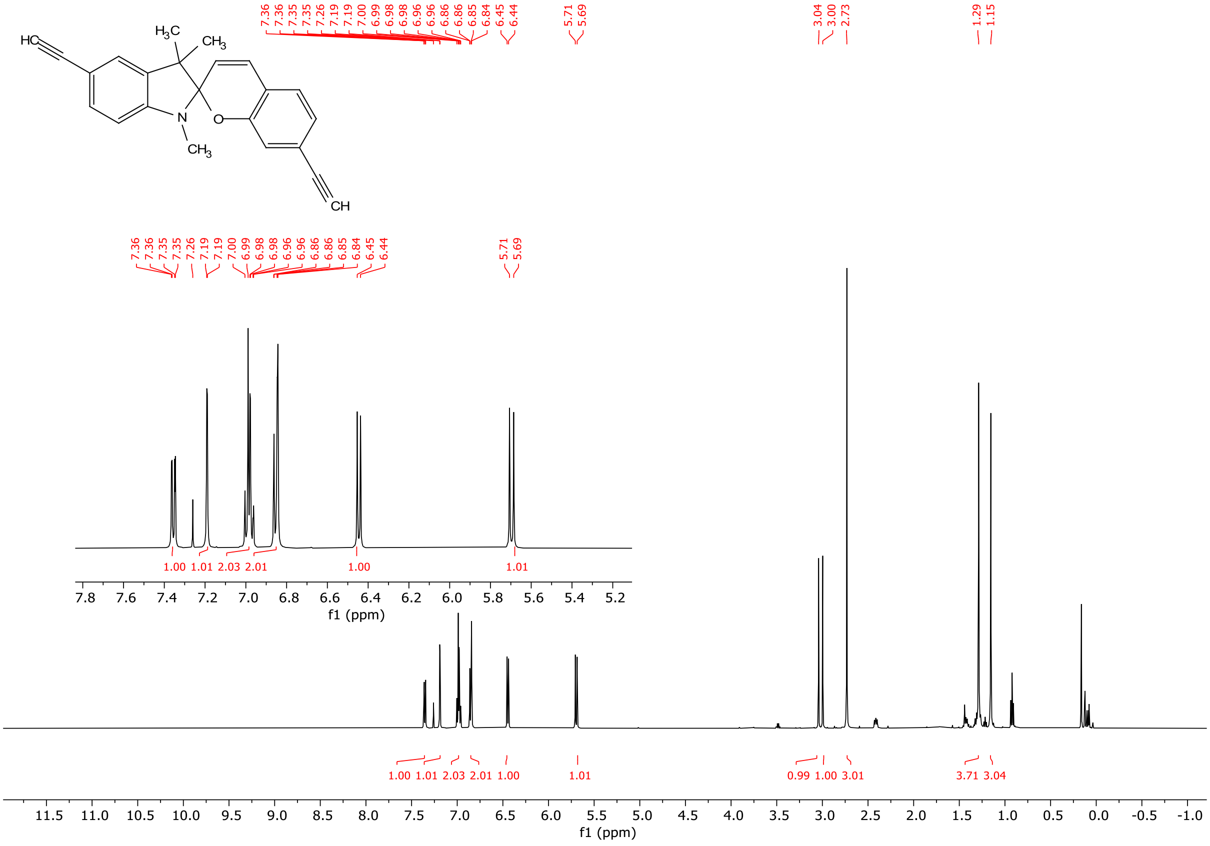

Figure S7 ^{1}H NMR Spectrum of **7** in $CDCl_3$.

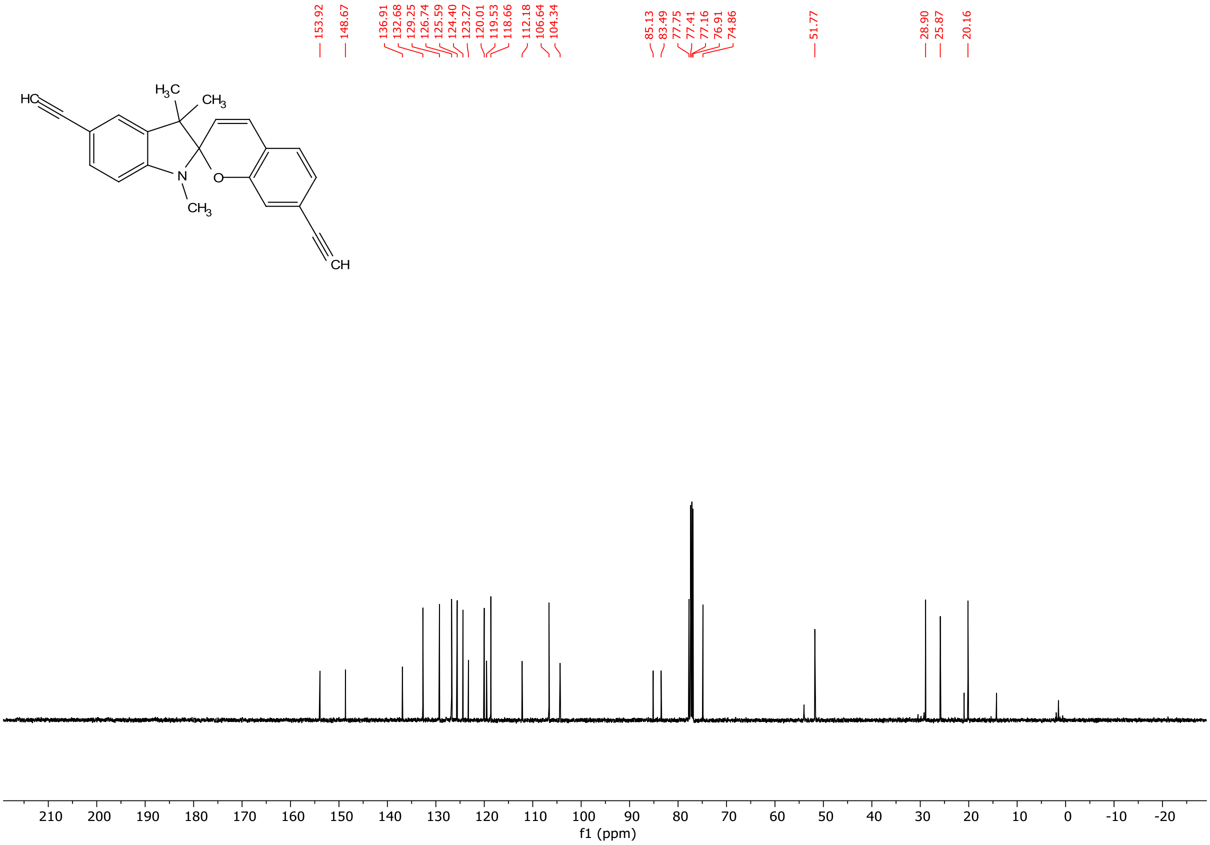

Figure S8 ^{13}C NMR Spectrum of **7** in $CDCl_3$.

1′,3′,3′-trimethyl-5′,7-bis(pyridin-4-ylethynyl)spiro[chromene-2,2′-indoline] (**SP2**):

5′,7-diethynyl-1′,3′,3′-trimethylspiro[chromene-2,2′-indoline] (**7**) (49 mg, 0.15 mmol) was charged to a Schlenk flask with THF (20 mL), NEt_3 (5 mL), 4-iodopyridine (80 mg, 0.39 mmol), $Pd(PPh_3)_2Cl_2$ (15 mg, 0.021 mmol) and CuI (6 mg, 0.03 mmol). The solution was stirred in the dark under argon for 3 d. The orange solution was evaporated to dryness and the orange residue was subject to flash column chromatography (silica, 45:50:5 EtOAc:hexanes:NEt_3) to give **SP2** as a pale yellow powder (45 mg, 63%).

^{1}H NMR ($CDCl_3$, 500 MHz) δ /ppm: 8.57 (d, *J* = 5.7 Hz, 2H, pyr-2,2′), 8.56 (d, *J* = 5.7 Hz, 2H, pyr-3′), 7.42 (dd, *J* = 1.0, 8.0 Hz, 1H, H6′), 7.35 (d, *J* = 5.7 Hz, 2H, pyr-3), 7.31 (d, *J* = 5.7 Hz, 2H, pyr-3), 7.25 (s (br.), 1H, H4′), 7.06 (d, *J* = 7.7 Hz, 1H, H5), 7.04 (d, *J* = 7.7 Hz, 1H, H6), 6.92 (s (br.), 1H, H8), 6.89 (d, *J* = 10.1, 1H, H4), 6.50 (d, *J* = 8.0 Hz, 1H, H7′), 5.74 (d, *J* = 10.1 Hz, 1H, H3), 2.77 (s, 3H, NCH_3), 1.33 (s, 3H, *gem*-CH_3), 1.19 (s, 3H, *gem*-CH_3).

^{13}C{^{1}H} NMR ($CDCl_3$, 126 MHz) δ / ppm: 154.00 (C8a), 149.88 (pyr), 149.76 (pyr), 149.10 (C7a′), 137.15 (C3a′), 132.79 (C6′), 132.39 (pyr), 131.42 (pyr), 129.36 (C4), 126.97 (C5), 125.61 (pyr), 125.43 (C4′), 125.40 (pyr), 124.26 (C6), 123.25 (C7), 120.21 (C4a), 119.89 (C3), 118.38 (C8), 112.14 (C5′), 106.84 (C7′), 104.39 (C2), 96.10 (Cα′), 93.85 (Cα), 87.28 (Cβ), 85.19 (Cβ′), 51.85 (C3′), 28.93 (NCH_3), 25.90 (*gem*-CH_3), 20.21 (*gem*-CH_3).

FTIR (ATR, neat) ν / cm^{-1}: 2214 (C≡C), 2193 (C≡C), 1639 (C=C), 1609, 1588, 1537, 1493, 938 (C-O).

HRMS (TOF ES(+)) *m/z*: $[M+H]^+$ Calcd for $C_{33}H_{26}N_3O$+ 480.2071; Found 480.2076.

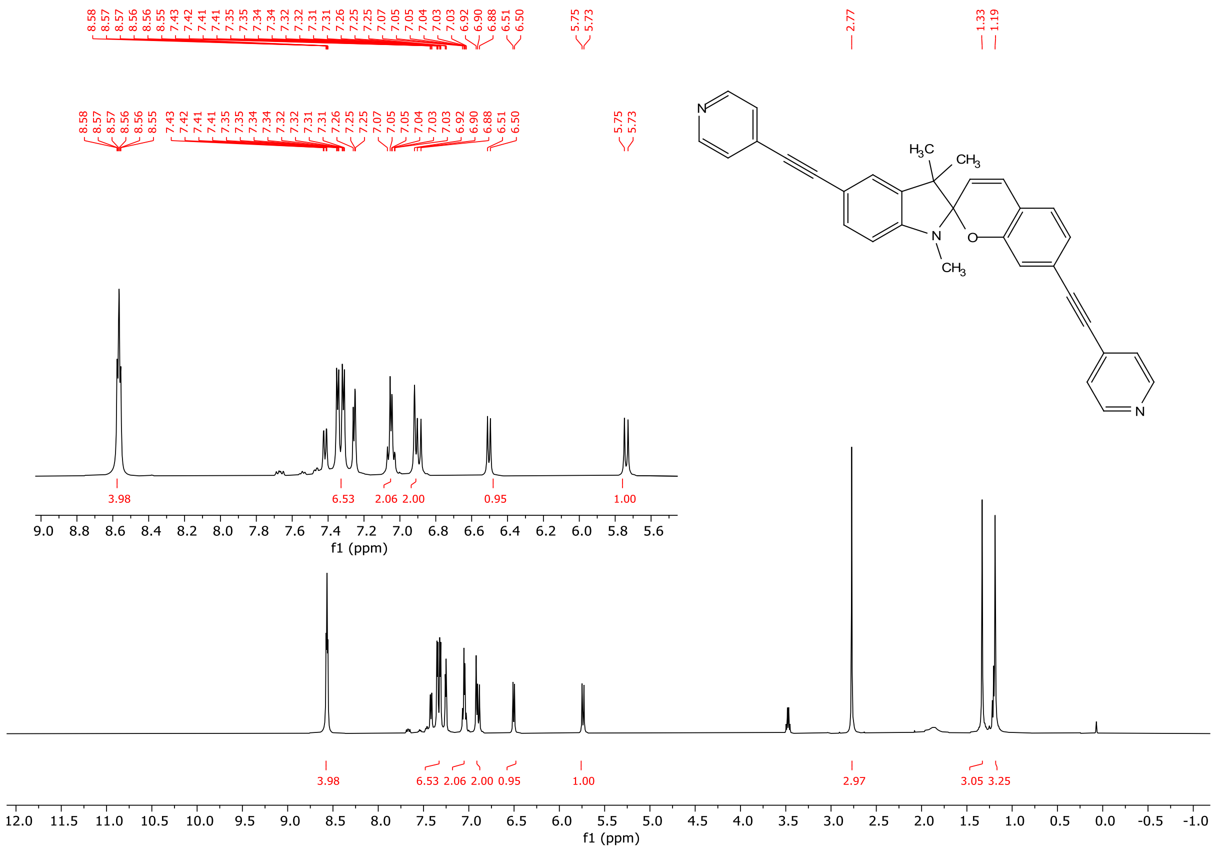

Figure S9 ^{1}H NMR Spectrum of **SP2** in $CDCl_3$.

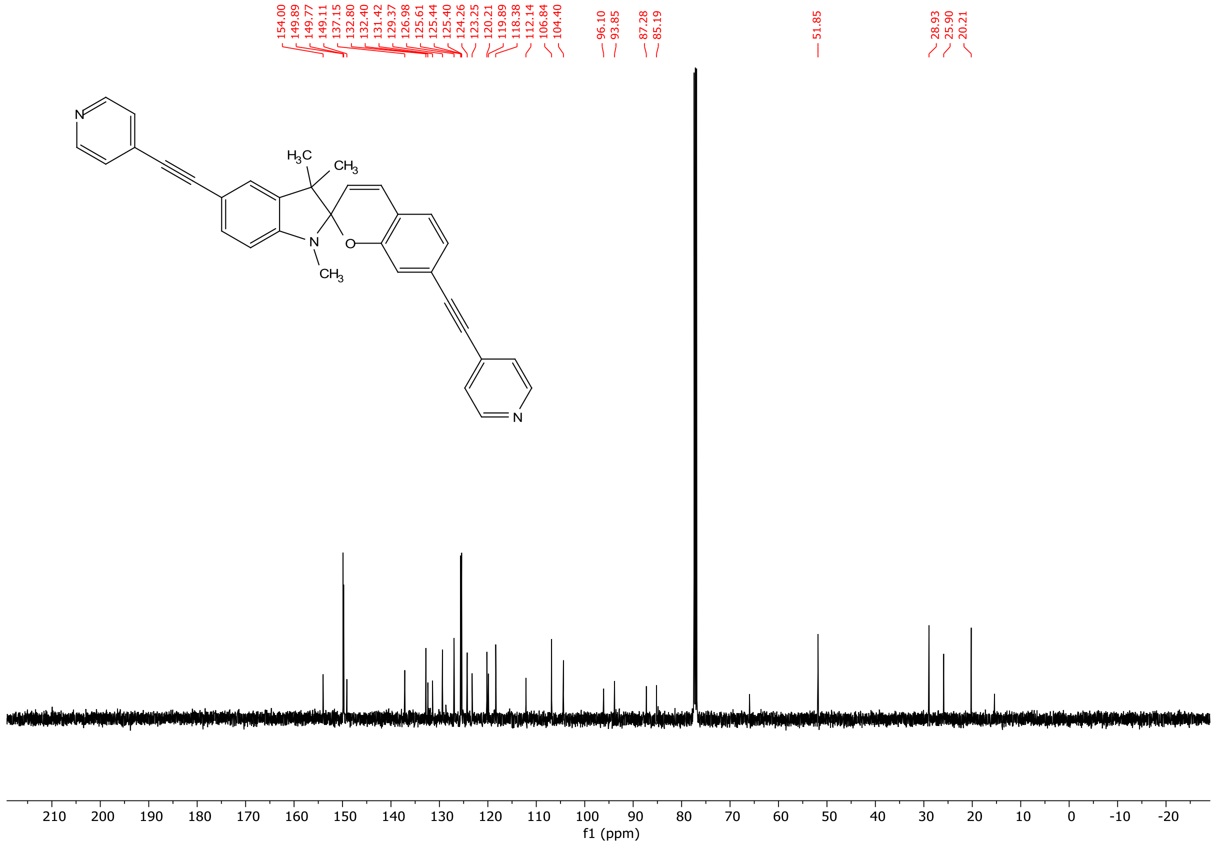

Figure S10 ^{13}C NMR Spectrum of **SP2** in $CDCl_3$.

1′,3′,3′-trimethyl-5′,6-bis((4-(methylthio)phenyl)ethynyl)spiro[chromene-2,2′-indoline] (**SP3**): 5′,6-diethynyl-1′,3′,3′-trimethylspiro[chromene-2,2′-indoline] (**6**) (48 mg, 0.15 mmol), (4-iodophenyl)(methyl)sulfane (115 mg, 0.460 mmol), $Pd(PPh_3)_2Cl_2$ (38 mg, 0.054 mmol) and CuI (27 mg, 0.14 mmol) were evacuated in a Schlenk flask for 30 min. After refilling with argon, THF (20 mL) and $HN(iPr)_2$ (2 mL) were added. The reaction vessel was covered in foil and stirred for 4 days at rt. The solvent was removed in vacuo and the orange residue was subject to preparative TLC (5% NEt_3, 10%, EtOAc, 85% Hexanes) to give **SP3** (37 mg, 43%).

^{1}H NMR ($CDCl_3$, 400 MHz) δ /ppm: 7.42 (d, *J* = 8.5 Hz, 2H, thio-3), 7.40 (d, *J* = 8.4 Hz, 2H, thio-3′), 7.38 (dd, *J* = 1.6, 8.0 Hz, 1H, H6′), 7.27 (dd, *J* = 1.9, 8.3 Hz, 1H, H7), 7.24 (d, *J* = 1.9 Hz, 1H, H5), 7.23 (d, *J* = 1.6 Hz, H4′), 7.20 (br. d, *J* = 8.5 Hz, 4H, thio-2/2′), 6.85 (d, *J* = 10.2 Hz, 1H, H4), 6.68 (d, *J* = 8.3 Hz, 1H, H8), 6.49 (d, *J* = 8.0 Hz, 1H, H7′), 5.71 (d, *J* = 10.2 Hz, 1H, H3), 2.76 (s, 3H, NCH_3), 2.50 (s, 3H, SCH_3), 2.49 (s, 3H, SCH_3), 1.31 (s, 3H, *gem*-CH_3), 1.18 (s, 3H, *gem*-CH_3).

^{13}C{^{1}H} NMR ($CDCl_3$, 101 MHz) δ / ppm: 154.53 (C8a), 148.35 (C7a′), 139.02 (thio-1 or 1′), 138.44 (thio-1 or 1′), 137.02 (C3a′), 133.41 (C7), 132.04 (C6′), 131.86 (thio-3 or 3′), 131.78 (thio-3 or 3′), 130.10 (C5), 129.27 (C4), 126.17 (thio-2 or 2′), 126.10 (thio-2 or 2′), 125.11 (C4′), 120.59 (thio-4 or 4′), 120.02 (thio-4 or 4′), 119.78 (C3), 118.89 (C4a), 115.43 (C8), 115.12 (C6), 113.48 (C5′), 106.82 (C7′), 104.73 (C2), 90.95 (Cα′), 89.38 (Cα), 87.89 (Cβ′), 87.17 (Cβ), 51.84 (C3′), 28.96 (NCH_3), 25.93 (*gem*-CH_3), 20.17 (*gem*-CH_3), 15.70 (SCH_3), 15.61 (SCH_3).

FTIR (ATR, neat) ν / cm^{-1}: 2958, 2919, 2869, 2210 (br. C≡C), 1646 (C=C), 1606, 1494.

HRMS (TOF ES(+)) *m/z*: $[M+H]^+$ Calcd for $C_{37}H_{32}NOS_2^+$ 570.1920; Found 570.1921.

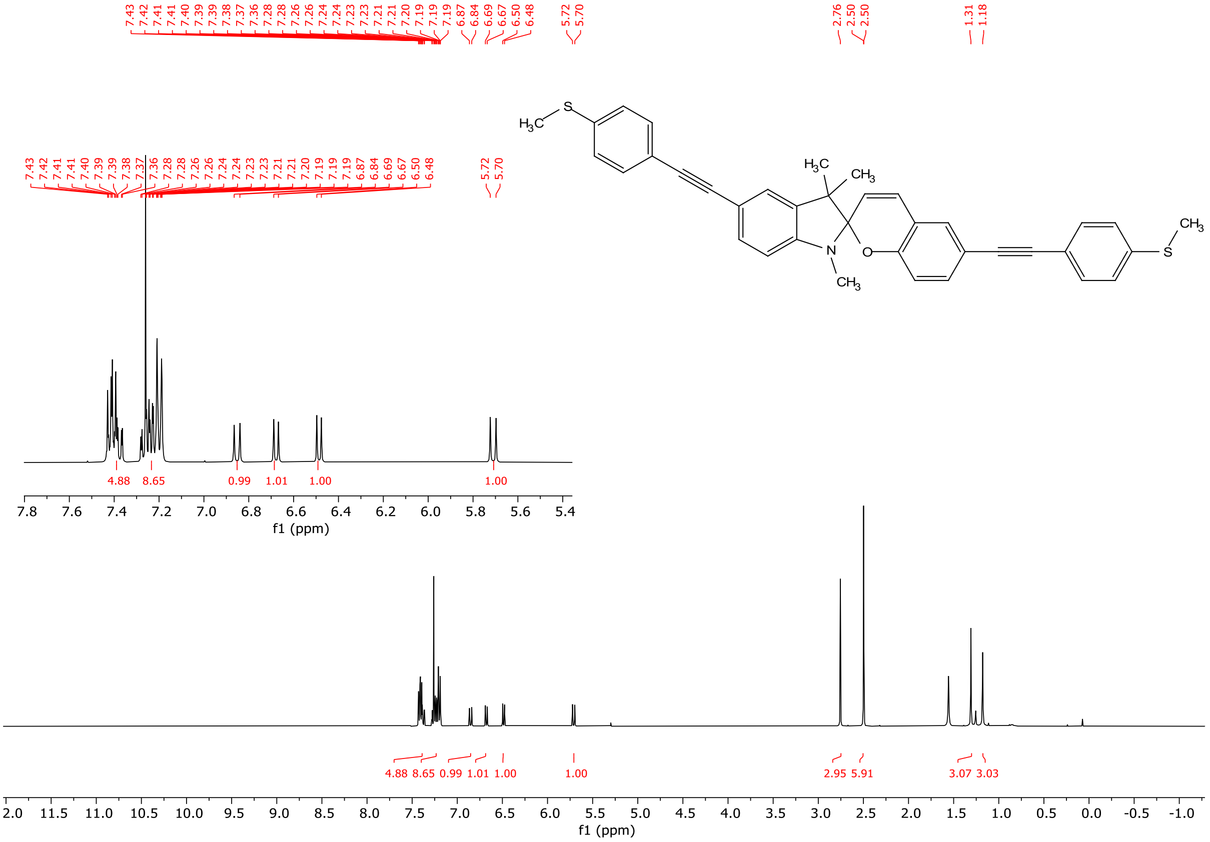

Figure S11 ^1H NMR Spectrum of **SP3** in $CDCl_3$.

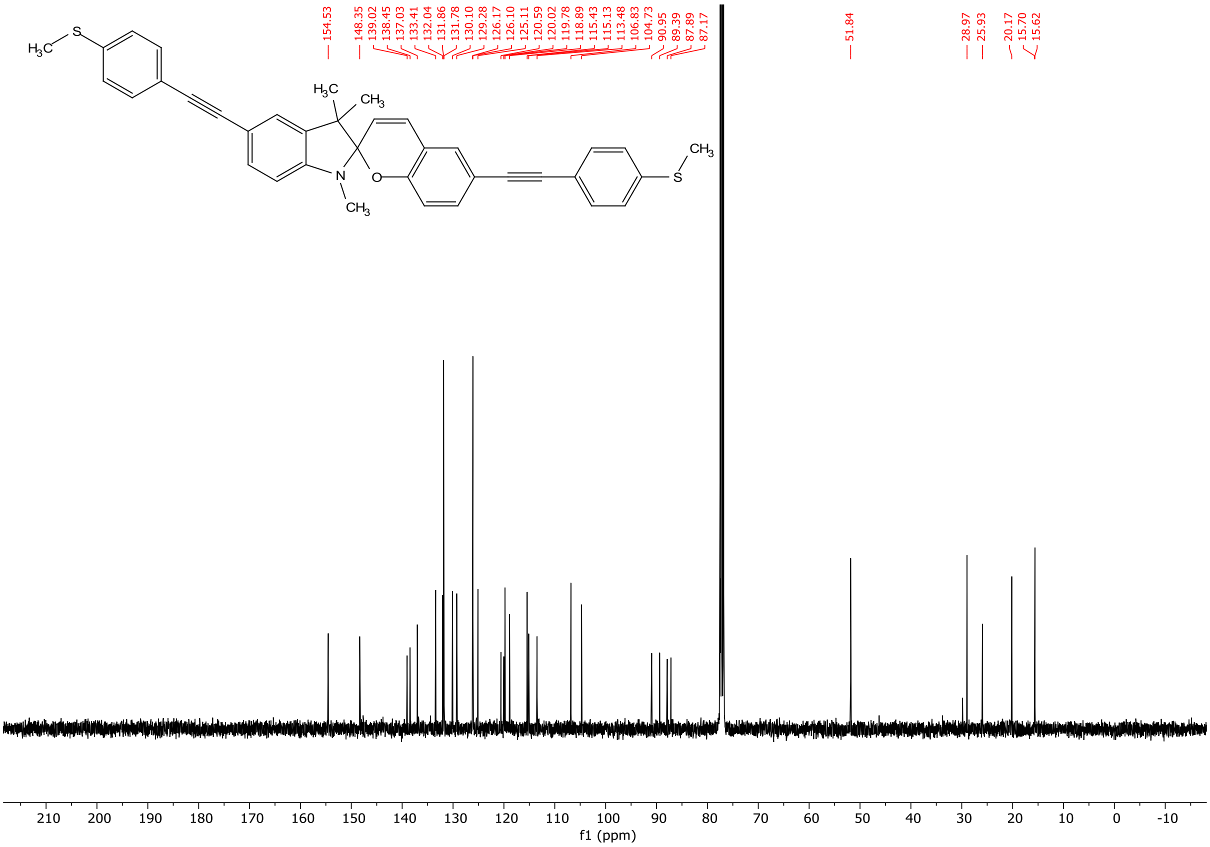

Figure S12 ^{13}C NMR Spectrum of **SP3** in $CDCl_3$.

1′,3′,3′-trimethyl-5′,7-bis((4-(methylthio)phenyl)ethynyl)spiro[chromene-2,2′-indoline] (**SP4**): 5′,7-diethynyl-1′,3′,3′-trimethylspiro[chromene-2,2′-indoline] **7** (48 mg, 0.15 mmol), (4-iodophenyl)(methyl)sulfane (110 mg, 0.440 mmol), $Pd(PPh_3)_2Cl_2$ (34 mg, 0.048 mmol) and CuI (28 mg, 0.15 mmol) were evacuated in a Schlenk flask for 30 min. After refilling with argon, THF (20 mL) and $HN(iPr)_2$ (2 mL) were added. The reaction vessel was covered in foil and stirred for 4 days at rt. The solvent was removed *in vacuo* and the orange residue taken up in EtOAc (50 mL) and washed with water (3 x 50 mL) and brine (50 mL). The organic layer was dried over Na_2SO_4, filtered and evaporated. The crude product was subject to preparative TLC (5% NEt_3, 10%, EtOAc, 85% Hexanes) to give **SP4** as a yellow powder (30 mg, 35%).

^{1}H NMR ($CDCl_3$, 400 MHz) δ /ppm: 7.42 (d, *J* = 8.5 Hz, 2H, thio-2), 7.38 (d, *J* = 8.5 Hz, 2H, thio-2′), 7.37 (dd, *J* = 1.7, 8.0 Hz, 1H, H6′), 7.22 (d, *J* = 1.7 Hz, 1H, H4′), 7.20 (d, *J* = 8.5 Hz, 2H, thio-3), 7.18 (d, *J* = 8.5 Hz, 2H, thio-3′), 7.02 (d, *J* = 7.8 Hz, 1H, H5), 6.99 (dd, *J* = 1.3, 7.8 Hz, 1H, H6), 6.88 (d, *J* = 1.3 Hz, 1H, H8), 6.86 (d, *J* = 10.4 Hz, 1H, H4), 6.48 (d, *J* = 8.0 Hz, 1H, H7′), 5.70 (d, *J* = 10.4 Hz, 1H, H3), 2.75 (s, 3H, NCH_3), 2.50 (s, 3H, SCH_3), 2.48 (s, 3H, $S'CH_3$), 1.32 (s, 3H, *gem*-CH_3), 1.18 (s, 3H, *gem*-CH_3).

^{13}C NMR ($CDCl_3$, 101 MHz) δ / ppm: 154.07 (C8a), 148.40 (C7a′), 139.53 (thio-4′), 138.40 (thio-4), 137.08 (C3a′), 132.01 (thio-2′ and C6′), 131.78 (thio-2), 129.35 (C4), 126.79 (C5), 126.18 (thio-3), 125.99 (thio-3′), 125.09 (C4′), 124.53 (C7), 123.81 (C6), 120.63 (thio-1), 119.74 (C3), 119.57 (thio-1′), 119.06 (C4a), 118.04 (C8), 113.39 (C5′), 106.78 (C7′), 104.35 (C2), 91.03 (Cα′), 89.97 (Cβ′), 89.52 (Cα), 87.12 (Cβ), 51.81 (C3′), 28.97 (NCH_3), 25.93 (*gem*-CH_3), 20.27 (*gem*-CH_3), 15.71 (SCH_3), 15.52 ($S'CH_3$).

FTIR (ATR, neat) ν / cm^{-1}: 2921, 2207 (br. C≡C), 1647 (C=C), 1608, 1545, 1496.

HRMS (TOF ES(+)) *m/z*: $[M+H]^+$ Calcd for $C_{37}H_{32}NOS_2^+$ 570.1920; Found 570.1929.

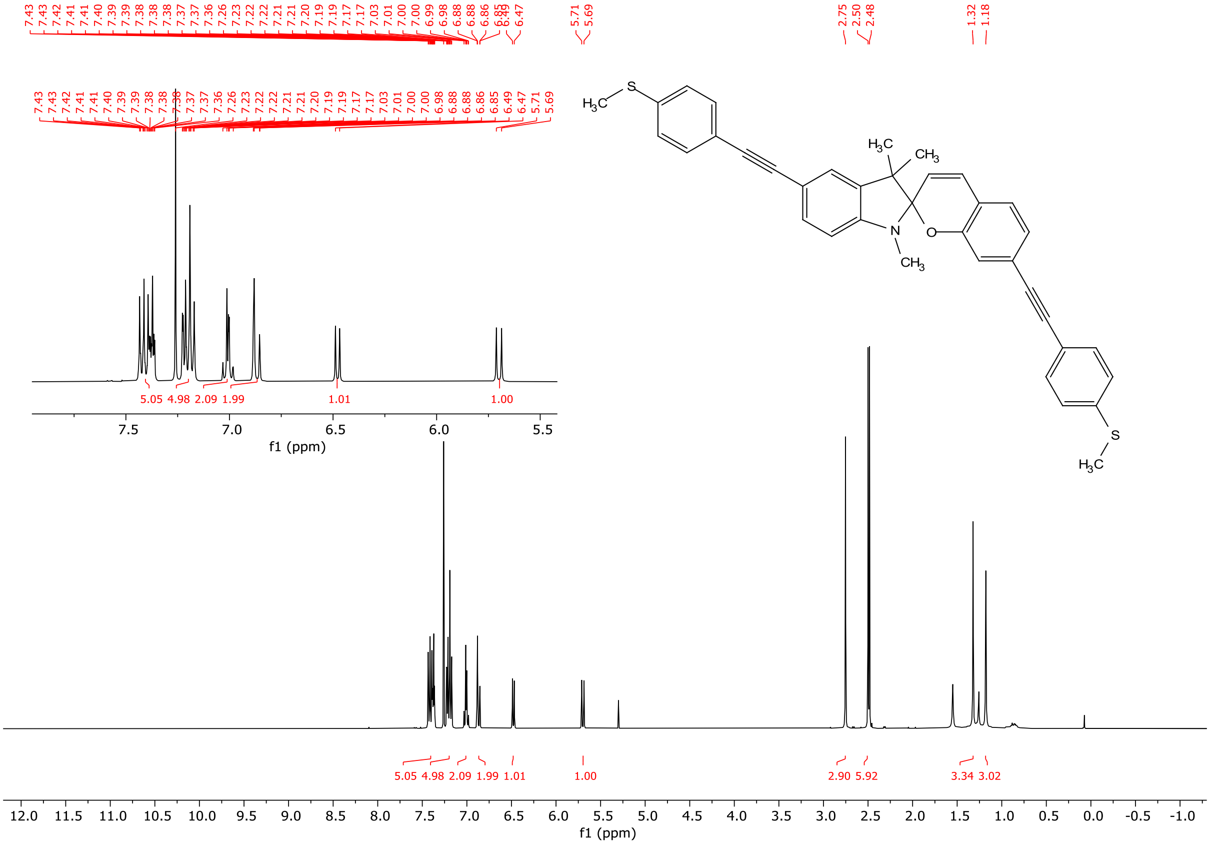

Figure S13 ^{1}H NMR Spectrum of **SP4** in $CDCl_3$.

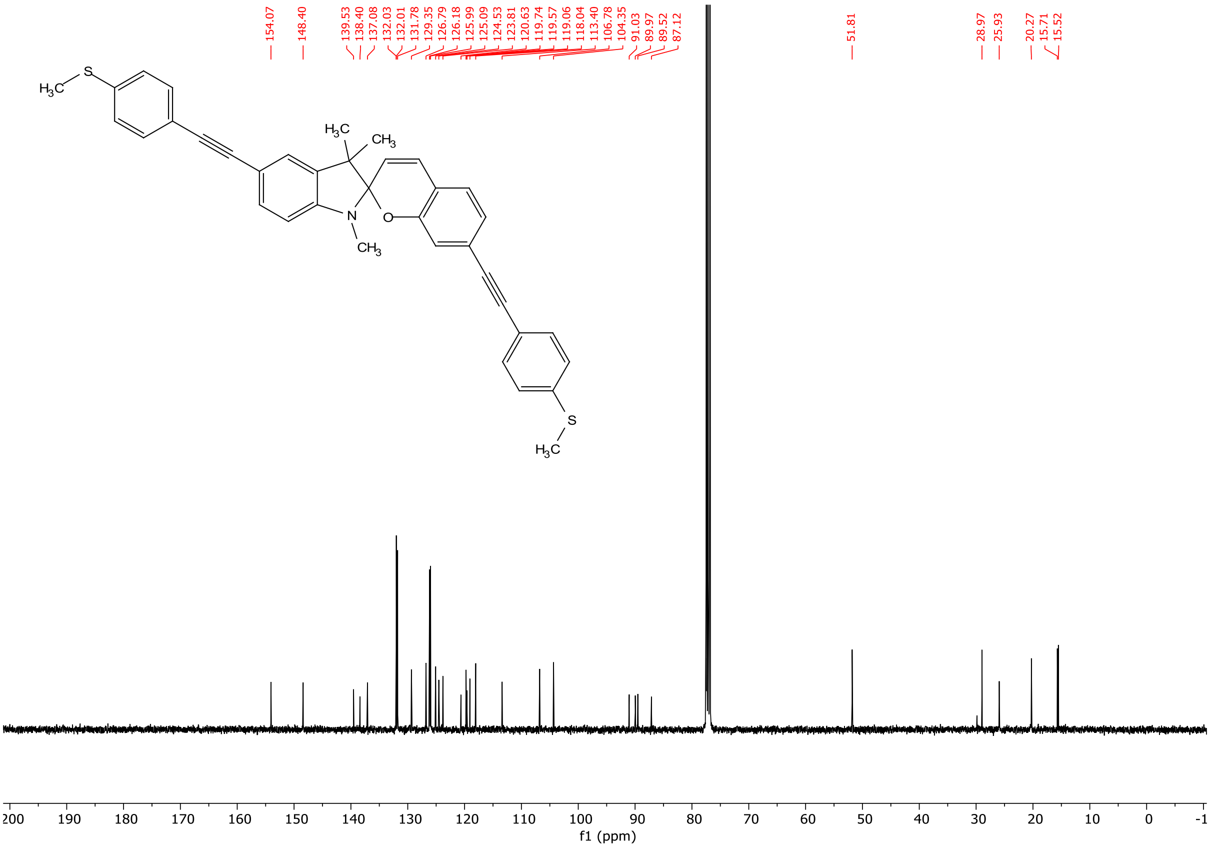

Figure S14 ^{13}C NMR Spectrum of **SP4** in $CDCl_3$.

1′,3′,3′-trimethyl-5′,6-bis(thiophen-3-ylethynyl)spiro[chromene-2,2′-indoline] (**SP5**):

5′,6-diethynyl-1′,3′,3′-trimethylspiro[chromene-2,2′-indoline] (**6**) (50 mg, 0.15 mmol) was charged to a Schlenk flask with THF (20 mL), NEt_3 (5 mL), 3-iodothiophene (0.05 mL, 0.100 mg 0.5 mmol), $Pd(PPh_3)_2Cl_2$ (19 mg, 0.027 mmol) and CuI (12 mg, 0.063 mmol). The solution was stirred in the dark under argon for 3 d. The orange solution was evaporated to dryness and the residue was subject to column chromatography (silica plug, 15:80:5 EtOAc:hexanes:NEt_3) to give a yellow oil. The pure product was isolated from preparative TLC using 5% NEt3 and 5% EtOAC in hexanes to give a white powder of **SP5** (50 mg, 68%).

^{1}H NMR ($CDCl_3$, 500 MHz) δ /ppm: 7.47 (dd, *J* = 1.1, 3.0 Hz, 1H, thio-2), 7.46 (dd, *J* = 1.1, 3.0 Hz, 1H, thio-2′), 7.37 (dd, *J* = 1.6, 8.0 Hz, 1H, H6′), 7.25 – 7.29 (m, 3H, H7, thio-5, thio-5′), 7.24 (d, *J* = 2.0 Hz, 1H, H5), 7.22 (d, *J* = 1.6 Hz, 1H, H4′), 7.18 (dd, *J* = 1.1, 5.0 Hz, 1H, thio-4), 7.17 (dd, *J* = 1.1, 5.0 Hz, 1H, thio-4′), 6.86 (d, *J* = 10.2 Hz, 1H, H4), 6.68 (d, *J* = 8.4 Hz, 1H, H8), 6.48 (d, *J* = 8.0 Hz, 1H, H7′), 5.71 (d, *J* = 10.2 Hz, 1H, H3), 2.75 (s, 3H, NCH_3), 1.31 (s, 3H, *gem*-CH_3), 1.18 (s, 3H, *gem*-CH_3).

^{13}C{^{1}H} NMR ($CDCl_3$, 126 MHz) δ / ppm: 154.51 (C8a), 148.32 (C7a′), 136.99 (C3a′), 133.37 (C7), 132.00 (C6′), 130.08, 130.05, 129.98, 129.25, 128.20, 127.67, 125.41, 125.25, 125.08, 123.10, 122.66, 119.76 (C4a), 118.86, 115.40, 115.00, 113.35, 106.80, 104.70 (C2), 90.18 (C≡C), 88.71 (C≡C), 83.16 (C≡C), 82.40 (C≡C), 51.82 (C3′), 28.94 (NCH_3), 25.91 (*gem*-CH_3), 20.15 (*gem*-CH_3).

FTIR (ATR, neat) ν / cm^{-1}: 2210 (C≡C).

HRMS (TOF AP(+)) m/z: $[M+H]^+$ Calcd for $C_{31}H_{24}NOS_2^+$ 490.1294; Found 490.1312.

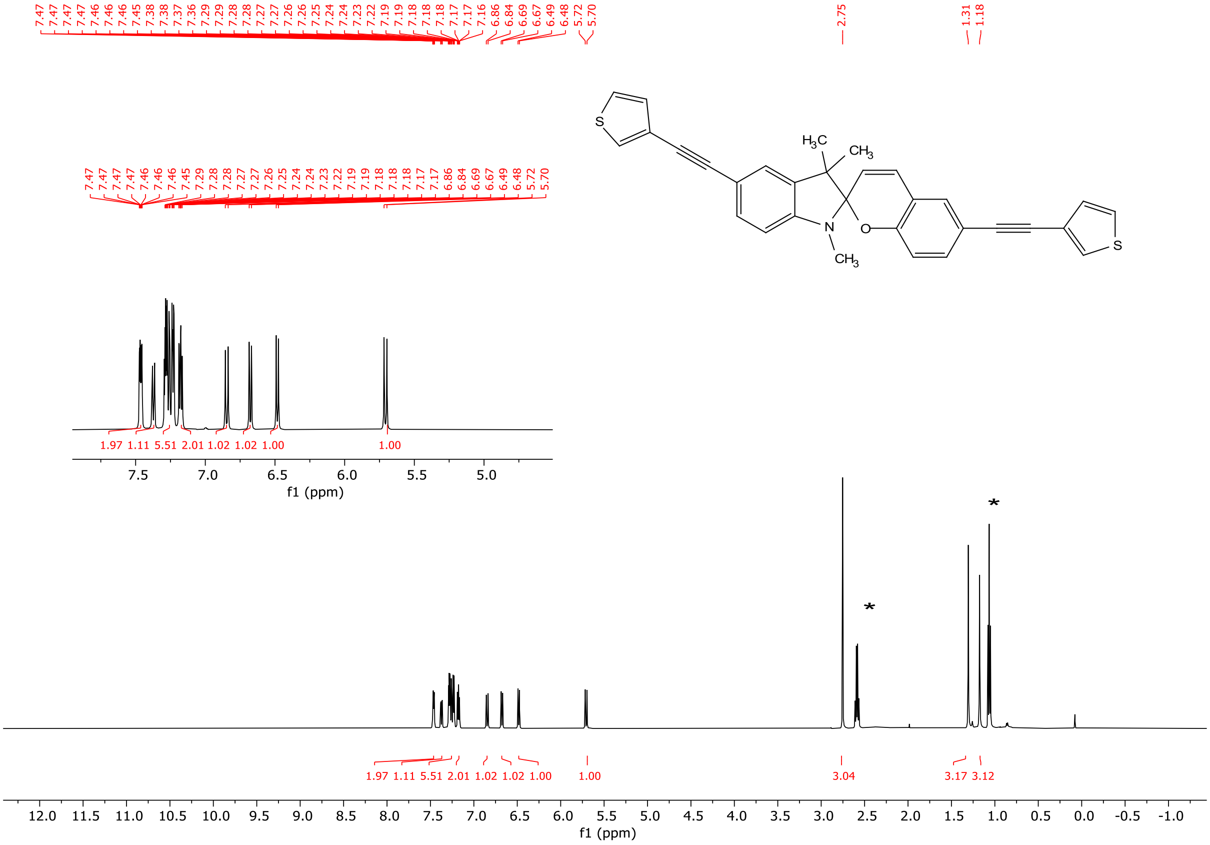

Figure S15 ^{1}H NMR Spectrum of **SP5** in $CDCl_3$.

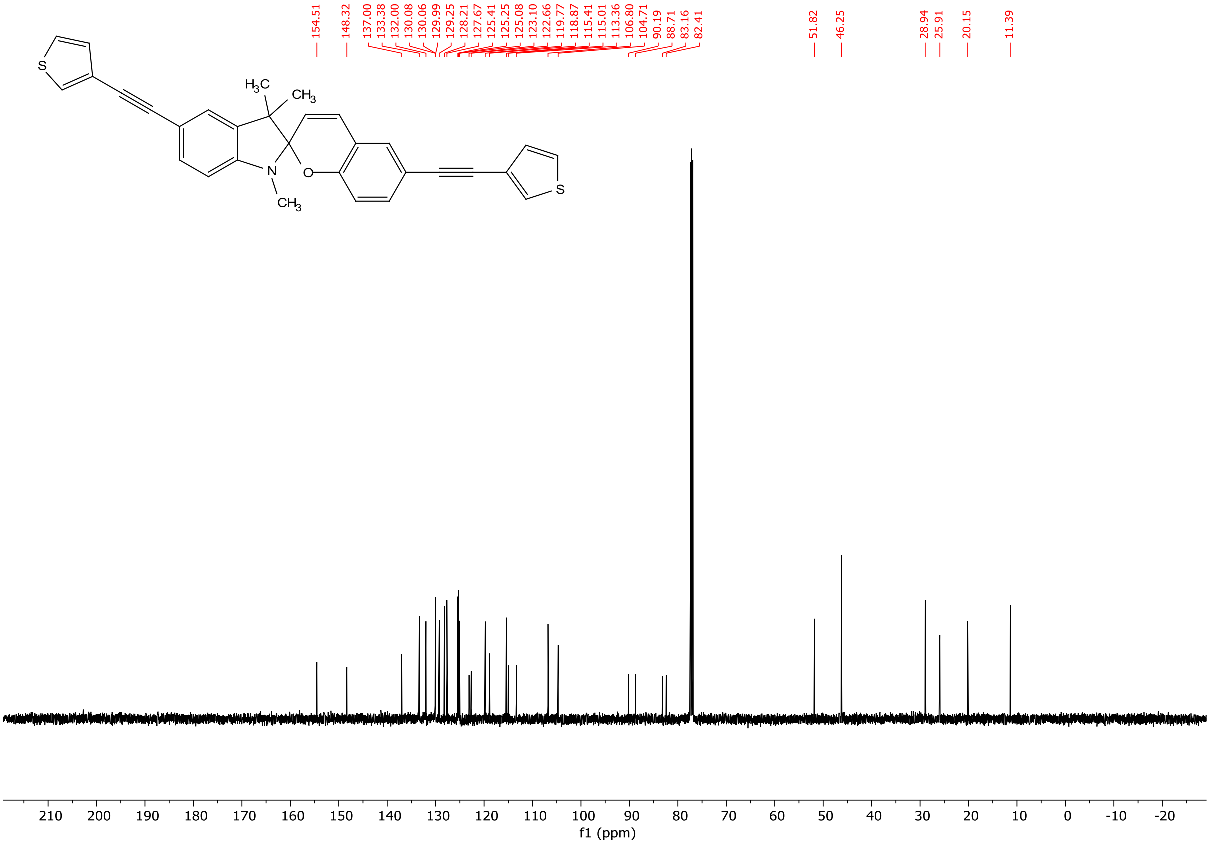

Figure S16 ^{13}C NMR Spectrum of **SP5** in $CDCl_3$.

1′,3′,3′-trimethyl-5′,7-bis(thiophen-3-ylethynyl)spiro[chromene-2,2′-indoline] (**SP6**):

5′,7-diethynyl-1′,3′,3′-trimethylspiro[chromene-2,2′-indoline] (**7**) (52 mg, 0.16 mmol) was charged to a Schlenk flask with THF (20 mL), NEt_3 (5 mL), 3-iodothiophene (0.05 mL, 0.100 mg 0.5 mmol), $Pd(PPh_3)_2Cl_2$ (10 mg, 0.014 mmol) and CuI (12 mg, 0.063 mmol). The solution was stirred in the dark under argon for 3 d. The orange solution was evaporated to dryness and the residue was subject to column chromatography (silica plug, 10:85:5 EtOAc:hexanes:NEt_3) to give an orange oil. The pure product was isolated from preparative TLC using 5% NEt_3 and 10% EtOAC in hexanes to give **SP6** as a white powder (43 mg, 55%).

^{1}H NMR ($CDCl_3$, 500 MHz) δ /ppm: 7.48 (dd, *J* = 1.1, 3.0 Hz, 1H, thio-2), 7.46 (d, *J* = 1.1, 3.0 Hz, 1H, thio-2′), 7.37 (dd, *J* = 1.5, 8.0 Hz, 1H, H6′), 7.29 (dd, *J* = 3.0, 4.9 Hz, 1H, thio-5), 7.27 (dd, *J* = 3.0, 4.9 Hz, 1H, thio-5′), 7.22 (d, *J* = 1.5 Hz, 1H, H4′), 7.19 (dd, *J* = 1.1, 4.9 Hz, 1H, thio-4), 7.15 (dd, *J* = 1.1, 4.9 Hz, 1H, thio-4′), 7.02 (d, *J* = 7.7 Hz, 1H, H5), 6.99 (dd, *J* = 1.3, 7.7 Hz, 1H, H6), 6.88 (s (br.), 1H, H8), 6.86 (d, *J* = 10.2 Hz, 1H, H4), 6.48 (d, *J* = 8.1 Hz, 1H, H7′), 5.70 (d, *J* = 10.2 Hz, 1H, H3), 2.75 (s, 3H, NCH_3), 1.32 (s, 3H, *gem*-CH_3), 1.18 (s, 3H, *gem*-CH_3).

^{13}C{^{1}H} NMR ($CDCl_3$, 126 MHz) δ / ppm: 154.05 (C8a), 148.38 (C7a′), 137.05 (C3a′), 131.99 (C6′), 130.07 (thio-4), 129.99 (thio-4′), 129.33 (C4), 128.83 (thio-2), 127.65 (thio-2′), 126.78 (C5), 125.48 (thio-5), 125.23 (thio-5′), 125.07 (C4′), 124.43 (C7), 123.78 (C6), 123.14 (thio-3), 122.33 (thio-3′), 119.76 (C3), 119.07 (C4a), 118.00 (C8), 113.27 (C5′), 106.76 (C7′), 104.34 (C2), 90.26 (Cα′), 88.88 (Cα), 85.21 (Cβ′), 82.36 (Cβ), 51.79 (C3′), 28.95 (NCH_3), 25.92 (*gem*-CH_3), 20.25 (*gem*-CH_3).

FTIR (ATR, neat) ν / cm^{-1}: 3105, 2961, 2208 (C≡C), 1732, 1641 (C=C), 1608, 1547, 1523, 1486, 946 (C-O).

HRMS (TOF ES(+)) *m/z*: $[M+H]^+$ Calcd for $C_{31}H_{24}NOS_2^+$ 490.1294; Found 490.1292.

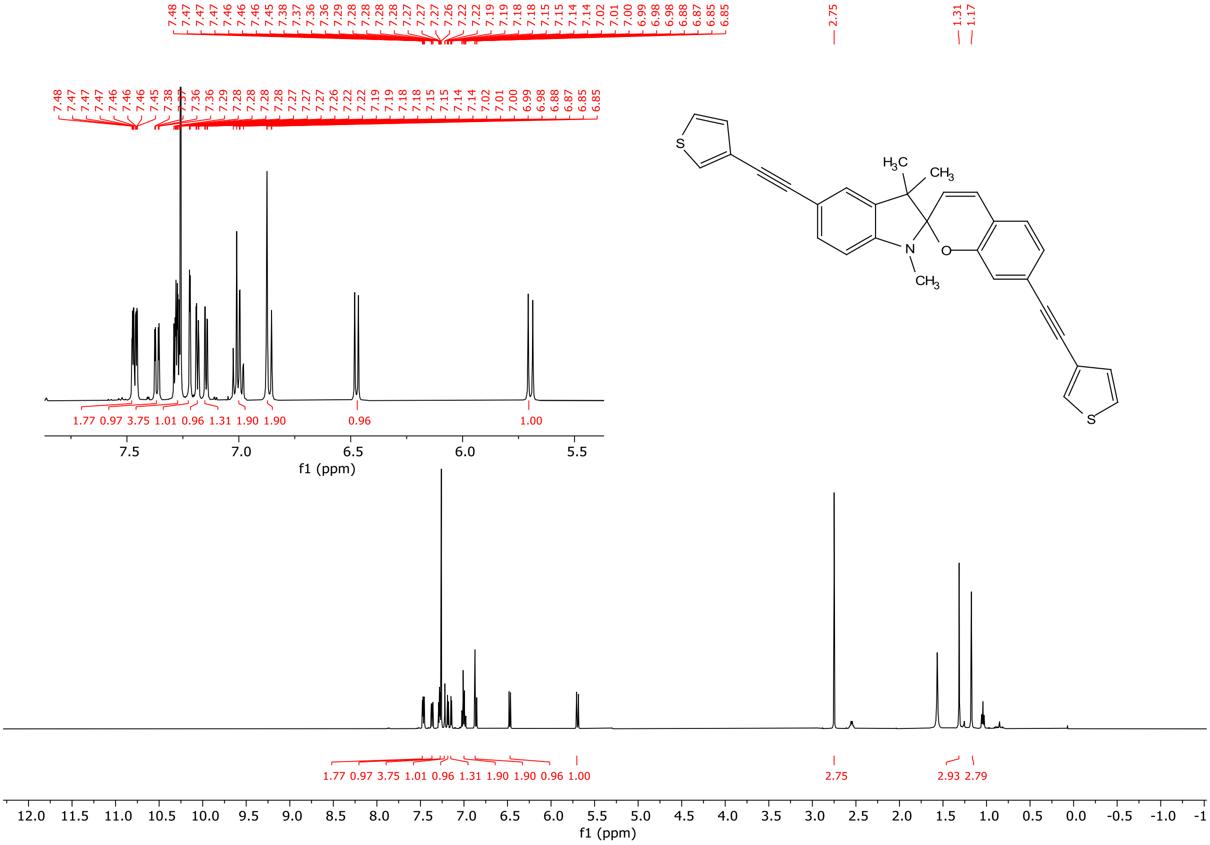

Figure S17 ^{1}H NMR Spectrum of **SP6** in $CDCl_3$.

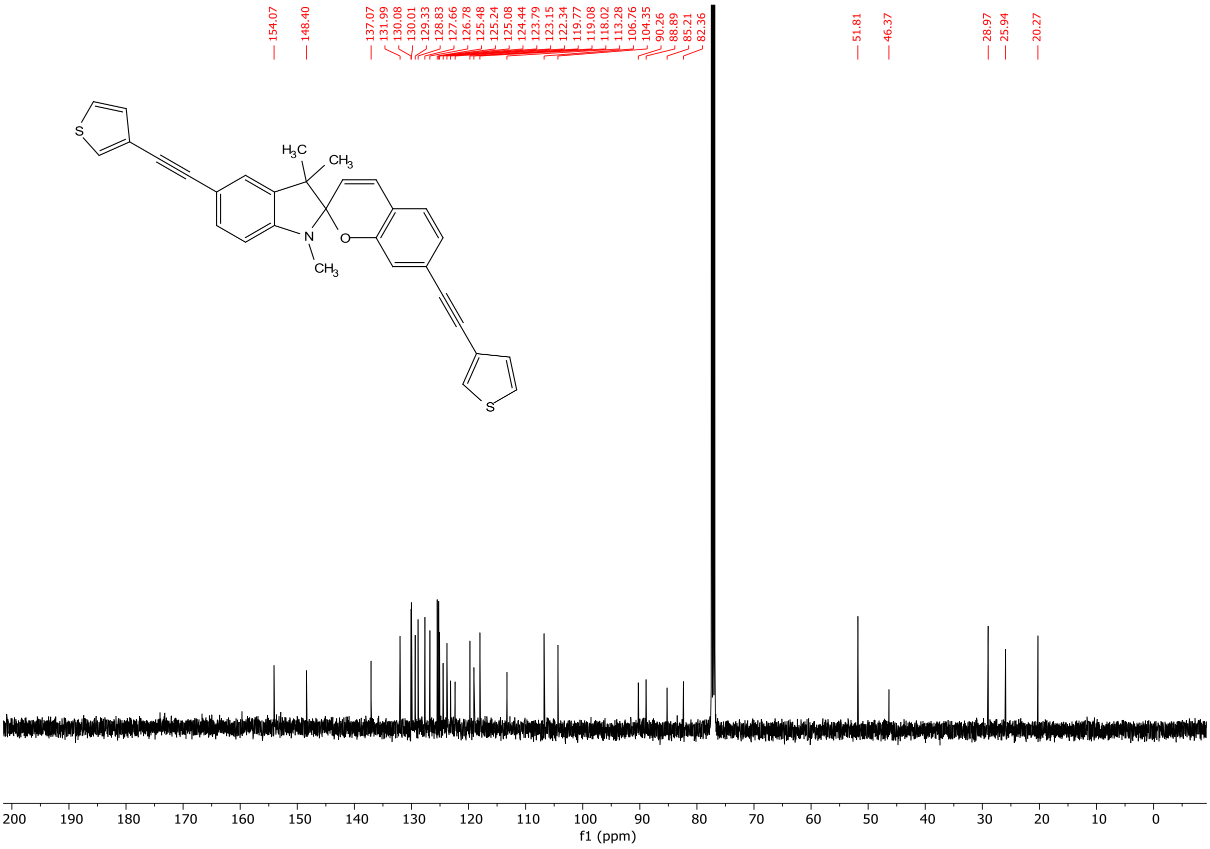

Figure S18 ^{13}C NMR Spectrum of **SP6** in $CDCl_3$.

5-bromo-2,3,3-trimethyl-3*H*-indole (**8**):

Synthesised by slightly modified literature procedure.[18] 4-bromophenylhydrazine hydrochloride (1.00 g, 4.47 mmol) and 3-methyl-butan-2-one (0.58 g, 6.71) were refluxed in acetic acid (15 mL) for 4 h under air. After the solution cooled, most of the solvent was removed by rotary evaporation to leave a red oil. This was taken up in diethyl ether (50 mL) and washed with a sat. $NaHCO_3$ solution (2 x 50 mL), water (50 mL), and brine (50 mL). The organic layer was dried over magnesium sulphate, filtered, and evaporated to dryness to give a red oil. This was subjected to flash column chromatography (silica) using 30% to 50% EtOAc in hexanes. A yellow band gave an orange oil of **8** upon concentration that solidifies in the freezer (990 mg, 93%). The $^{13}C\{^1H\}$ NMR spectral data has been reassigned.

^{1}H NMR ($CDCl_3$, 600 MHz) δ/ ppm: 7.42 (dd, *J* = 2.1, 7.8 Hz, 1H, H6), 7.39 (d, *J* = 2.0 Hz, 1H, H4), 7.38 (d, *J* = 8.0 Hz, 1H, H7), 2.26 (s, 3H, $NCCH_3$), 1.29 (s, 6H, *gem*-CH_3);

$^{13}C\{^1H\}$ NMR ($CDCl_3$, 151 MHz) δ/ ppm: 188.58 (C2), 152.86 (C7a), 147.97 (C3a), 130.79 (C6), 124.98 (C4), 121.41 (C7), 118.98 (C5), 54.27 (C3), 23.08 (*gem*-CH_3), 15.55 ($NCCH_3$);

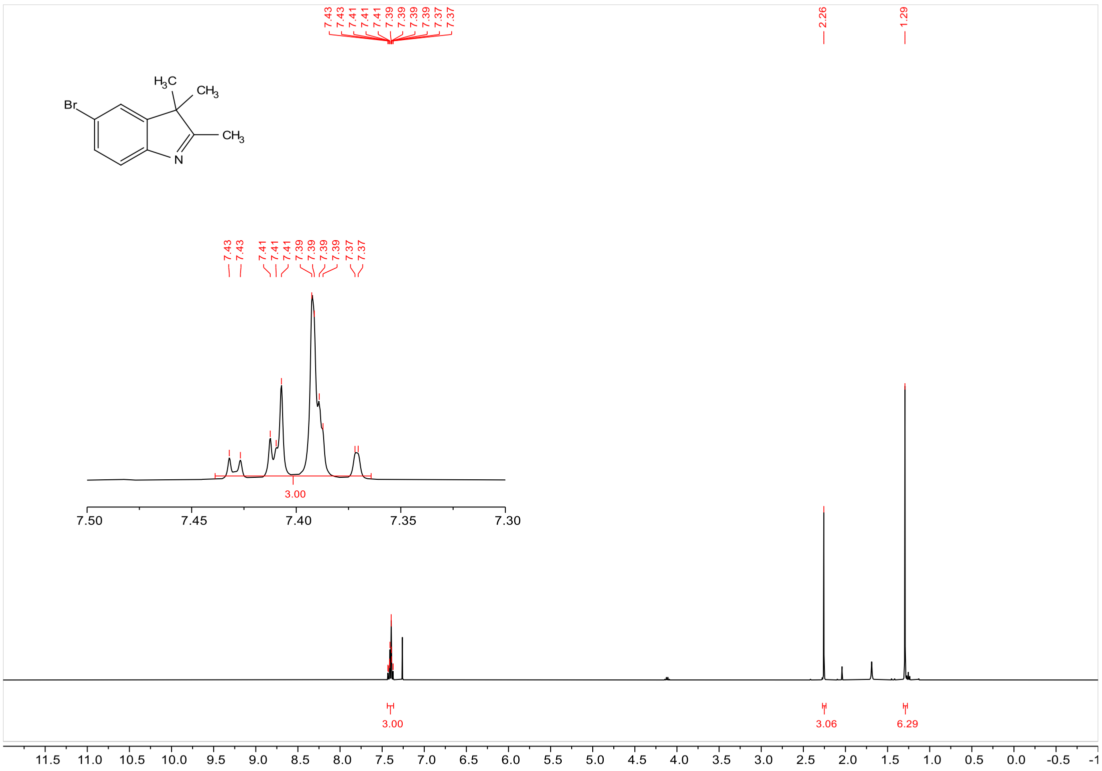

Figure S19 ^{1}H NMR Spectrum of **8** in $CDCl_3$.

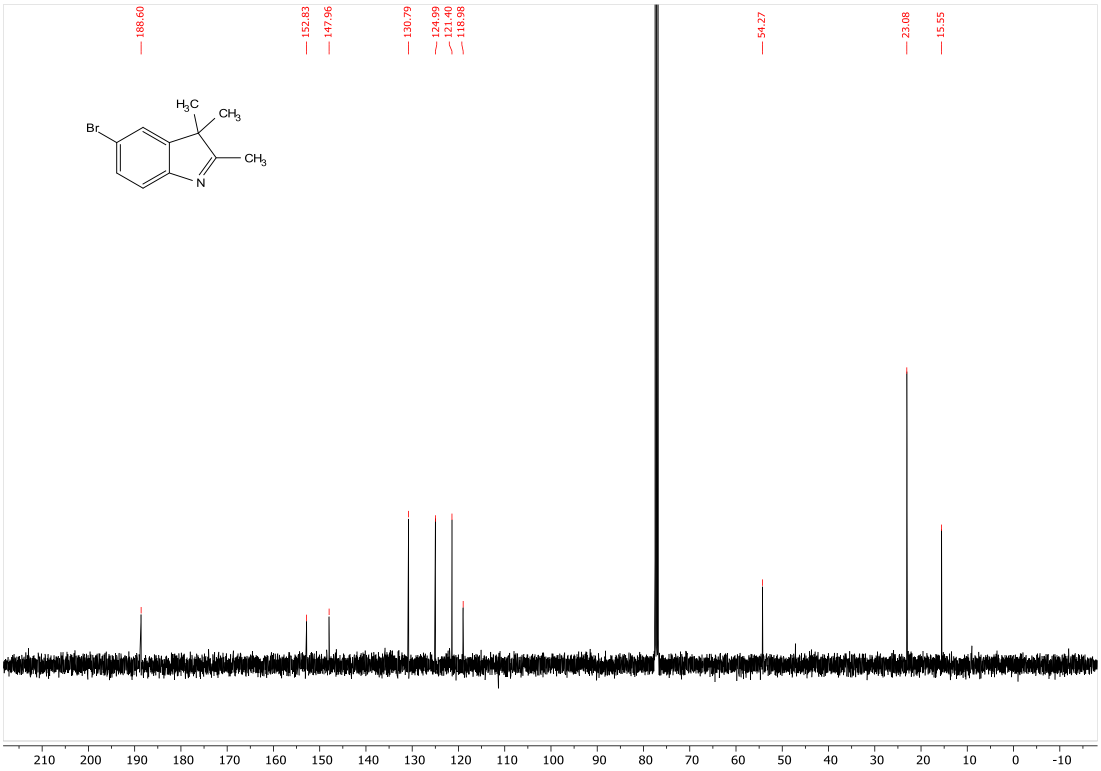

Figure S20 ^{13}C NMR Spectrum of **8** in $CDCl_3$.

5-bromo-1,2,3,3-tetramethyl-3*H*-indol-1-ium iodide (**9**):

Synthesised by slightly modified literature procedure. 5-bromo-2,3,3-trimethyl-3H-indole (**8**) (1.48 g, 6.24 mmol) and iodomethane (0.87 mL, 14 mmol) were refluxed in anhydrous MeCN (15 mL) under nitrogen overnight. Once the solution cooled to ambient temperature, diethyl ether (15 mL) was added, and the microcrystalline solid **9**, which was pink like the flesh of fresh salmon, was collected by vacuum filtration and washed with diethyl ether (1.96 g, 83%). The spectral data is consistent with the literature.[19]

^{1}H NMR (DMSO-d$_6$, 600 MHz) δ/ ppm: 8.17 (d, *J* = 1.3 Hz, 1H, H4), 7.88 (d, *J* = 8.5 Hz, 1H, H7), 7.85 (dd, *J* = 1.3, 8.5 Hz, 1H, H6), 3.95 (s, 3H, NCH_3), 2.76 (s, 3H, $NCCH_3$), 1.54 (s, 6H, *gem*-CH_3);

^{13}C{^{1}H} NMR (DMSO-d$_6$, 151 MHz) δ/ ppm: 196.50 (C2), 143.83 (C7a), 141.41 (C3a), 131.70, 126.66, 122.61, 117.08, 54.16 (C3), 34.89 (NCH_3), 21.44 (*gem*-CH_3), 14.27 ($NCCH_3$);

FTIR (ATR, neat) ν / cm^{-1}: 2966, 1627, 1605 (N=C), 1582, 1467, 1450, 1406;

HRMS (TOF AP(+)) *m/z*: $[M-I]^+$ Calcd for $C_{12}H_{15}BrN^+$ 252.0383; Found 252.0393.

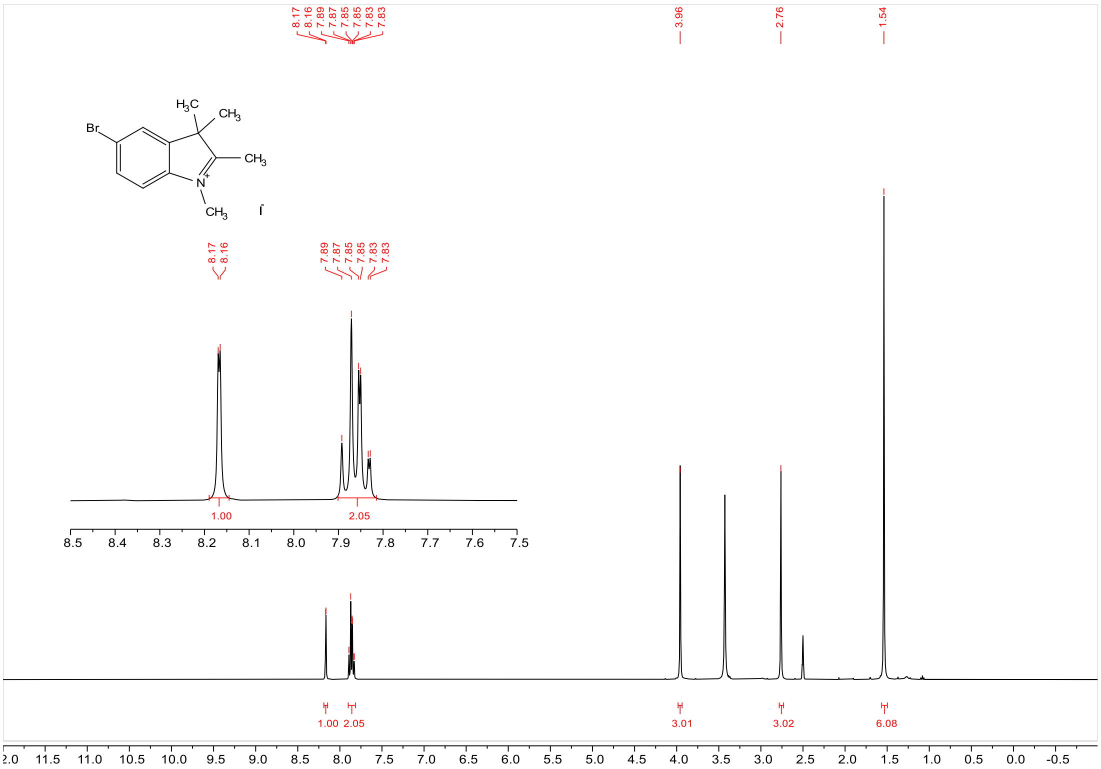

Figure S21 ^{1}H NMR Spectrum of **9** in DMSO-d$_6$.

5',6-dibromo-1',3',3'-trimethylspiro[chromene-2,2'-indoline] (**11**):

Anhydrous EtOH (30 mL) was deoxygenated by sparging with argon for 20 min. Piperidine (0.4 mL, 4.05 mmol), 5-bromo-1,2,3,3-tetramethyl-3*H*-indole iodide **9** (1.00 g, 2.63 mmol) and 5-bromo-2-hydroxybenzaldehyde **10** (580 mg, 2.88 mmol) were then charged and the solution was refluxed for 2 h. The solution was allowed to cool to rt, and the solvent was evaporated on a rotarvap. The residue was dissolved in EtOAc (100 mL) and washed with water (2 x 100 mL) and brine (100 mL). The organic layer was dried over sodium sulphate, filtered, and evaporated to dryness to give a brown amorphous solid. The residue was subject to flash column chromatography (alumina, 20% CH_2Cl_2/Hexanes). The purple/blue band was collected and evaporated to give a white amorphous solid (1.14 g, 99%). The solid becomes slightly pink over time. Compound has been previously reported without comprehensive spectral details.[8, 20]

m.p.: 137-139°C [lit.27 135 -137°C]

1H NMR ($CDCl_3$, 500 MHz) δ/ ppm: 7.28 (dd, *J* = 2.1, 8.3 Hz, 1H, H6’), 7.18 – 7.21 (m, 2H, H5 & H8), 7.16 (d, *J* = 2.1 Hz, 1H, H4’), 6.80 (d, *J* = 10.3 Hz, 1H, H4), 6.60 (d, *J* = 6.9 Hz, 1H, H7), 6.40

(d, *J* = 8.3 Hz, 1H, H7'), 5.71 (d, *J* = 10.3 Hz, 1H, H3), 2.70 (s, 3H, -NCH_3), 1.28 (s, 3H, *gem*-CH_3), 1.17 (s, 3H, *gem*-CH_3);

^{13}C{^{1}H} NMR ($CDCl_3$, 126 MHz) δ/ ppm: 153.38 (C8a), 147.24 (C7a'), 139.04 (C3a'), 132.50 (C8), 130.37 (C6'), 129.27 (C5), 128.79 (C4), 124.92 (C4'), 120.56 (C4a), 120.16 (C3), 116.88 (C8), 112.13 (C6), 111.14 (C5'), 108.49 (C7'), 104.60 (C2), 52.04 (C3'), 29.04 (-NCH_3), 25.78 (*gem*-CH_3), 20.04 (*gem*-CH_3);

FTIR (ATR, neat) ν / cm^{-1}: 2962, 2870, 1645 (C=C), 1599, 1473;

HRMS (TOF AP(+)) *m/z*: $[M+H]^+$ Calcd for $C_{19}H_{18}Br_2NO^+$ 435.9730, Found 435.9744.

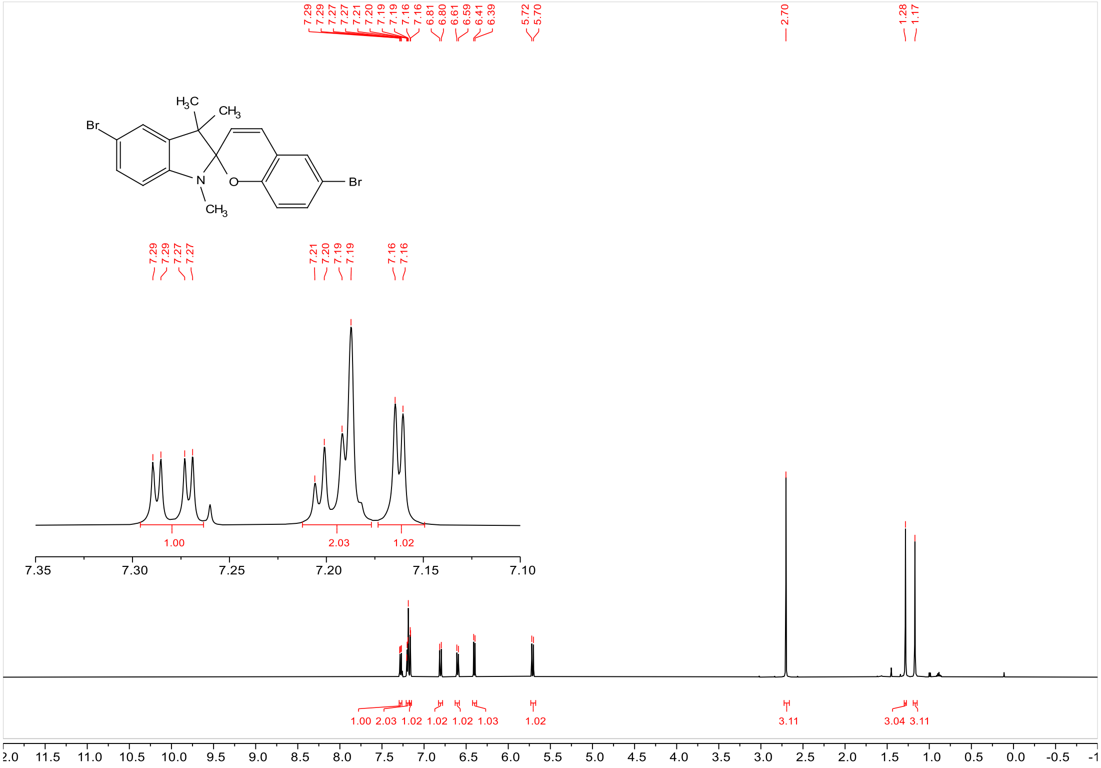

Figure S22 ^{1}H NMR Spectrum of **11** in $CDCl_3$.

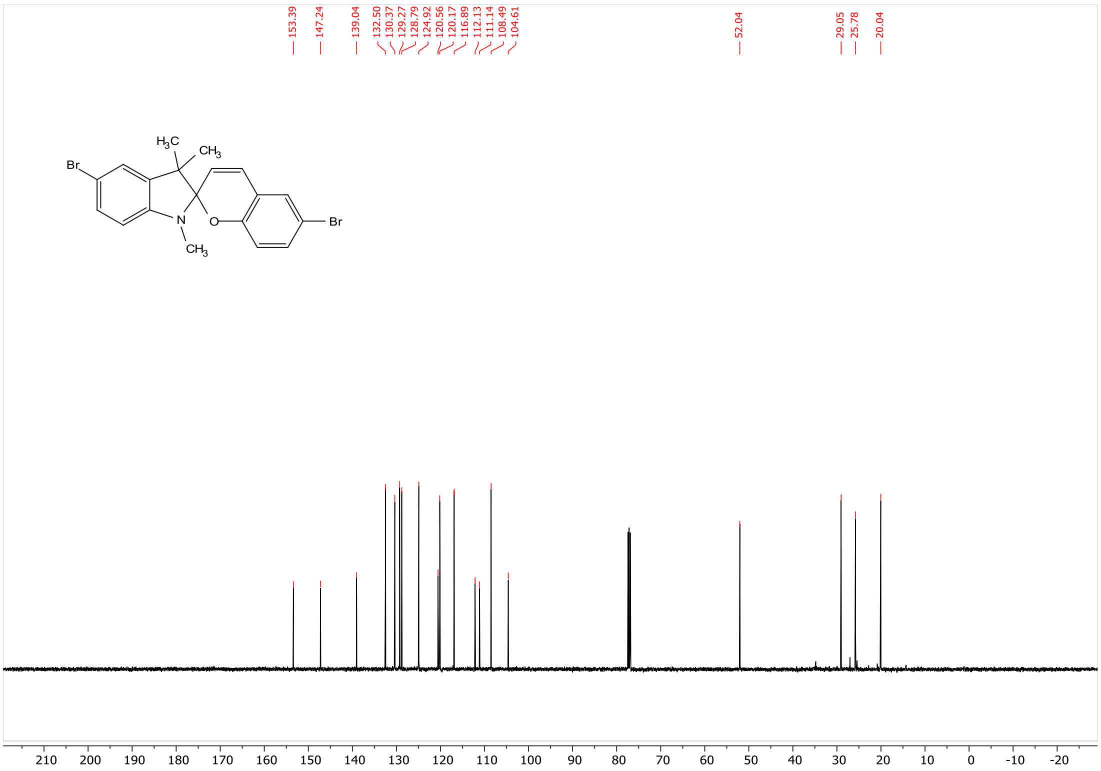

Figure S23 ^{13}C NMR Spectrum of **11** in $CDCl_3$.

5',6-dipyridiyl-1',3',3'-trimethylspiro[chromene-2,2'-indoline] (**SP7**):

5',6-dibromo-1',3',3'-trimethylspiro[chromene-2,2'-indoline] **11** (50 mg, 0.11 mmol), 4-pyridinylboronic acid (59 mg, 0.48 mmol) and K_2CO_3 (100 mg, 0.724 mmol) were suspended in dimethoxyethane (5 mL) and distilled water (1 mL). The solution was subject to three cycles of FPT to remove dissolved oxygen and backfilled with nitrogen. Pd(dppf)Cl_2.CH_2Cl_2 (5mg, 0.006 mmol) was added, and the solution was refluxed for 20 h. The solution was allowed to cool to rt, and the solvent was removed under reduced pressure. The residue was extracted (with trituration) with EtOAc (3 x 5 mL). The organic layer was washed with water (3 x 15 mL) and brine (15 mL). The organic layer was dried over Na_2SO_4, filtered, and evaporated to dryness on a rotary evaporator. The residue was subject to flash column chromatography (alumina, CH_2Cl_2 to 1% CH_3OH). The blue band was collected as a pale-yellow fraction. Removal of the eluent gives a yellow powder of **SP7** (26 mg, 55%). The NMR agrees with the literature.[7] Crystals for X-ray diffraction studies were grown by slow cooling a methanolic solution.

m.p.: 176 – 177°C

^{1}**H NMR** (CD_2Cl_2, 400 MHz) δ/ ppm: 8.58 (m, 4H, py-2 and py-2'), 7.55 (dd, *J* = 1.9, 8.1 Hz, 1H, H6'), 7.51 (m, 2H, py-3 or py-3'), 7.41 – 7.48 (m, 5H, py-3 or py-3', H7 and H5 and H4'), 7.01 (d, *J* = 10.2 Hz, 1H, H4), 6.82 (d, *J* = 8.4 Hz, 1H, H8), 6.64 (d, *J* = 8.1 Hz, 1H, H7'), 5.81 (d, *J* = 10.2 Hz, 1H, H3), 2.82 (s, 3H, NCH_3), 1.38 (s, 3H, *gem*-CH_3), 1.24 (s, 3H, *gem*-CH_3);

^{13}**C{^{1}H} NMR** (CD_2Cl_2, 101 MHz) δ/ ppm: 155.64 (C8a), 150.60 (py-2 or py-2'), 150.48 (py-2 or py-2'), 149.66 (C7a'), 148.77 (py-4'), 147.74 (py-4), 138.21 (C3a'), 130.51 (C6), 129.89 (C4), 129.32 (C5'), 128.76 (C7), 127.25 (C6'), 125.74 (C5), 121.18 (py-3 or py-3'), 121.06 (py-3 or py-3'), 120.66 (C4'), 120.09 (C3), 119.73 (C4a), 115.92 (C8), 107.49 (C7'), 105.48 (C2), 52.29 (C3'), 29.10 (NCH_3), 26.06 (*gem*-CH_3), 20.12 (*gem*-CH_3);

FTIR (ATR, neat) ν / cm^{-1}: 3026, 2962, 2203, 1645 (C=C), 1612, 1591, 1544, 1507, 1477, 956 (C_{spiro}-O);

HRMS (TOF AP(+)) *m/z*: $[M+H]^+$ Calcd for $C_{29}H_{26}N_3O^+$ 432.2071, Found 432.2075.

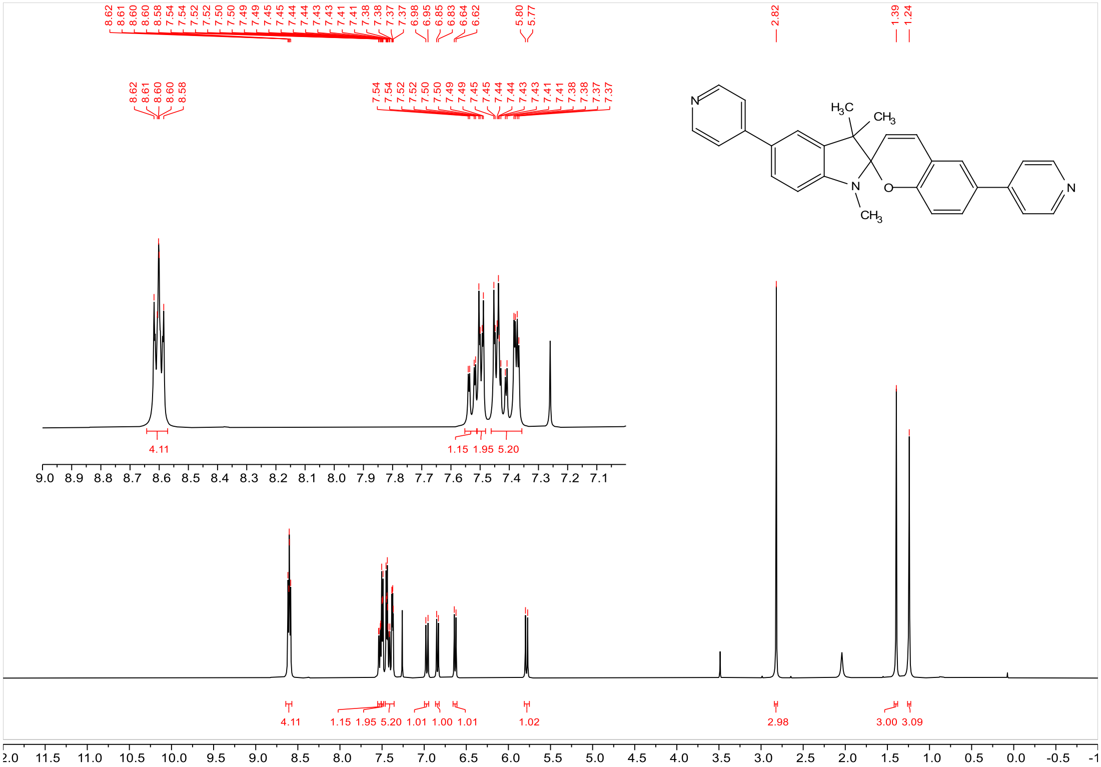

Figure S24 ^{1}H NMR Spectrum of **SP7** in $CDCl_3$.

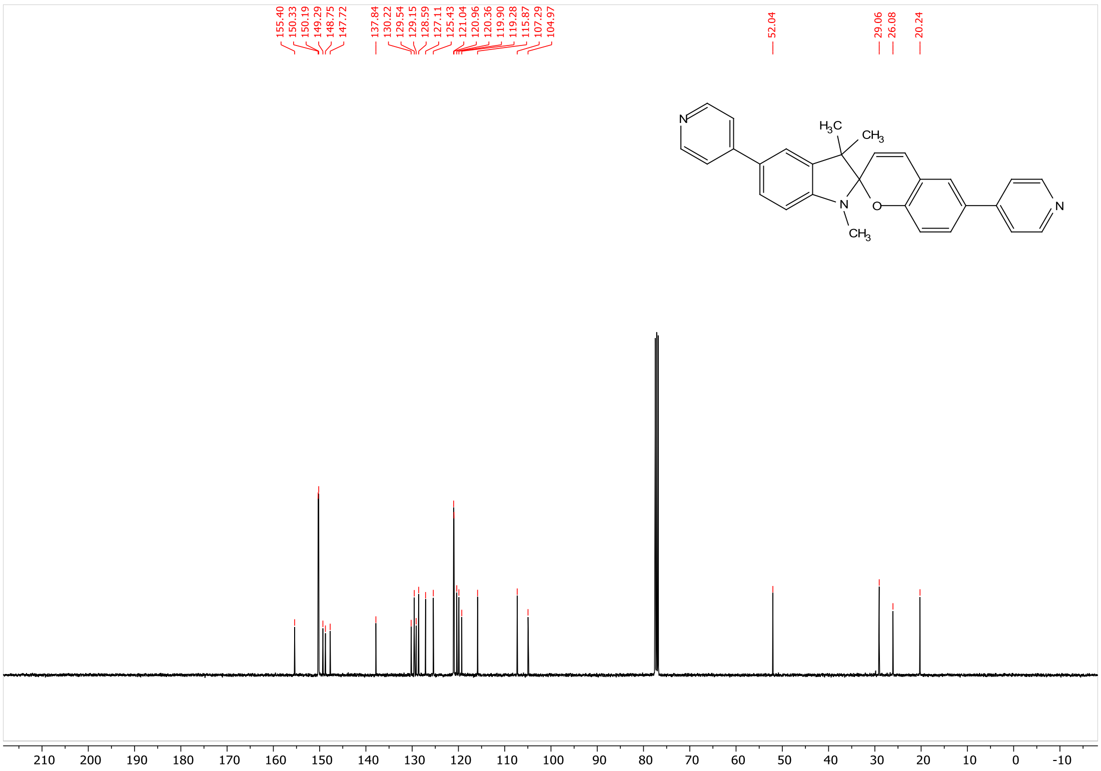

Figure S25 ^{13}C NMR Spectrum of **SP7** in $CDCl_3$.

1-((4’-bromophenyl)thio)-2-methylpropan-2-ol (**12**):

Synthesised by a literature procedure.[9] Into AR Acetone (120 mL) was added 4-bromobenzenethiol (3.00 g, 15.9 mmol) and 2,2-dimethyloxirane (2.28 g, 31.6 mmol). The solution was deoxygenated by sparging with argon for 30 minutes. NEt_3 (9 mL) was degassed by 3 x FPT cycles and then added by syringe. The clear solution was stirred overnight at room temperature under an argon blanket. The solution was then evaporated *in vacuo* to yield a clear oil that solidifies as a white crystalline solid (4.10 g, 99%). IR has also been obtained.

^{1}H NMR ($CDCl_3$, 400 MHz) δ/ ppm: 7.39 (d, J = 8.5 Hz, 1H, H3’), 7.27 (d, J = 8.5 Hz, 2H, H2’), 3.08 (s, 2H, H1), 2.06 (s (br.), 1H, -OH), 1.30 (s, 6H, H3);

^{13}C{^{1}H} NMR ($CDCl_3$, 101 MHz) δ/ ppm: 136.50 (C1’), 132.13 (C3’), 131.08 (C2’), 120.15 (C4’), 70.89 (C2), 48.69 (C1), 28.86 (C3);

FTIR (ATR, neat) ν / cm^{-1}: 3244 (OH), 2970, 1471, 1377, 1362

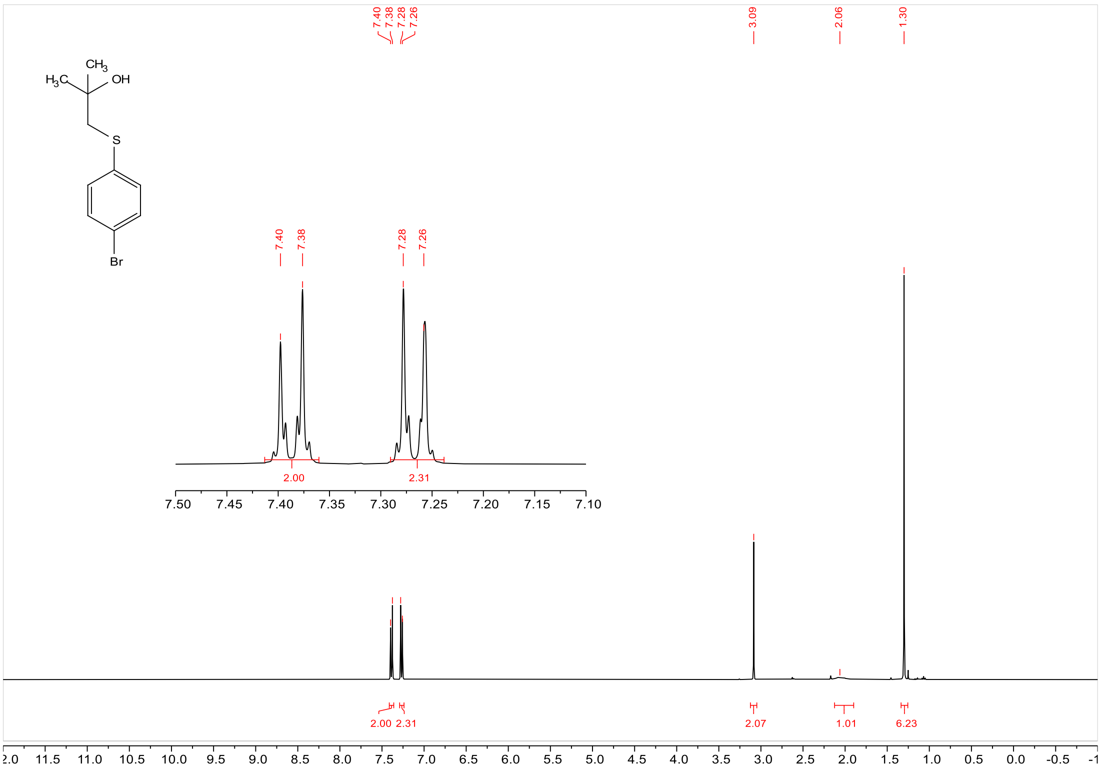

Figure S26 ^{1}H NMR Spectrum of **12** in $CDCl_3$.

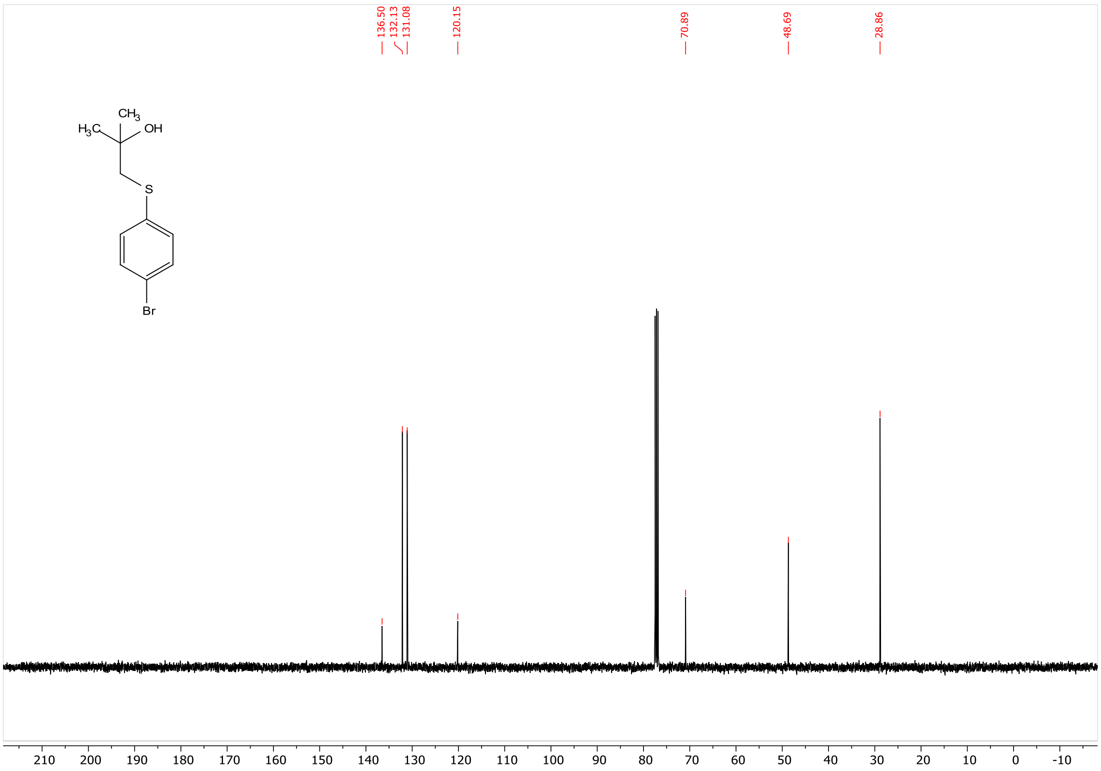

Figure S27 ^{13}C NMR Spectrum of **12** in $CDCl_3$.

5-bromo-3,3-dimethyl-2,3-dihydrobenzo[*b*]thiophene (**13**):

Synthesised by a modified literature procedure.[4b] Compound **12** (1.05 g, 3.83 mmol) was dissolved in CH_2Cl_2 (30 mL) (sparged with nitrogen for 20 min) and added dropwise overnight to $AlCl_3$ (3.50 g, 26.2 mmol) in CH_2Cl_2 (70 mL) (sparged with nitrogen for 20 min). A water bath was used to control temperature overnight. The solution becomes deep red to dark brown. After 3 days the solution was cooled using an ice bath and 5% HCl (100 mL) was carefully added to quench the reaction. The now pale-yellow organic phase was separated and washed with water (100 mL) and brine (100 mL). The organic layer was dried over Na_2SO_4, filtered and the solvent was removed to give a yellow oil. The compound was purified using flash silica column chromatography (hexanes) to give a clear oil **13** after removal of the eluent (516 mg, 55%). The NMR agrees with the literature.[9]

^{1}H NMR ($CDCl_3$, 400 MHz) δ/ ppm: 7.22 (dd, *J* = 2, 8.4 Hz, 1H, H6), 7.14 (d, *J* = 2 Hz, 1H, H4), 7.03 (d, *J* = 8.4 Hz, 1H, H7), 3.18 (s, 2H, H2), 1.36 (s, 6H, *gem*-CH_3);

^{13}C{^{1}H} NMR ($CDCl_3$, 101 MHz) δ/ ppm: 150.41 (C3a), 139.81 (C7a), 130.34 (C6), 126.12 (C4), 123.85 (C7), 117.86 (C5), 47.55 (C3), 47.47 (C2), 27.31 (*gem*-CH_3);

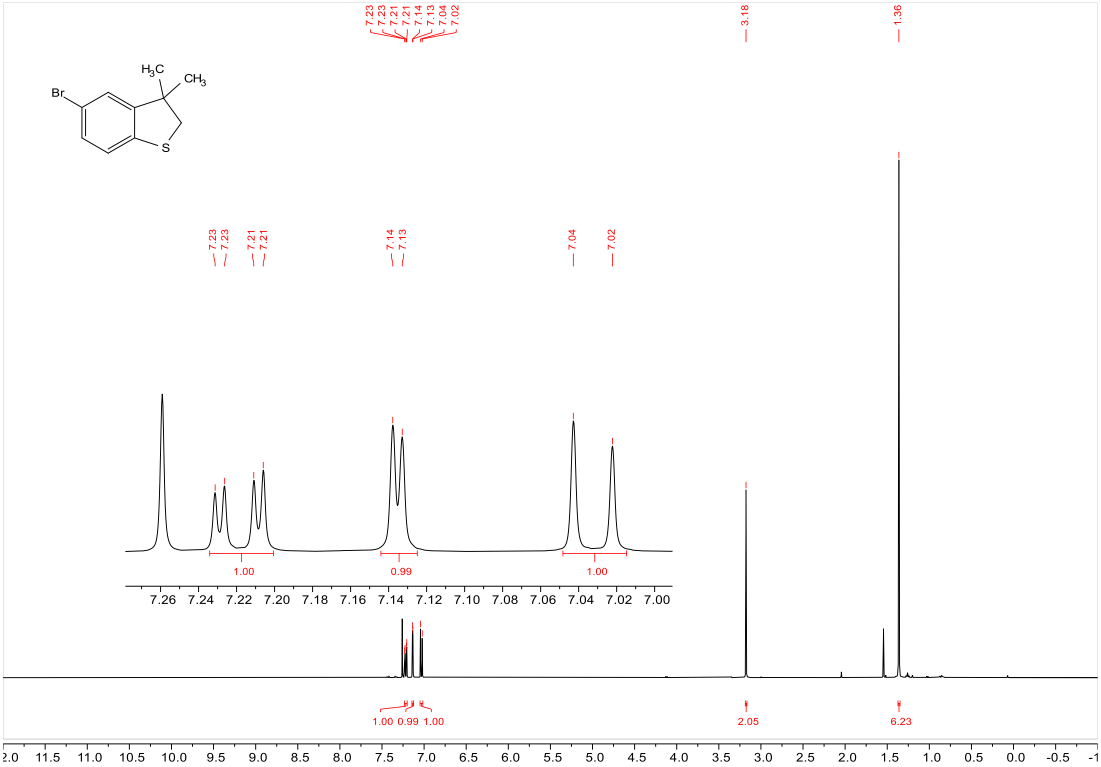

Figure S28 ^{1}H NMR Spectrum of **13** in $CDCl_3$.

(3,3-dimethyl-2,3-dihydrobenzo[*b*]thiophen-5-yl)boronic acid (**15**):

13 (516 mg, 2.12 mmol) was dissolved in dry THF (20 mL) and cooled to -78°C using a dry-ice and acetone bath. 1.6 M (2.0 mL ,3.2 mmol) *n*-BuLi was added dropwise over 30 min and then continued to stir for another 30 min. B(*i*-OPr)$_3$ (0.70 mL, 3.2 mmol) was then added dropwise over 15 min and the solution was stirred for a further 15 min. The cooling bath was removed, and the solution was allowed to warm to room temperature and stirred overnight. The reaction was quenched with water (20 mL), and then acidified using 5% HCl solution (to ~ pH 1). The THF was removed under reduced pressure and the white precipitate **15** was collected, washed with cold water and hexanes, air dried and then stored in a vacuum desiccator overnight (344 mg, 78%).

m.p.: 182 – 185°C

1H NMR (DMSO-d$_6$, 400 MHz) δ/ ppm: 7.91 (s, 2H, B(OH)$_2$), 7.56 (d, *J* = 1.2 Hz, 1H, H4), 7.55 (dd, *J* = 1.2, 8.2 Hz, 1H, H6), 7.15 (d, *J* = 8.2 Hz, 1H, H7), 3.18 (s, 2H, H2), 1.31 (s, 6H, *gem*-CH$_3$);

^{13}C{^{1}H} NMR (DMSO-d$_6$, 101 MHz) δ/ ppm: 146.83 (C3a), 142.32 (C7a), 133.45 (C6), 130.22 (C5; ^{1}H – ^{13}C{^{1}H} HMBC), 128.38 (C4), 121.15 (C7), 46.56 (C3), 46.22 (C2), 27.15 (*gem*-CH$_3$),

FTIR (ATR, neat) ν / cm^{-1}: 3248 (OH), 2959, 2923, 1593, 1463, 1405, 1335 (BO), 1278;

HRMS (TOF AP(+)) *m/z*: [M]$^+$ Calcd for $C_{10}H_{13}BO_2S$+ 208.0729, Found 208.0723.

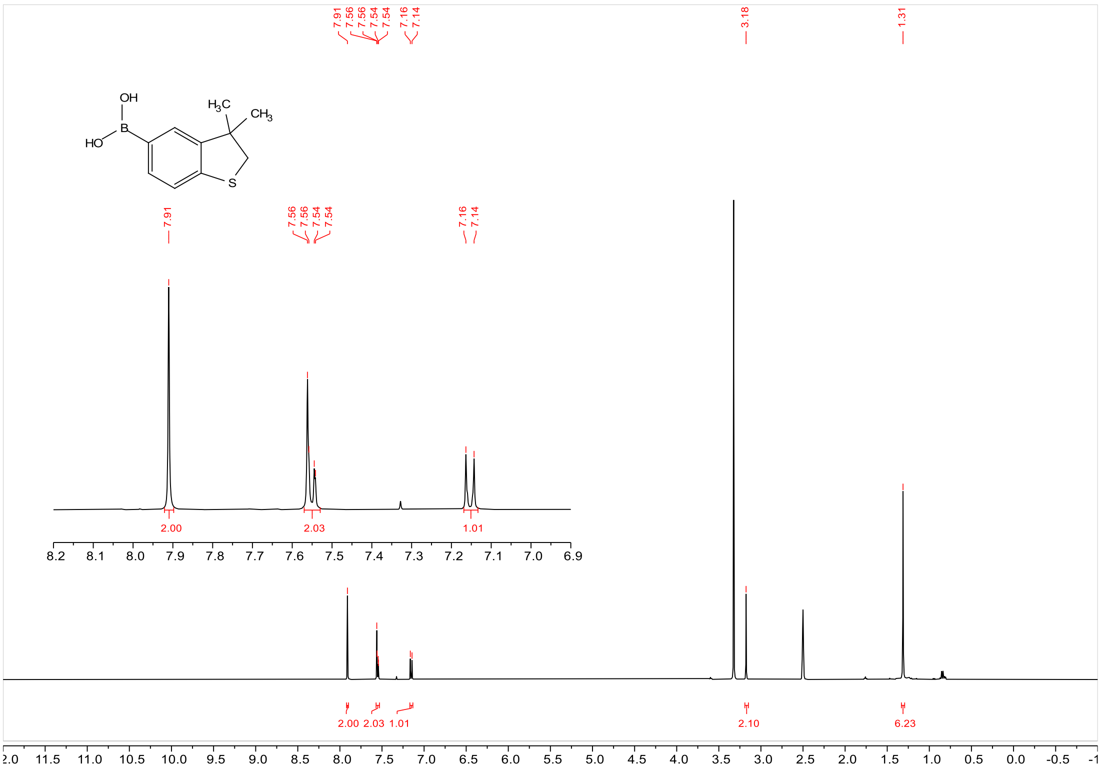

Figure S29 ^{1}H NMR Spectrum of **15** in DMSO-d_6.

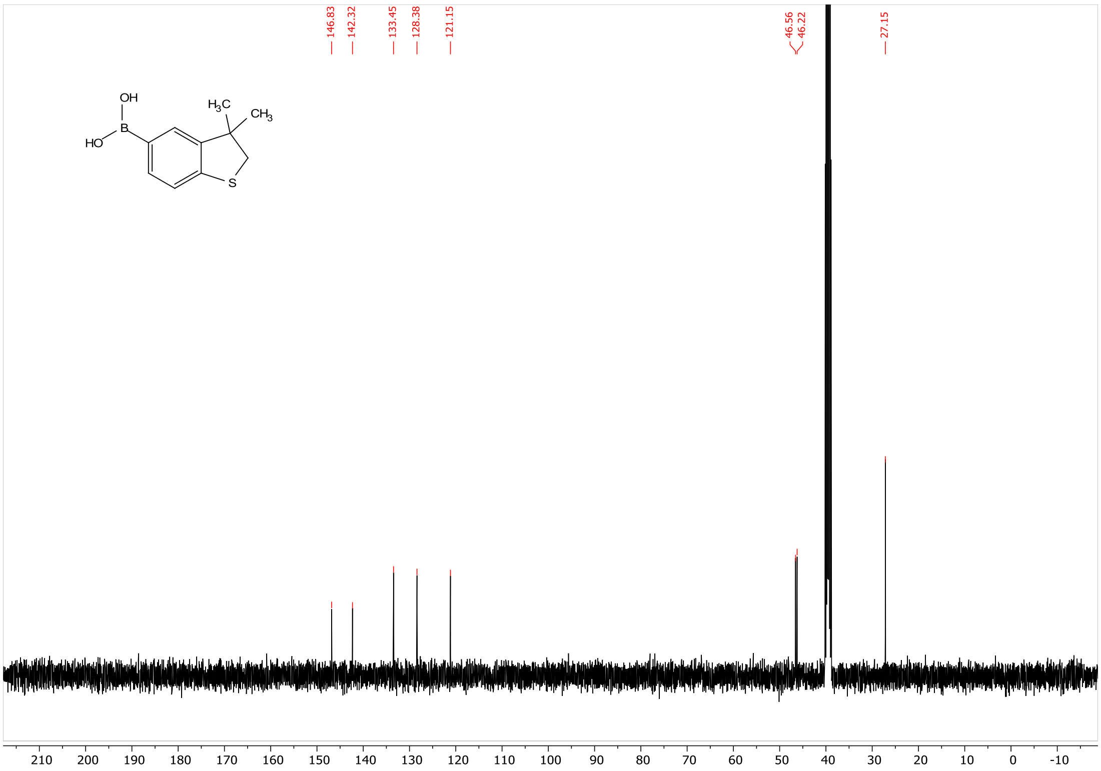

Figure S30 ^{13}C NMR Spectrum of **15** in DMSO-d_6.

(4-(methylthio)phenyl)boronic acid (**16**):

Synthesised by a modified literature procedure.[11] Bromothioanisole **14** (2.13 g, 10.5 mmol) was dissolved in THF (75 mL) and cooled to -78°C. 1.6 M *n*-BuLi (14 mL, 22.4 mmol) in hexanes was added dropwise over 30 min and the reaction was stirred for 1 h. B(*i*-OPr)$_3$ (4.0 mL, 17 mmol) was added dropwise over 30 min and stirred for 15 min. The dry-ice/acetone bath was removed and the reaction was stirred overnight. Water (75 mL) was added slowly and carefully to quench the reaction. The solution was acidified to pH 1 using a 2M solution of HCl. The THF was removed under reduced pressure to give a white precipitate that was collected on a Büchner funnel, washed with water and hexanes. The white powder **16** (1.42 g, 81%) was stored in a vacuum desiccator. IR and HRMS has also been acquired.

^{1}H NMR (DMSO-d$_6$, 600 MHz) δ/ ppm: 7.98 (s, 2H, -OH), 7.72 (d, *J* = 8.3 Hz, 2H), 7.21 (d, *J* = 8.3 Hz, 2H), 2.48 (s, 3H, -CH3);

FTIR (ATR, neat) ν / cm^{-1}: 3331 (OH), 1590, 1546, 1497, 1389, 1327 (-BO);

HRMS (TOF AP(+)) *m/z*: [M]$^+$ Calcd for $C_7H_9BO_2S$ 168.0416, Found 168.0141.

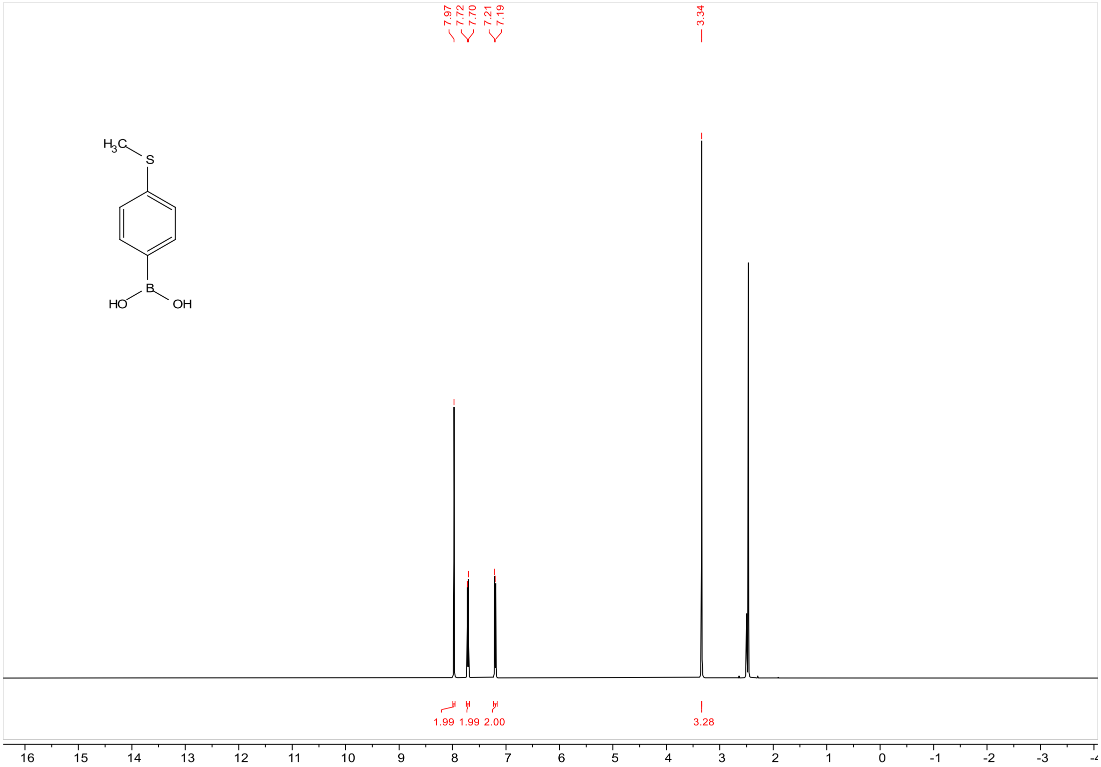

Figure S31 ^{1}H NMR Spectrum of **16** in DMSO-*d*$_6$.

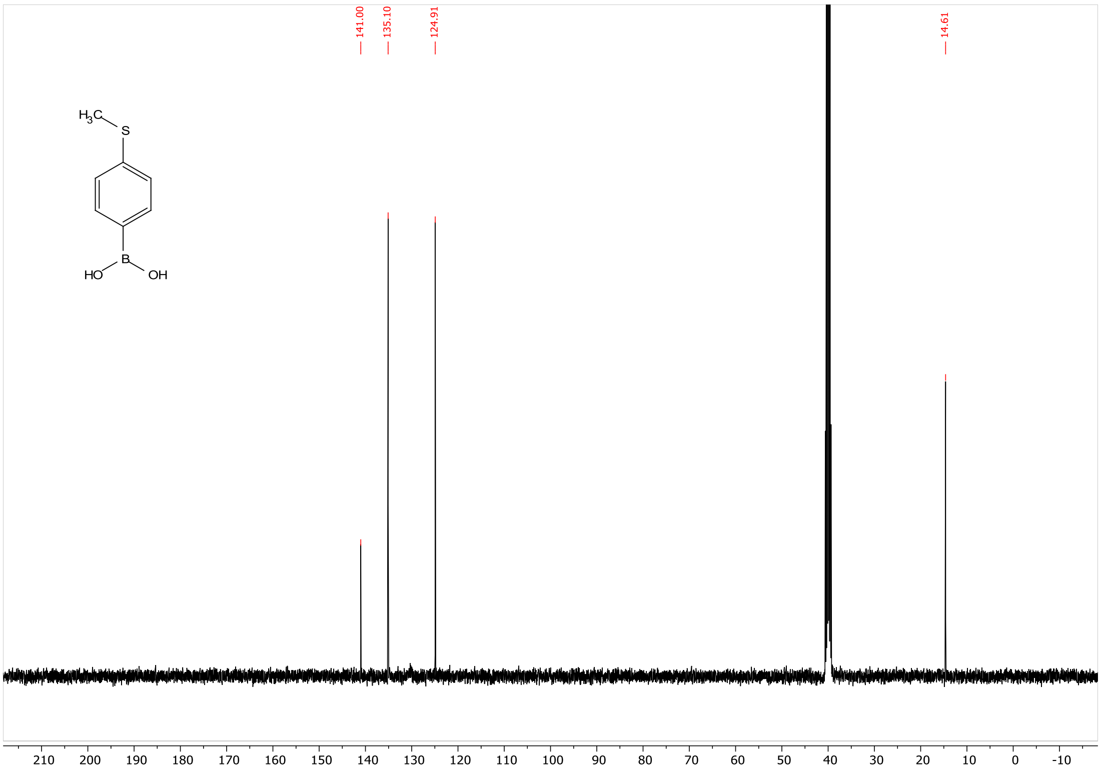

Figure S32 ^{13}C NMR Spectrum of **16** in DMSO-d_6.

2-hydroxy-4-iodobenzaldehyde (**17**):

Synthesised by literature procedure.[21] ^{1}H and ^{13}C are consistent with the literature. IR is also consistent with previous literature characterisation.[22] All spectral data are collected here for future reference.

1H NMR ($CDCl_3$, 500 MHz) δ/ ppm: 11.02 (s, 1H, -OH), 9.85 (s, 1H, -CHO), 7.44 (d, *J* = 1.3 Hz, 1H, H3), 7.40 (d, *J* = 1.3, 8.1 Hz, 1H H5), 7.23 (d, *J* = 8.1 Hz, 1H, H6).

^{13}C{^{1}H} ($CDCl_3$, 126 MHz) δ/ ppm: 196.24, 161.44, 134.34, 129.55, 127.40, 120.09, 105.30.

FTIR (ATR, neat) ν / cm^{-1}: 3172 (-OH), 2879, 2241, 1661 (C=O), 1598, 1547.

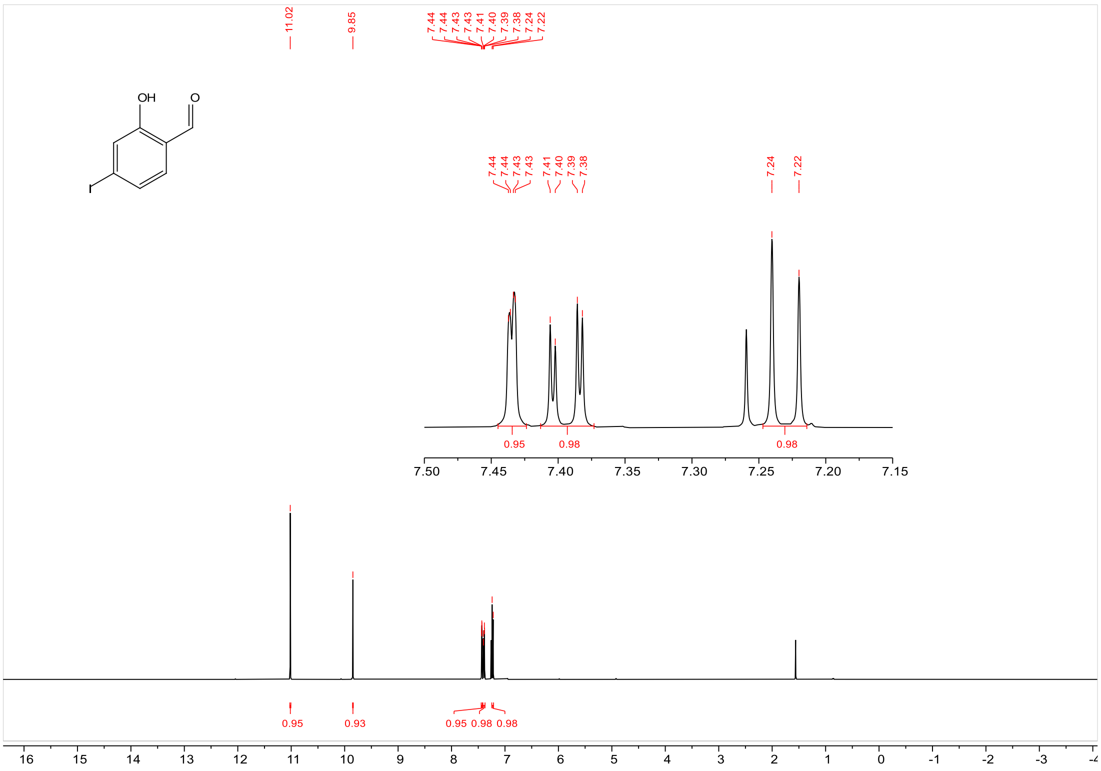

Figure S33 ^{1}H NMR Spectrum of **17** in $CDCl_3$.

5-(3,3-dimethyl-2,3-dihydrobenzo[*b*]thiophen-5-yl)-2-hydroxybenzaldehyde (**18**): 5-bromosalicylaldehyde **10** (100 mg, 0.497 mmol), (3,3-dimethyl-2,3-dihydrobenzo[*b*]thiophen-5-yl)boronic acid **15** (124 mg, 0.596 mmol) and Na_2CO_3 (166 mg, 1.57 mmol) were suspended in dimethoxyethane (4 mL) and water (1 mL). The solution was subject to FPT x 3 and backfilled with nitrogen. Pd(dppf)Cl_2.CH_2Cl_2 (20 mg, 0.024 mmol) was added, and the solution was refluxed for 16 h under nitrogen. The reaction was cooled, and the solvent removed under reduced pressure. The residue was extracted with EtOAc/2M HCl mixture (3 x 10 mL) (1:1). The organic layer was washed with water (3 x 15 mL). The aqueous layer was extracted with EtOAc (15 mL). The combined organic layers were washed with brine (30 mL), dried over sodium sulphate, filtered, and evaporated to dryness. The orange solid was then subject to flash column chromatography (silica, CH_2Cl_2:Hexanes (1:1)) to give a yellow microcrystalline solid **18** (111 mg, 78%). The compound can be further recrystallized from hot ethanol if required.

m.p.: 137-139°C

^{1}**H NMR** ($CDCl_3$, 600 MHz) δ/ ppm: 10.98 (s, 1H, OH), 9.98 (s, 1H, CHO), 7.74 (dd, *J* = 2.3, 8.5 Hz, 1H, H4), 7.71 (d, *J* = 2.3 Hz, 1H, H6), 7.31 (dd, *J* = 1.8, 7.9 Hz, 1H, H6’), 7.26 (d, *J* = 7.9 Hz,

1H, H7'), 7.20 (d, J = 1.8 Hz, 1H, H4'), 7.06 (d, J = 8.5 Hz,1H, H3), 3.24 (s, 2H, H2'), 1.44 (s, 6H, *gem*-CH_3).

$^{13}C\{^1H\}$ NMR ($CDCl_3$, 151 MHz) δ/ ppm: 196.83 (CHO), 160.91 (C2), 149.07 (C3a'), 140.24 (C7a'), 136.18 (C5'), 135.73 (C4), 133.51 (C5), 131.66 (C6), 126.06 (C6'), 122.97 (C7'), 121.09 (C4'), 120.86 (C6), 118.26 (C3), 47.59 (C3'), 47.48 (C2'), 27.57 (*gem*-CH_3).

FTIR (ATR, neat) ν / cm^{-1}: 3191 (OH), 2959, 2925, 2864, 1694, 1662 (C=O), 1617, 1584, 1493, 1459.

HRMS (TOF AP(+)) *m/z*: $[M]^+$ Calcd for $C_{17}H_{16}O_2S^+$ 284.0871, Found 284.0875.

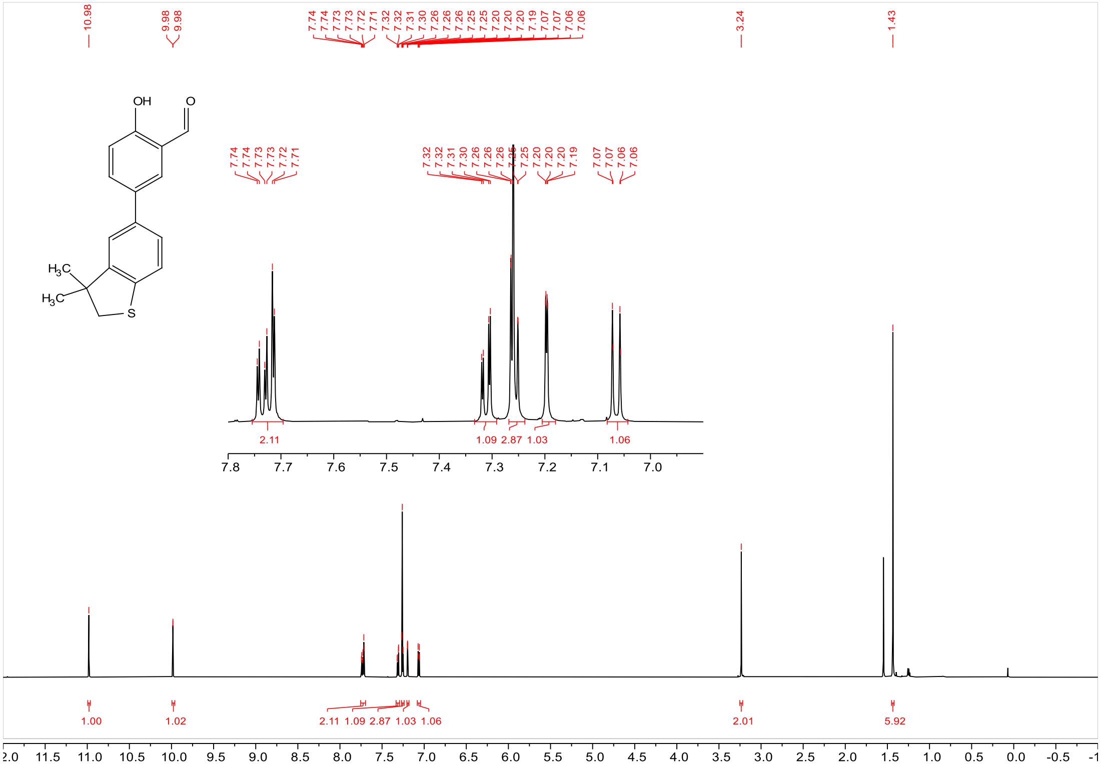

Figure S34 ^1H NMR Spectrum of **18** in $CDCl_3$.

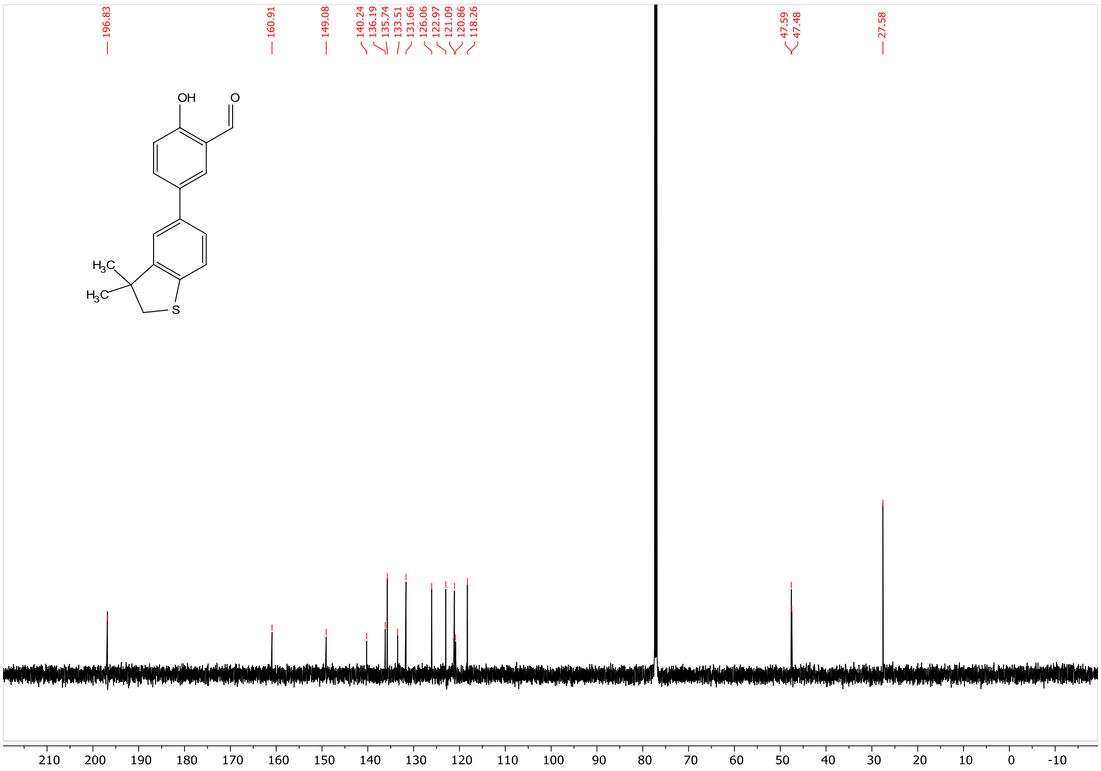

Figure S35 ^{13}C NMR Spectrum of **18** in $CDCl_3$.

4-hydroxy-4'-(methylthio)-[1,1'-biphenyl]-3-carbaldehyde (**19**):

Synthesised by a modified literature procedure.[12] To 5-bromo-2-hydroxybenzaldehyde **10** (100 mg, 0.497 mmol), (4-(methylthio)phenyl)boronic acid **16** (125 mg, 0.744 mmol) and sodium carbonate (166 mg, 1.57 mmol) was added dimethoxyethane (4 mL) and water (1 mL). After 3 cycles of freeze, pump, thaw, Pd(dppf)Cl_2.CH_2Cl_2 (20 mg, 0.024 mmol) was added, and the solution refluxed overnight (16 h) under nitrogen. The solution cooled and then the solvent evaporated in vacuo. The residue was triturated with diethyl ether (20 mL) and washed with 1M HCl (3 x 20 mL) solution. The aqueous layer was further extracted with diethyl ether (2 x 20 mL). The organic layer was washed with water (20 mL) and a saturated brine solution (20 mL), dried over sodium sulphate, filtered, and evaporated. The yellow solid was subject to flash silica chromatography using CH_2Cl_2/Hexanes (1:1) as the eluent. A pale-yellow powder was obtained after evaporation of solvent (92 mg, 76%). ^{13}C NMR is assigned, and IR obtained. Compound can be recrystallised from hot ethanol if needed.

m.p.: 97-100°C

^{1}H NMR ($CDCl_3$, 400 MHz) δ/ ppm: 10.99 (s, 1H, OH), 9.97 (s, 1H, CHO), 7.73 – 7.76 (m, 2H, H2 & H6), 7.48 (d, *J* = 8.3 Hz, H2'), 7.34 (d, *J* = 8.3 Hz, H3'), 7.07 (d, *J* = 8.6 Hz, H5), 2.53 (s, 3H, SCH_3);

^{13}C{^{1}H} NMR ($CDCl_3$, 101 MHz) δ/ ppm: 196.78 (CHO), 161.06 (C4), 138.03 (C4'), 136.25 (C1'), 135.58 (C6), 132.83 (C1), 131.66 (C2), 127.21 (C3'), 127.06 (C2'), 120.88 (C3), 118.34 (C5), 16.00 (SCH_3);

FTIR (ATR, neat) ν / cm^{-1}: 3023 (OH), 1682,1647 (C=O), 1591, 1503, 1475, 1433, 1397, 1363, 1330, 1293, 1266 1229 1177.

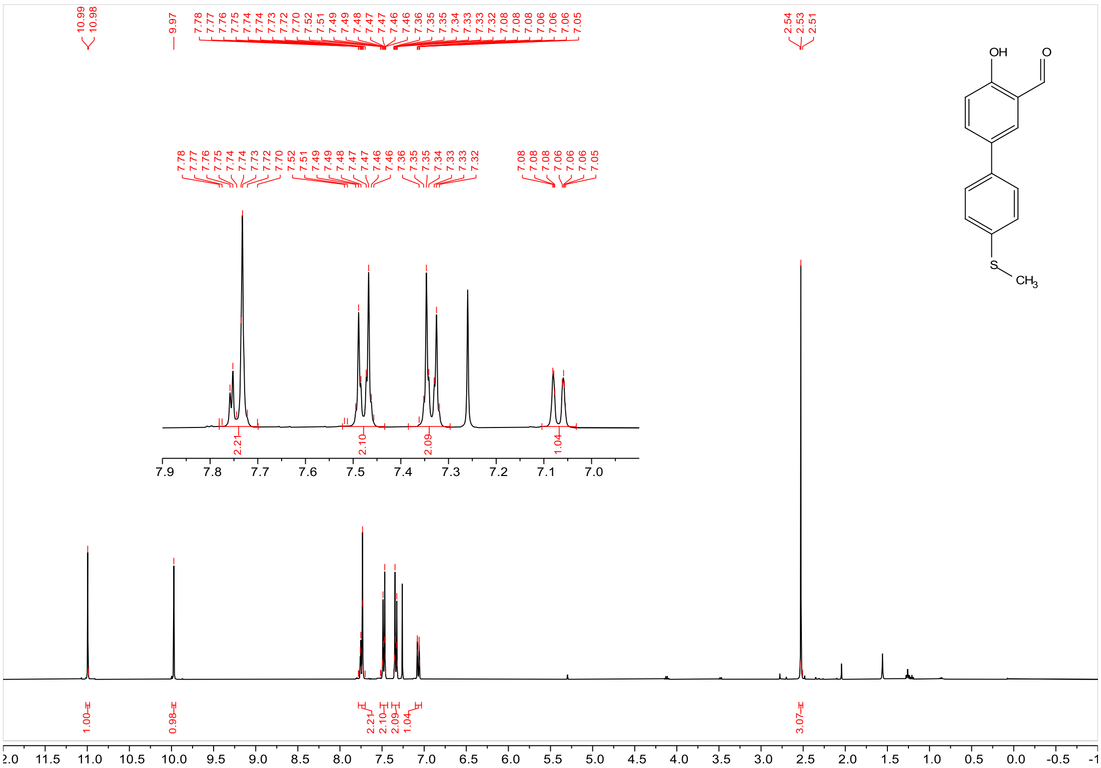

Figure S36 ^{1}H NMR Spectrum of **19** in $CDCl_3$.

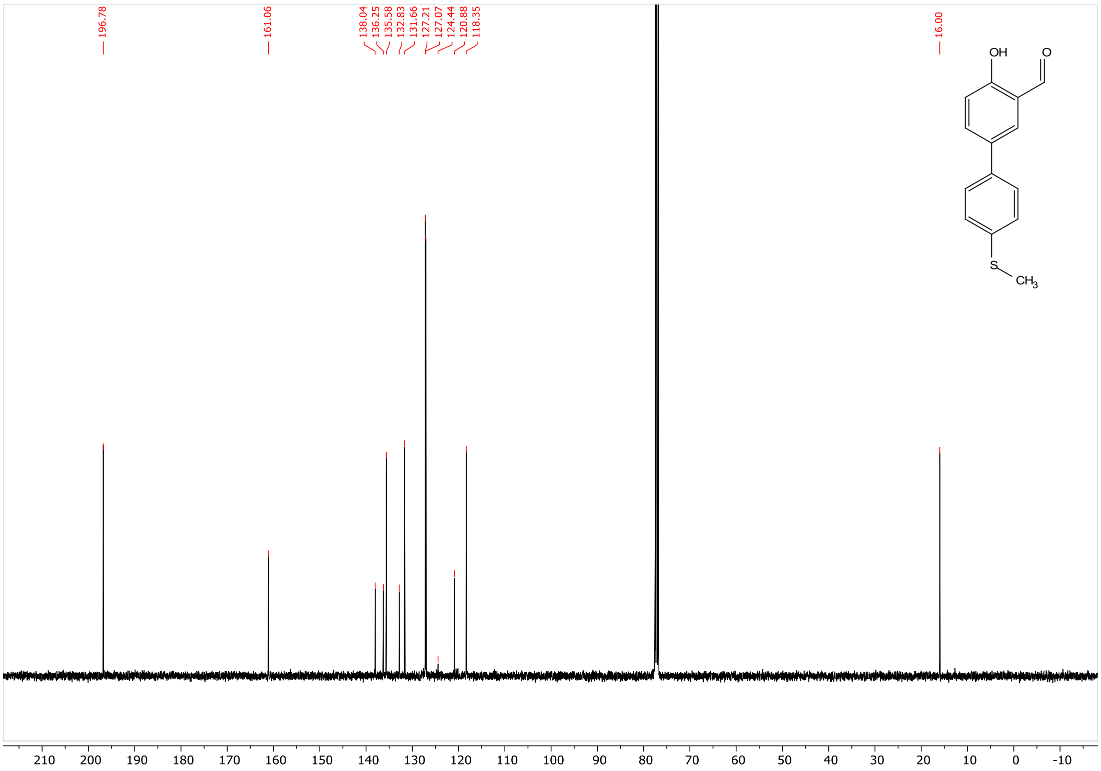

Figure S37 ^{13}C NMR Spectrum of **19** in $CDCl_3$.

4-(3,3-dimethyl-2,3-dihydrobenzo[*b*]thiophen-5-yl)-2-hydroxybenzaldehyde (**20**):

A Schlenk vessel was charge with **17** (148 mg, 0.597 mmol), **15** (150 mg, 0.721 mmol), Na_2CO_3 (154 mg, 1.45 mmol) and evacuated for 20 min. Dimethoxyethane (5 mL) and water (1 mL) were added, and the solution was freeze, pump, thawed three times. Pd(dppf)Cl_2.CH_2Cl_2 (20 mg, 0.024 mmol) was added, and the solution was refluxed for 4 h. The solution was cooled to r.t. and the solvent removed under reduced pressure. The residue was extracted with EtOAc / 2M HCl (3 x 20 mL). After removal of the aqueous layer the organic layer was washed with water (3 x 30 mL) and brine (30 mL), dried over Na_2SO_4, filtered through a celite pad and the solvent was removed to give a dark orange oil. The residue was filtered through a small silica plug using 1:1 CH_2Cl_2:Hexanes to give a yellow powder (160 mg, 94%). Further recrystallisation from hot hexanes gives faint yellow needles (116 mg, 67%).

m.p.: 113-114°C

^{1}H NMR ($CDCl_3$, 400 MHz) δ/ ppm: 11.13 (s, 1H, OH), 9.90 (s, 1H, CHO), 7.59 (d, *J* = 8.0 Hz, 1H, H6), 7.40 (dd, *J* = 2.0, 8.0 Hz, 1H, H6'), 7.29 (d, *J* = 2.0 Hz, 1H, H4'), 7.27 (d, *J* = 8.0 Hz, 1H, H7'), 7.23 (dd, *J* = 1.7, 8.0 Hz, 1H, H5), 7.19 (d, *J* = 1.7 Hz, 1H, H3), 3.24 (s, 2H, H2'), 1.43 (s, 6H, *gem*-CH_3).

$^{13}C\{^{1}H\}$ NMR ($CDCl_3$, 101 MHz) δ/ ppm: 195.98 (CHO), 162.12 (C2), 149.92 (C3a'), 149.13 (C4), 142.49 (C7a'), 135.95 (C5'), 134.22 (C6), 126.79 (C6'), 122.96 (C7'), 121.70 (C4'), 119.50 (C1), 118.69 (C5), 115.40 (C3), 47.60 (C3'), 47.44 (C2'), 27.59 (*gem*-CH_3).

FTIR (ATR, neat) ν / cm^{-1}: 3040 (br., OH), 1638 (CHO).

HRMS (TOF AP(+)) *m/z*: $[M+H]^+$ Calcd for $C_{17}H_{17}O_2S^+$ 285.0944, Found 285.0941.

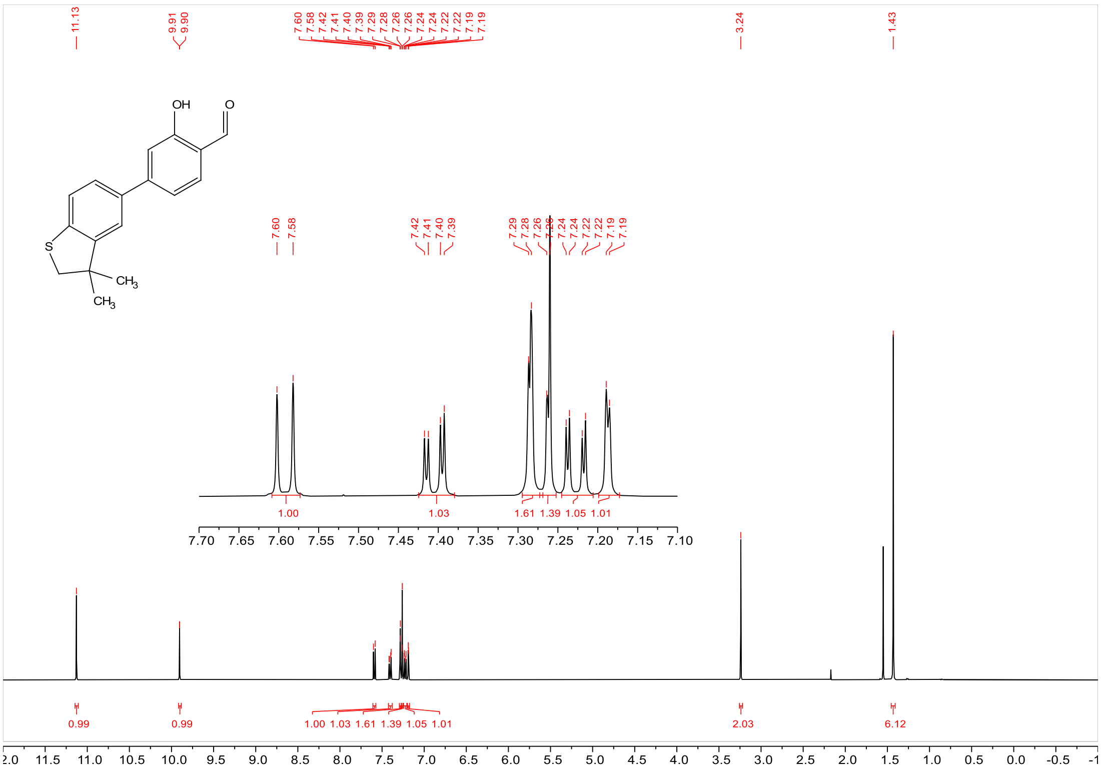

Figure S38 ^{1}H NMR Spectrum of **20** in $CDCl_3$.

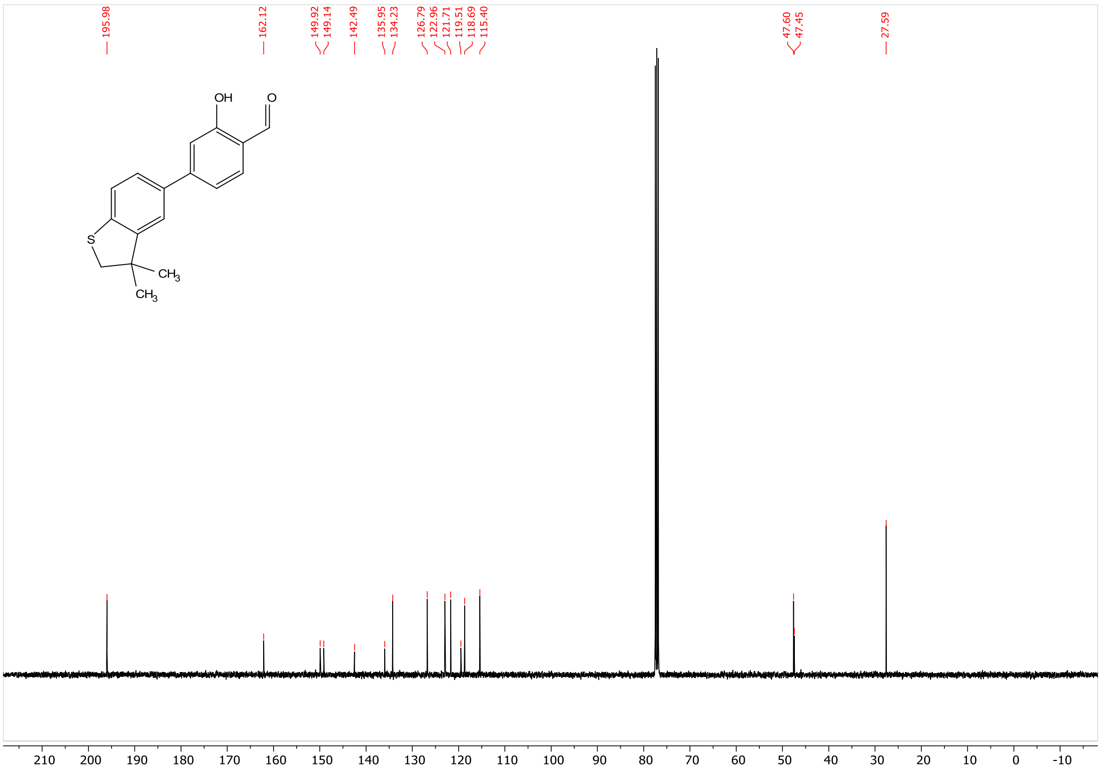

Figure S39 ^{13}C NMR Spectrum of **20** in $CDCl_3$.

3-hydroxy-4'-(methylthio)-[1,1'-biphenyl]-4-carbaldehyde (**21**):

17 (138 mg, 0.55 mmol), **16** (110 mg, 0.65 mmol) and Na_2CO_3 (116 mg) were charged to a Schlenk vessel. Dimethoxyethane (5 mL) and water (2 mL) were added. The solution was degassed by FPT (x3). Pd(dppf)Cl_2.CH_2Cl_2 (22 mg, 0.027 mmol) was then added, and the solution was refluxed for 4 h. After the cooling to room temperature, volatiles were removed under reduced pressure. The dark residue was extracted 3 times EtOAc (10 mL) and 2M HCl (10 mL). The organic phase was washed with a further 2 x 30 mL water. The aqueous phase was extracted once more with EtOAc (30 mL). The combined organic layers were washed with brine (30 mL), dried over sodium sulphate, filtered, and the solvent removed on a rotary evaporator. The dark brown-orange solid was subject to flash column chromatography (silica, CH_2Cl_2:Hexanes, 1:1) to give a bone coloured solid **21** (110 mg, 82%).

m.p.: 132-133°C

^{1}H NMR ($CDCl_3$, 400 MHz) δ/ ppm: 11.12 (s, 1H, OH), 9.91 (s, 1H, CHO), 7.60 (d, *J* = 8.0 Hz, 1H, H5), 7.56 (d, *J* = 8.4 Hz, 2H, H2’), 7.33 (d, *J* = 8.4 Hz, 2H, H3’), 7.24 (dd, *J* = 1.7, 8.0 Hz, 1H, H6), 7.19 (d, *J* = 1.4 Hz, 1H, H2), 2.53 (s, 3H, SCH_3);

$^{13}C\{^1H\}$ NMR ($CDCl_3$, 101 MHz) δ/ ppm: 196.03 (CHO), 162.14 (C3), 149.28 (C1), 140.28 (C4’), 135.87 (C1’), 134.28 (C5), 127.77 (C2’), 126.70 (C3’), 119.63 (C4), 118.62 (C6), 115.46 (C2), 15.62 (SCH_3);

FTIR (ATR, neat) ν / cm^{-1}: 3205 (OH), 2922, 2858, 1663 (C=O), 1622, 1592, 1567, 1540, 1508, 1481, 1428;

HRMS (TOF AP(+)) *m/z*: $[M+H]^+$ Calcd for $C_{14}H_{13}O_2S^+$ 245.0631, Found 245.0631.

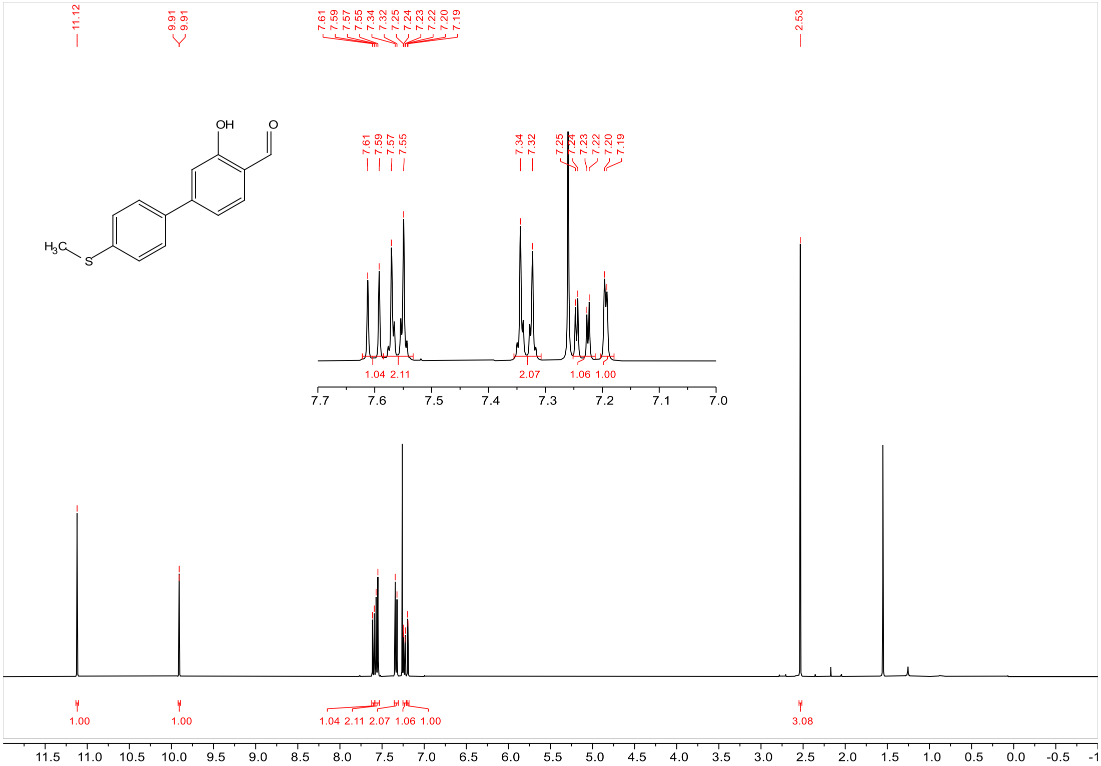

Figure S40 ^{1}H NMR Spectrum of **21** in $CDCl_3$.

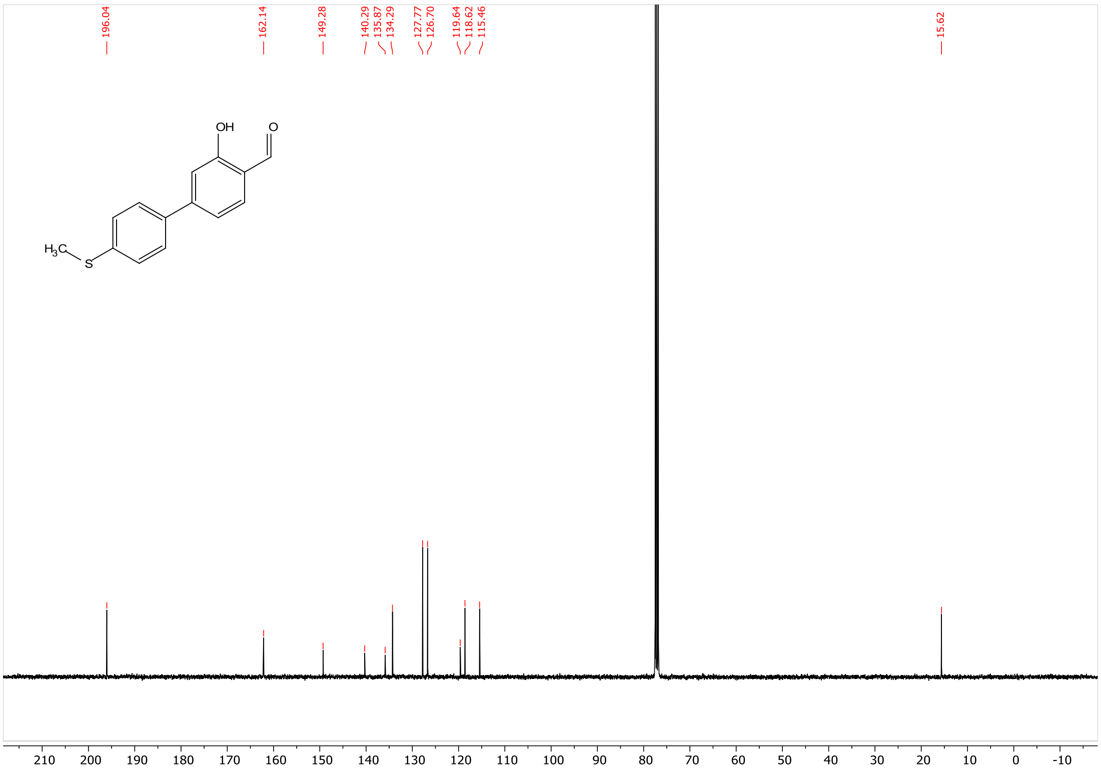

Figure S41 ^{13}C NMR Spectrum of **21** in $CDCl_3$.

5-(3,3-dimethyl-2,3-dihydrobenzo[*b*]thiophen-5-yl)-2,3,3-trimethyl-3*H*-indole (**22**): 5-bromo-2,3,3-trimethyl-3*H*-indole **8** (100 mg, 0.420 mmol), (3,3-dimethyl-2,3-dihydrobenzo[*b*]thiophen-5-yl)boronic acid **15** (96 mg, 0.46 mmol), Na_2CO_3 (133 mg, 1.25 mmol) was added dimethoxyethane (5 mL) and water (1 mL). The reaction mixture was subject to FPT x 3 and backfilled with nitrogen. Pd(dppf)Cl_2.CH_2Cl_2 (20 mg, 0.024 mmol) was added the solution was refluxed overnight under argon. The reaction was cooled to room temperature and the solvent was removed under reduced pressure. The residue was extracted with EtOAc (3 x 30 mL) and washed with water (3 x 30 mL). The aqueous washings were extracted with EtOAc (30 mL) and the combined organic layers were washed with brine (30 mL). The organic layer was then dried over Na_2SO_4, filtered, and evaporated to dryness on a rotary evaporator. The crude product was subject to flash chromatography (silica, Hexanes:EtOAc, 1:1) to afford a yellow sticky solid **22** (122 mg, 90%).

^{1}H NMR (CD_2Cl_2, 400 MHz) δ/ ppm: 7.60 (d, *J* = 8.0 Hz, 1H, H7), 7.51 (dd, *J* = 1.8, 8.0 Hz, 1H, H6), 7.45 (d, *J* = 1.7 Hz, 1H, H4), 7.39 (dd, *J* = 1.7, 8.1 Hz, 1H, H6’), 7.27 – 7.28 (m, 2H, H7’and H4’), 3.25 (s, 2H, H2’), 2.33 (s, 3H, NCCH$_3$), 1.46 (s, *gem*’-CH$_3$), 1.38 (s, 3H, *gem*-CH$_3$);

$^{13}C\{^{1}H\}$ NMR (CD_2Cl_2, 101 MHz) δ/ ppm: 188.31 (C2), 153.05 (C7a), 148.75 (C3a’), 146.43 (C3a), 139.69 (C7a’), 138.60 (C5), 138.29 (C5’), 126.66 (C6’), 126.62 (C6), 122.74 (C7’), 121.72 (C4’), 120.11 (C7 and C4), 53.88 (C3), 47.59 (C3’), 47.44 (C2’), 27.57 (*gem*’-CH_3), 23.33 (*gem*-CH_3), 15.62 ($NCCH_3$);

FTIR (ATR, neat) ν / cm^{-1}: 2959, 2924, 2863, 1688, 1574 (C=N), 1455, 1428;

HRMS (TOF AP(+)) *m/z*: $[M+H]^+$ Calcd for $C_{21}H_{24}NS^+$ 322.1624, Found 322.1617.

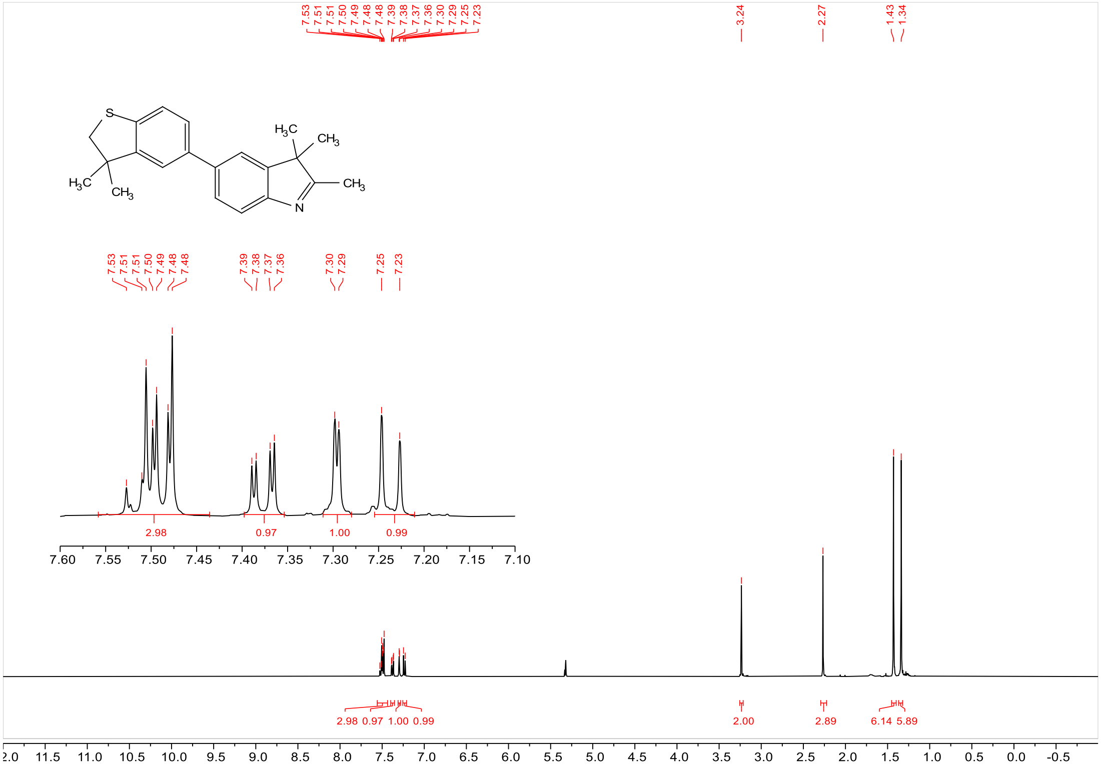

Figure S42 ^{1}H NMR Spectrum of **22** in $CDCl_3$.

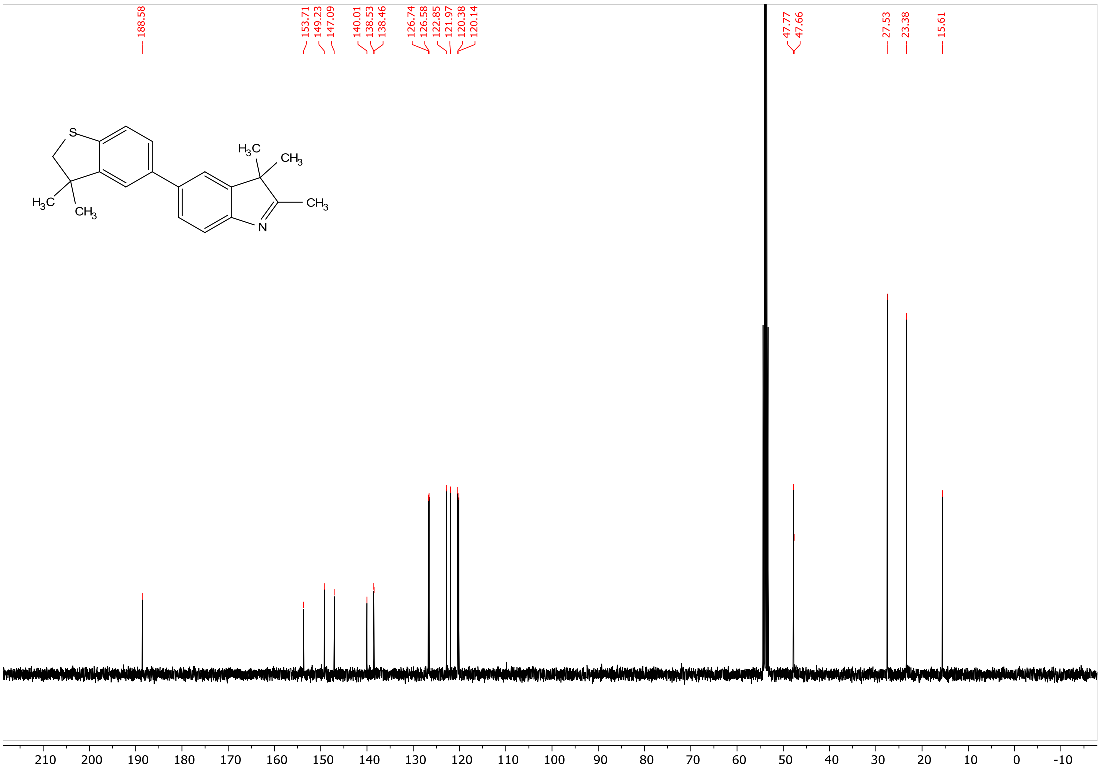

Figure S43 ^{13}C NMR Spectrum of **22** in $CDCl_3$.

2,3,3-trimethyl-5-(4-(methylthio)phenyl)-3*H*-indole (**23**):

Na_2CO_3 (160 mg, 1.51 mmol), **8** (100 mg, 0.42 mmol) and **16** (105 mg, 0.63 mmol) were suspended in dimethoxyethane (4 mL) and H_2O (1 mL). The solution was degassed by FPT x 3. Pd(dppf)Cl_2.CH_2Cl_2 (22 mg, 0.027 mmol) was then added to the solution was refluxed overnight. After cooling to room temperature, the solvent was removed, and the residue was triturated with aliquots of diethyl ether (5 x 5 mL) until the extracts were colourless. The organic layer was washed with H_2O (3 x 25 mL). The aqueous layer was then extracted with EtOAc (25 mL). The organic layer was then washed with brine solution (25 mL), dried over sodium sulphate, filtered through a small celite plug and the solvent was removed on a rotary evaporator. Rapid silica column chromatography using 1:1 EtOAc:Hexanes gives **14** as a yellow powder (102 mg, 86%) after removal of the eluent.

m.p.: 122 -124°C

^{1}H NMR ($CDCl_3$, 400 MHz) δ/ ppm: 7.58 (d, *J* = 7.9 Hz, 1H, H7), 7.53 (d, *J* = 8.4 Hz, 2H, H2'), 7.50 (dd, *J* = 7.9, 1.8 Hz, 1H, H6), 7.46 (d, *J* = 1.8 Hz, 1H, H4), 7.33 (d, *J* = 8.4 Hz, 1H, H3'), 2.52 (s, 3H, SCH_3), 2.30 (s, 3H, $NCCH_3$), 1.35 (s, 6H, *gem*-CH_3);

$^{13}C\{^{1}H\}$ NMR ($CDCl_3$, 101 MHz) δ/ ppm: 188.43 (C2), 153.30 (C7a), 146.50 (C3a), 138.43 (C1'), 137.89 (C5), 137.43 (C4'), 127.68 (C2'), 127.15 (C3'), 126.57 (C6), 120.17 (C7), 120.06 (C4), 53.89 (C3), 23.33 (*gem*-CH_3), 16.12 (SCH_3), 15.64 ($NCCH_3$);

FTIR (ATR, neat) ν / cm^{-1}: 2957, 2925, 2861, 1915, 1696, 1573 (C=N), 1497, 1458, 1437, 1398,

HRMS (TOF ES(+)) *m/z*: $[M+H]^+$ Calcd for $C_{18}H_{20}NS^+$ 282.1311, Found 282.1311.

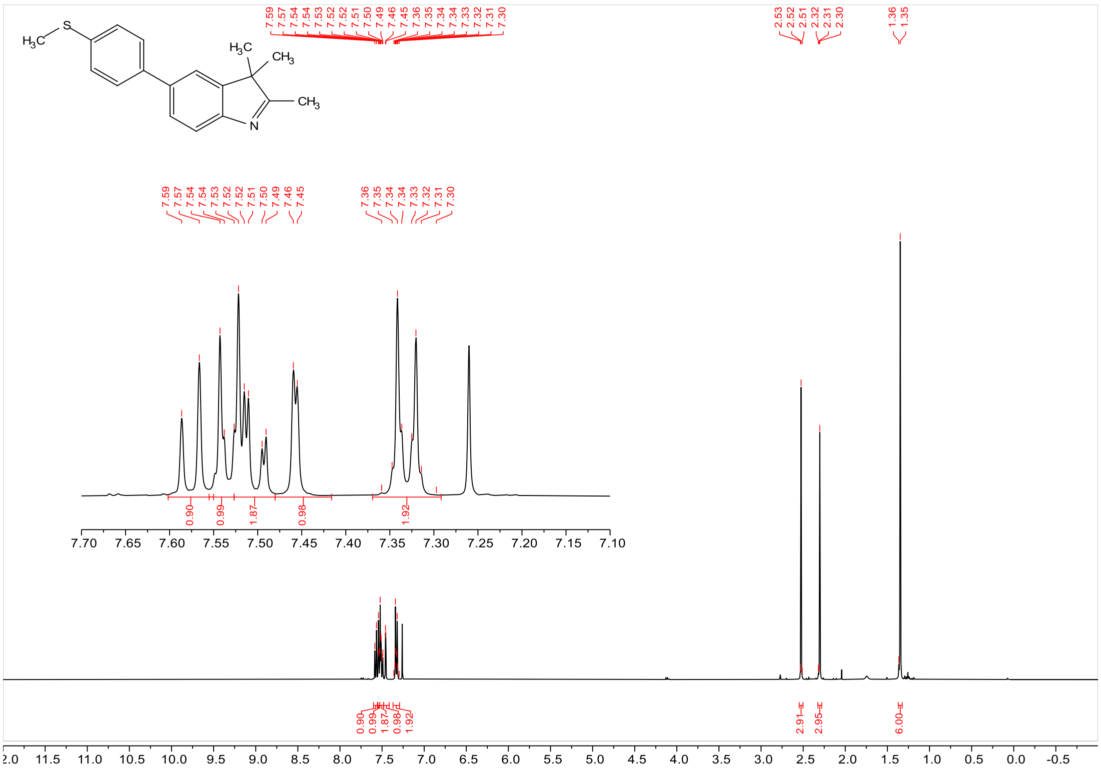

Figure S44 ^{1}H NMR Spectrum of **23** in $CDCl_3$.

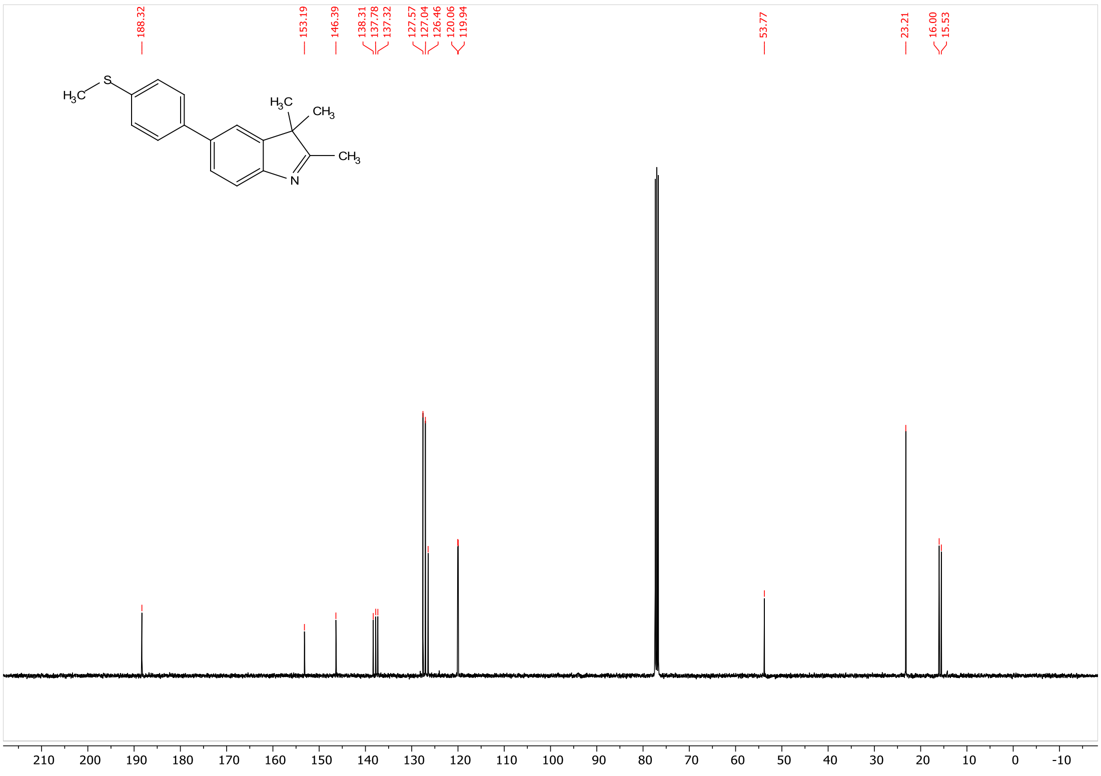

Figure S45 ^{13}C NMR Spectrum of **23** in $CDCl_3$.

5-(3,3-dimethyl-2,3-dihydrobenzo[*b*]thiophen-5-yl)-1,2,3,3-tetramethyl-3*H*-indol-1-ium iodide (**24**):

5-(3,3-dimethyl-2,3-dihydrobenzo[b]thiophen-5-yl)-2,3,3-trimethyl-3H-indole **22** (244 mg, 0.759 mmol) was dissolved in acetonitrile (20 mL) and sparged with nitrogen for 20 min. Iodomethane (0.1 mL, excess) was added and the mixture was refluxed for 18 h. The solvent was concentrated until a precipitate formed and then diethyl ether was added to complete precipitation of a beige solid that was collected by vacuum filtration. Recrystallisation from hot ethanol affords purple needle-like crystals of **24** (278 mg, 79%).

m.p.: 249-252°C

^{1}H NMR (DMSO-d$_6$, 400 MHz) δ/ ppm: 8.15 (s, 1H, H4), 7.94 (d, *J* = 8.4 Hz, 1H, H7), 7.90 (dd, *J* = 1.4, 8.4 Hz, 1H, H6), 7.56 (dd, *J* = 1.7, 8.7 Hz, 1H, H6'), 7.54 (s, 1H, H4') 7.34 (dd, *J* = 8.7 Hz, 1H, H7'), 3.99 (s, 3H, NCH_3), 3.26 (s, 2H, H2'), 2.77 (s, 3H, $NCCH_3$), 1.59 (s, 6H, *gem*-CH_3), 1.41 (s, 6H, *gem*'-CH_3);

^{13}C{^{1}H} NMR (DMSO-d$_6$, 101 MHz) δ/ ppm: 195.61 (C2), 149.06 (C3a'), 142.41 (C3a), 141.33 (C5), 141.13 (C7a), 140.48 (C7a'), 135.39 (C5'), 126.85 (C6), 126.52 (C6'), 122.61 (C7'), 121.62

(C4’), 121.33 (C4), 115.39 (C7), 54.02 (C3), 46.97 (C3’), 46.89 (C2’), 34.73 (NCH_3), 27.01 (*gem*’-CH_3), 21.78 (*gem*-CH_3), 14.07 ($NCCH_3$);

FTIR (ATR, neat) ν / cm^{-1}: 3513, 2964, 2911, 1615, 1595 (N=C), 1459, 822;

HRMS (TOF AP(+)) *m/z*: $[M-I]^+$ Calcd for $C_{22}H_{26}NS^+$ 336.1781, Found 336.1772.

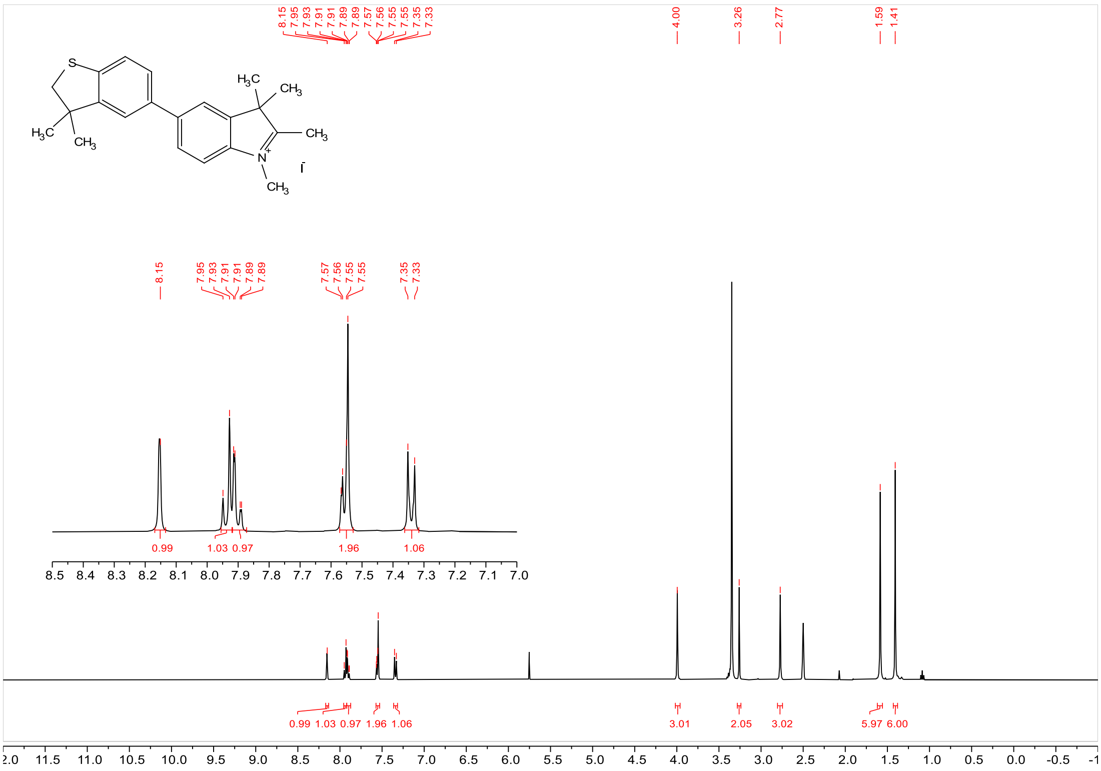

Figure S46 ^{1}H NMR Spectrum of **24** in DMSO-d_6.

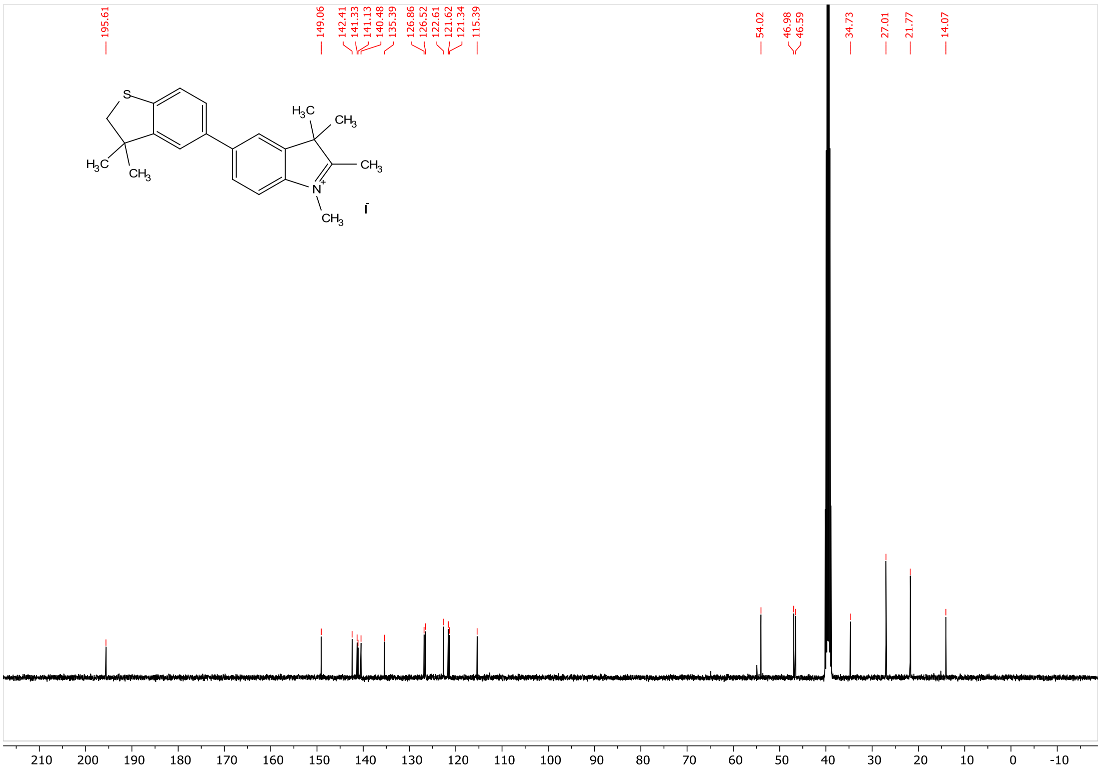

Figure S47 ^{13}C NMR Spectrum of **24** in DMSO-d$_6$.

1,2,3,3-tetramethyl-5-(4-(methylthio) phenyl)-3*H*-indol-1-ium iodide (**25**):

23 (50 mg, 0.18 mmol) was dissolved in anhydrous CH_3CN (5 mL). The yellow solution was sparged with nitrogen for 20 min. CH_3I (0.02 mL, 0.3 mmol) was added and the mixture was heated under reflux for 12 h. The red solution was cooled to room temperature and then placed in the freezer for 24 h. A dark red microcrystalline product **25** was then collected and washed with aliquots of diethyl ether (63 mg, 83%).

m.p.: 260 -262°C

^{1}H NMR (DMSO-d$_6$, 400 MHz) δ/ ppm: 8.17 (s, 1H, H4), 7.96 (d, *J* = 8.8 Hz, 1H, H7), 7.91 (dd, *J* = 1.2, 8.8 Hz, 1H, H6), 7.75 (d, *J* = 8.4 Hz, 1H, H2’), 7.40 (d, *J* = 8.4 Hz, 2H, H3’), 3.99 (s, 3H, NCH_3), 2.77 (s, 3H, $NCCH_3$), 2.50 (s, 3H, SCH_3), 1.58 (s, 6H, *gem*-CH_3);

^{13}C{^{1}H} NMR (DMSO-d$_6$, 101 MHz) δ/ ppm: 195.77 (C2), 142.47 (C3a), 141.30 (C7a), 140.75 (C5), 138.76 (C4’), 135.08 (C1’), 127.54 (C2’), 126.75 (C6), 126.29 (C3’), 121.27 (C4), 115.50 (C7), 54.03 (C3), 34.69 (NCH_3), 21.75 (*gem*-CH_3), 14.56 (SCH_3), 14.03 ($NCCH_3$);

FTIR (ATR, neat) ν / cm^{-1}: 2971, 2908, 1630, 1615, 1594 (C=N), 1475, 1447, 1400, 1364

HRMS (TOF ES(+)) *m/z*: $[M-I]^+$ Calcd for $C_{19}H_{22}NS^+$ 296.1468, Found 296.1474.

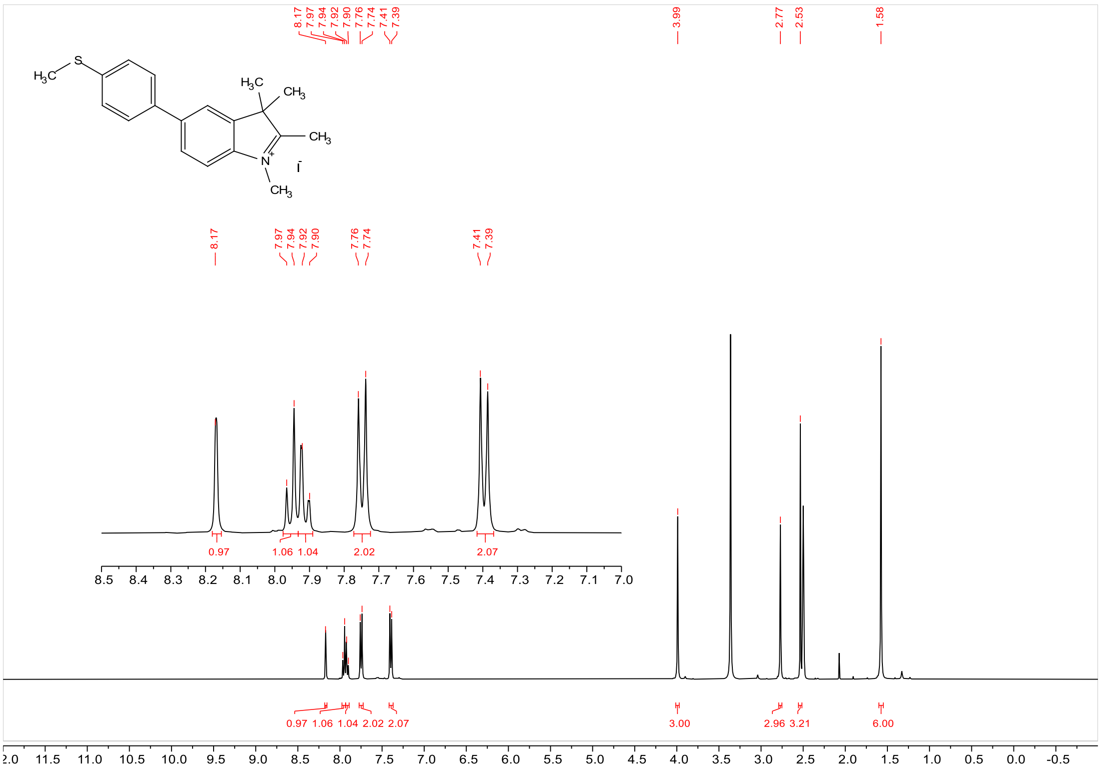

Figure S48 ^{1}H NMR Spectrum of **25** in DMSO-d$_6$.

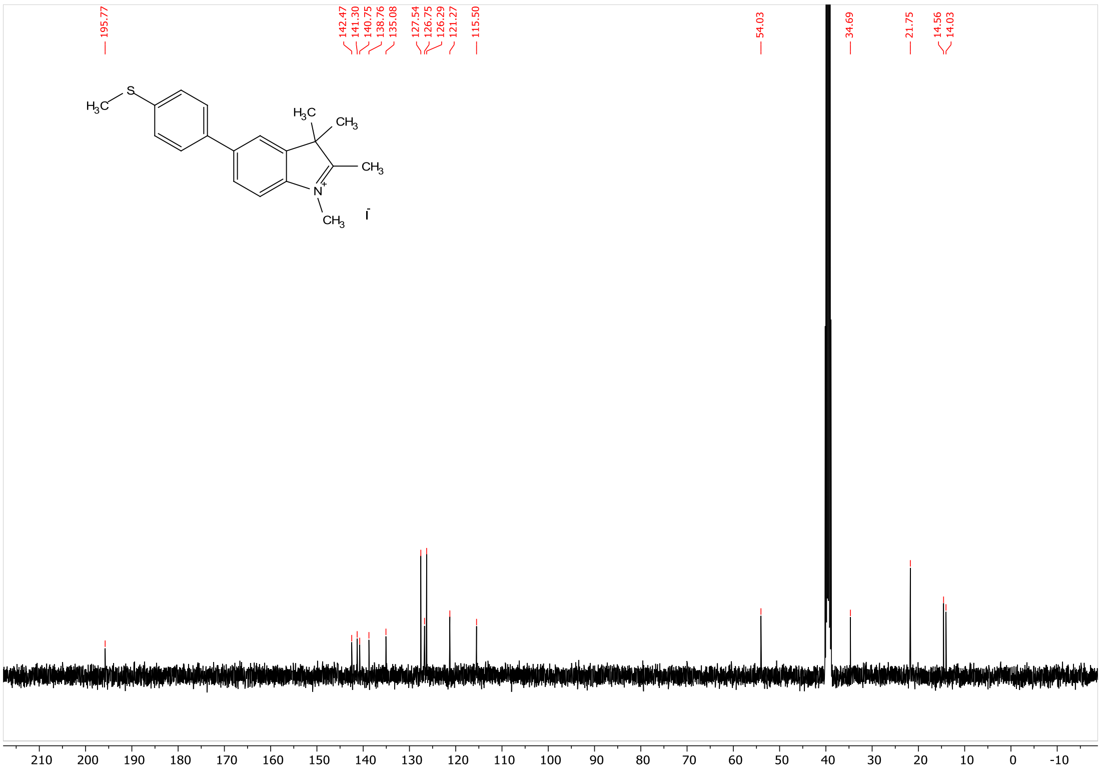

Figure S49 ^{13}C NMR Spectrum of **25** in DMSO-d$_6$.

1',3',3'-trimethyl-5',6-bis(4-(methylthio)phenyl)spiro[chromene-2,2'-indoline] (**SP8**):

Anhydrous ethanol (10 mL) and piperidine (0.5 mL) were sparged with argon for 20 min. Following this, 1,2,3,3-tetramethyl-5-(4-(methylthio)phenyl)-3H-indol-1-ium iodide **25** (38 mg, 0.090 mmol) and 4-hydroxy-4'-(methylthio)-[1,1'-biphenyl]-3-carbaldehyde **19** (26 mg, 0.11 mmol) were added and the solution was refluxed for 5 h, and cooled overnight to room temperature. The solution goes from a deep orange to yellow and a precipitate slowly forms. The solution was cooled in an ice bath and the yellow precipitate was filtered and washed with cold ethanol and hexane (26 mg). The supernatant solution was evaporated to dryness and the residue was taken up in EtOAc (30 mL) and washed with $NaHCO_3$, water and brine. The organic layer was then dried over Na_2SO_4, filtered, and evaporated. The residue was then run through a short alumina column using CH_2Cl_2 and hexanes (1:1). A yellow fraction was evaporated to give a pale yellow amorphous solid (12 mg). 38 mg total yield (0.073 mmol), 81% yield. Crystals for Single X-ray crystallography were grown by slow diffusion of hexane into a CH_2Cl_2 solution.

m.p.: 192-194°C

^{1}H NMR (CD_2Cl_2, 400 MHz) δ/ ppm: 7.53 (d, *J* = 8.4 Hz, 2H, thio-2’), 7.48 (d, *J* = 8.4 Hz, 2H, thio-2), 7.41 (dd, *J* = 2.0, 8.0 Hz, 1H, H6’), 7.34 (dd, *J* = 2.2, 8.4 Hz, 1H, H7), 7.29 – 7.33 (m, 6H, H5, H4’,thio-3, thio-3’), 6.97 (d, *J* = 10.4 Hz, 1H, H4), 6.78 (d, *J* = 8.4 Hz, 1H, H8), 6.60 (d, *J* = 8.0 Hz. 1H, H7’), 5.78 (d, *J* = 10.4 Hz, 1H, H3), 2.79 (s, 3H, NCH_3), 2.52 (s, 3H, SCH_3), 2.51 (s, 3H, SCH_3),1.37 (s, 3H, *gem*-CH_3), 1.23 (s, 3H, *gem*-CH_3);

^{13}C{^{1}H} NMR (CD_2Cl_2, 101 MHz) δ/ ppm: 154.36 (C8a), 148.28 (C7a’), 139.01 (thio-1‘), 138.03 (C3a’), 137.71 (thio-1), 137.42 (thio-4), 136.62 (thio-4’), 132.96 (C6), 132.08 (C5‘), 130.00 (C4), 128.42 (C7), 127.41 (thio-3’), 127.24 (thio-3), 127.21 (thio-2’), 127.19 (thio-2), 126.58 (C6’), 125.37 (C5), 120.62 (C4’), 119.96 (C3), 119.53 (C4a), 115.55 (C8), 107.39 (C7‘), 105.31 (C2), 52.26 (C3’), 29.19 (NCH_3), 26.11 (*gem*-CH_3), 20.18 (*gem*-CH_3), 16.25 (SCH_3), 16.06 (SCH_3);

FTIR (ATR, neat) ν / cm^{-1}: 2919, 1645 (C=C), 1610, 1513, 1477;

HRMS (TOF ES(+)) *m/z*: $[M+H]^+$ Calcd for $C_{33}H_{32}NOS_2^+$ 522.1920, Found 522.1920.

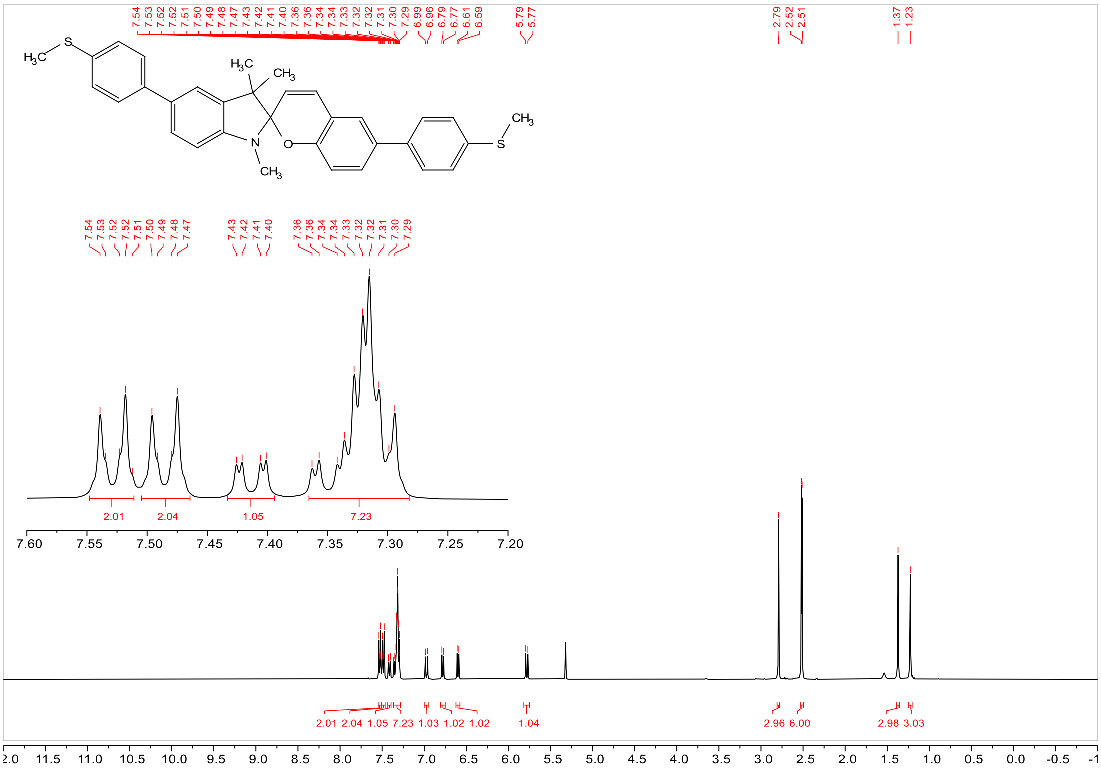

Figure S50 ^{1}H NMR Spectrum of **SP8** in CD_2Cl_2.

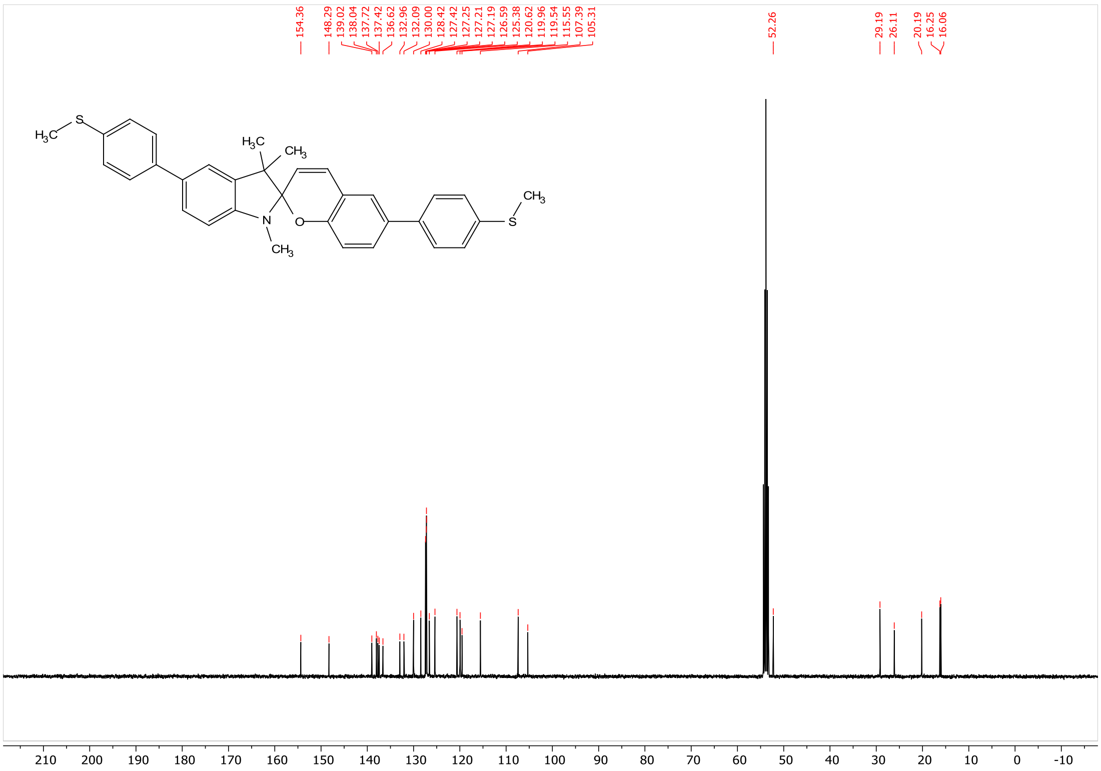

Figure S51 ^{13}C NMR Spectrum of **SP8** in CD_2Cl_2.

1',3',3'-trimethyl-5',7-bis(4-(methylthio)phenyl)spiro[chromene-2,2'-indoline] (**SP9**): Anhydrous ethanol (10 mL) was sparged with nitrogen for 20 min. **25** (85 mg, 0.20 mmol), **21** (60 mg, 0.24 mmol), and piperidine (0.02 mL, 0.2 mmol) were added and the solution was refluxed for 5 h. The green solution was cool to r.t. overnight. The solution become orange as it was cooling, and a bone coloured microcrystalline solid formed. The solution was cooled in an ice-bath and the precipitate was collected by vacuum filtration and washed with a little cold ethanol. Yield 88 mg (0.17 mmol), 85%.

m.p.: 174-176°C

^{1}H NMR ($CDCl_3$, 400 MHz) δ/ ppm: 7.51 (d, *J* = 8.5 Hz, 2H, thio-3 or thio-3'), 7.46 (d, *J* = 8.6 Hz, 2H, thio-3 or thio-3'), 7.40 (dd, *J* = 1.9, 8.0 Hz, 1H, H6'), 7.31 (d, *J* = 8.5 Hz, 2H, thio-2 or thio-2'), 7.29 (d, *J* = 1.9 Hz, 1H, H4'), 7.26 (d, *J* = 8.6 Hz, 2H, thio-2 or thio-2'), 7.11 (d, *J* = 7.8 Hz, 1H, H5), 7.07 (dd, *J* = 1.7, 7.8 Hz, 1H, H6), 7.01 (d, *J* = 1.7 Hz, 1H, H8), 6.91 (d, *J* = 10.2 Hz, 1H, H4), 6.59 (d, *J* = 8.2 Hz, 1H, H7'), 5.71 (d, *J* = 10.2 Hz, 1H, H3), 2.80 (s, 3H, NCH_3), 2.52 (s, 3H, SCH_3), 2.49 (s, 3H, SCH_3), 1.39 (s, 3H, *gem*-CH_3), 1.23 (s, 3H, *gem*-CH_3);

^{13}C{^{1}H} NMR ($CDCl_3$, 101 MHz) δ/ ppm: 154.92 (C8a), 147.94 (C7a'), 142.10 (C7), 139.11.(thio-4 or thio-4'), 137.98 (C3a'), 137.69 (thio-1 or thio-1'), 137.61 (thio-4 or thio-4'), 136.00 (thio-1 or thio-1'), 132.01 (C5'), 129.33 (C4), 127.42 (thio-2 or thio-2'), 127.26 (thio-3 or thio-3'), 127.20 (C5), 127.17 (thio-3 or thio-3'), 126.91 (thio-2 or thio-2'), ,126.52 (C6'), 120.45 (C5'), 119.19 (C4), 118.60 (C6), 117.96 (C4a), 113.25 (C8), 107.15 (C7'), 104.71 (C2), 52.00 (C3'), 29.20 (NCH_3), 26.14 (*gem*-CH_3), 20.39 (*gem*-CH_3), 16.41 (SCH_3), 15.93 (SCH_3);

FTIR (ATR, neat) ν / cm^{-1}: 2955, 2915, 1638 (C=C), 1612, 1566, 1542, 1510, 1481, 961 (C_{spiro}-O);

HRMS (TOF AP(+)) *m/z*: $[M+H]^+$ Calcd for $C_{33}H_{32}NOS_2^+$ 522.1920, Found 522.1901.

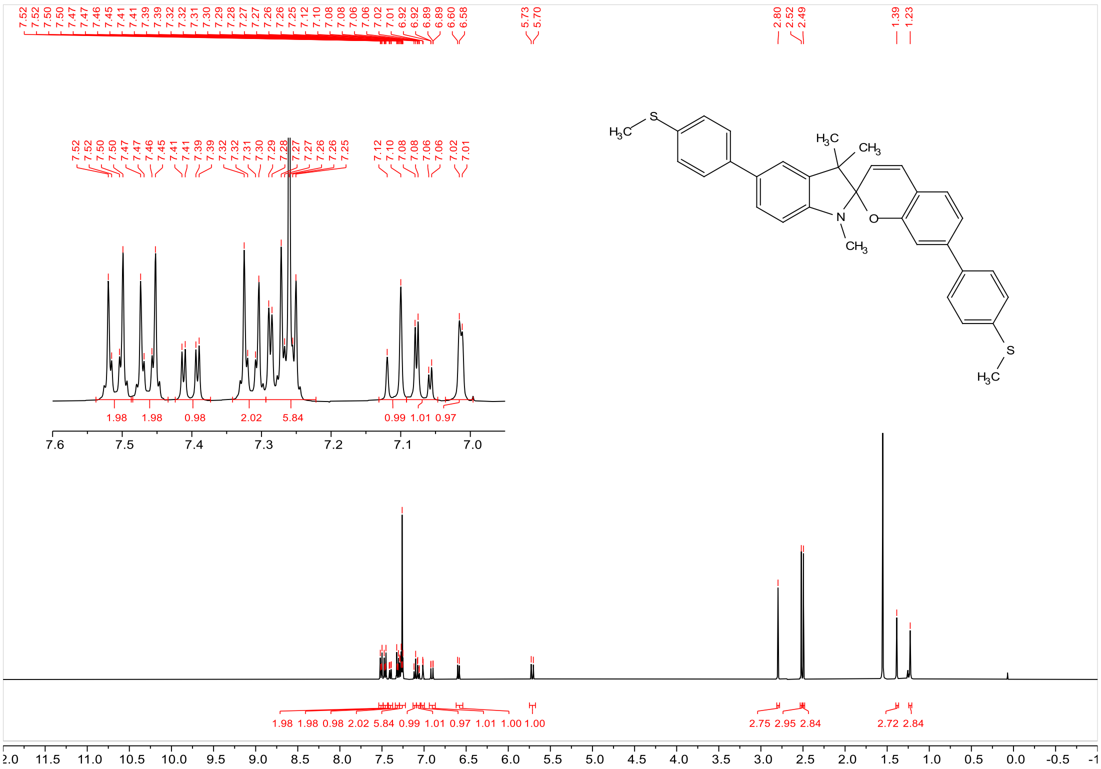

Figure S52 ^{1}H NMR Spectrum of **SP9** in $CDCl_3$.

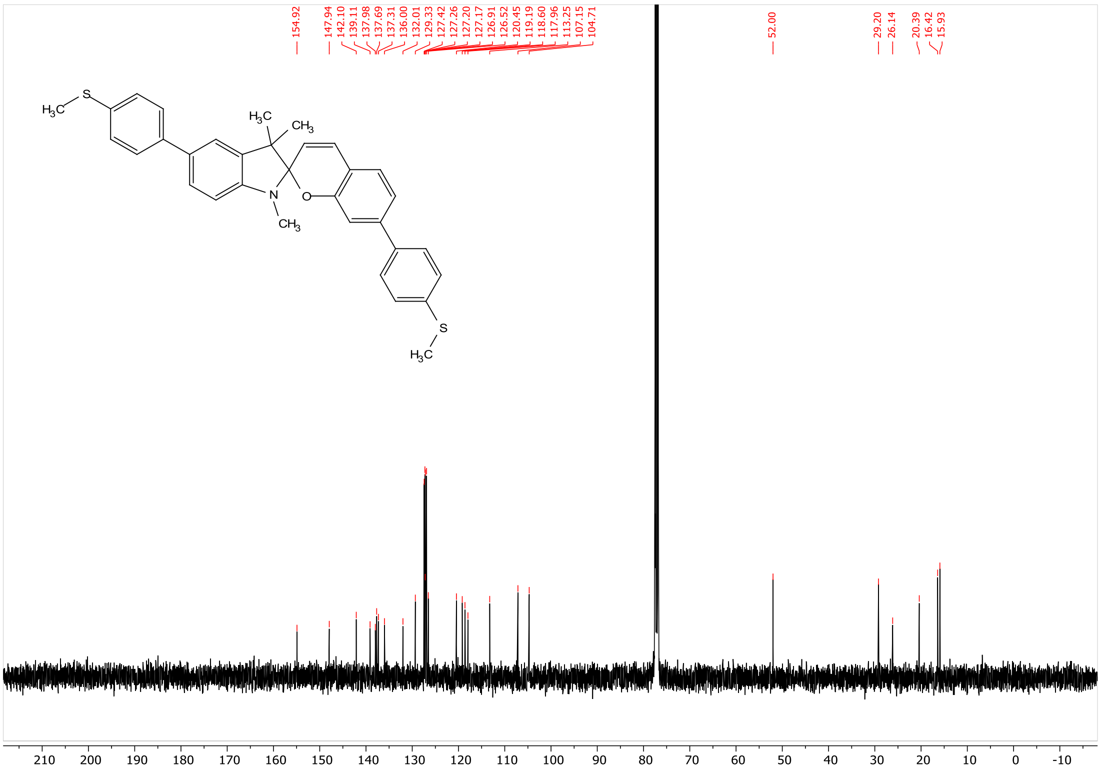

Figure S53 ^{13}C NMR Spectrum of **SP9** in $CDCl_3$.

5′,6-bis(3,3-dimethyl-2,3-dihydrobenzo[*b*]thiophen-5-yl)-1',3',3'-trimethylspiro-[chromene-2,2'-indoline] (**SP10**):

Piperidine (0.02 mL, 0.2 mmol) and EtOH (10 mL) were sparged with nitrogen for 20 min in a Schlenk flask. 5-(3,3-dimethyl-2,3-dihydrobenzo[b]thiophen-5-yl)-2,3,3-trimethyl-3*H*-indol-1-ium iodide **24** (34 mg, 0.073 mmol) and 5-(3,3-dimethyl-2,3-dihydrobenzo[*b*]thiophen-5-yl)-2-hydroxybenzaldehyde (**18**) (36 mg , 0.13 mmol) were added and the solution was refluxed for 3 h. Cooling to room temperature induced precipitation of a fine yellow solid. The solvent was removed under reduced pressure. The residue was taken up in EtOAc (30 mL) and washed with $NaHCO_3$, water and brine. The organic layer was dried over sodium sulphate, filtered, and evaporated to dryness. The orange residue was purified by column chromatography (alumina, 2:8 CH_2Cl_2:Hexanes) to give a white powder of **SP10** (35 mg, 80%). Crystal for X-ray diffraction studies were grown by slow evaporation of a $CHCl_3$ solution.

m.p.: 259-260°C;

^{1}H NMR (CD_2Cl_2, 400 MHz) δ/ ppm: 7.38 (dd, *J* = 1.8, 8.1 Hz, 1H, H6′), 7.34 (dd, *J* = 1.9, 8.0 Hz, 1H, H6′′ or H6′′′), 7.33 (dd, *J* = 2.3, 8.4 Hz, 1H, H7), 7.31 (dd, *J* = 1.8, 8.0 Hz, 1H, H6′′ or H6′′′), 7.29 (d, *J* = 2.3 Hz, 1H, H5), 7.28 (d, *J* = 1.9 Hz, 1H, H4′), 7.27 (d, *J* = 1.8 Hz, 1H, H4′′ or H4′′′), 7.23 (d, *J* = 1.8 Hz, 1H, H4′′ or H4′′′), 7.21 (d, *J* = 8.0 Hz, 1H, H7′′ or H7′′′), 7.20 (d, *J* = 8.0 Hz, 1H, H7′′ or H7′′′), 6.98 (d, *J* = 10.2 Hz, 1H, H4), 6.77 (d, *J* = 8.4 Hz, 1H, H8), 6.59 (d, *J* = 8.1 Hz, 1H, H7′), 5.78 (d, *J* = 10.2 Hz, 1H, H3), 3.22 (s, 2H, SCH_2), 3.21 (s, 2H, SCH_2), 2.79 (s, 3H, NCH_3), 1.43 (s, 3H, *gem*′′-CH_3), 1.43 (s, 3H, *gem*″-CH_3) 1.42 (s, 6H, *gem*′-CH_3), 1.37 (s, 3H, *gem*-CH_3), 1.23 (s, 3H, *gem*-CH_3);

^{13}C{^{1}H} NMR (CD_2Cl_2, 101 MHz) δ/ ppm: 154.21 (C8a), 149.19 (C3a′′ or C3a′′′), 149.10 (C3a′′ or C3a′′′), 148.12 (C7a′), 139.55 (C7a′′ or C7a′′′), 138.93 (C5′′ or C5′′′), 138.82 (C7a′′ or C7a′′′), 137.99 (3a′), 137.66 (C5′′ or C5′′′), 133.65 (C6), 132.88 (C5′), 130.00 (C4), 128.47 (C7), 126.62 (C6′), 126.19 (C6′′ or C6′′′), 126.09 (C6′′ or C6′′′), 125.40 (C5), 122.83 (C7′′ or C7′′′), 122.77 (C7′′ or C7′′′), 121.50 (C4′′ or C4′′′), 121.43 (C4′′ or C4′′′), 120.71 (C4′), 119.97 (C3), 119.50 (C4a), 115.47 (C8), 107.36 (C7′), 105.30 (C2), 52.28 (C3′), 47.79 (C2′′ or C2′′′), 47.76 (C2′′ or C2′′′), 47.65 (C3′′ or C3′′′), 47.63 (C3′′ or C3′′′), 29.22 (NCH_3), 27.53 (*gem*-CH_3), 27.51 (*gem*-CH_3), 26.11 (*gem*-CH_3), 20.20 (*gem*-CH_3);

FTIR (ATR, neat) ν / cm^{-1}: 2959, 2921, 1642 (C=C), 1613, 1499, 1461, 951 (C_{spiro}-O);

HRMS (TOF AP(+)) *m/z*: $[M+H]^+$ Calcd for $C_{39}H_{40}NOS_2^+$ 602.2546, Found 602.2546.

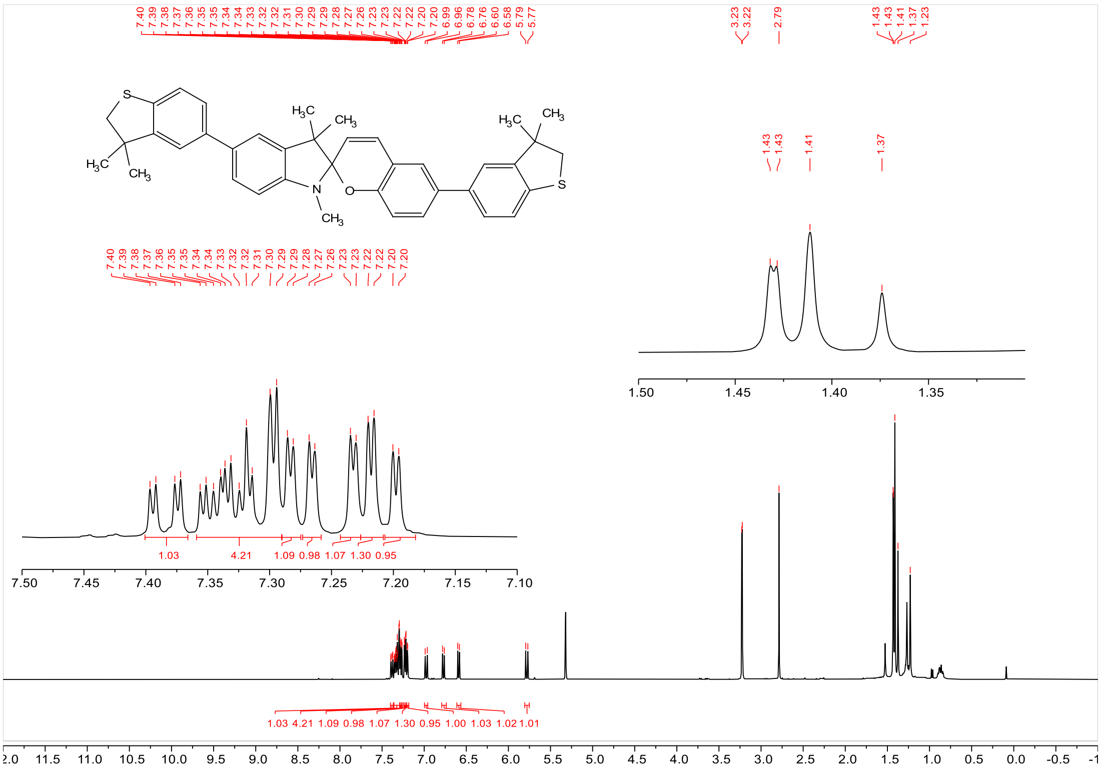

Figure S54 ^{1}H NMR Spectrum of **SP10** in CD_2Cl_2.

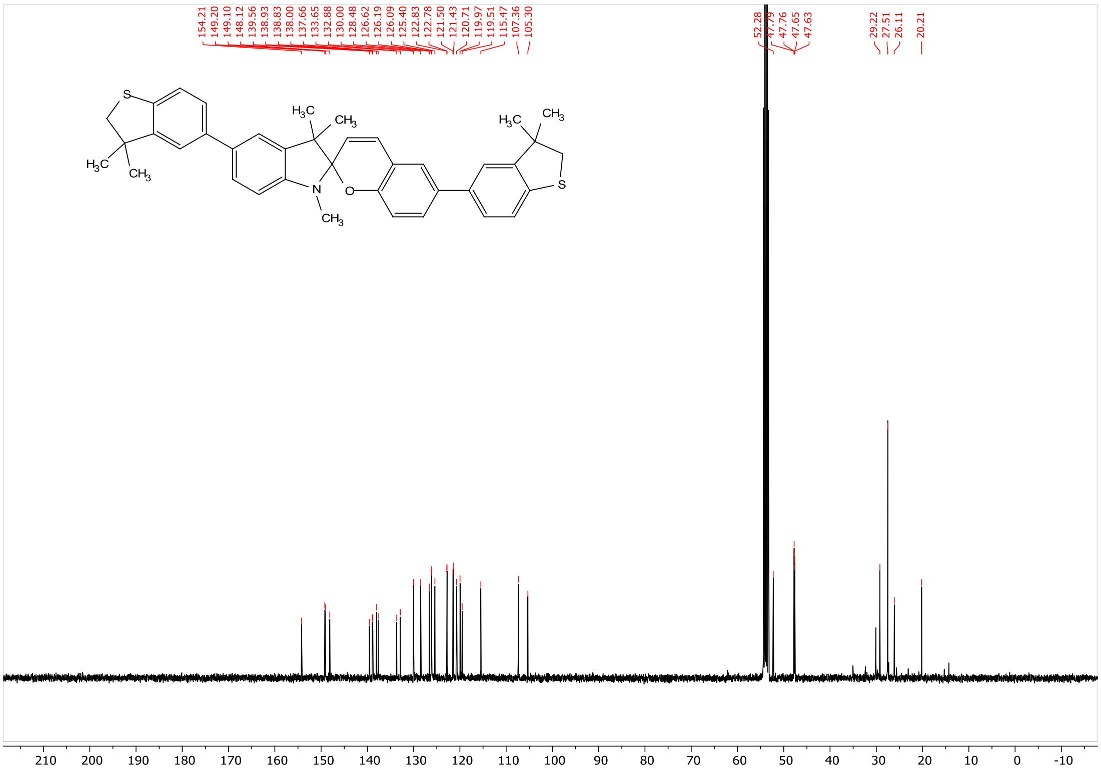

Figure S55 ^{13}C NMR Spectrum of **SP10** in CD_2Cl_2.

5’,7-bis(3,3-dimethyl-2,3-dihydrobenzo[b]thiophen-5-yl)-1',3',3'-trimethylspiro-[chromene-2,2'-indoline] (**SP11**):

A Schlenk tube was charge with **24** (77 mg, 0.17 mmol), **20** (52 mg, 0.18 mmol) and evacuated for 20 min. EtOH (3 mL) and piperidine (0.02 mL) were added. The mixture was refluxed for 90 min under nitrogen. Over this time a precipitate formed in the green-yellow solution. The reaction was cooled to r.t. and then in an ice-bath. The precipitate was collected by vacuum filtration and washed with a little cold ethanol to give a white precipitate **SP11** (67 mg, 67%).

m.p.: 198-200°C;

^{1}H NMR ($CDCl_3$, 400 MHz) δ/ ppm: 7.38 (dd, *J* = 1.9, 8.0 Hz, 1H, H6’), 7.34 (dd, *J* = 1.9, 7.9 Hz, 1H, DMBT-6), 7.32 (dd, *J* = 1.9, 8.1 Hz, 1H, DMBT-6), 7.18 – 7.26 (m, 5H, DMBT-4/4’, DMBT-7/7’,H4’), 7.10 (d, *J* = 7.8 Hz, 1H, H5), 7.06 (dd, *J* = 1.7, 7.8 Hz, 1H, H6), 7.02 (d, *J* = 1.6 Hz, 1H, H8), 6.91 (d, *J* = 10.1 Hz, 1H, H4), 6.59 (d, *J* = 8.5 Hz, 1H, H7’), 5.71 (d, *J* = 10.1 Hz, 1H, H3), 3.22 (s, 2H, SCH_2), 3.19 (s, 2H, SCH_2), 2.80 (s, 3H, NCH_3), 1.43 (s, 6H, DMBT-*gem*-CH_3), 1.40 (s, 3H, *gem*-CH_3), 1.39 (s, 3H, DMBT-*gem*-CH_3), 1.38 (s, 3H, DMBT-*gem*-CH_3), 1.24 (s, 3H, *gem*-CH_3);

^{13}C{^{1}H} NMR ($CDCl_3$, 101 MHz) δ/ ppm: 154.90 (C8a), 148.75 (C3a’’’), 148.55 (C3a’’), 147.76 (C7a’), 142.70 (C7), 140.22 (C7a’’’), 138.90, 138.36 (C7a’’), 137.65 (C3a’), 137.19 (C5’’), 132.81 (C5’), 129.36 (C4), 127.15 (CH), 126.56 (CH), 126.18 (CH), 122.68 (CH), 122.63 (CH), 121.35 (CH), 121.32 (CH), 120.54 (CH), 119.03 (C3), 118.58 (CH), 117. 76 (C4a), 113.25 (CH), 107.13 (CH), 104.73 (C2), 52.01 (C3’), 47.63 (C2’’), 47.58 (C2’’’), 47.45 (C3’’), 47.40 (C3’’’), 29.23 (NCH_3), 27.59 (DMBT-*gem*-CH_3), 27.56 (DMBT-*gem*-CH_3), 26.14 (*gem*-CH_3), 20.39 (*gem*-CH_3);

FTIR (ATR, neat) ν / cm-1: 2957, 2927, 1634 (C=C), 1609, 1592, 1546, 1494, 1460, 964 (C_{spiro}-O);

HRMS (TOF AP(+)) *m/z*: $[M+H]^+$ Calcd for $C_{39}H_{40}NOS_2^+$ 602.2546, Found 602.2559.

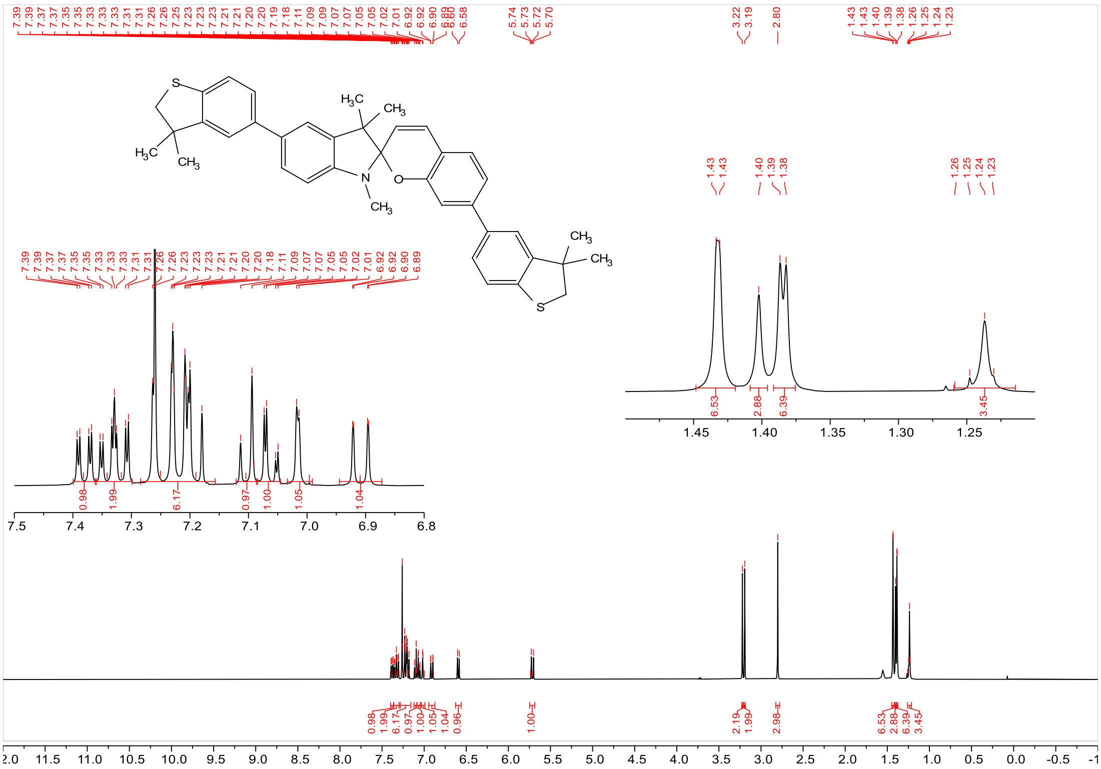

Figure S56 ^{1}H NMR Spectrum of **SP11** in $CDCl_3$.

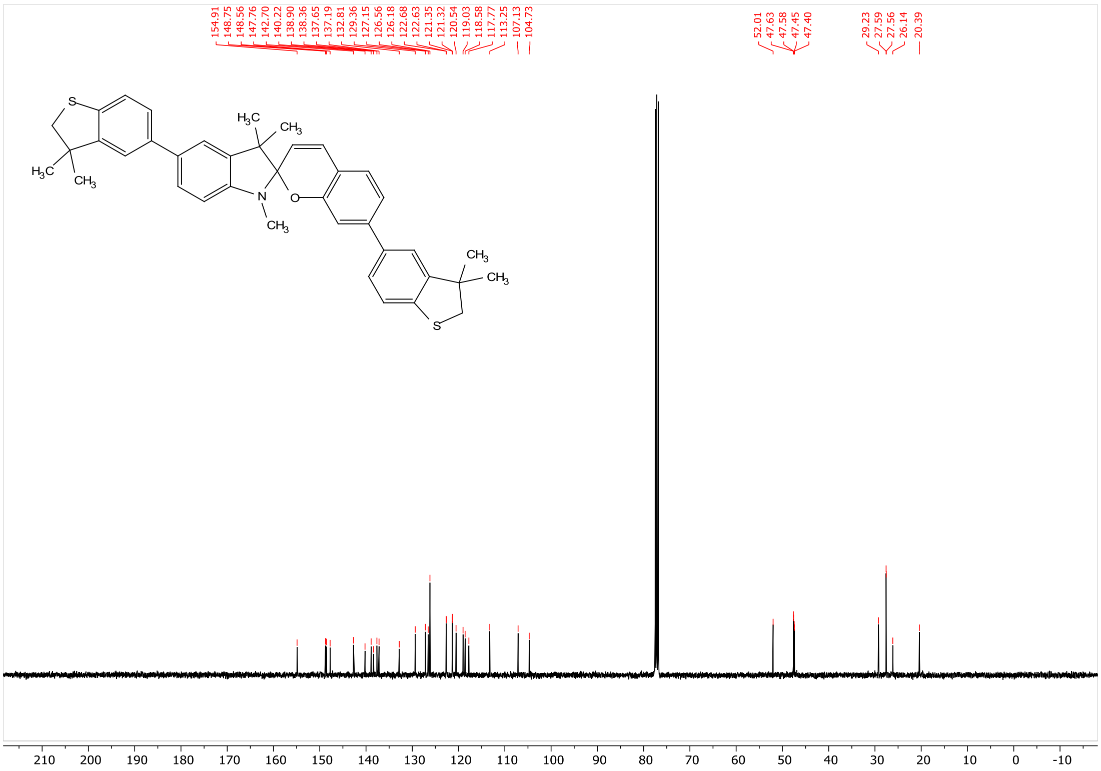

Figure S57 ^{13}C NMR Spectrum of **SP11** in $CDCl_3$.

1,4-dibromo-2-nitrobenzene (**27**):

Prepared by modified literature procedure.[14c] Reaction conducted under standard atmospheric conditions. A mixture of nitric acid (3.8 mL, 59 mmol) in sulfuric acid (5.6 mL) was added dropwise to a solution of 1,4-dibromobenzene **26** (10 g, 43 mmol) in CH_2Cl_2 (27 mL) and sulfuric acid (18 mL). The pale-yellow solution became blue upon addition of the acid mixture before becoming a deep canary yellow. The reaction was then quenched with NaOH (25 % w/v, 30 mL) and the organic phase extracted with CH_2Cl_2 (3 × 30 mL). The combined organic extracts were then dried over Na_2SO_4, filtered, and the solvent was subsequently removed by rotary evaporation before drying under high vacuum, affording a yellow solid. The crude product was purified via recrystallisation from hot hexanes, furnishing pale yellow crystals (9.95 g, 35 mmol, 84 %). Spectroscopic data are consistent with the literature.[23]

m.p.: 83-84 °C [Lit.[23] 83-84 °C]

^{1}H NMR ($CDCl_3$, 400 MHz) δ / ppm: = 7.98 (d, *J* = 2.2 Hz, 1H, H6), 7.61 (d, *J* = 8.5 Hz, 1H, H3), 7.56 (dd, *J* = 8.5, 2.2 Hz, 1H, H4).

^{13}C{^{1}H} NMR ($CDCl_3$, 101 MHz) δ / ppm: 150.3 (C2), 136.4 (C4), 136.3 (C3), 128.7 (C6), 121.6 (C1), 113.4 (C5).

FTIR (ATR, neat) ν / cm^{-1}: 3081 (C-H), 1524 (N=O), 1347 (N=O), 1254 (C-N).

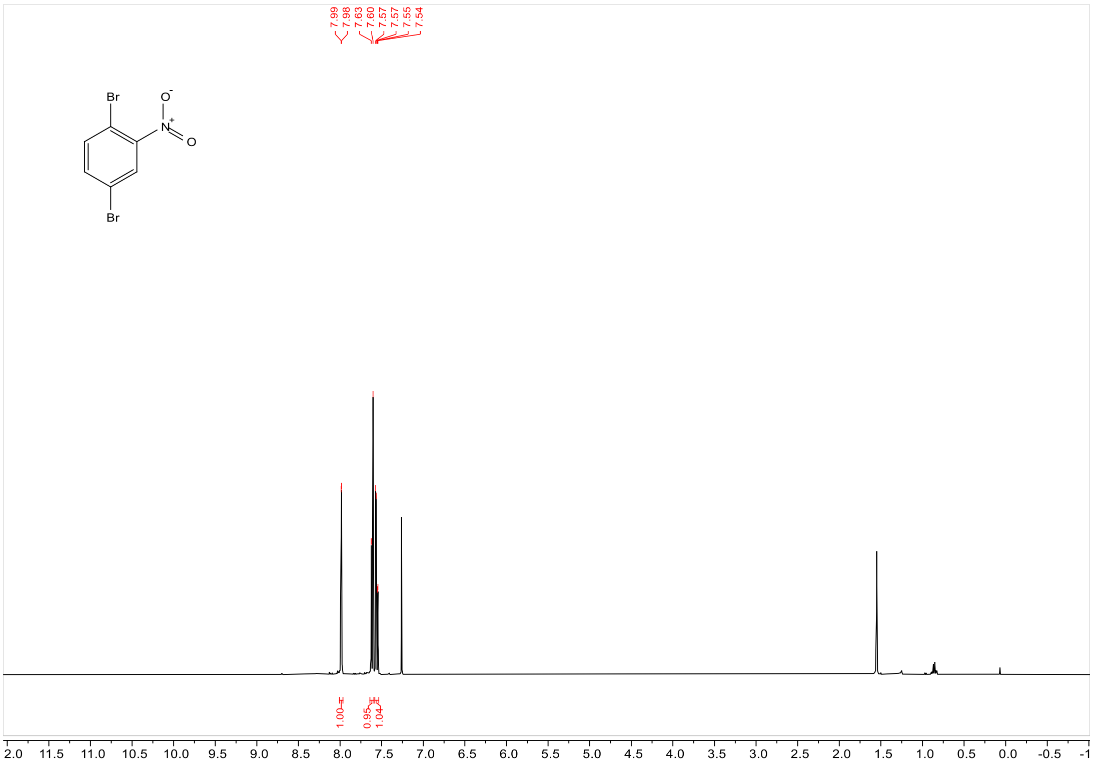

Figure S58 ^{1}H NMR spectrum of **27** in $CDCl_3$.

2,5-dibromobenzenamine (**28**):

Prepared by a literature procedure.[24] A solution of **27** (6.80 g, 24 mmol) in THF (30 mL) and ethanol (30 mL) was sparged (Ar) for 30 min, before the portion-wise addition of stannous chloride dihydrate (22.43 g, 99 mmol). Upon addition of the stannous chloride, the yellow solution warmed and deepened to orange and intensified in colour. The solution was stirred for 4 h before quenching with NaOH (15 %, 250 mL), with evolution of a pale orange precipitate. The organic material was extracted with ethyl acetate (100 mL), washed with water (2 × 50 mL) and brine (50 mL), dried over Na_2SO_4, filtered through cotton wool, and the solvent was removed in vacuo before drying under high vacuum to yield the product as bright orange crystals (5.25 g, 20.9 mmol, 85 %)

m.p.: 53-54 °C [Lit.[25] 54-55 °C]

^{1}H NMR ($CDCl_3$, 400 MHz) δ/ ppm: 7.24 (d, *J* = 8.4 Hz, 1H, H3), 6.90 (d, *J* = 2.4 Hz, 1H, H6), 6.73 (dd, *J* = 8.4, 2.4 Hz, 1H, H4), 4.16 (br s, 2H, NH_2).

^{13}C{^{1}H} NMR ($CDCl_3$, 101 MHz) δ/ ppm: 145.4 (C2), 133.8 (C3), 122.3 (C4), 121.9 (C5), 118.3 (C6), 107.9 (C1).

FTIR (ATR, neat) ν / cm^{-1}: 3419 (N-H), 3317 (N-H), 3222 (C-H), 1257 (C-N), 1619 (N-H).

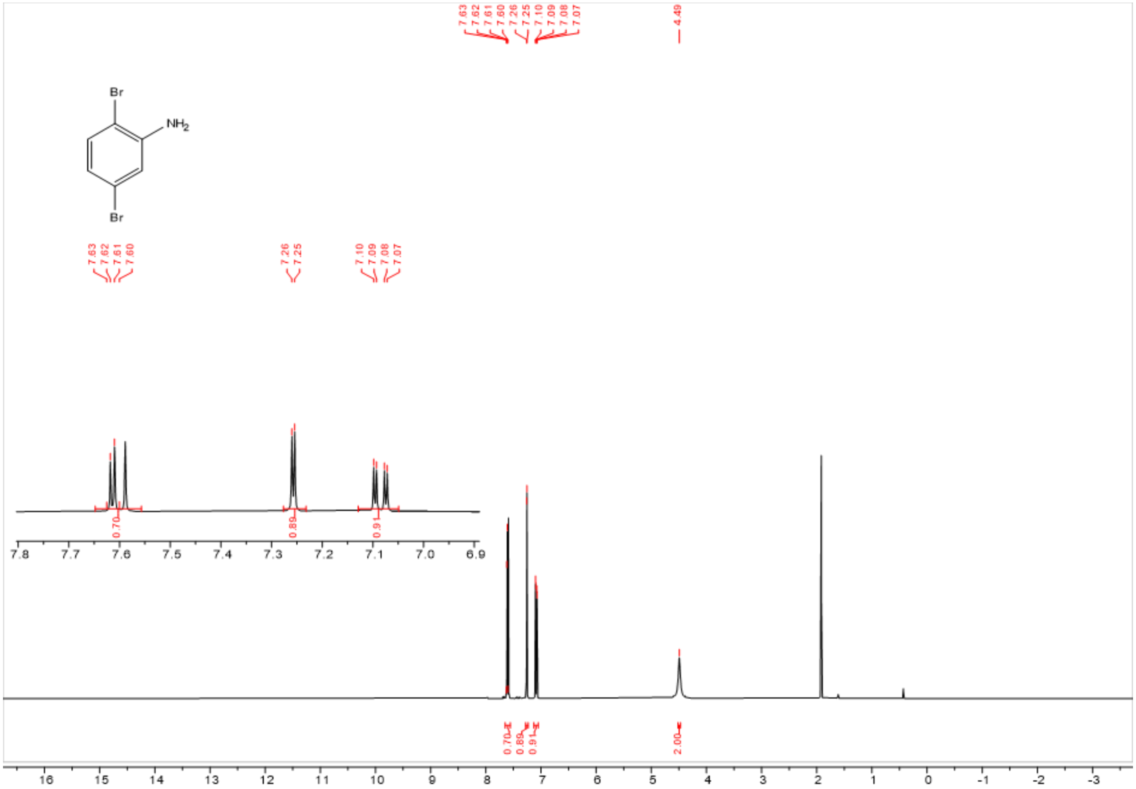

Figure S59 ^{1}H NMR spectrum of **28** in $CDCl_3$.

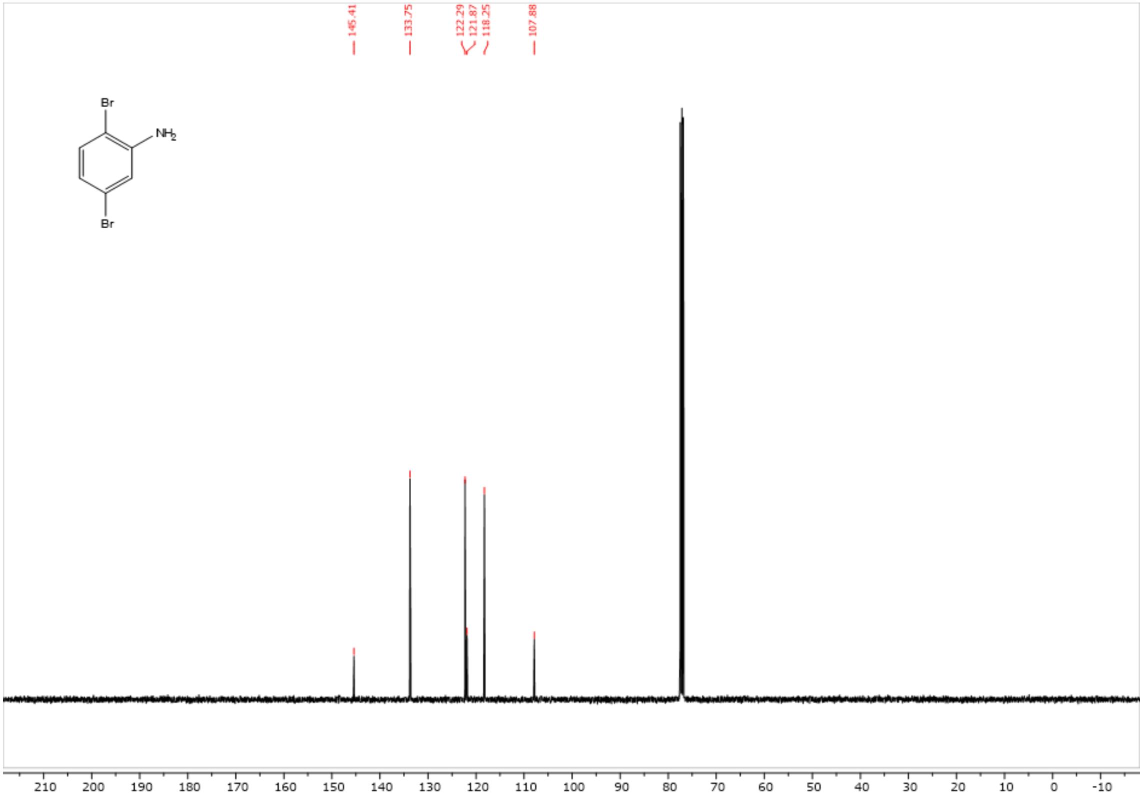

Figure S60 ^{13}C NMR spectrum of **28** in $CDCl_3$.

(2,5-dibromophenyl)hydrazine hydrochloride (**29**):

Prepared by modified literature procedure.[14b] Reaction conducted under standard atmospheric conditions and the reaction is and kept below 5 ºC. To a suspension of **28** (4.98 g, 19.8 mmol) in HCl (conc., 100 mL), sodium nitrite (1.55 g, 22.6 mmol) in water (20 mL) was added dropwise, and the solution became yellow in colour. After stirring for 90 min, urea (302 mg, 4.98 mmol) was added and the reaction mixture stirred for an additional 15 min. Stannous chloride dihydrate (9.00 g, 39.9 mmol) in HCl (conc., 30 mL) was then added dropwise, yielding a pale orange precipitate. After stirring for 1.5 h, the precipitate was collected by vacuum filtration, washing successively with ethyl acetate, diethyl ether and pentane, affording a pale orange solid of **21**. (5.17 g, 17.1 mmol, 86 %).

m.p.: 187°C [decomposition].

^{1}H NMR (DMSO-d$_6$, 400 MHz) δ/ ppm: 10.37 (br s, 3H, NH_3), 8.18 (br s, 1H, NH), 7.52 (d, *J* = 8.4 Hz, 1H, H3), 7.28 (d, *J* = 2.5 Hz, 1H, H6), 7.10 (dd, *J* = 8.4, 2.5 Hz, 1H, H4).

^{13}C{^{1}H} NMR (DMSO-d$_6$, 101 MHz) δ/ ppm: 143.9 (C2), 134.4 (C3), 125.2 (C4), 121.1 (C5), 117.2 (C6), 108.1 (C1).

FTIR (ATR, neat) ν / cm^{-1}: 3368 (N-H), 3221 (N-H), 3095 (C-H).

HRMS (TOF AP(+)) *m/z*: 249.8873 [M-NH]$^{+}$.

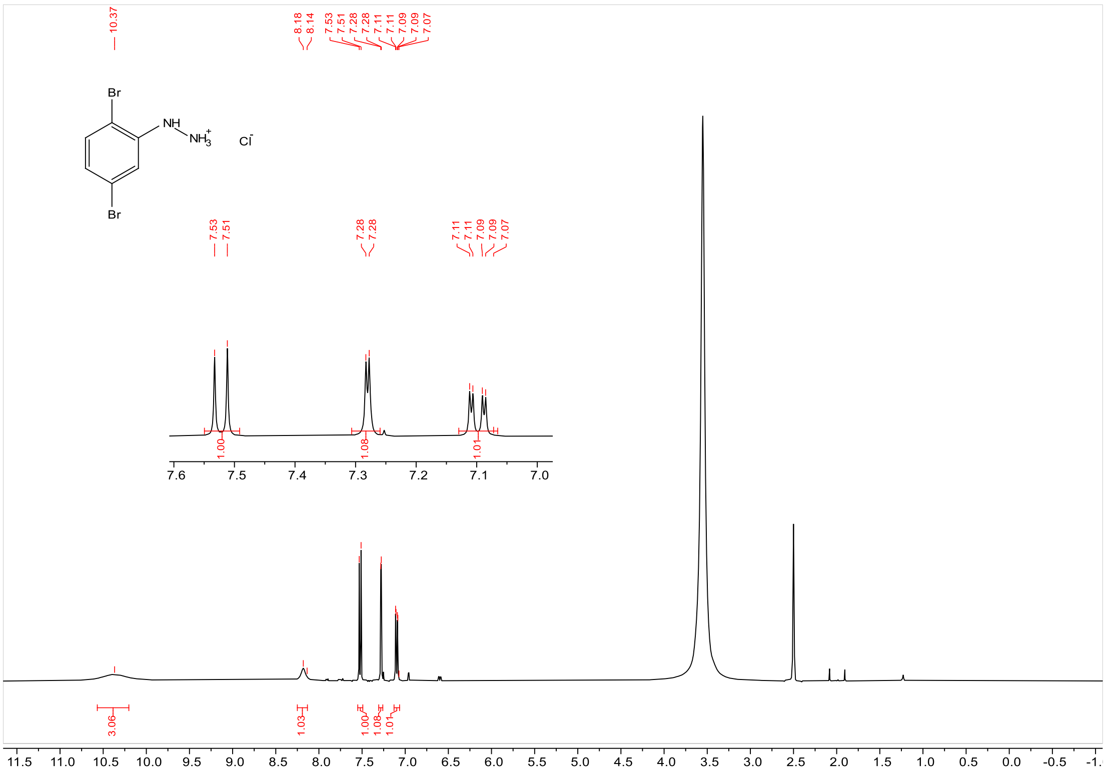

Figure S61 ^{1}H NMR spectrum of **29** in DMSO-d_6.

4,7-dibromo-2,3,3-trimethyl-3*H*-indole (**30**):

Synthesised according to literature procedure.[14b] 3-Methyl-2-butanone (3.6 mL, 33.1 mmol) was added to a solution of **29** (4.91 g, 16.3 mmol) in acetic acid (250 mL). The orange solution was refluxed for 24 h, becoming crimson. The reaction was cooled and most of the acetic acid was removed by rotary evaporation and then quenched with sodium bicarbonate (saturated aqueous solution) to yield a brown-orange precipitate. The organic material was extracted with ethyl acetate (100 mL), washed with water (3 × 100 mL) and brine (50 mL), dried over Na_2SO_4, filtered through cotton wool and the solvent was removed *in vacuo* before drying under high vacuum. The resultant crude orange solid was then purified by flash chromatography (gradient eluent of hexanes to 20% ethyl acetate in hexanes) to yield orange crystals (1.93 g, 6.1 mmol, 38 %): Spectroscopic data consistent with literature data.[14b]

m.p.: 131-133 °C [lit.[14b] = 132 °C]

^{1}H NMR ($CDCl_3$, 400 MHz) δ/ ppm: 7.33 (d, *J* = 8.5 Hz, 1H, H6), 7.18 (d, *J* = 8.5 Hz, 1H, H5), 2.33 (s, 3H, CH_3), 1.46 (s, 6H, *gem*-CH_3)

$^{13}C\{^1H\}$ NMR ($CDCl_3$, 101 MHz) δ/ ppm: 191.0 (C2), 153.8 (C7a), 144.2 (C3a), 132.6 (C6), 130.5 (C5), 116.6 (C4), 113.3 (C7), 58.4 (C3), 19.7 (*gem*-CH_3), 15.8 (CH_3).

FTIR (ATR, neat) ν / cm^{-1}: 3280, 2968, 2928, 1664.

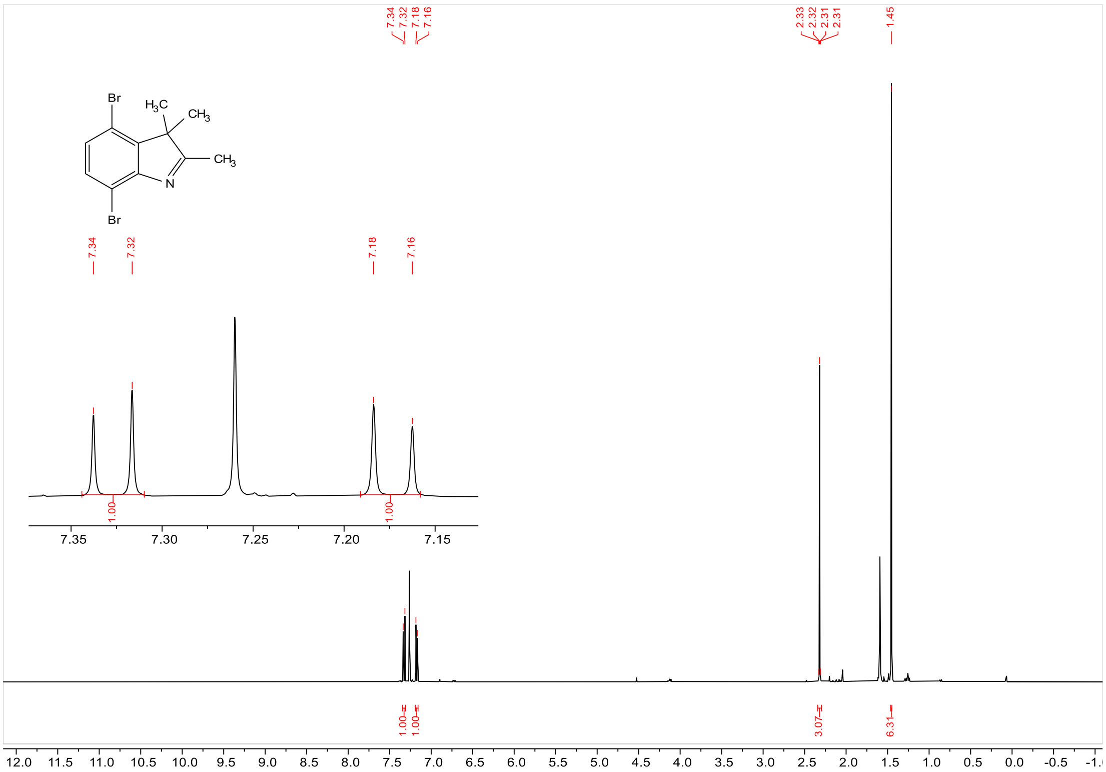

Figure S62 ^1H NMR spectrum of **30** in $CDCl_3$**.**

4,7-dibromo-1,2,3,3-tetramethyl-3*H*-indol-1-ium tetrafluoroborate (**31**):

30 (589 mg) and $(CH_3)_3O(BF_4)$ (295 mg) were dissolved in dry CH_2Cl_2 (10 mL) and stirred at room temperature for 24 h. Over this time the solution goes from orange to red. CH_3OH (1 mL) is added and the solution is stirred for another 1 h. After removal of the volatiles under reduced pressure the residue is triturated with Et_2O, and the resulting pink precipitate is collected by vacuum filtration and washed with further aliquots of Et_2O until the washings are colourless. Recrystallisation from CH_3CN and Et_2O gives thin pale pink needles (672 mg, 86%).

m.p.: 262 °C (decomposed)

^1H NMR (CD_3CN, 500 MHz) δ/ ppm: 8.27 (d, *J* = 8.6 Hz, 1H, H6), 8.20 (d, *J* = 8.6 Hz, 1H, H5), 4.75 (s, 3H, NCH_3), 3.27 (s, 3H, $NCCH_3$), 2.25 (s, 6H, *gem*-CH_3).

$^{13}C\{^1H\}$ **NMR** (CD_3CN, 101 MHz) δ/ ppm: 200.48 (C2), 142.59 (C7a), 141.88 (C3a), 137.71 (C6), 135.94 (C5), 118.59 (C7), 108.58 (C4), 57.07 (C3), 40.33 (NCH_3), 18.75 (*gem*-CH_3), 15.78 ($NCCH_3$).

^{19}F **NMR** (CD_3CN, 470 MHz) δ/ ppm: -146.54 (s).

^{11}B **NMR** (CD_3CN, 160 MHz) δ/ ppm: 4.10 (s).

FTIR (ATR, neat) ν / cm^{-1}: 1613 (C=N), 1452, 1369, 1179, 1151, 1094, 1030 (BF_4), 989, 939.

HRMS (TOF AP(+)) *m/z*: $[M-BF_4]^+$ Calcd for $C_{12}H_{14}Br_2N^+$ 331.9468, Found 331.9466.

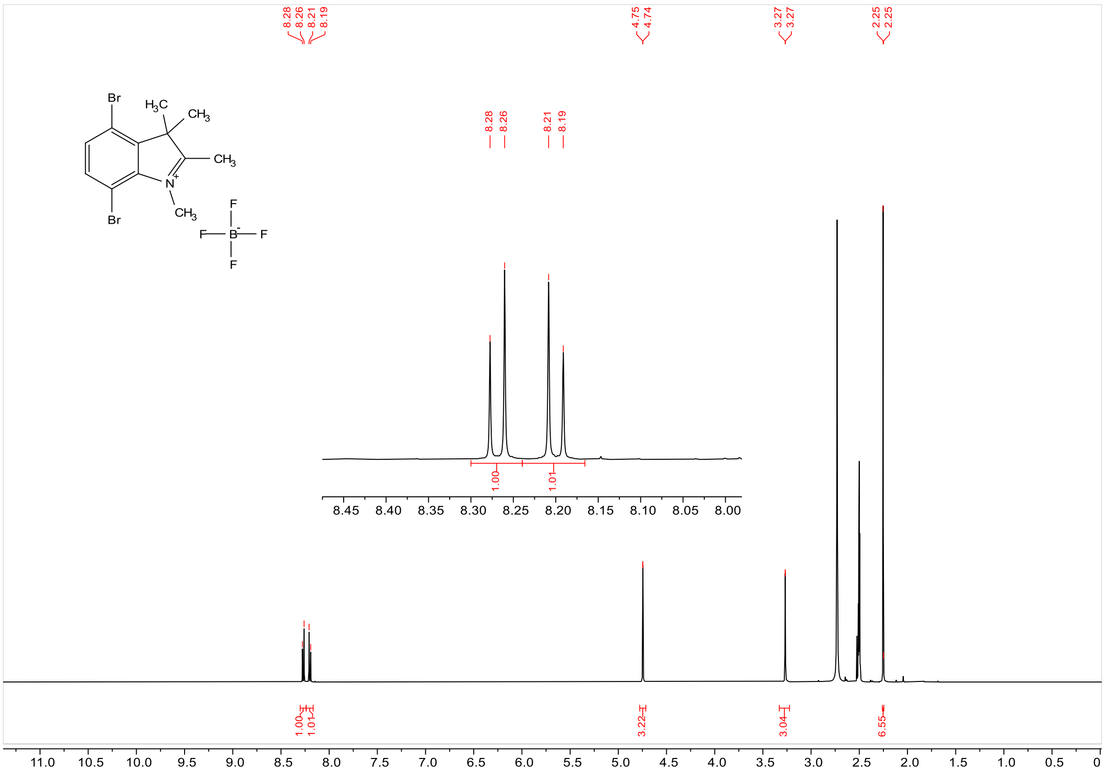

Figure S63 ^1H NMR spectrum of **31** in CD_3CN.

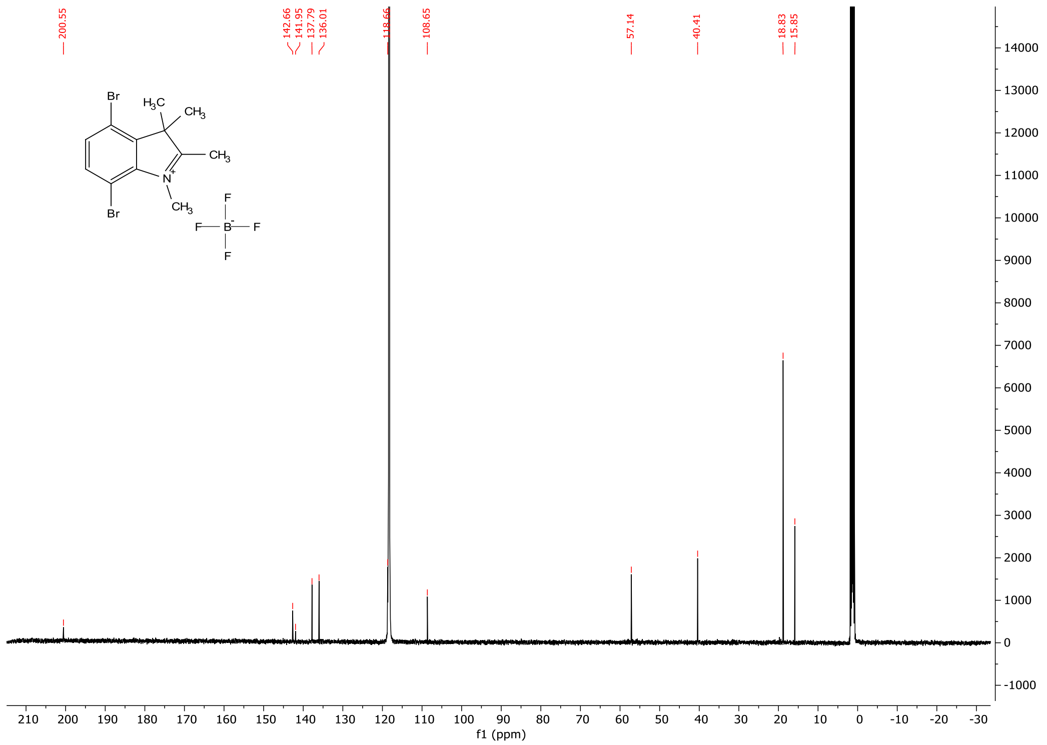

Figure S64 ^{13}C NMR spectrum of **31** in CD_3CN.

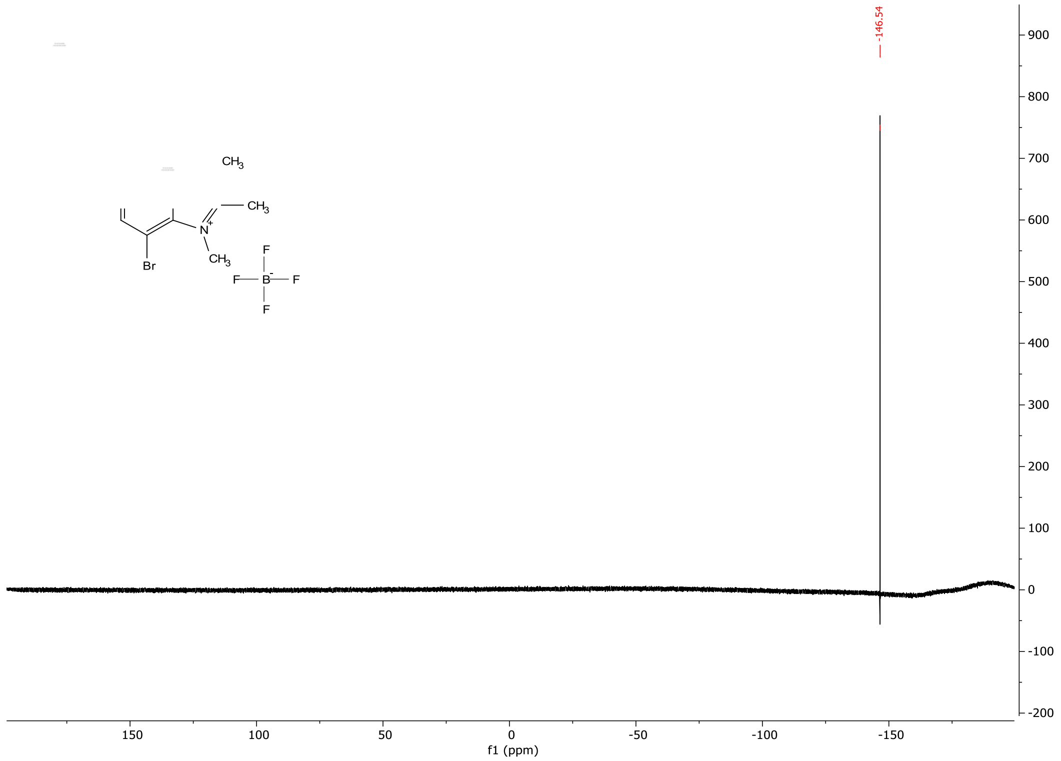

Figure S65 ^{19}F NMR spectrum of **31** in CD_3CN.

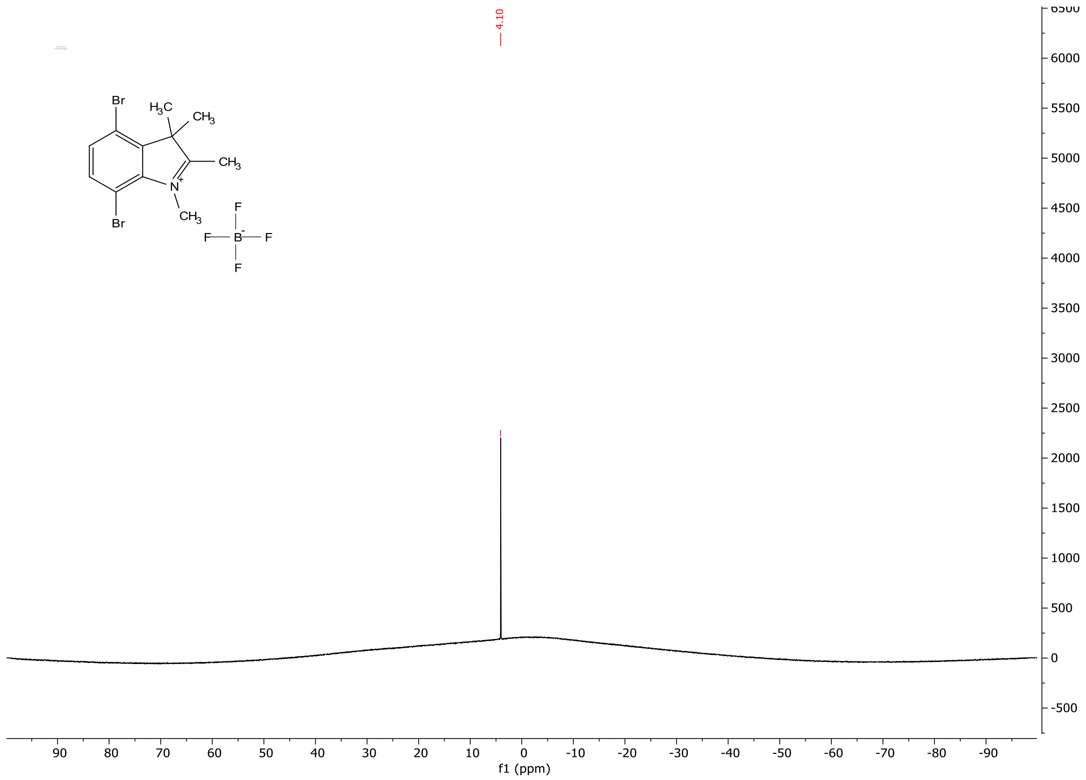

Figure S66 ^{11}B NMR spectrum of **31** in CD_3CN.

4',7'-dibromo-1',3',3'-trimethylspiro[chromene-2,2'-indoline] (**33**):

A solution of **31** (150 mg, 0.33 mmol) and 2-hydroxy-5-nitrobenzaldehyde **32** (61 mg, 0.36 mmol) in ethanol (3 mL) was prepared and the solution sparged for 20 min with argon. Piperidine (0.050 mL, 0.50 mmol) was then added, and the resultant yellow-orange solution refluxed for 3 h. The deep orange solution was then cooled to room temperature and then in an ice bath. The resulting yellow precipitate was collected by vacuum filtration and washed with a little cold ethanol and pentane (113 mg, 72%). Spectral data is consistent with the literature.[14a]

m.p.: 184-186°C

^{1}H NMR ($CDCl_3$, 500 MHz) δ/ ppm: 8.05 (dd, *J* = 9.0, 2.7 Hz, 1H, H7), 8.01 (d, *J* = 2.7 Hz, 1H, H5), 7.15 (d, *J* = 8.6 Hz, 1H, H6′), 6.97 (d, J = 10.4 Hz, H4), 6.86-6.79 (m, 2H, H5′ & H8), 5.78 (d, *J* = 10.4 Hz, 1H, H3), 3.16 (s, 3H, NCH_3), 1.46 (s, 3H, *gem*-CH_3), 1.23 (s, 3H, *gem*-CH_3) ppm.

^{13}C{^{1}H} NMR ($CDCl_3$, 126 MHz) δ/ ppm: 159.60 (C6), 146.43 (C7a′), 141.33 (C8a), 135.88 (C3a′), 135.01 (C6′), 129.52 (C4), 126.29 (C7), 126.02 (C5′), 123.05 (C8), 120.85 (C3), 118.19 (C4a),

117.64 (C4′), 115.54 (C5), 107.25 (C2), 100.84 (C7′), 53.98 (C3′), 32.67 (NCH3), 22.26 (*gem*-CH_3), 20.34 (*gem*-CH_3).

FTIR (ATR, neat) ν / cm^{-1}: 3078, 2999, 2930, 2851, 1653 (C=C) 1255, 954 (C-O).

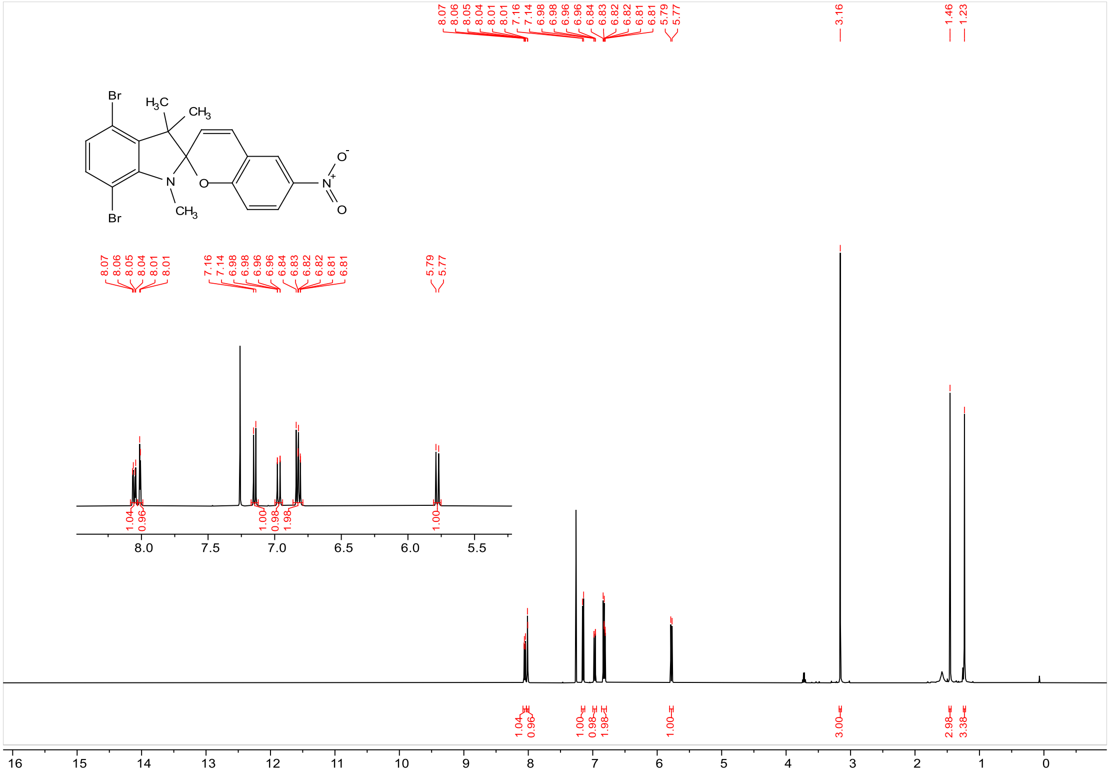

Figure S67 ^1H NMR spectrum of **33** in $CDCl_3$.

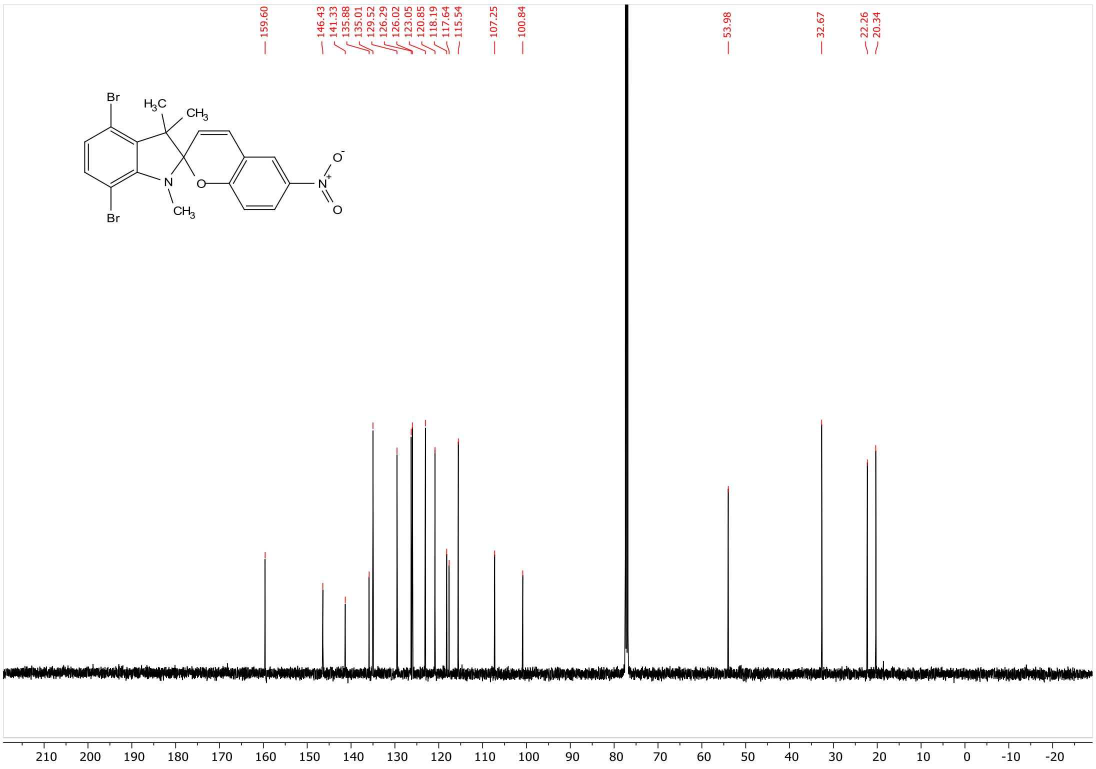

Figure S68 ^{13}C NMR spectrum of **33** in $CDCl_3$.

4',7'-dipyridiyl-1',3',3'-trimethylspiro[chromene-2,2'-indoline] (**SP12**):

A mixture of 1,4-dioxane (3 mL) and water (0.6 mL) was subject to 3 cycles of freezer-pump-thaw. To the vessel **33** (136 mg, 0.283 mmol), 4-pyridinylboronic acid (88 mg, 0.72 mmol), K_2CO_3 (896 mg, 22.9 mmol) and Pd(dppf)Cl_2.CH_2Cl_2 (28 mg; 0.12 mmol) were charged. The solution refluxed for 3 days. Upon heating, the solution deepened to a dark orange colour. The solvent was then removed, and the crude product extracted into CH_2Cl_2 (20 mL). The organic extract was then washed with water (2 x 20 mL) and brine (20 mL), dried over Na_2SO_4, filtered, and the solvent removed. Purification of the crude red residue by flash chromatography (alumina, CH_2Cl_2) and PTLC (silica, 5% triethylamine, 45% ethyl acetate, 50% hexanes), yielded a pale-yellow powder **SP12** (7 mg, 5%). The ^{1}H NMR is consistent with the literature.[14a]

^{1}H NMR ($CDCl_3$, 400 MHz) δ/ ppm: 8.64 (m, 4H, py), 8.00 (dd, *J* = 2.2, 8.6 Hz, 1H), 7.94 (d, *J* = 2.7 Hz, 1H) 7.37 (m, 2H, py), 7.31 (m, 2H, py), 7.07 (d, *J* = 7.8Hz, 1H), 6.90 (d, *J* = 10.5 Hz, 1H), 6.79 (d, *J* = 9.1 Hz, 1H), 6.68 (d, *J* = 7.9 Hz, 1H), 5.80 (d, *J* = 10.4 Hz, 1H), 2.37 (s, 3H), 1.25 (s, 3H), 0.79 (s, 3H).

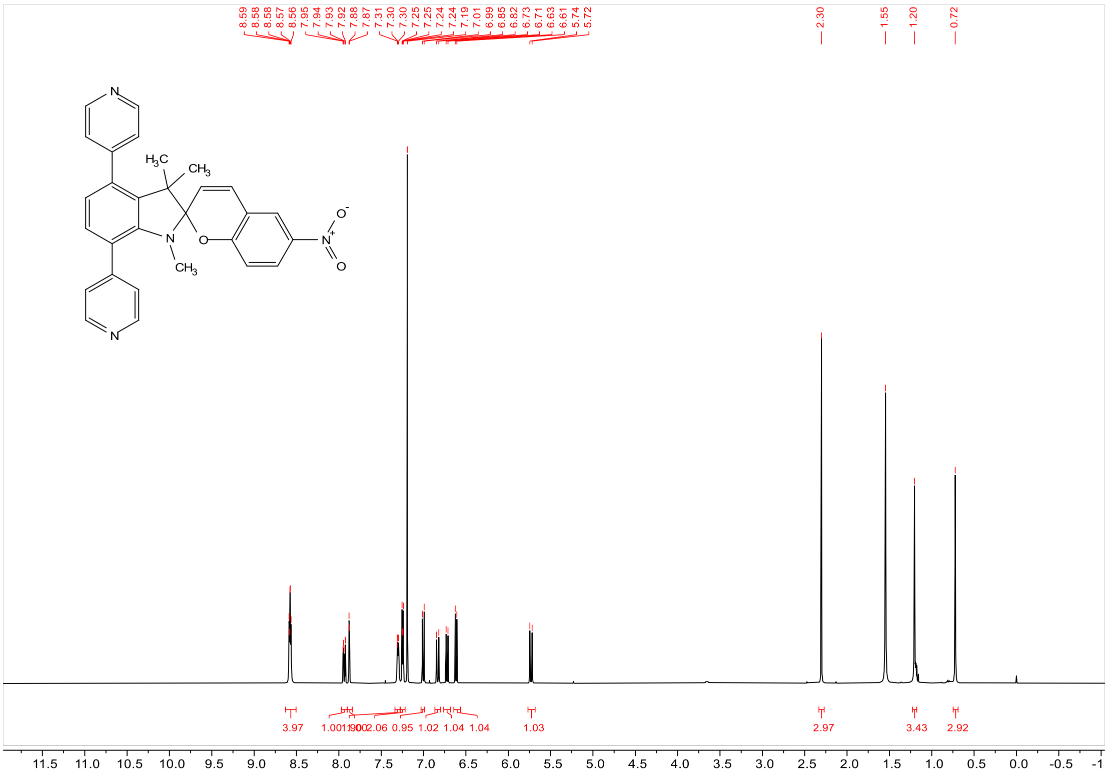

Figure S69 ^{1}H NMR spectrum of **SP12** in $CDCl_3$.

4,7-bis(3,3-dimethyl-2,3-dihydrobenzo[*b*]thiophen-5-yl)-2,3,3-trimethyl-3*H*-indole (**34**):

A solution of **30** (204 mg; 0.64 mmol), **15** (337 mg; 1.6 mmol) and sodium carbonate (426 mg; 4.0 mmol) in dimethoxyethane (20 mL) and DI water (5 mL) was sparged for 1 h. $Pd(dppf)Cl_2.CH_2Cl_2$ (55 mg; 0.07 mmol, 10 mol %) was then added, and the orange solution refluxed for 24 h. The solution becomes dark yellow in colour. The solvent was removed, and the organic material extracted with ethyl acetate (20 mL) and washed with DI water (20 mL) and brine (20 mL), before being dried (Na_2SO_4), filtered through cotton wool and the solvent removed to yield a dark yellow/green oil. The crude product was passed through a silica plug (10 % ethyl acetate in hexanes) to yield fluffy orange crystals (226 mg, 73 %).

m.p.: 70-72°C

^{1}H NMR ($CDCl_3$, 400 MHz) δ/ ppm: 7.62 (dd, *J* = 8.1, 1.7 Hz, 1H, H6′), 7.46 (d, *J* = 1.7 Hz, 1H, H4′), 7.39 (d, *J* = 7.8 Hz, 1H, H6), 7.28 (d, *J* = 8.1 Hz, 1H, H7′), 7.23 (d, *J* = 7.8, 1H, H7′′), 7.07 (dd, *J* = 7.8, 1.7 Hz, 1H, H6′′), 7.04 (d, *J* = 7.84, 1H, H5), 6.99 (d, *J* = 1.7 Hz, H4′′), 3.26 (s, 2H, H2′′), 3.22 (s, 2H, H2′), 2.22 (s, 3H, $NCCH_3$), 1.44 (s, 6H, 3′-*gem*-CH_3), 1.39 (s, 6H, 3′′-*gem*-CH_3), 1.13 (s, 6H, *gem*-CH_3).

$^{13}C\{^{1}H\}$ NMR ($CDCl_3$, 101 MHz) δ/ ppm: 207.1 (C2), 148.0 (C3a′), 147.6 (C3a′′), 142.9 (C3a), 139.8 (C7a′), 139.6 (C7a′′), 137.7 (C7a), 136.2 (C5′′), 135.1 (C5′), 132.5 (C4 & C7), 129.3 (C6′), 128.8 (C6′′), 127.6 (C5 & C6), 124.5 (C4′′), 124.1 (C4′), 122.3 (C7′), 121.6 (C7′′), 54.8 (C3), 47.6 (C2′′), 47.4 (C2′), 31.1 (C3′ & C3′′), 27.6 (C3′-*gem*-CH_3), 27.6 (C3′′-*gem*-CH_3), 22.8 (*gem*-CH_3), 15.5 ($NCCH_3$).

FTIR (ATR, neat) ν / cm^{-1}: 2959, 2923, 2864, 1594 (C=N).

HRMS (TOF ES(+)) *m/z*: $[M+H]^+$ Calcd for $C_{31}H_{34}NS_2^+$ 484.2128, Found 484.2125.

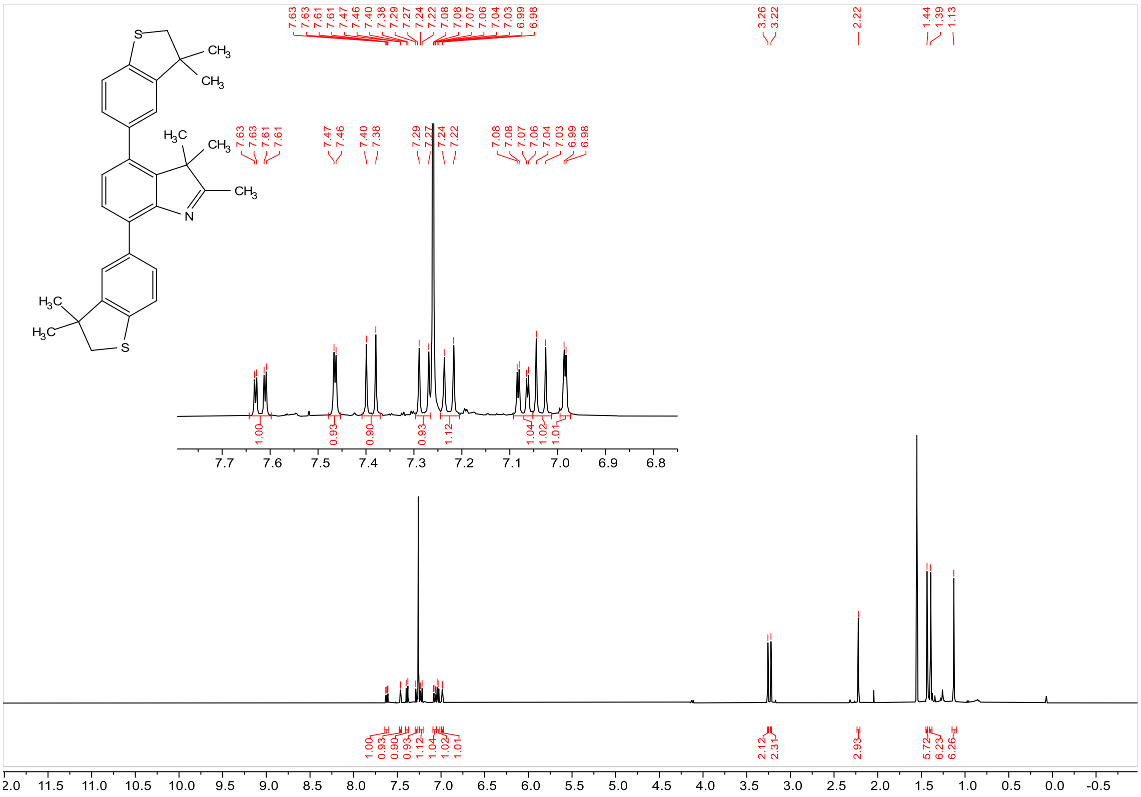

Figure S70 ^{1}H NMR spectrum of **34** in $CDCl_3$.

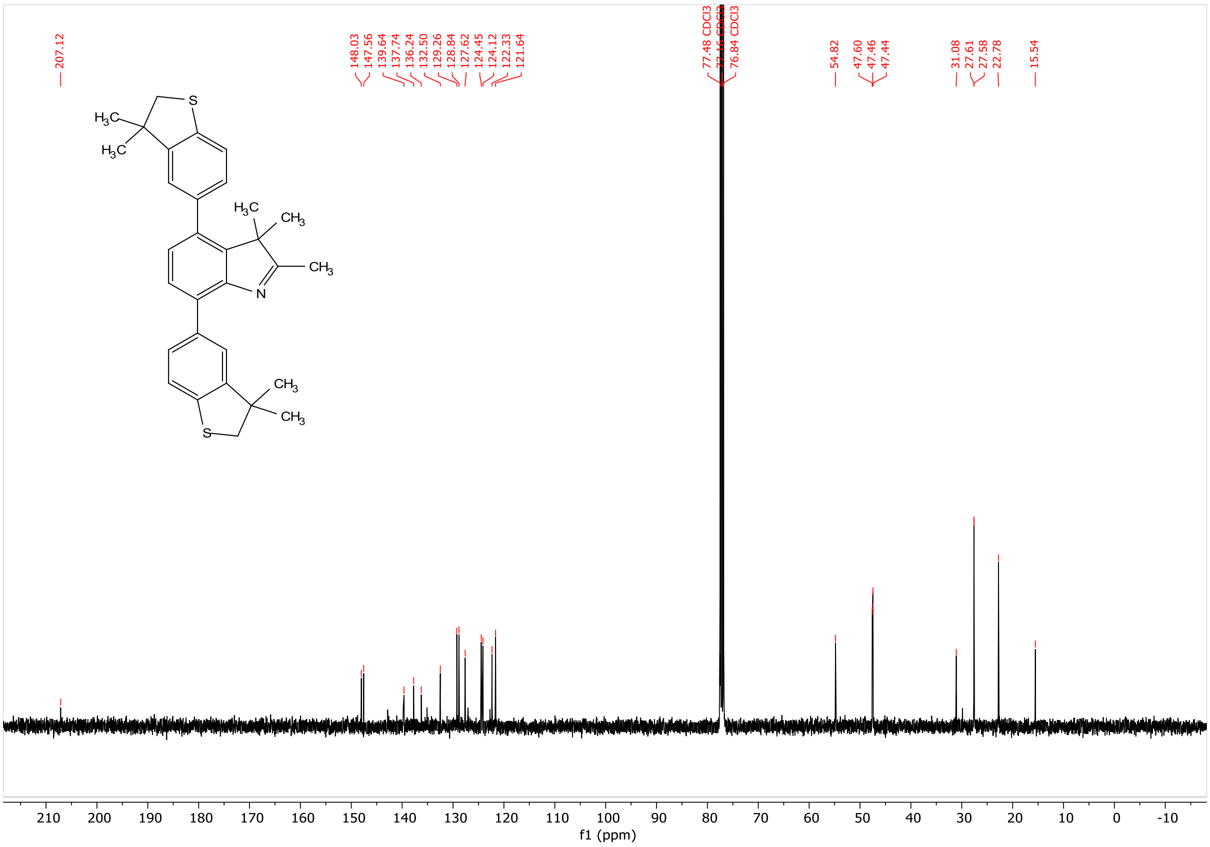

Figure S71 ^{13}C NMR Spectrum of **34** in $CDCl_3$.

2,3,3-trimethyl-4,7-bis(4-(methylthio)phenyl)-3*H*-indole (**35**):

A solution of **30** (200 mg; 0.63 mmol), **16** (266 mg; 1.6 mmol) and sodium carbonate (420 mg; 4.0 mmol) in dimethoxyethane (20 mL) and water (5 mL) was sparged for 25 min with argon. $Pd(dppf)Cl_2.CH_2Cl_2$ (54 mg; 0.07 mmol, 10 mol %) was then added, and the orange solution refluxed for 24 h. The solvent was then removed, and the organic material extracted into ethyl acetate (20 mL) and washed with DI water (20 mL) and brine (20 mL). The extract was then dried (Na_2SO_4), filtered through cotton wool and the solvent removed, yielding a crude yellow oil. The crude product was purified by flash chromatography (20 % ethyl acetate in hexanes), affording a yellowy-white crystalline solid (125 mg, 49 %):

m.p.: 145-147°C

^{1}H NMR ($CDCl_3$, 400 MHz) δ/ ppm: 7.77 (m, 2H, H3′), 7.40 (d, *J* = 7.9 Hz, 1H, H6), 7.38 (m, 2H, H2′), 7.32 (m, 2H, H3′′), 7.25 (m, 2H, H2′′), 7.02 (d, *J* = 7.9 Hz, 1H, H5), 2.56 (s, 3H, S′-CH_3), 2.53 (s, 3H, S′′-CH_3), 2.24 (s, 3H, $NCCH_3$), 1.15 (s, 6H, *gem*-CH_3).

$^{13}C\{^{1}H\}$ NMR ($CDCl_3$, 101 MHz) δ/ ppm: 189.2 (C2*), 151.2 (C7a†), 142.9 (C3a), 137.8, 137.5, 137.4 (C4), 136.7, 135.6, 130.2, 130.1, 127.7 (C5), 127.6 (C6), 126.9, 125.7, 54.9 (C3), 22.8 (*gem*-CH_3), 16.2 (S″-CH_3), 15.8 (S′-CH_3), 15.5 ($NCCH_3$).

FTIR (ATR, neat) ν / cm^{-1}: 2959, 2923, 2865, 1594 (C=N), 1455.

HRMS (TOF ES(+)) *m/z*: $[M+H]^+$ Calcd for $C_{25}H_{26}NS_2^+$ 404.1502, Found 404.1517.

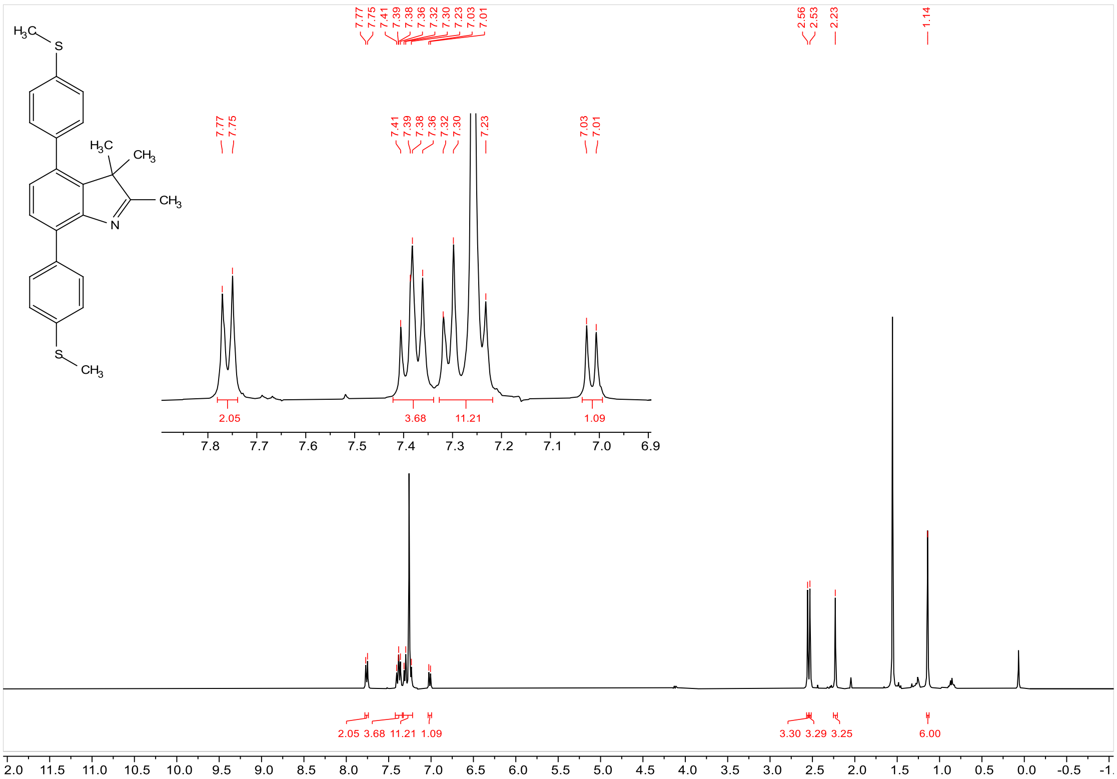

Figure S72 ^{1}H NMR spectrum of **35** in $CDCl_3$.

---

* Observed using 2D NMR

† Observed using 2D NMR

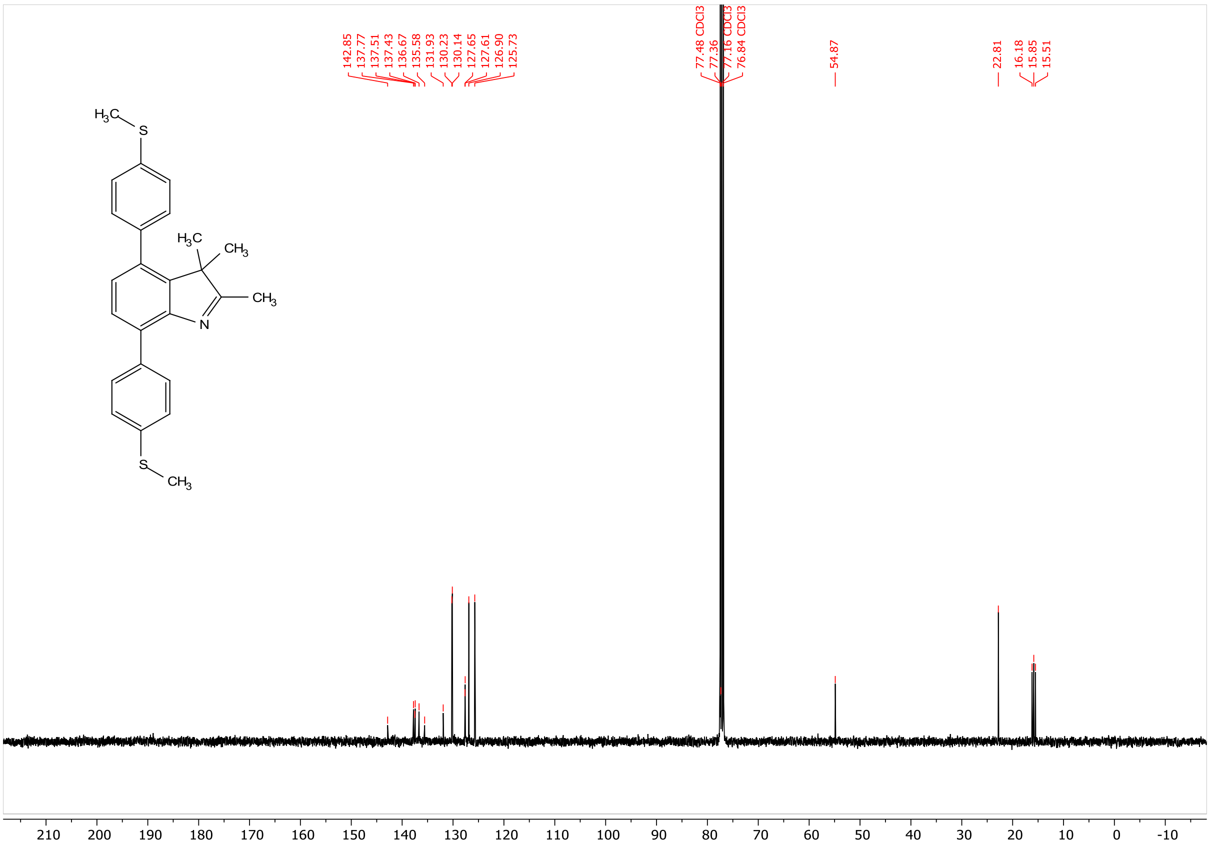

Figure S73 ^{13}C NMR spectrum of **35** in $CDCl_3$.

4,7-bis(3,3-dimethyl-2,3-dihydrobenzo[*b*]thiophen-5-yl)-1,2,3,3-tetramethyl-3*H*-indol-1-ium iodide (**36**):

To a solution of **34** (84 mg, 0.17 mmol) in CH_3CN (10 mL) was added iodomethane (0.1 mL, 1.6 mmol). The pale-yellow solution was then refluxed for 22 h, and the solvent removed in vacuo. The yellow precipitate was then collected by vacuum filtration, washing with diethyl ether (62 mg, 58 %).

m.p.: 177-179°C

^{1}H NMR (CD_3CN, 400 MHz) δ/ ppm: 7.41 (d, *J* = 7.8 Hz, 1H, H6), 7.37 (d, *J* = 8.0 Hz, 1H, H7), 7.34 (d, *J* = 7.8 Hz, 1H, H5), 7.32 (d, *J* = 7.9 Hz, 1H, H7′′), 7.24 (dd, *J* = 7.9, 1.8 Hz, 1H, H6′), 7.21 (d, *J* = 1.4 Hz, 1H, H4′), 7.10 (dd, *J* = 7.8, 1.9 Hz, 1H, H6′′), 7.06 (d, *J* = 1.36 Hz, 1H, H4′′), 3.39 (s, 3H, NCH_3), 3.31 (s, 2H, H2′), 3.30 (s, 2H, H2′′), 2.58 (s, 3H, $NCCH_3$), 1.44 (s, 3H, 3′-*gem*-CH_3), 1.40 (s, 3H, 3′-*gem*-CH_3), 1.40 (s, 3H, *gem*-CH_3), 1.39 (s, 3H, *gem*-CH_3), 1.38 (s, 3H, 3′′-*gem*-CH_3), 1.33 (s, 3H, 3′′-*gem*-CH_3).

^{13}C{^{1}H} NMR (CD_3CN, 101 MHz) δ/ ppm: 198.2 (C2), 149.7 (C3a′), 149.3 (C3a), 142.9 (C7a′), 142.0 (C7a′′), 140.4 (C7a), 140.0 (C3a′′), 134.9 (C4), 133.6 (C7), 133.0 (C6), 131.9 (C5), 131.9 (C5′ & C5′′), 129.7 (C6′′), 129.5 (C6′), 125.5 (C4′), 125.2 (C4′′), 123.2 (C7′), 122.6 (C7′′), 55.5 (C3), 48.2 (C3′), 48.1 (C3′′), 47.7 (C2′), 47.7 (C2′′), 40.2 (NCH_3), 27.5 (C3′-*gem*-CH_3), 27.3 (*gem*-CH3), 27.3 (3′-*gem*-CH_3), 22.4 (3′′-*gem*-CH_3), 22.1 (3′′-*gem*-CH_3), 15.3 (*gem*-CH_3).

FTIR (ATR, neat) ν / cm^{-1}: 2960, 2924, 1598 (C=N), 1456.

HRMS (TOF ES(+)) *m/z*: [M-I]$^+$ Calcd for $C_{32}H_{36}NS_2^+$ 498.2284, Found 498.2282.

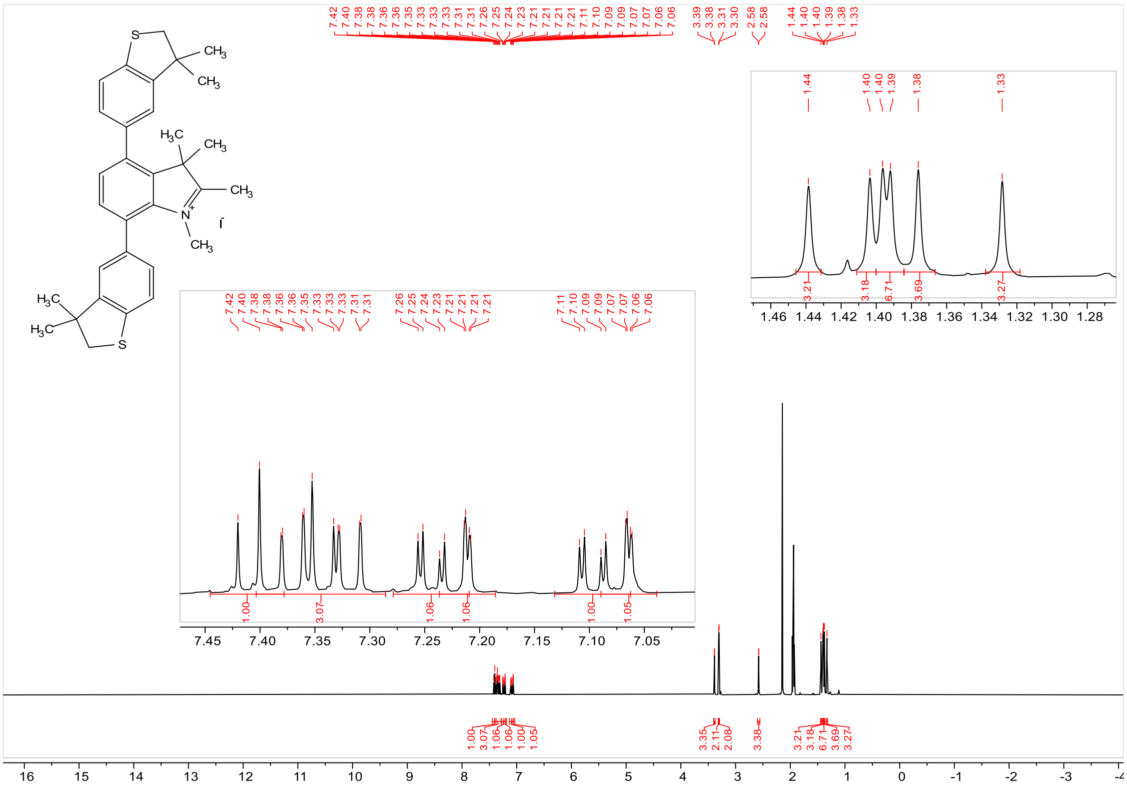

Figure S74 ^{1}H NMR spectrum of **36** in CD_3CN.

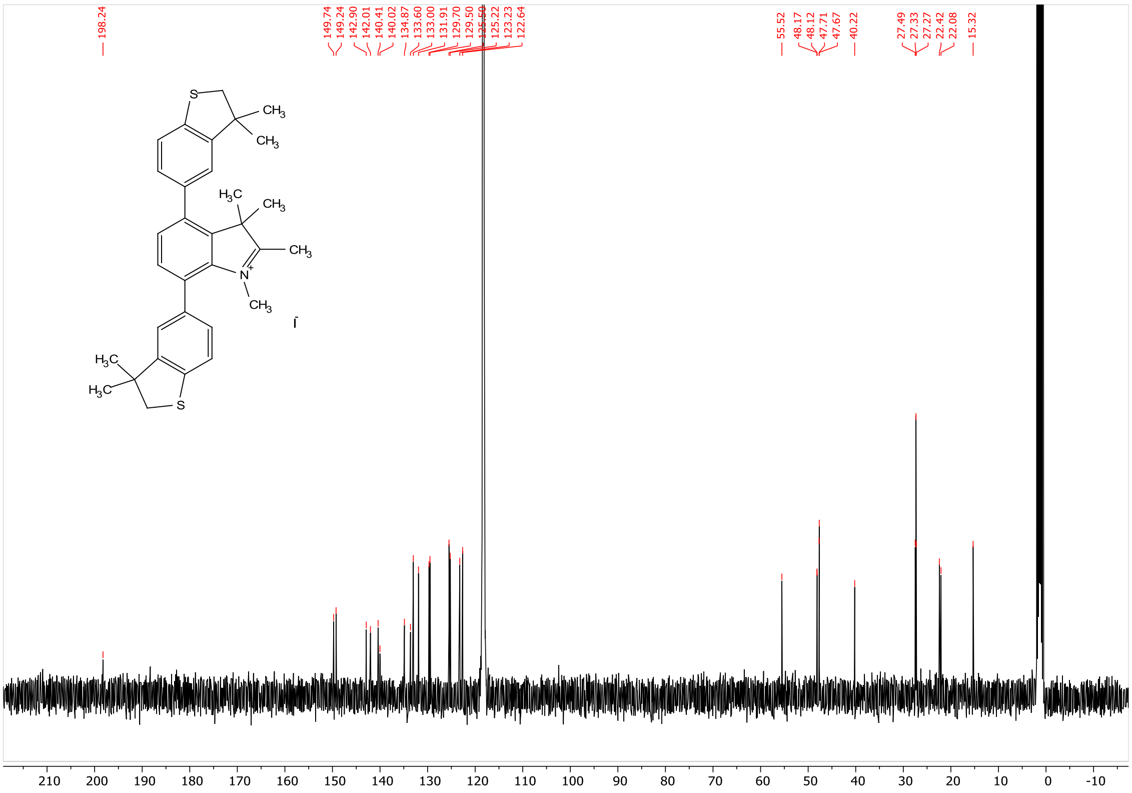

Figure S75 ^{13}C NMR spectrum of **36** in CD_3CN.

1,2,3,3-tetramethyl-4,7-bis(4-(methylthio)phenyl)-3*H*-indol-1-ium iodide (**37**):

A solution of **35** (50 mg, 0.12 mmol) and iodomethane (0.05 mL, 0.80 mmol) in CH_3CN (5 mL) was refluxed for 19 h, over which time the solution deepened in colour. The volatiles were then removed in vacuo, and the precipitate collected by vacuum filtration, washing with diethyl ether, to afford a beige powder (31 mg, 48 %).

m.p.: 214-216°C

^{1}H NMR (CD_3CN, 400 MHz) δ/ ppm: 7.42 (m, 4H), 7.39 (m, 3H), 7.35 (d, *J* = 7.9 Hz, H5), 7.29 (m, 2H), 3.40 (s, 3H, NCH_3), 2.58 (s, 3H, $NCCH_3$), 2.56 (s, 3H, S′-CH_3), 2.56 (s, 3H, S′′-CH_3), 1.36 (s, 6H, *gem*-CH_3).

^{13}C{^{1}H} NMR (CD_3CN, 101 MHz) δ/ ppm: 198.3 (C2), 141.3, 140.4, 140.1, 140.0, 135.0, 133.7, 133.1, 132.0 (C5), 131.5, 131.0,131.0, 126.6, 126.3, 55.6 (C3), 40.2 (NCH3), 22.2 (gem-CH3), 15.3 (S′-CH3), 15.29 (S′′-CH3).

FTIR (ATR, neat) ν / cm^{-1}: 3012, 2970, 2909, 1639 (C=N).

HRMS (TOF ES(+)) *m/z*: $[M-I]^+$ Calcd for $C_{26}H_{28}NS_2^+$ 418.1658, Found 418.1658.

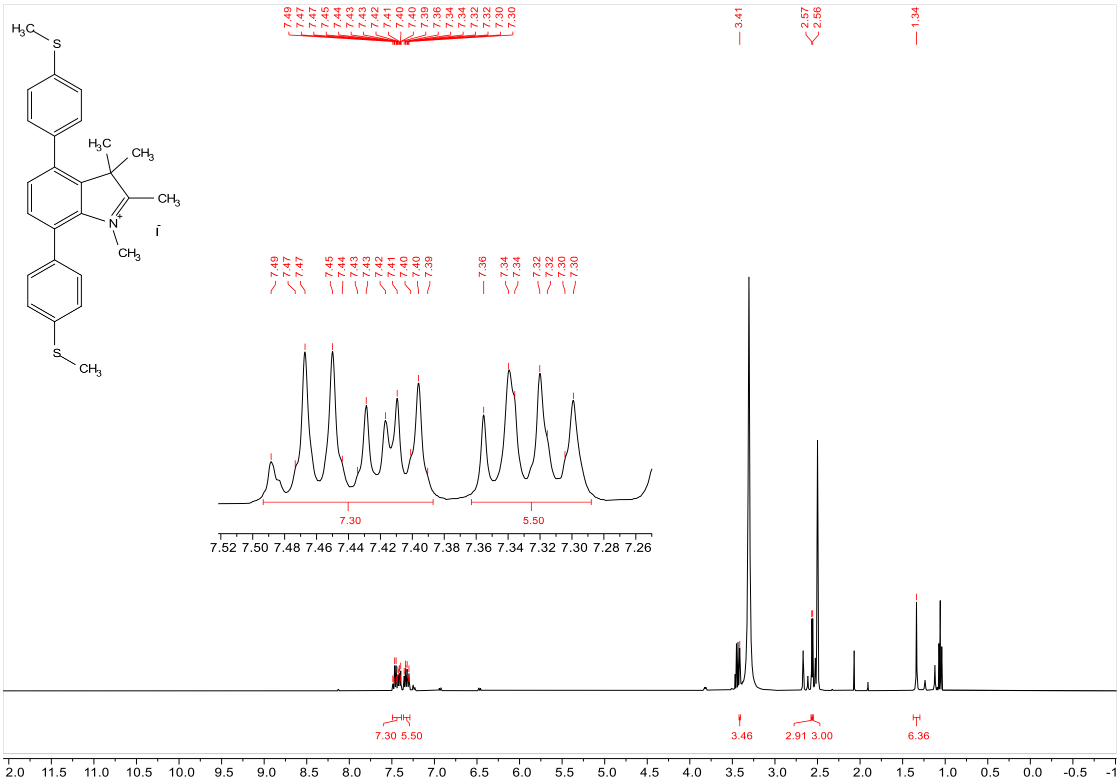

Figure S76 ^{1}H NMR spectrum of **37** in CD_3CN.

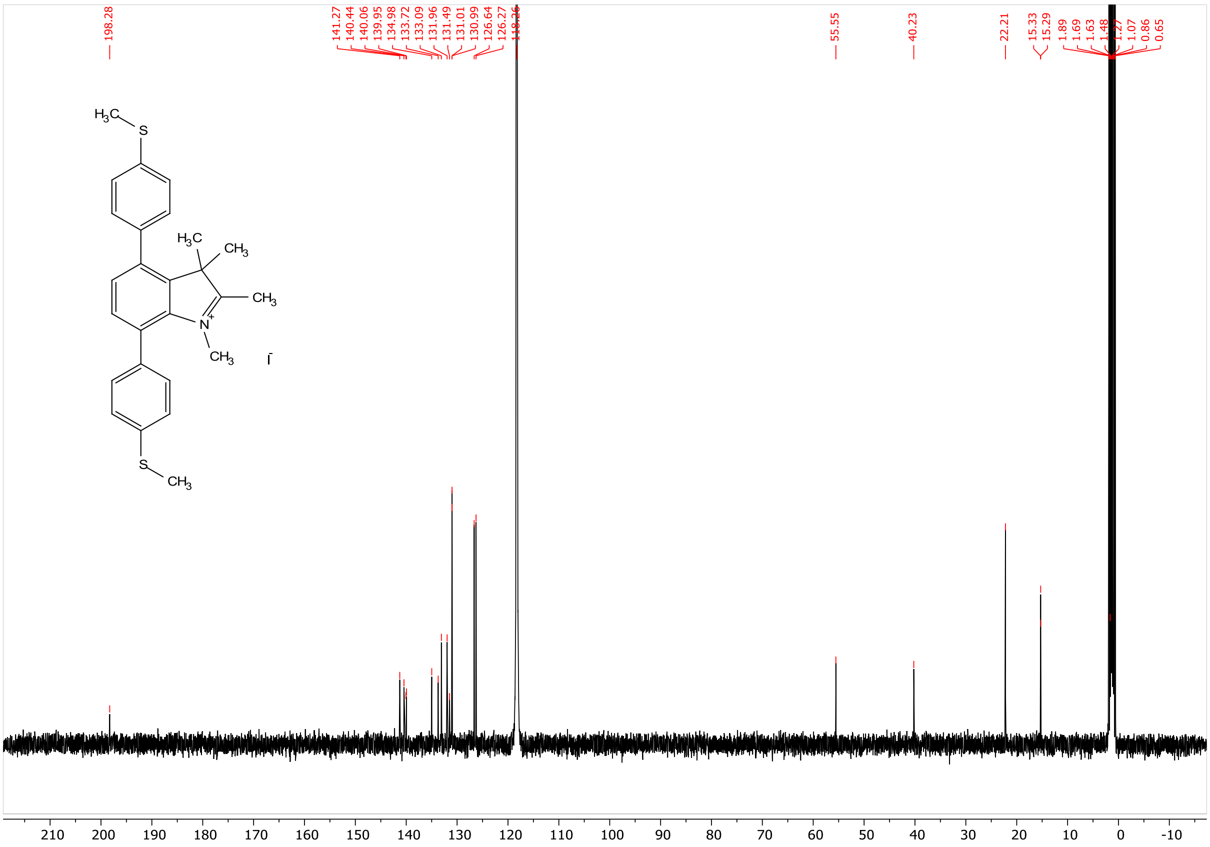

Figure S77 ^{13} NMR Spectrum of **37** in CD_3CN.

1',3',3'-trimethyl-4',7'-bis(4-(methylthio)phenyl)spiro[chromene-2,2'-indoline] (**SP13**):

A mixture of ethanol (10 mL) and piperidine (0.2 mL, 2 mmol) was sparged (Ar) for 30 min. **37** (33 mg, 0.060 mmol) and 2-hydroxy-5-nitrobenzaldehyde **32** (21 mg, 0.12 mmol) were added. The orange solution was then refluxed for 3 h, over which time a precipitate formed. The precipitate was collected by vacuum filtration, washing with cold ethanol, to afford a bright lime green powder (22 mg, 65 %).

m.p.: 251-253°C

^{1}H NMR ($CDCl_3$, 400 MHz) δ/ ppm: 7.98 (dd, *J* = 9.0, 2.7 Hz, 1H, H7), 7.92 (d, *J* = 2.7 Hz, 1H, H5), 7.37-7.24 (m, 8H), 7.04 (d, *J* = 7.8 Hz, 1H, H6'), 6.89 (d, *J* = 10.4 Hz, 1H, H4), 6.79 (d, *J* = 9.0 Hz, H8), 6.68 (d, *J* = 7.8 Hz, 1H, H5'), 5.79 (d, *J* = 10.3 Hz, 1H, H3), 2.52 (br s, 6H, S''-CH_3 and S'''-CH_3), 2.35 (s, 3H, NCH_3), 1.25 (s, 3H, *gem*-CH_3), 0.78 (s, 3H, *gem*-CH_3).

^{13}C{^{1}H} NMR ($CDCl_3$, 101 MHz) δ/ ppm: 160.1 (C6), 145.2 (C8a), 141.0 (C7a'), 137.8, 137.5 (C7'), 137.5 (C4'), 137.2, 133.7 (C3a'), 130.5 (C6'), 130.2, 128.8 (C4), 126.0 (C7), 125.9, 125.8, 123.1 (C5), 122.6 (C5'), 121.8 (C3), 118.5 (C4a), 115.5 (C8), 108.0 (C2), 52.9 (C3'), 33.4 (NCH_3), 25.8 (*gem*-CH_3), 21.2 (*gem*-CH_3), 15.9 (S''-CH_3 and S'''-CH_3).

FTIR (ATR, neat) ν / cm^{-1}: 2992, 1650 (C=C), 1279, 954 (C-O).

HRMS (TOF ES(+)) *m/z*: $[M+H]^+$ Calcd for $C_{33}H_{31}N_2O_3S_2^+$ 567.1771, Found 567.1771.

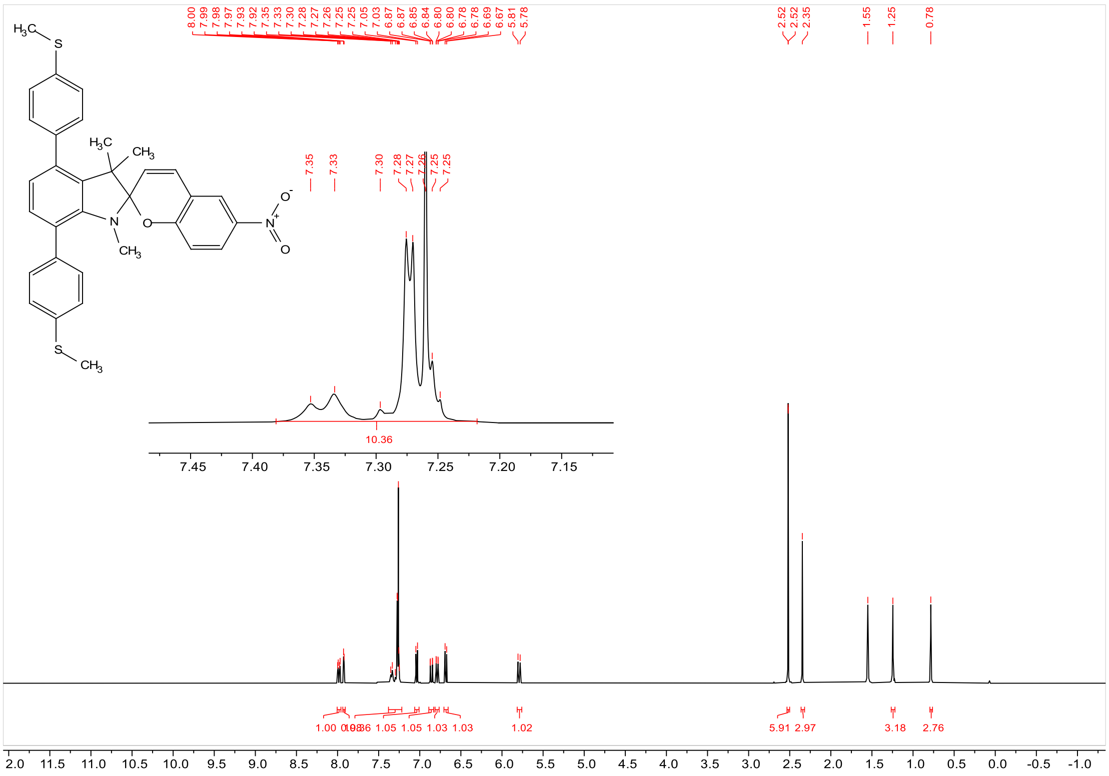

Figure S78 ^{1}H NMR spectrum of **SP13** in $CDCl_3$.

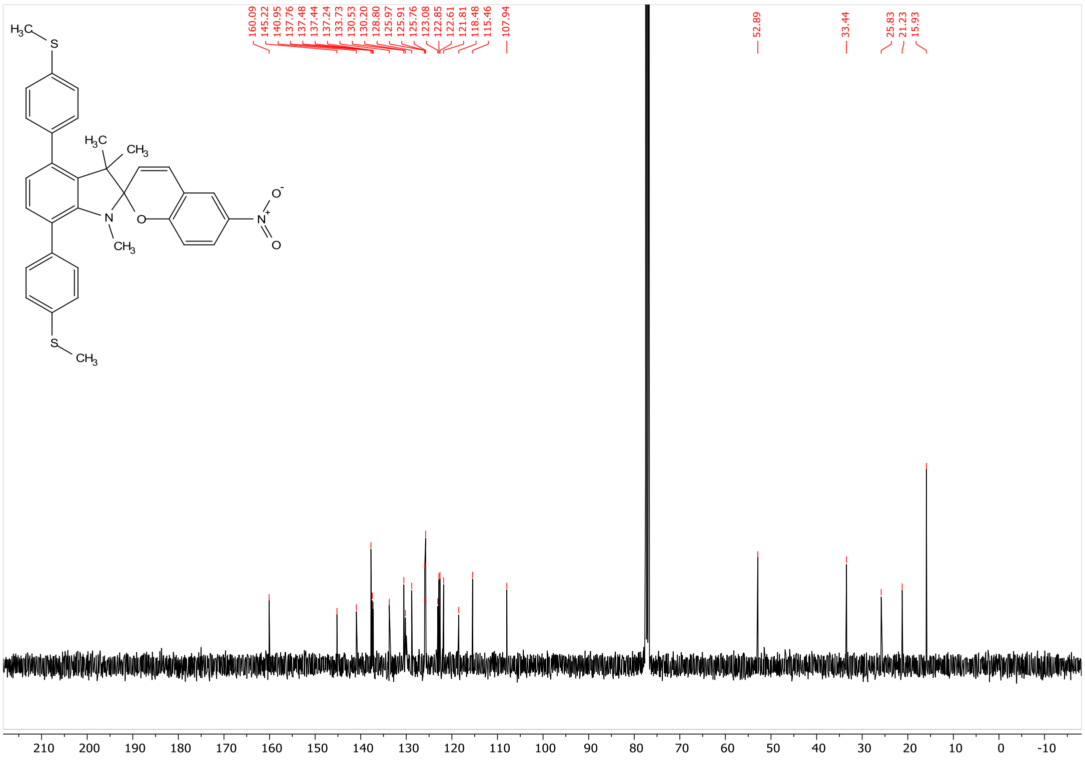

Figure S79 ^{13}C NMR spectrum of **SP13** in $CDCl_3$.

4′,7′-bis(3,3-dimethyl-2,3-dihydrobenzo[*b*]thiophen-5-yl)-1',3',3'-trimethylspiro-[chromene-2,2'-indoline] (**SP14**):

A mixture of ethanol (10 mL) and piperidine (0.6 mL, 6.1 mmol) was sparged (Ar) for 30 min, and **36** (54 mg, 0.086 mmol) and 2-hydroxy-5-nitrobenzaldehyde **32** (30 mg, 0.18 mmol) added. The bright orange solution was refluxed for 19 h, after which the solvent was removed in vacuo. A precipitate formed, which was then extracted into ethyl acetate (30 mL) and the extract washed with sodium bicarbonate. Purification by flash chromatography (20% dichloromethane in hexanes) monitoring by UV activity afforded a gold powder (41 mg, 74 %).

m.p.: 265-267°C

^{1}H NMR ($CDCl_3$, 400 MHz) δ/ ppm: 7.99 (dd, *J* = 9.0, 2.7 Hz, 1H, H7), 7.93 (d, *J* = 2.7 Hz, 1H, H5), 7.18 (d, *J* = 7.8 Hz, 1H, H6′), 7.18-7.03 (m, 6H), 6.86 (d, *J* = 10.5 Hz, 1H, H4), 6.81 (d, *J* = 9.0 Hz, 1H, H8), 6.71 (d, *J* = 7.8 Hz, 1H, H5′), 5.81 (d, *J* = 10.5 Hz, 1H, H3), 3.22 (br s, 4H, H2′′ and H2′′′), 2.34 (s, 3H, NCH_3), 1.42 (s, 3H, 3′′-*gem*-CH_3), 1.40 (s, 3H, 3′′-*gem*-CH_3), 1.36 (s, 3H, 3′′′-*gem*-CH_3), 1.34 (s, 3H, 3′′′-*gem*-CH_3), 1.24 (s, 3H, *gem*-CH_3), 0.78 (s, 3H, *gem*-CH_3).

^{13}C{^{1}H} NMR ($CDCl_3$, 101 MHz) δ/ ppm: 160.1 (C6), 147.4, 145.1 (C7a), 140.9 (C8a), 139.5, 139.2, 138.2, 137.2 (C4′ and C7′), 137.0, 133.7 (C3a′), 130.6, 128.7 (C4), 126.0 (C7), 124.3, 123.5, 122.8 (C8), 122.5 (C8), 122.5 (C5′), 121.9 (C3), 121.6 (C6′), 118.5 (C4a), 115.5 (C8), 108.0 (C2), 52.8 (C3′), 47.5 (C2′′), 47.4 (C2′′′), 33.2 (NCH3), 27.6 (3′′-gem-CH3 and 3′′′-gem-CH3), 25.8 (gem-CH3), 21.2 (gem-CH3).

FTIR (ATR, neat) ν / cm^{-1}: 2995, 2950, 2851, 1655 (C=C), 1280, 952 (C-O).

HRMS (TOF ES(+)) *m/z*: $[M+H]^+$ Calcd for $C_{39}H_{39}N_2O_3S_2^+$ 647.2397, Found 647.2399.

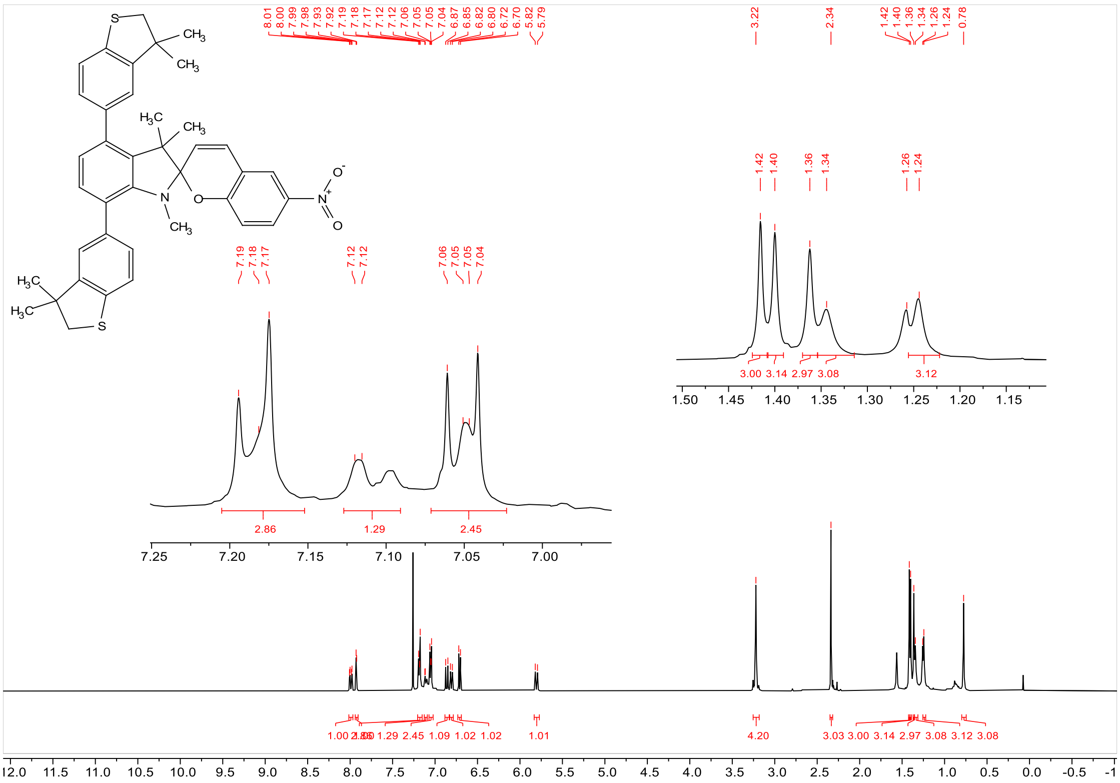

Figure S80 ^{1}H NMR spectrum of **SP14** in CDCl$_3$.

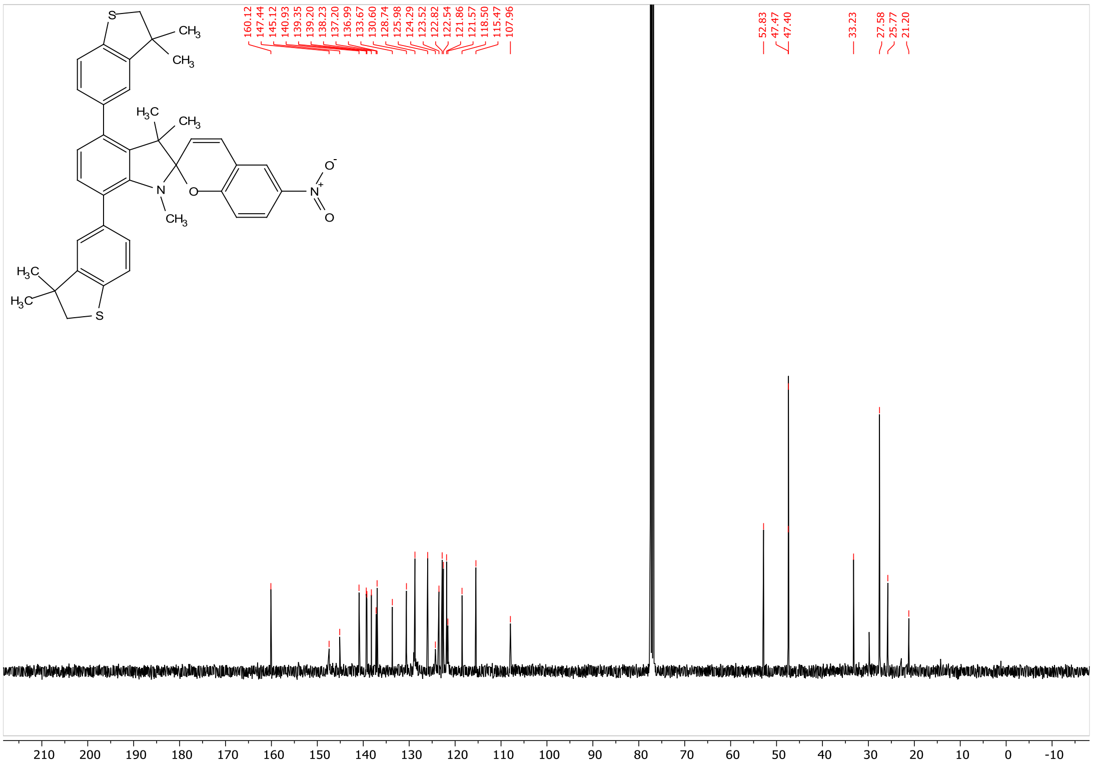

Figure S81 ^{13}C NMR spectrum of **SP14** in CDCl$_3$.

X-ray Crystallography

Single crystals with suitable dimensions were selected and mounted on a XtaLAB Synergy, Single source at home/near, HyPix diffractometer or Oxford Diffraction Gemini-R Ultra. The crystal was kept at a steady T(K) during data collection. The structure was solved with the ShelXT 2018/2 solution program using dual methods and by using Olex2 as the graphical interface.[26] The model was refined with XL using full matrix least squares minimisation on $\boldsymbol{F^2}$.[27] All non-hydrogen atoms were refined anisotropically. Hydrogen atom positions were calculated geometrically and refined using the riding model. Graphics of the molecular structures have been created by using ORTEP.

Single crystal X-ray-diffraction studies were carried out on several structures. The structures are shown below in Figures S82 to S91, respectively. Selected bond lengths and angles are collected in Table S1 and S2. Table S3 and S4 shows the crystallographic data and structural refinement parameters.

Compound **SP10** crystallises with two independent molecules in the asymmetric unit. These are denoted as **SP10A** and **SP10B**. The two molecules are enantiomers and adopt slightly different conformations. The differences between the conformations are ascribed to packing rather than molecular or electronic properties.[28] The joint crystallisation of enantiomers has only been reported a few times for spiropyrans.[29]

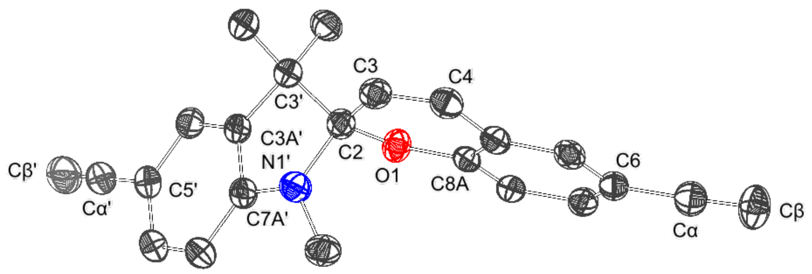

Figure S82 ORETP (50% probability level) of the molecular structure of **6** and atom labelling scheme. Hydrogens removed for clarity.

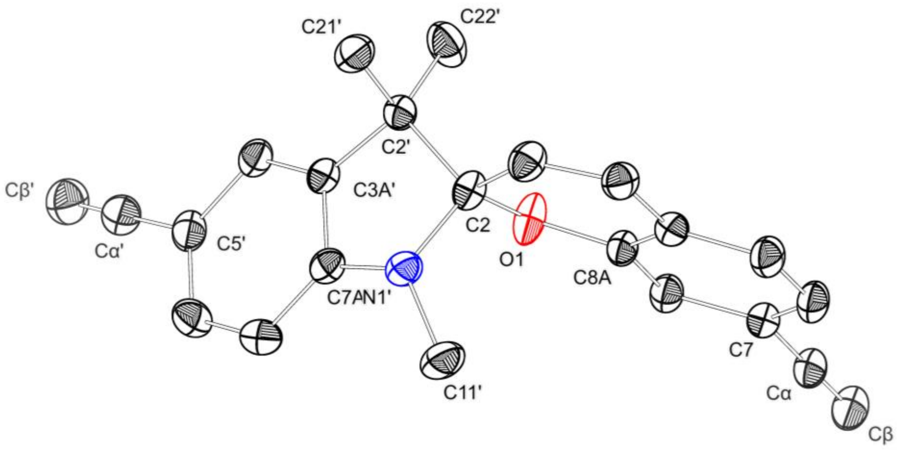

Figure S83 ORETP (50% probability level) of the molecular structure of **7** and atom labelling scheme. Hydrogens removed for clarity.

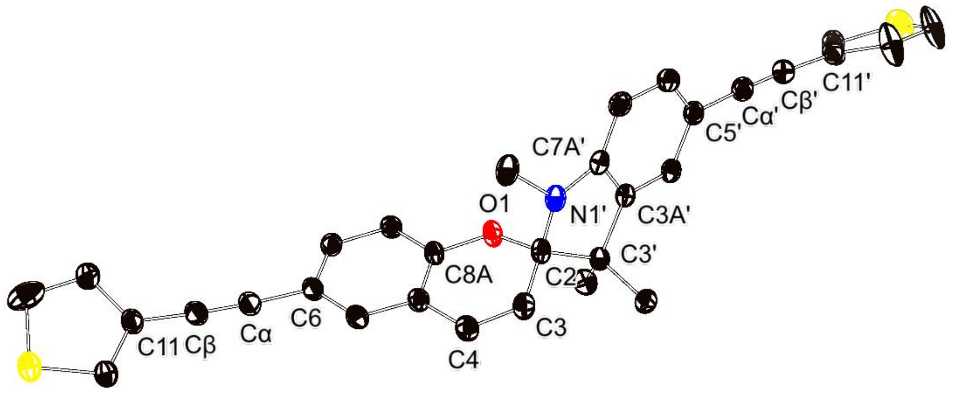

Figure S84 ORETP (50% probability level) of the molecular structure of **SP5** and atom labelling scheme. Hydrogens removed for clarity.

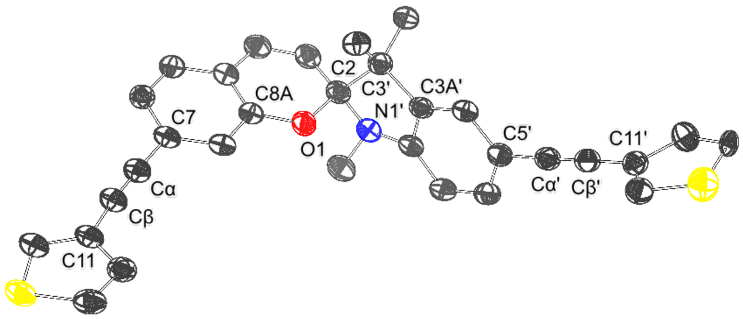

Figure S85 ORETP (50% probability level) of the molecular structure of **SP6** and atom labelling scheme. Hydrogens and solvent removed for clarity.

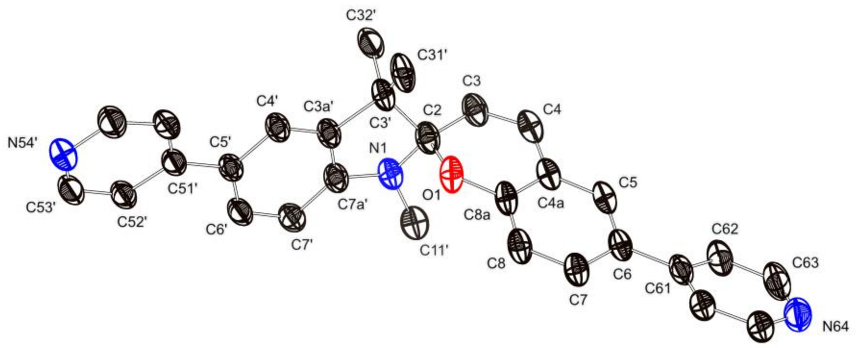

Figure S86 ORETP (50% probability level) of the molecular structure of **SP7** and atom labelling scheme. Hydrogens removed for clarity.

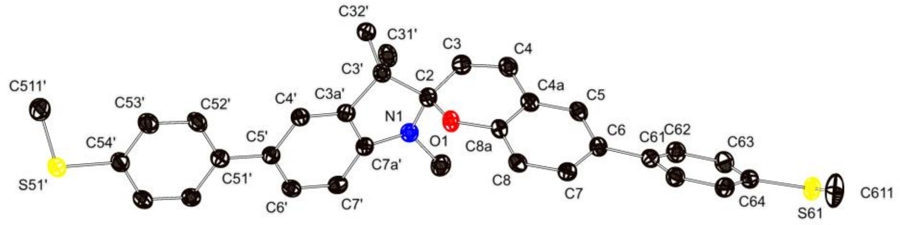

Figure S87 ORETP (50% probability level) of the molecular structure of **SP8** and atom labelling scheme. Hydrogens removed for clarity.

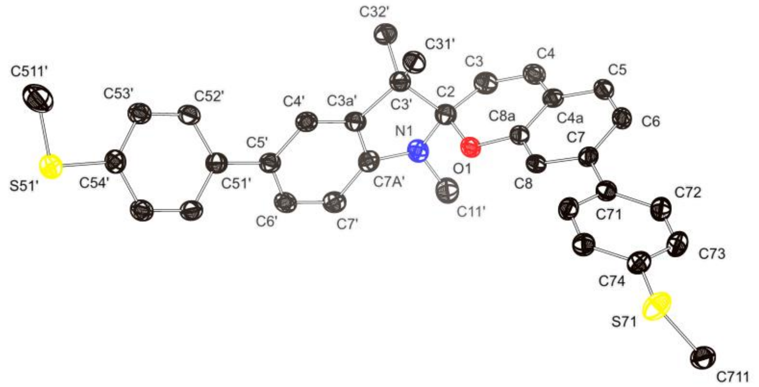

Figure S88 ORETP (50% probability level) of the molecular structure of **SP9** and atom labelling scheme. Hydrogens removed for clarity.

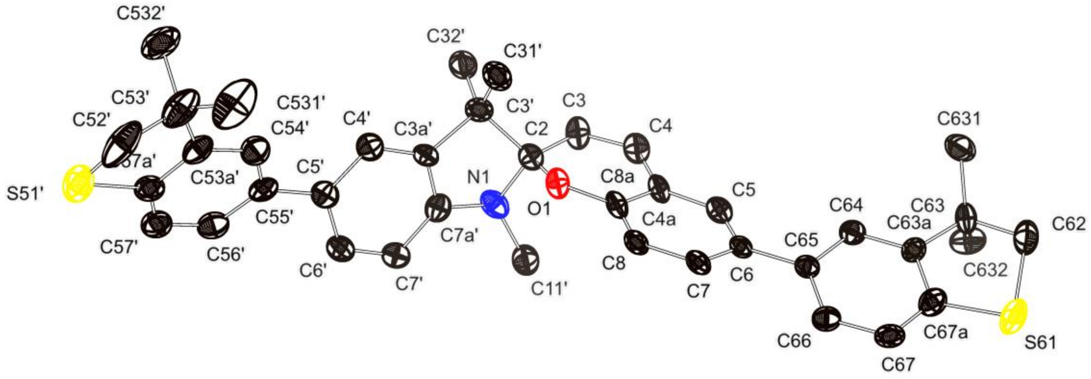

Figure S89 ORETP (50% probability level) of the molecular structure of **SP10** and atom labelling scheme. Hydrogens removed for clarity.

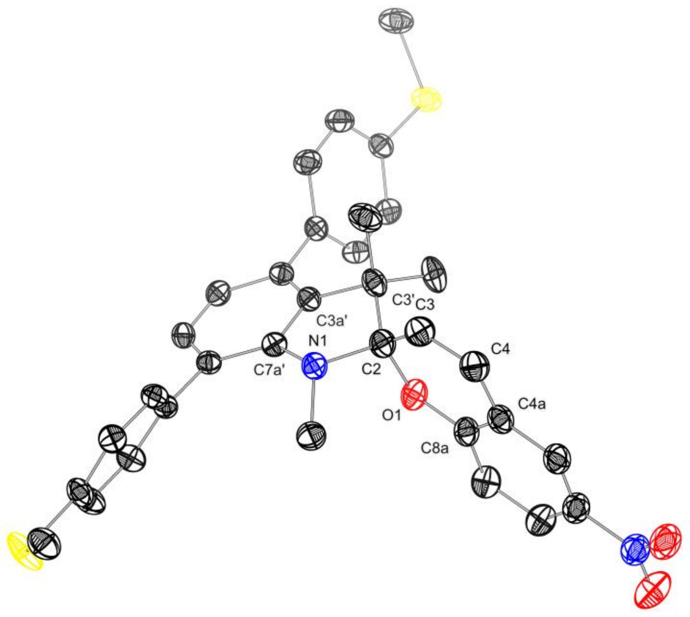

Figure S90 ORETP (50% probability level) of the molecular structure of **SP13** and atom labelling scheme. Hydrogens removed for clarity.

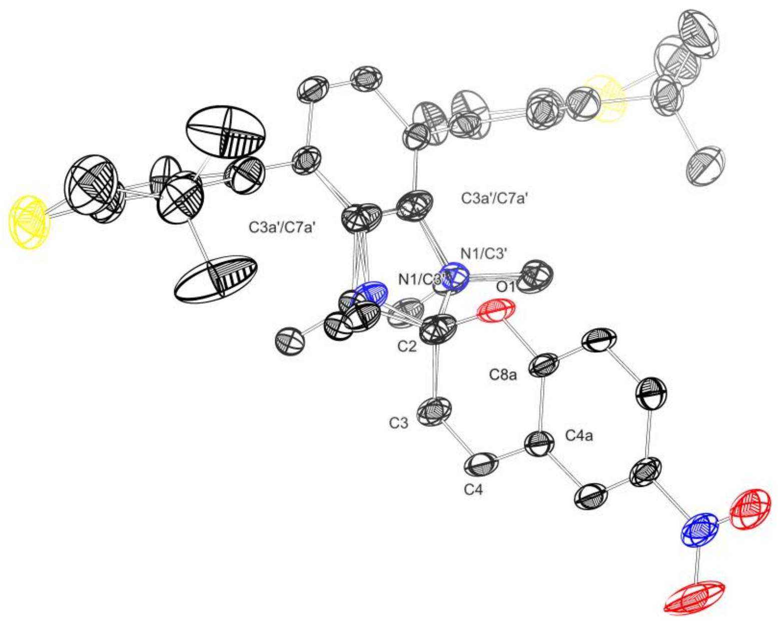

Figure S91 ORETP (50% probability level) of the molecular structure of **SP14** and atom labelling scheme. Hydrogens removed for clarity.

All compounds studied by single crystal X-ray diffraction show a substituted indoline and chromene moiety orthogonally connected through a tetrahedral spiro-carbon. The dihedral angle between the planes of the two rings range from 86.9 to 89.9°. The pyyrole ring of the indoline moiety adopts an envelope conformation due to the pyramid environment required by the N1 atom. This puckering is more pronounced in **SP6**. This conformation results in the C2 carbon being out of plane of the indoline ring by between 27.3 to 31.0°. All compounds adopt slightly different bending of the chromene moiety. For compounds **SP10**, **SP8**, **SP7**, and **SP13** the chromene moiety bends away from the *geminal*-methyl groups, and for compound **SP9**, the chromene moiety bends towards the *geminal*-methyl groups. These conformational differences have been attributed to packing effects.[28]

SP6 crystallises in the P*nma* space group. The spiropyran structure itself sits on a mirror, with Z′ = 0.5, meaning that half of the molecule is present in the asymmetric unit, with the other half being symmetry equivalent atoms. This leads to a high degree of static disorder in the structure itself due to the various mirrored configurations within the unit cell. Despite the interesting solution, the presence of two orthogonal ring systems is evident. Furthermore, the geometry of the contact groups can be distinguished, with the DMBT substituents lying in the same plane as each other, perpendicular to the indoline moiety. Elucidation of the specific structural characteristics of **SP14** proved problematic, owing to the high degree of disorder.

The bond length between the C3-C4 atoms ranges from 1.330(2) – 1.337(8) consistent with the double bond assignment. Most bond lengths around the C_{spiro} carbon are within standard uncertainties for all compounds. Whilst the C2-N1 bond length in **SP7** is longer than the other spiropyrans it is still within the literature range.[28b, 30] The C2-O1 bond length is of interest due to its dissociation in the spiropyran to merocyanine transformation. In the spiropyrans studied here, this bond ranges from 1.462(2) to 1.472(6). This bond in an unsubstituted spiropyran is 1.471(2) Å.[30a]. Introducing a para-nitro group on the chromene lengthens this bond to 1.478(3) Å,[31] whilst a para-methoxy group shortens it to 1.467(2) Å.[32] These bond lengths are also in agreement with related structures in the literature.[28b, 30]

The ethynyl groups show typical linearity with a deviation of no more than 3.3° for all compounds. Bond lengths between the Cβ to Cα, and Cβ to Cα for **4** and **5** are similar. A slight, albeit not significant, increase in the bond length is seen by the presence of the thiophene groups. However, **SP5** and **SP6** show smaller Cβ to Cα bond lengths than their terminal alkyne counterparts. The thiophene group is closest to being co-planar on chromene moiety of **SP5** and **SP6**, with dihedral angles of 26.45 and 12.64°, respectively. In contrast, the thiophene on the indoline moiety is near perpendicular.

Table S1 Selected bond lengths, angles, and dihedral angles (°) for Spiropyrans. α is angle between mean planes through O1-C2-C3 and N1-C2-C3’. β is the angle between the mean planes through C3’-C3a’-C7a’-N1 and N1-C2-C3’. γ is the angle between the mean planes through C3-C2-O1 and O1-C8a-C4a-C4. δ is the dihedral angle through the planes of the chromene ring and the aryl substituent or 4’-aryl substituent and indoline ring. ε is the dihedral angle through the planes of the indoline ring and the aryl substituent or 7’-aryl substituent and indoline ring.

Compound	6	7	SP5	SP6
**C2-O1**	1.476(2)	1.461(2)	1.466(2)	1.467(3)
**C2-C3**	1.498(3)	1.496(2)	1.499(2)	1.498(5)
**C2-N1**	1.451(2)	1.460(2)	1.457(2)	1.457(4)
**C2-C3’**	1.573(3)	1.567(2)	1.575(2)	1.563(4)
**C3-C4**	1.327(3)	1.330(2)	1.326(5)	1.332(3)
**N1-CH_3**	1.453(3)	1.456(3)	1.456(2)	1.455(5)
**O1-C8a**	1.362(2)	1.368(2)	1.366(2)	1.365(4)
**N1-C7a’**				
**C11’-Cβ’**			1.433(3)	1.430(5)
**Cβ’-Cα’**	1.184(4)	1.189(2)	1.206(3)	1.196(5)
**Cα’-C5’**	1.440(3)	1.442(2)	1.436(3)	1.442(4)
**C6/7-Cα**	1.443(3)	1.440(2)	1.437(3)	1.431(4)
**Cα-Cβ**	1.181(3)	1.193(2)	1.204(3)	1.201(5)
**Cβ-C11**			1.430(3)	1.425(4)
**O1-C2-C3**	111.7(2)	112.0(1)	112.2(1)	112.2(2)
**O1-C2-N1**	108.7(1)	109.1(1)	108.5(1)	105.5(2)
**C3-C2-N1**	110.8(2)	111.3(1)	111.1(1)	112.9(3)
**O1-C2-C3’**	104.8(1)	104.9(1)	106.1(1)	107.8(2)
**C3-C2-C3’**	117.4(2)	116.4(1)	115.1(1)	114.1(3)
**N1-C2-C3’**	102.8(1)	102.4(1)	103.2(1)	103.6(2)
**C8a-O1-C2**				
**C7a’-N1-C2**				
**C2-C3-C4**				
**C11’-Cβ’-Cα’**			176.7(2)	179.3(4)
**Cβ’-Cα’-C5’**	178.5(3)	177.9(2)	177.1(2)	178.3(3)
**C6/7-Cα-Cβ**	178.0(2)	179.1(2)	175.3(2)	175.3(2)
**Cα-Cβ-C11**			176.9(2)	176.9(2)
**α**	86.40	86.72	87.79	89.39
**β**				

**γ**				
**δ**			63.62	84.25
**ε**			26.45	12.64
**C3a'-C7a'-N1-C2**	17.0(2)	17.4(2)	14.1(2)	21.7(3)
**C31'-C3'-C2-O1**	86.6(2)	83.9(1)	89.3(1)	86.1(3)
**C3-C2-O1-C8a**	20.1(2)	21.6(2)	17.8(2)	10.0(4)

Table S2 Selected bond lengths, angles, and dihedral angles (°) for spiropyrans. α is angle between mean planes through O1-C2-C3 and N1-C2-C3'. β is the angle between the mean planes through C3'-C3a'-C7a'-N1 and N1-C2-C3'. γ is the angle between the mean planes through C3-C2-O1 and O1-C8a-C4a-C4. δ is the dihedral angle through the planes of the chromene ring and the aryl substituent or 4'-aryl substituent and indoline ring. ε is the dihedral angle through the planes of the indoline ring and the aryl substituent or 7'-aryl substituent and indoline ring.

**Compound**	**SP10A**	**SP10B**	**SP8**	**SP9**	**SP7**	**SP14**	**SP13**
**C2-O1**	1.468(6)	1.472(6)	1.466(2)	1.470(2)	1.462(2)	1.484	1.469(2)
**C2-C3**	1.494(8)	1.479(8)	1.496(2)	1.501(2)	1.500(2)	1.469	1.504(3)
**C2-N1**	1.444(7)	1.455(8)	1.456(2)	1.445(2)	1.460(2)	1.461(8)	1.460(3)
**C2-C3'**	1.580(8)	1.607(8)	1.573(2)	1.569(2)	1.566(2)	1.554(8)	1.553(3)
**C3-C4**	1.337(8)	1.336(8)	1.330(2)	1.336(2)	1.335(2)	1.337	1.330(3)
**N1-CH_3**	1.464(8)	1.461(8)	1.449(2)	1.457(3)	1.446(2)	1.467(9)	1.468(3)
**O1-C8a**	1.378(7)	1.358(7)	1.370(2)	1.364(2)	1.364(2)	1.350	1.352(2)
**N1-C7a'**	1.398(7)	1.373(8)	1.394(2)	1.394(2)	1.398(2)	1.422(8)	1.422(2)
**O1-C2-C3**	112.2(4)	112.6(4)	111.6(1)	111.3(1)	111.2(1)	113.6	112.4(2)
**O1-C2-N1**	108.3(4)	107.7(4)	109.1(1)	105.5(1)	108.1(1)	99.4	107.4(1)
**C3-C2-N1**	111.8(4)	111.3(5)	111.7(1)	112.6(1)	112.0(1)	115.3	109.8(2)
**O1-C2-C3'**	105.3(4)	104.6(4)	105.6(1)	107.7(1)	105.9(1)	111.4	105.2(1)
**C3-C2-C3'**	115.9(4)	117.5(4)	115.4(1)	115.6(1)	115.8(1)	111.3	116.3(2)
**N1-C2-C3'**	102.6(4)	102.3(4)	103.0(1)	103.4(1)	103.1(1)	105.1(4)	105.1(2)
**C8a-O1-C2**	120.5(4)	121.2(4)	120.3(1)	121.8(1)	121.2(1)	122.2	122.9(1)
**C7a'-N1-C2**	108.3(4)	109.5(5)	109.0(1)	108.6(1)	107.7(1)	107.3(5)	107.3(1)
**C2-C3-C4**	122.2(5)	122.4(5)	122.8(1)	122.7(1)	123.0(1)	123.0	123.5(2)
**α**	87.5	86.9	87.4	89.9	88.0	83.72	87.16
**β**	31.0	29.2	27.3	28.7	30.7	27.27	27.17
**γ**	-23.5	-21.1	-23.5	19.4	-20.5	3.48	4.70
**δ**	19.8	22.8	29.1	33.5	18.4	74.18	84.54
**ε**	21.2	25.7	7.9	36.3	11.6	73.72	49.97
**C3a'-C7a'-N1-C2**	-18.5(6)	18.1(6)	17.8(2)	19.2(2)	18.3(2)	-12.5(7)	-12.4(2)
**C31'-C3'-C2-O1**	-36.5(5)	36.7(6)	33.3(1)	37.4(2)	37.5(2)	-45.7	-39.1(2)
**C3-C2-O1-C8a**	27.2(6)	-24.4(7)	-27.1(2)	22.3(2)	-25.3(2)	-4.1	6.6(2)
**Sum of angles at N1**	346.8(8)	346.9(9)	350.5(2)	349.9(2)	347.7(2)	340.2(9)	340.3(2)

Table S3 Crystallographic data and structural refinement parameters

Compound	**6**	**7**	**SP5**	**SP6** (needle)
Crystal data				
Chemical formula	$C_{23}H_{19}NO$	$C_{23}H_{19}NO$	$C_{31}H_{23}NOS_2$	$2(C_{31}H_{23}NOS_2)\cdot CH_2Cl_2$
M_r	325.39	325.39	489.62	1064.17
Crystal system, space group	Monoclinic, $P2_1/c$	Monoclinic, $P2_1/n$	Monoclinic, $P2_1/c$	Orthorhombic, *Pccn*
Temperature (K)	100	100	100	100
a, *b*, *c* (Å)	8.3153 (2), 19.8667 (3), 11.2165 (2)	7.9496 (2), 11.2691 (3), 20.1496 (5)	15.4496 (5), 10.4560 (3), 15.9716 (4)	18.9672 (8), 22.2873 (11), 12.3392 (5)
α, β, γ (°)	90, 104.231 (2), 90	90, 101.022 (3), 90	90, 105.952 (3), 90	90, 90, 90
V (Å^3)	1796.08 (6)	1771.80 (8)	2480.72 (13)	5216.1 (4)
Z	4	4	4	4
Radiation type	Cu $K\alpha$	Mo $K\alpha$	Mo $K\alpha$	Mo $K\alpha$
μ (mm^{-1})	0.57	0.07	0.24	0.33
Crystal size (mm)	0.22 × 0.14 × 0.05	0.32 × 0.25 × 0.14	0.39 × 0.24 × 0.06	0.43 × 0.07 × 0.02
Data collection				
Diffractometer	Oxford Diffraction Gemini-R Ultra	Oxford Diffraction Xcalibur-S	Oxford Diffraction Xcalibur-S	Oxford Diffraction Xcalibur-S
Absorption correction	Multi-scan *CrysAlis PRO* 1.171.39.46 (Rigaku Oxford Diffraction, 2018) Empirical absorption correction using spherical harmonics, implemented in SCALE3 ABSPACK scaling algorithm.	Multi-scan *CrysAlis PRO* 1.171.38.46 (Rigaku Oxford Diffraction, 2015) Empirical absorption correction using spherical harmonics, implemented in SCALE3 ABSPACK scaling algorithm.	Multi-scan *CrysAlis PRO* 1.171.39.46 (Rigaku Oxford Diffraction, 2018) Empirical absorption correction using spherical harmonics, implemented in SCALE3 ABSPACK scaling algorithm.	Multi-scan *CrysAlis PRO* 1.171.39.46 (Rigaku Oxford Diffraction, 2018) Empirical absorption correction using spherical harmonics, implemented in SCALE3 ABSPACK scaling algorithm.
T_{min}, T_{max}	0.859, 1.0	0.985, 1.0	0.937, 1.0	0.966, 1.0
No. of measured, independent and observed [$I > 2\sigma(I)$] reflections	35578, 3211, 2444	10927, 5761, 3932	27891, 8200, 6006	27356, 8584, 5420
R_{int}	0.076	0.029	0.033	0.061
(sin θ/λ)$_{max}$ (Å^{-1})	0.599	0.753	0.751	0.753
Refinement				
$R[F^2 > 2\sigma(F^2)]$, $wR(F^2)$, S	0.050, 0.112, 1.00	0.063, 0.132, 1.00	0.056, 0.121, 1.00	0.065, 0.145, 1.00
No. of parameters	302	229	326	340
No. of restraints	0	0	2	2
H-atom treatment	All H-atom parameters refined	H-atom parameters constrained	H-atom parameters constrained	H-atom parameters constrained
$\Delta\rangle_{max}$, $\Delta\rangle_{min}$ (e Å^{-3})	0.19, -0.21	0.40, -0.22	0.53, -0.69	0.86, -0.75
Absolute structure	–	–	–	–
Absolute structure parameter	–	–	–	–
CCDC #				

Table S4 Crystallographic data and structural refinement parameters

	**SP6** (plate)	**SP7**	**SP8**	**SP9**
Crystal data				
Chemical formula	$C_{31}H_{23}NOS_2$	$C_{29}H_{25}N_3O$	$C_{33}H_{31}NOS_2$	$C_{33}H_{31}NOS_2$
M_r	489.62	431.52	521.71	521.71
Crystal system, space group	Monoclinic, $P2_1/c$	Monoclinic, $P2_1/c$	Monoclinic, $P2_1/c$	Monoclinic, $P2_1/c$
Temperature (K)	100	102	100	100
a, *b*, *c* (Å)	6.8605 (1), 10.3620 (3), 35.5163 (8)	11.0850 (1), 10.4684 (1), 22.4926 (2)	11.8616 (1), 10.4670 (1), 21.7376 (2)	12.0791 (2), 7.3997 (1), 30.3737 (5)
α, β, γ (°)	90, 93.710 (2), 90	90, 93.999 (1), 90	90, 90.764 (1), 90	90, 101.351 (2), 90
V (Å^3)	2519.51 (10)	2603.74 (4)	2698.60 (4)	2661.75 (7)
Z	4	4	4	4
Radiation type	Cu $K\alpha$	Cu $K\alpha$	Cu $K\alpha$	Cu $K\alpha$
μ (mm^{-1})	2.10	0.53	1.99	2.01
Crystal size (mm)	0.24 × 0.23 × 0.02	0.25 × 0.17 × 0.13	0.13 × 0.07 × 0.05	0.21 × 0.15 × 0.08
Data collection				
Diffractometer	Oxford Diffraction Gemini-R Ultra	XtaLAB Synergy, Single source at home/near, HyPix	XtaLAB Synergy, Single source at home/near, HyPix	XtaLAB Synergy, Single source at home/near, HyPix
Absorption correction	Multi-scan *CrysAlis PRO* 1.171.39.46 (Rigaku Oxford Diffraction, 2018) Empirical absorption correction using spherical harmonics, implemented in SCALE3 ABSPACK scaling algorithm.	Multi-scan *CrysAlis PRO* 1.171.41.104a (Rigaku Oxford Diffraction, 2021) Empirical absorption correction using spherical harmonics, implemented in SCALE3 ABSPACK scaling algorithm.	Multi-scan *CrysAlis PRO* 1.171.40.53 (Rigaku Oxford Diffraction, 2019) Empirical absorption correction using spherical harmonics, implemented in SCALE3 ABSPACK scaling algorithm.	Gaussian *CrysAlis PRO* 1.171.41.103a (Rigaku Oxford Diffraction, 2021) Numerical absorption correction based on gaussian integration over a multifaceted crystal model using spherical harmonics, implemented in SCALE3 ABSPACK scaling algorithm.
T_{min}, T_{max}	0.774, 1.0	0.910, 1.000	0.841, 1.000	0.659, 1.000
No. of measured, independent and observed [$I > 2\sigma(I)$] reflections	22173, 4504, 3636	48830, 5310, 4594	78910, 5506, 4910	23665, 5231, 4698
R_{int}	0.063	0.044	0.088	0.038
$(\sin \theta/\lambda)_{max}$ (Å^{-1})	0.598	0.628	0.628	0.628
Refinement				
$R[F^2 > 2\sigma(F^2)]$, $wR(F^2)$, S	0.066, 0.148, 1.00	0.048, 0.136, 1.07	0.038, 0.100, 1.06	0.038, 0.098, 1.06
No. of parameters	319	301	339	339
No. of restraints	0	0	0	0
H-atom treatment	H-atom parameters constrained	H-atom parameters constrained	H-atom parameters constrained	H-atom parameters constrained
$\Delta\rangle_{max}$, $\Delta\rangle_{min}$ (e Å^{-3})	0.68, -0.63	0.37, -0.30	0.28, -0.34	0.28, -0.37
Absolute structure	–	–	–	–
Absolute structure parameter	–	–	–	–
CCDC #				

Table S5 Crystallographic data and structural refinement parameters

	**SP10**	**SP13**	**SP14**
Crystal data			
Chemical formula	$C_{39}H_{39}NOS_2$	$C_{33}H_{30}N_2O_3S_2$	$C_{39}H_{38}N_2O_3S_2$
M_r	601.83	566.71	646.83
Crystal system, space group	Orthorhombic, $Pna2_1$	Triclinic, $P\bar{1}$	Orthorhombic, *Pnma*
Temperature (K)	100	101	100
a, *b*, *c* (Å)	17.5880 (1), 11.4351 (1), 32.1259 (2)	6.8419 (1), 13.1702 (2), 15.4840 (1)	11.0154 (2), 21.3534 (5), 14.4197 (3)
α, β, γ (°)	90, 90, 90	84.448 (1), 86.799 (1), 89.081 (1)	90, 90, 90
V (Å^3)	6461.18 (8)	1386.46 (3)	3391.75 (12)
Z	8	2	4
Radiation type	Cu $K\alpha$	Cu $K\alpha$	Cu $K\alpha$
μ (mm^{-1})	1.73	2.05	1.74
Crystal size (mm)	0.21 × 0.13 × 0.12	0.23 × 0.05 × 0.04	0.09 × 0.06 × 0.05
Data collection			
Diffractometer	XtaLAB Synergy, Single source at home/near, HyPix	XtaLAB Synergy, Single source at home/near, HyPix	XtaLAB Synergy, Single source at home/near, HyPix
Absorption correction	Multi-scan *CrysAlis PRO* 1.171.41.103a (Rigaku Oxford Diffraction, 2021) Empirical absorption correction using spherical harmonics, implemented in SCALE3 ABSPACK scaling algorithm.	Multi-scan *CrysAlis PRO* 1.171.41.103a (Rigaku Oxford Diffraction, 2021) Empirical absorption correction using spherical harmonics, implemented in SCALE3 ABSPACK scaling algorithm.	Multi-scan *CrysAlis PRO* 1.171.41.103a (Rigaku Oxford Diffraction, 2021) Empirical absorption correction using spherical harmonics, implemented in SCALE3 ABSPACK scaling algorithm.
T_{min}, T_{max}	0.687, 1.000	0.508, 1.000	0.784, 1.000
No. of measured, independent and observed [$I > 2\sigma(I)$] reflections	209337, 12942, 12610	46258, 5484, 5096	30366, 3545, 3177
R_{int}	0.066	0.050	0.051
$(\sin \theta/\lambda)_{max}$ (Å^{-1})	0.628	0.627	0.629
Refinement			
$R[F^2 > 2\sigma(F^2)]$, $wR(F^2)$, S	0.083, 0.215, 1.11	0.048, 0.121, 1.08	0.074, 0.150, 1.15
No. of parameters	802	366	240
No. of restraints	8	0	16
H-atom treatment	H-atom parameters constrained	H-atom parameters constrained	H-atom parameters constrained
$\Delta\rangle_{max}$, $\Delta\rangle_{min}$ (e Å^{-3})	1.10, -0.44	0.53, -0.35	0.31, -0.44
Absolute structure	Refined as an inversion twin.	–	–
Absolute structure parameter	0.47 (3)	–	–
CCDC #			

Switching

Solutions of the spiropyrans were made up in acetonitrile at concentrations approximately 10^{-5} mol L^{-1}. A drop of trifluoroacetic acid was added to the cuvette containing the dilute spiropyran solution. Excess triethylamine was added to the cuvette to revert to the spiropyran.

The ethynyl functionalised spiropyrans could not photochemically switched. Therefore, the acidchromism of the spiropyrans was investigated. Upon addition of trifluoroacetic acid (TFA) to a dilute solution of the spiropyrans results in a bright yellow solution. The addition of triethylamine (NEt_3) bleaches the solution (Figure S92 to Figure S94; Table S6). The six spiropyrans studied exhibited reversible switching of the colouration from TFA and bleaching using NEt_3.

The UV-vis results for the spiropyrans depict absorbance peaks characteristic for the SP and MCH^+. The compounds **4**, **5**, **SP1** and **SP5** show the π-π* transitions in the UV region typical of spiropyrans. The pyridyl species absorb at longer wavelengths than the thiophenyl derivatives, which in turn absorb at lower energies that the terminal alkynes. This is consistent with the number of delocalised π-bond in the molecules. In addition, the λ_{max} for the 7-substituted merocyanines are red-shifted compared to their 6-substituted analogues; however, this red-shifting is less pronounced for compounds **SP1** and **SP2**. This red shifting is predicted due to the higher conjugation between the two alkynyl moieties.

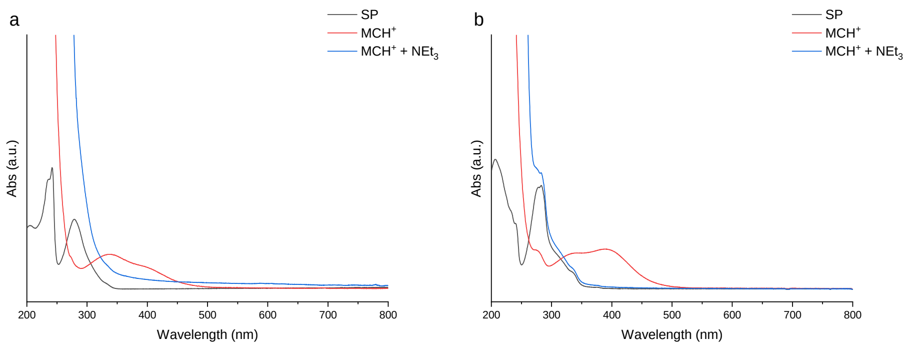

Figure S92 UV-vis spectra of **4** and **5** after the addition of TFA and NEt_3. Measurements recorded in acetonitrile.

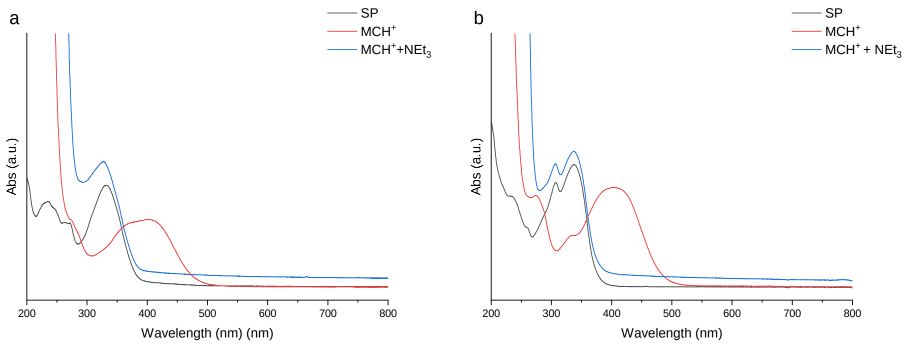

Figure S93 UV-vis spectra of **SP1** and **SP2** after the addition of TFA and NEt_3. Measurements recorded in acetonitrile

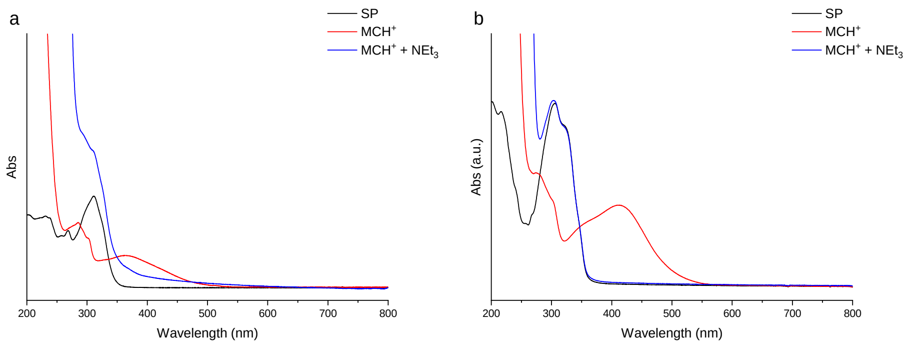

Figure S94 UV-vis spectra of **SP5** and **SP6** after the addition of TFA and NEt_3. Measurements recorded in acetonitrile

Table S6 UV-vis data for spiropyrans before and after the addition of TFA to give the MCH+ form. Measurements done in acetonitrile.

	Spiropyran		Merocyanine-H$^+$	
**Compound**	λ_{max}	ε (L mol-1 cm-1)	λ_{max}	ε (L mol-1 cm-1)
**4**	279	30,900	336	15,400
**5**	283	33,200	389	12,800
**SP1**	331	53,700	402	35,500
**SP2**	307	36,500	404	34,800
	338	42,700		
**SP5**	311	47,700	362	16,800
**SP6**	305	44,500	413	19,900

The switching on **SP8**, **SP9** and **SP14** using light and chemical stimuli were studied using UV-vis absorption spectroscopy. Figure S95, Figure S96 and Figure S97 show the absorption spectra of **SP8**, **SP9** and **SP14** in THF. The inset of the spectra show the absorption of the merocyanine after irradiation with UV light. The λ_{max} for mercyanine isomers of **SP8** and **SP9** appear at 450 and 460 nm, respectively.

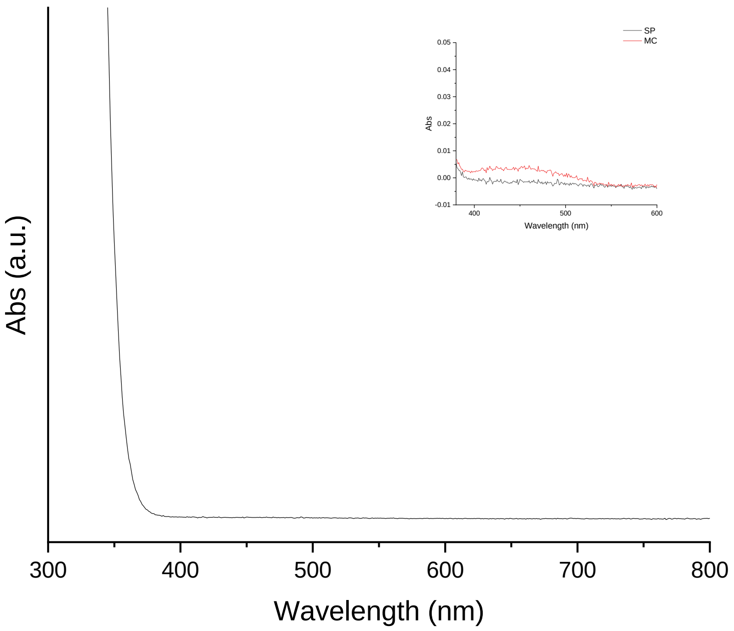

Figure S95 UV-vis absorption spectra of **SP8** in THF. Inset area shows isolated area of merocyanine absorption.

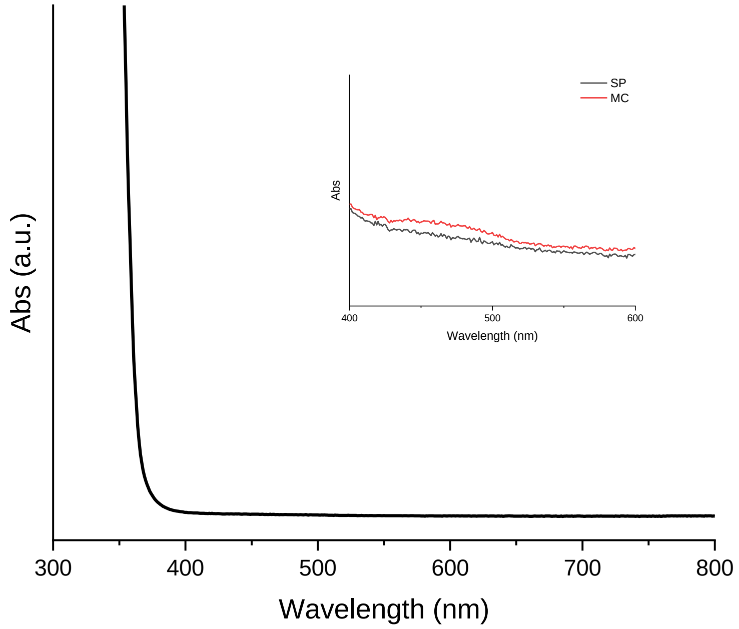

Figure S96 UV-vis absorption spectra of **SP9** in THF. Inset area shows isolated area of merocyanine absorption.

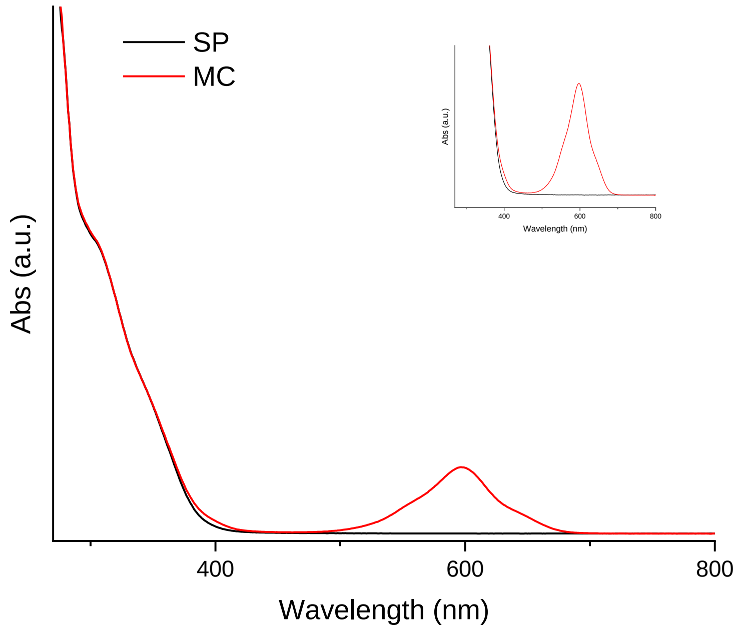

Figure S97 UV-vis absorption spectra of **SP14** in THF. Inset area shows isolated area of merocyanine absorption.

In mesitylene (common solvent for single-molecule junctions) the UV light switching of **SP8** and **SP9** was unable to be observed using UV-vis absorption spectroscopy. The fast thermal reversion of the merocyanine to spiropyran is the likely cause. Upon the addition of TFA, new absorptions at λ_{max} 398 and 466 nm appear for **SP8** and **SP9**, respectively (Figure S98 and S99). This band comes from the protonated *cis*-[MCH]$^+$. Upon exposure to UV light this band blue-shifts to λ_{max} 500 and 510 nm for **SP8** and **SP9** respectively. Isomerisation to the *trans*-[MCH]$^+$ accounts for this change in absorption. The addition of base recovers the initial absorption spectrum for both spiropyrans.

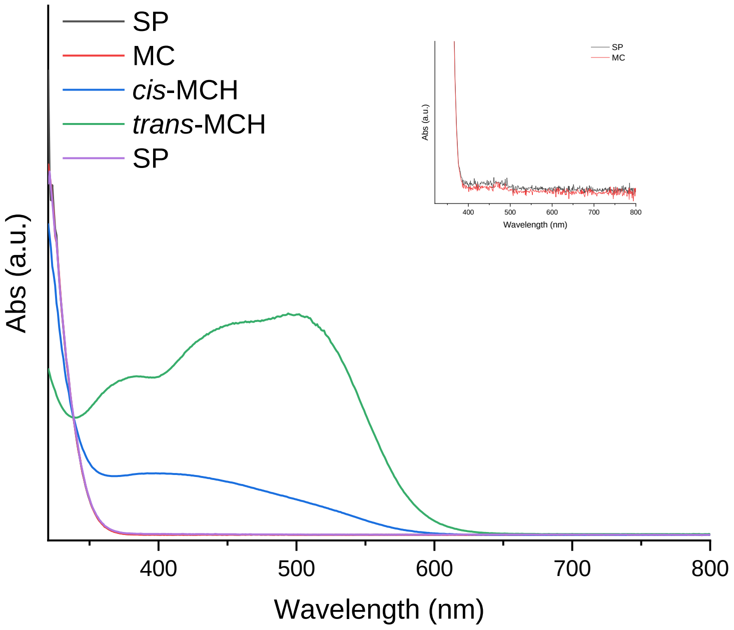

Figure S98 UV-vis absorption Spectra of **SP8** upon exposure to UV light, TFA, TFA and UV light, and base. All spectra are recorded in mesitylene with concentration of starting spiropyran at 1.2 x 10^{-4} mol L^{-1}.

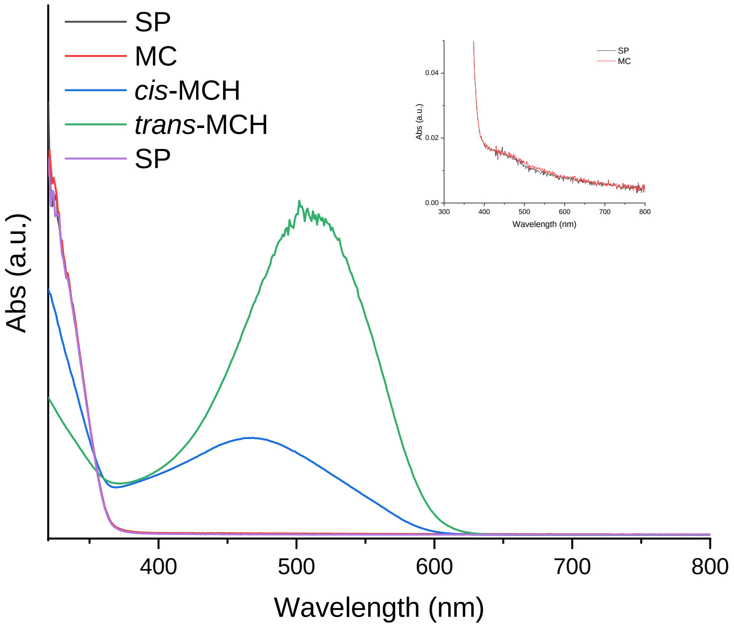

Figure S99 UV-vis absorption Spectra of **SP9** upon exposure to UV light, TFA, TFA and UV light, and base. All spectra are recorded in mesitylene with concentration of starting spiropyran at 8.4 x 10^{-5} mol L^{-1}.

Exposing a mesitylene solution of **SP14** to UV light coincides with a new absorption at λ_{max} 616 nm associated the open merocyanine (Figure S100). This band disappears over time due to thermal isomerisation of merocyanine to spiropyran and it can be accelerated with white light. The addition of TFA to the mesitylene solution causes a small shoulder to appear around λ_{max} 400 nm for the *cis*-MCH. Exposure of this solution to UV light gives a λ_{max} at 423 nm for the *trans*-MCH. The addition of base recovers the starting spiropyran absorption spectrum (Figure S100).

Additional ^{1}H NMR data for these switching processes are shown in Figures S101 to S104. Kinetic data for the thermal merocyanine to spiropyran reaction for **SP13** and **SP14** are shown in Table S7.

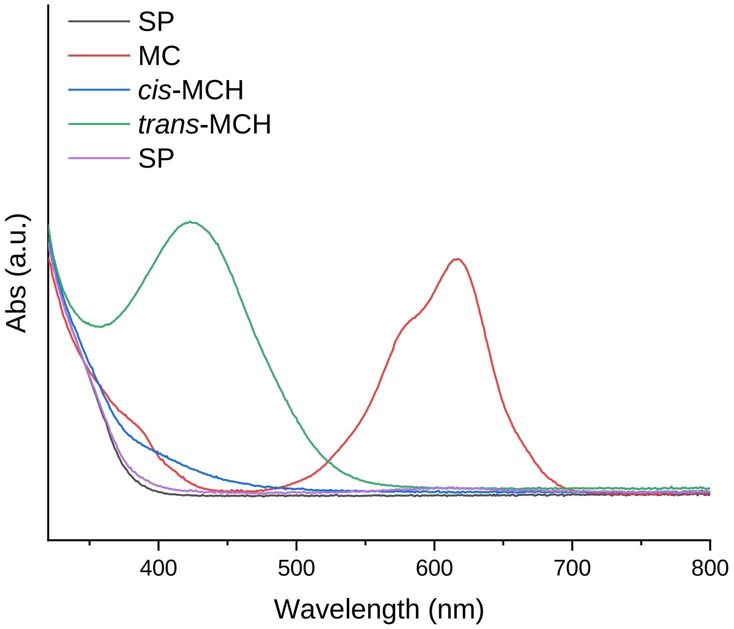

Figure S100 UV-vis absorption Spectra of **SP14** upon exposure to UV light, TFA, TFA and UV light, and base. All spectra are recorded in mesitylene with concentration of starting spiropyran at 3.7 x 10^{-5} mol L^{-1}.

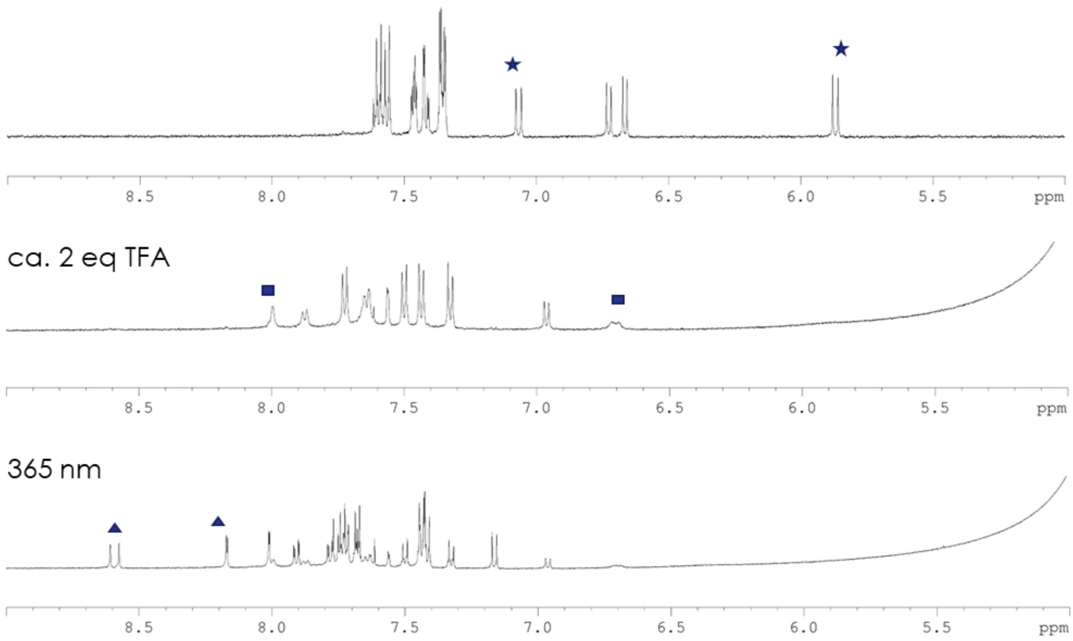

Figure S101 ^{1}H NMR of **SP8** upon exposure to TFA, and UV light. All spectra are recorded in CD_3CN. Stars depict SP, square depicts cis-MCH, and triangles depict trans-MCH.

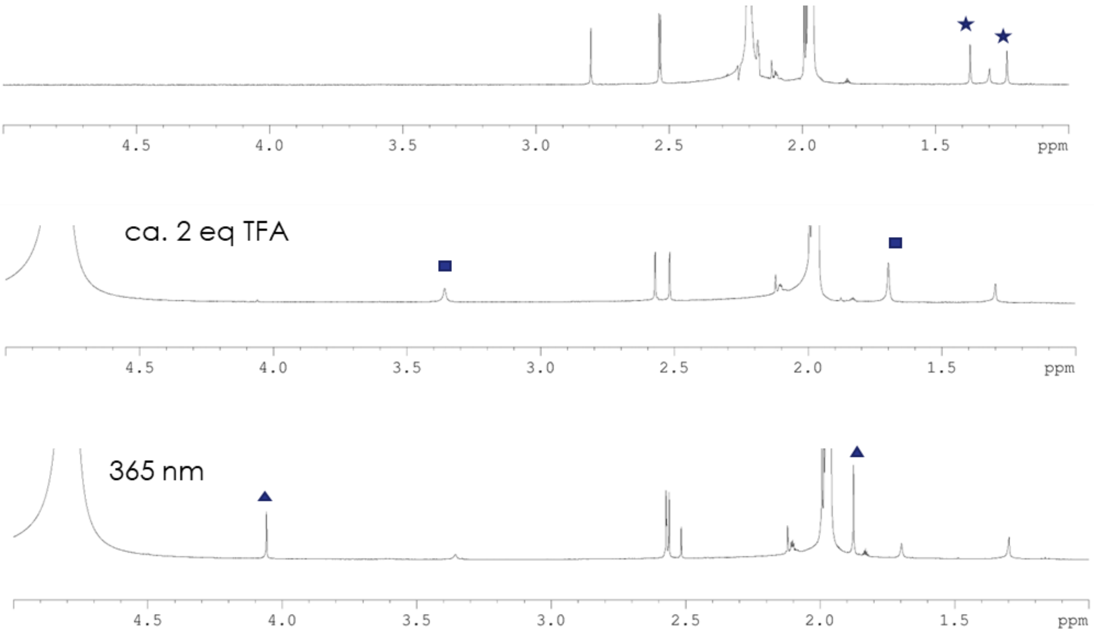

Figure S102 ^{1}H NMR of **SP8** upon exposure to TFA, and UV light. All spectra are recorded in CD_3CN. Stars depict SP, square depicts cis-MCH, and triangles depict trans-MCH.

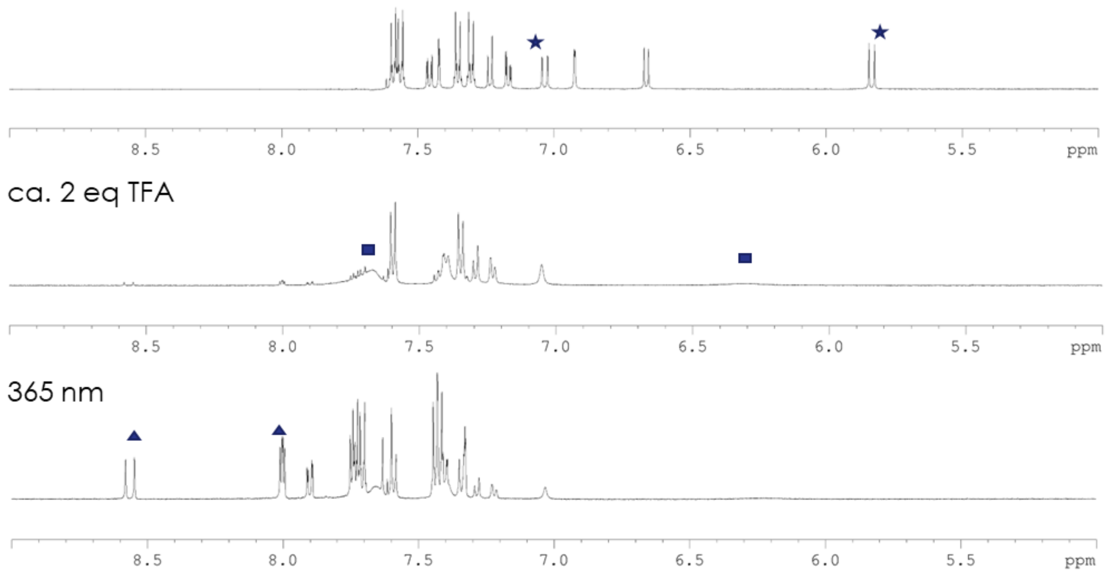

Figure S103 ^{1}H NMR of **SP9** upon exposure to TFA, and UV light. All spectra are recorded in CD_3CN. Stars depict SP, square depicts cis-MCH, and triangles depict trans-MCH.

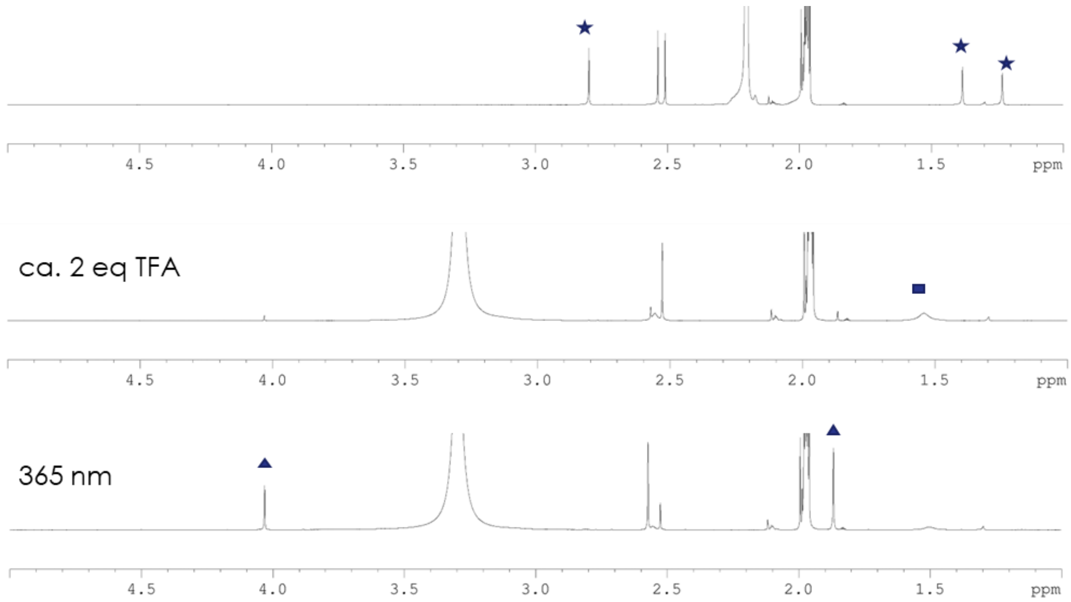

Figure S104 ^{1}H NMR of **SP9** upon exposure to TFA, and UV light. All spectra are recorded in CD_3CN. Stars depict SP, square depicts cis-MCH, and triangles depict trans-MCH.

Table S7 Kinetic Parameters for the thermal ring-closing of the merocyanine forms of **SP0** (**MC0**), **SP13** (**MC13**) and **SP14** (**MC14**) in THF at ambient temperature. Where **SP0** is 1′,3′,3′-trimethyl-6-nitrospiro[chromene-2,2′-indoline].

Compound	λmax	k (s^{-1})	Half-life (s)
**MC0**	586	0.02162(0.00104)	32.1(1.5)
**MC13**	597	0.07417(0.00104)	9.35(0.13)
**MC14**	595	0.0747	9.28

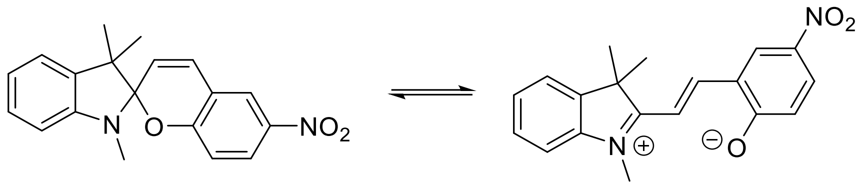

SP0 **MC0**

Single-molecule Conductance

Table S8. Summary of conductance data for the studied spiropyrans

Generation	Spiropyran	Conductance [G_0]	Break-off Distance (95% C.I) [nm]
	4	$10^{-4.53\pm0.94}$	0.80
	6	$10^{-4.17\pm0.70}$	1.38
	7	$10^{-4.16\pm0.71}$	0.31
**G1**	SP1	$10^{-3.44\pm0.31}$	0.18
		$10^{-4.69\pm0.60}$	0.42
	SP1[b]	$10^{-2.64\pm0.36}$	0.39
		$10^{-4.14\pm0.94}$	1.03
	SP2	$10^{-4.80\pm0.64}$	0.49
	SP3	Not detected	-
	SP5	$10^{-2.17\pm0.40}$	0.12
		$10^{-3.99\pm0.56}$	0.38
	SP5[a]	$10^{-2.22\pm0.38}$	0.12
		$10^{-4.13\pm0.51}$	0.39
	SP5[b]	$10^{-2.37\pm0.41}$	0.15
		$10^{-4.78\pm1.02}$	0.41
	SP5[e]	$10^{-4.15\pm0.61}$	0.41
**G2**	SP8[a]	Not detected	
	SP9[a]	Not detected	
**G3**	SP12[b]	Not detected	

SP13[b]	$10^{-4.93\pm0.58}$	0.52
SP13[c]	$10^{-4.84\pm0.55}$	0.50
SP14[c]	$10^{-4.51\pm0.50}$	0.48
SP14[c,d]	$10^{-4.08\pm0.56}$	0.44
SP14[c,e]	$10^{-4.22\pm0.51}$	0.46

All conductance values are recorded at 100 mV bias except where otherwise stated. Break-off distances are calculated as 95th percentile of junction extension and are unadjusted for snap-back. The 95th percentile was calculated by extracting the electrode separation profile from the 2D density map in the range G±σ, and fitting it to a Gaussian distribution. [a]200 mV bias, [b]300 mV bias, [c]500 mV bias, [d]after UV irradiation, [e]upon addition of TFA.

*Single-Molecule Conductance Data for compounds **4**, **6** and **7**.*

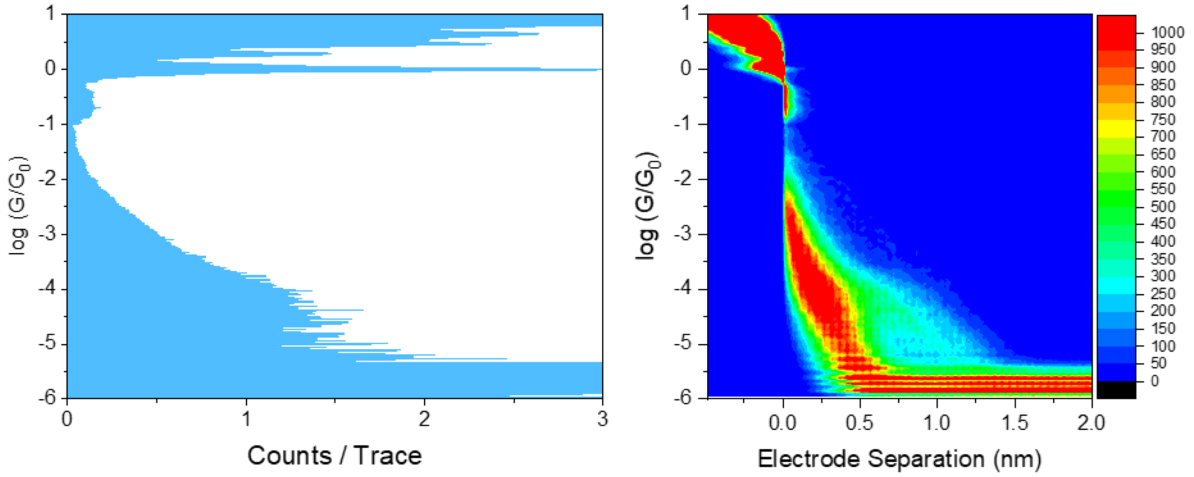

Figure S105 Conductance of **4** (100 mV)

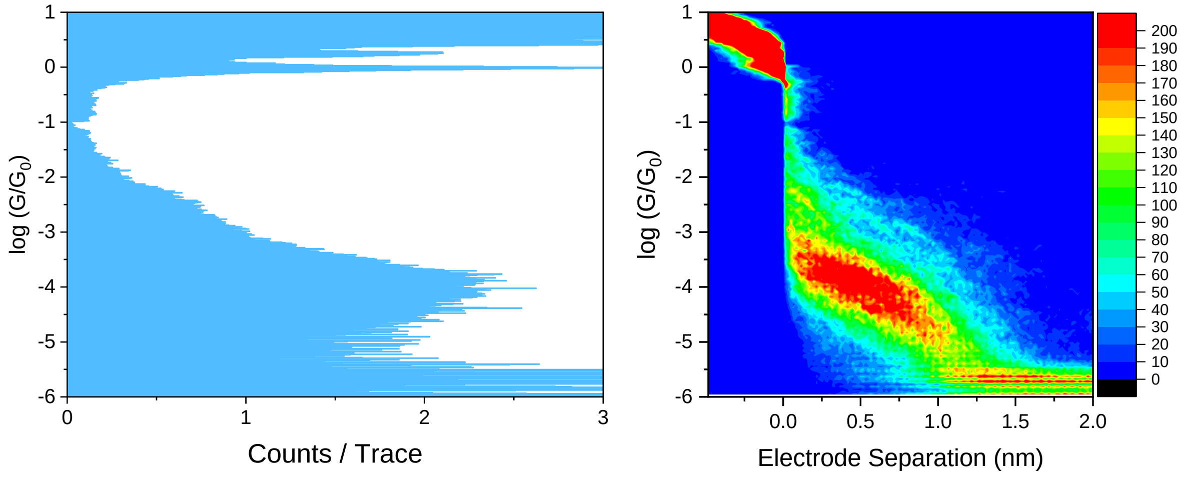

Figure S106 Conductance of **6** (100 mV)

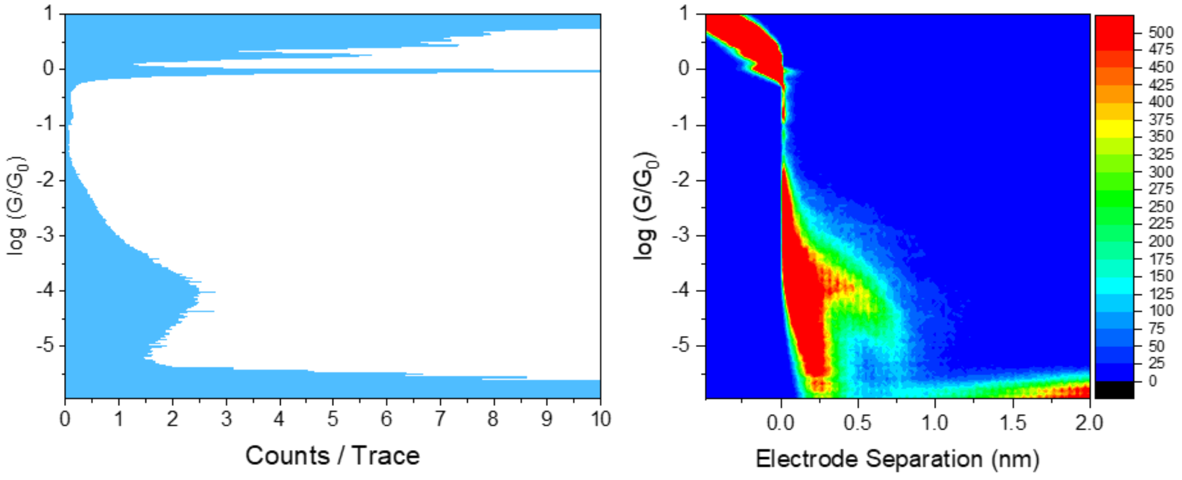

Figure S107 Conductance of **7** (100 mV)

Single-Molecule Conductance Data for ***G1*** *compounds*

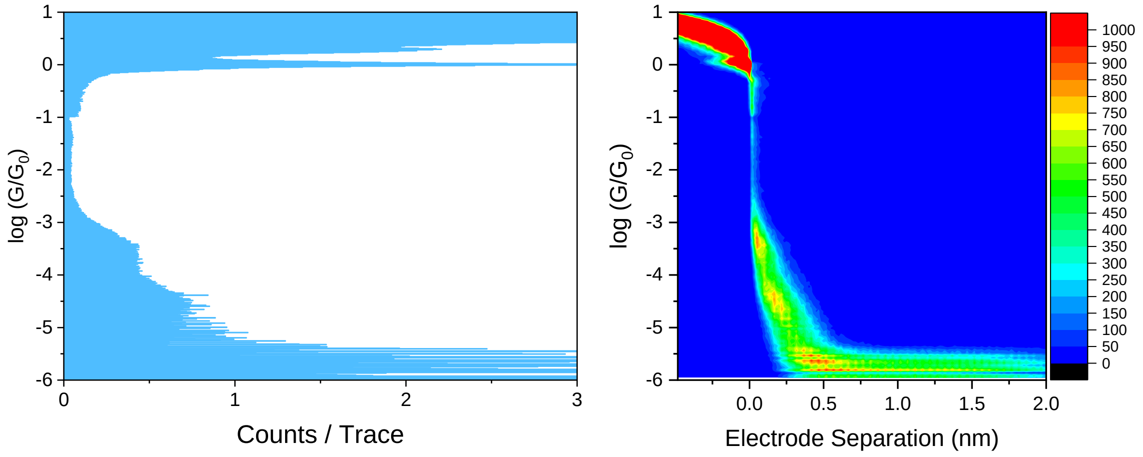

Figure S108 Conductance of **SP1** (100 mV)

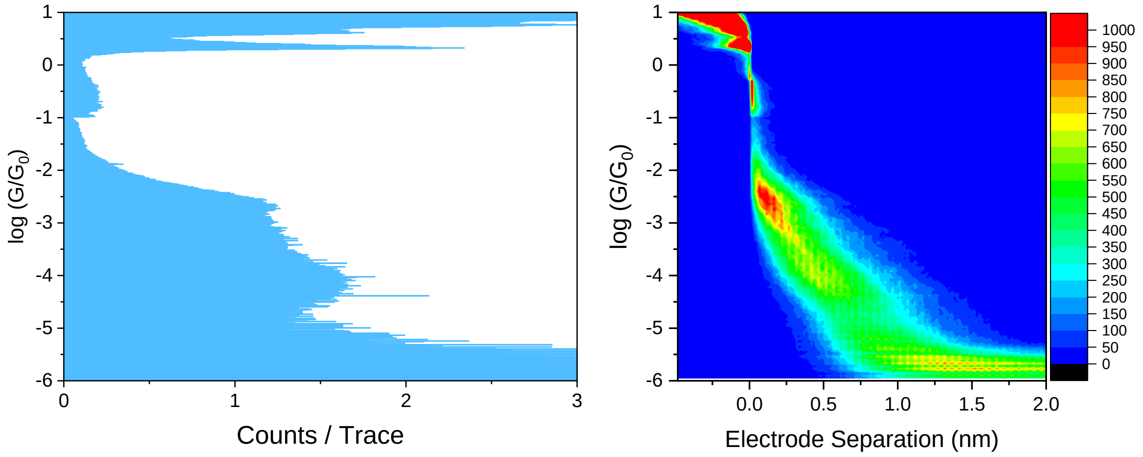

Figure S109 Conductance of **SP1** (300 mV)

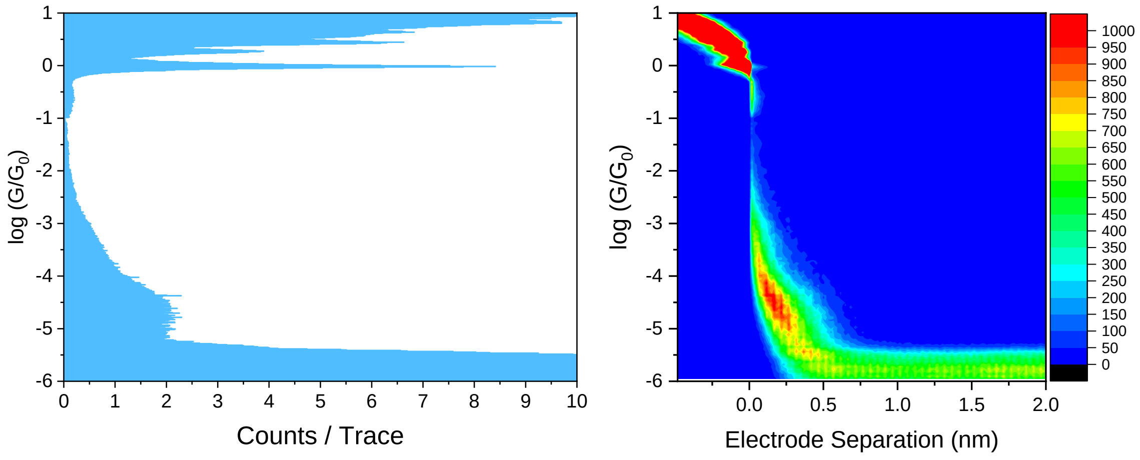

Figure S110 Conductance of **SP2** (100 mV)

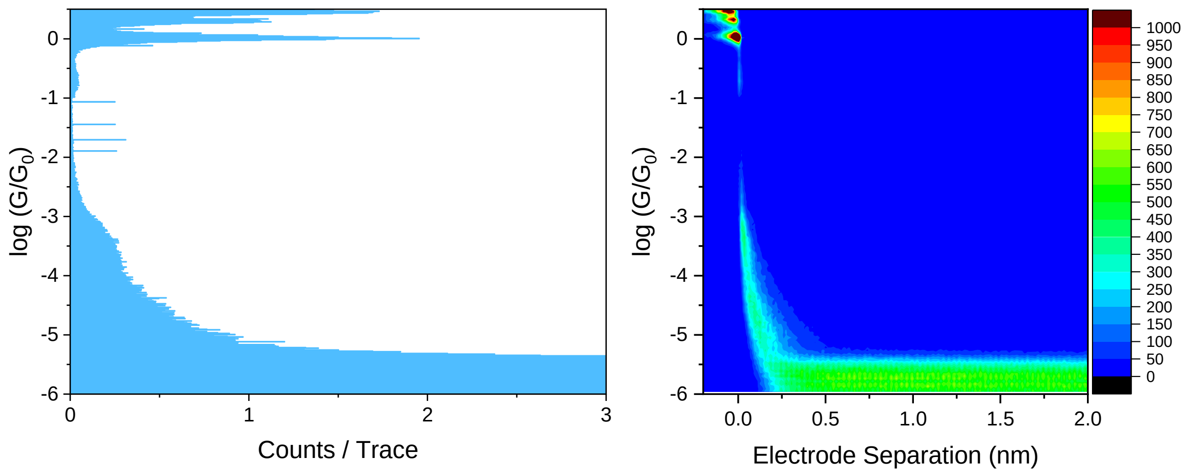

Figure S111 Conductance of **SP3** (100 mV)

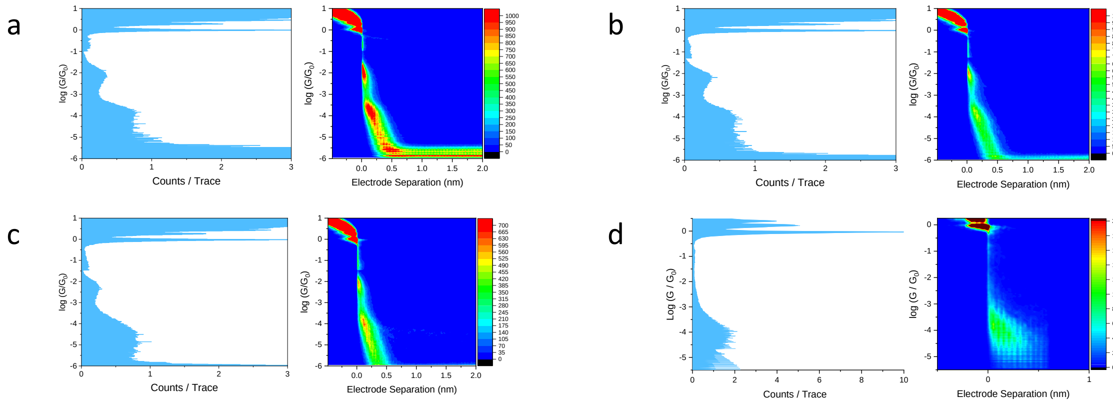

Figure S112 SP5 at a. 100 mV, b. 200 mV, c. 300 mV and d. after the addition of TFA

*Single-Molecule Conductance Data for **G2** compounds*

All **G2** compounds failed to produce discernible features in the histograms and 2D plots. In contrast with **G1** compounds, no evidence of binding through the central spiropyran unit could be observed, most probably due to the steric bulk provided by the aryl contact placed directly on the spiropyran scaffold (*i.e.* without the alkynyl "spacers"). Here we reproduce representative examples from **SP8** (5',6-substituted) and **SP9** (5',7-substituted). The elongated tunnelling signal and raised counts at low conductance suggests successful self-assembly of the molecule in the junction, but failure to settle on a plateau with conductance above our noise floor of approximately $10^{-5.5}$ G_0.

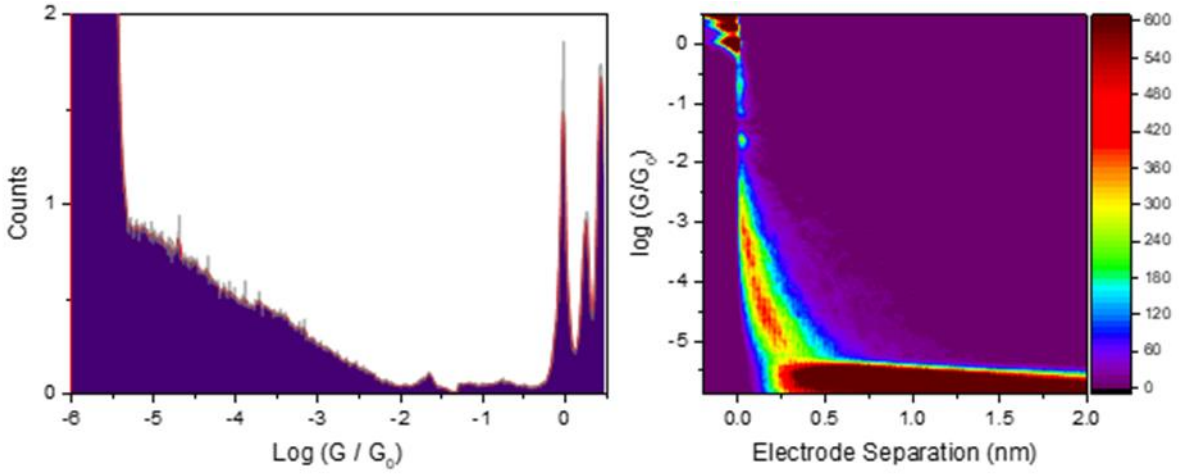

Figure S113 Conductance of **SP8** (300 mV)

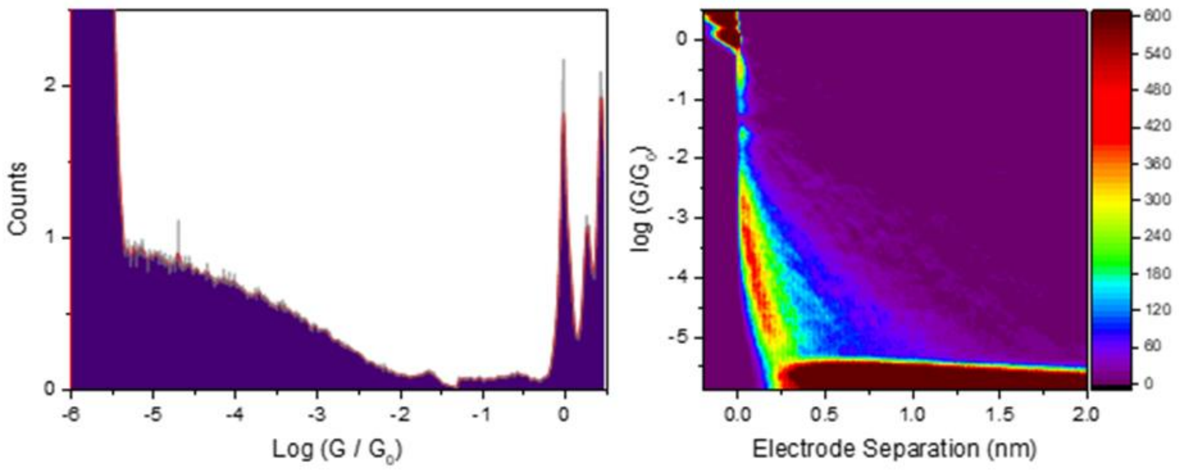

Figure S114 Conductance of **SP9** (300 mV)

*Single-Molecule Conductance Data for **G3** compounds*

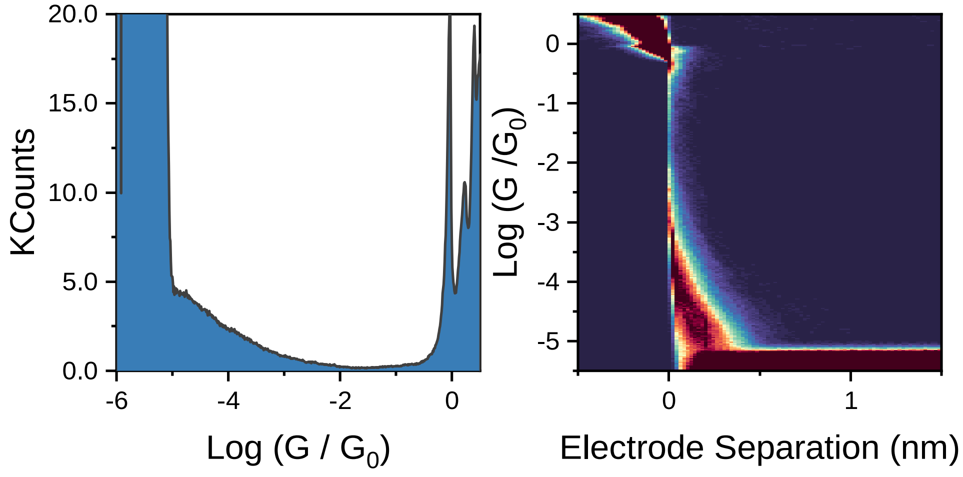

Figure S115 Conductance of **SP12** (500 mV)

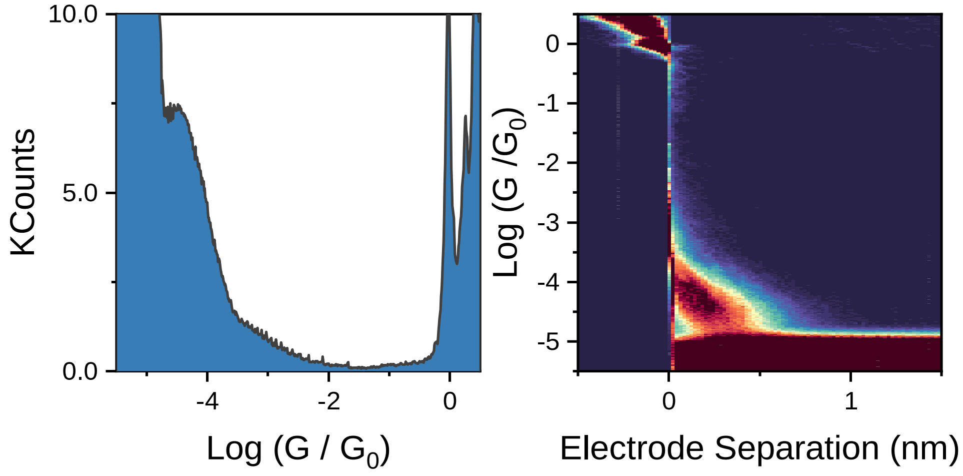

Figure S116 Conductance of **SP13** (300 mV)

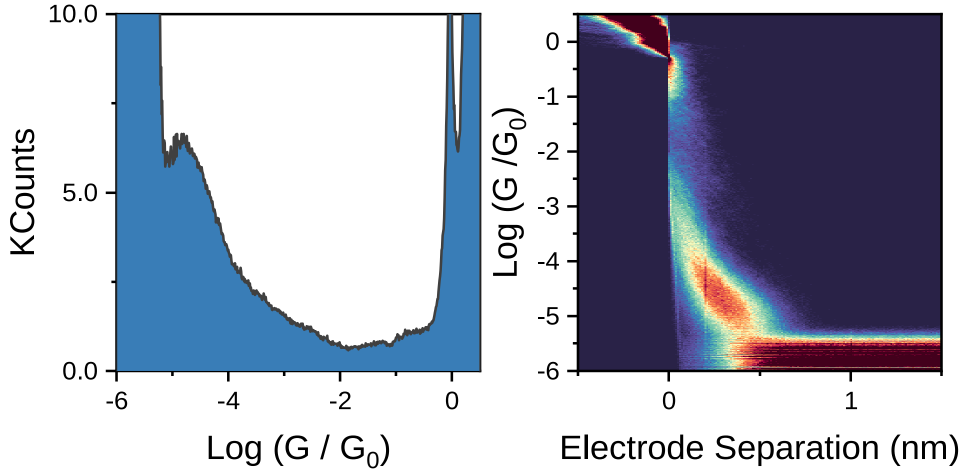

Figure S117 Conductance of **SP13** (500 mV)

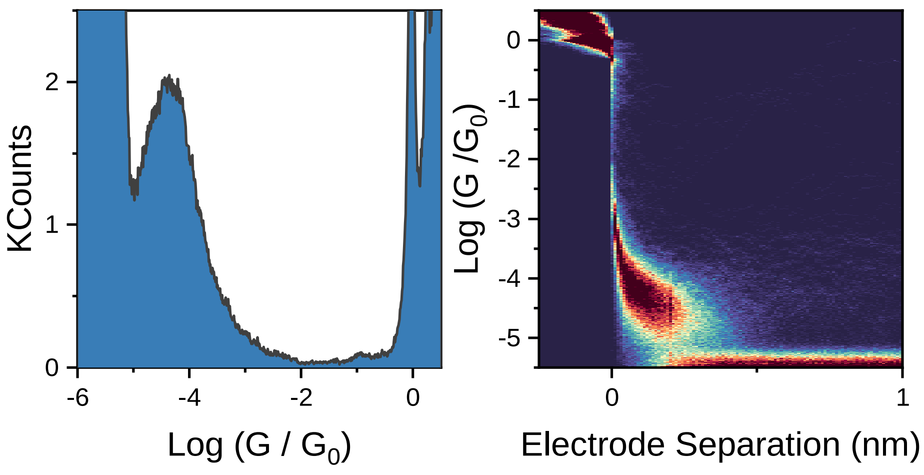

Figure S118 Conductance of **SP14** (500 mV)

Additional DFT-NEGF Details

DFT Methods

The optimized geometry and ground state Hamiltonian and overlap matrix elements of each structure was self-consistently obtained using the SIESTA[33] implementation of density functional theory (DFT). SIESTA employs norm-conserving pseudo-potentials to account for the core electrons and linear combinations of atomic orbitals to construct the valence states. The local density approximation (GGA) of the exchange and correlation functional is used with PBE parameterization, a double-ζ polarized (DZP) basis set, a real-space grid defined with an equivalent energy cut-off of 250 Ry. The geometry optimization for each structure is performed to the forces smaller than 10 meV/Å.

The mean-field Hamiltonian obtained from the converged DFT calculation was combined with the GOLLUM[34] implementation of the non-equilibrium Green's function method to calculate the phase-coherent, elastic scattering properties of the each system consist of left gold (source) and right gold (drain) leads and the scattering region. The transmission coefficient *T(E)* for electrons of energy *E* (passing from the source to the drain) is calculated via the relation: $T(E) = Trace(\Gamma_R(E)G^R(E)\Gamma_L(E)G^{R\dagger}(E))$. In this expression, $\Gamma_{L,R}(E) = i\left(\sum_{L,R}(E) - \sum_{L,R}{}^{\dagger}(E)\right)$ describe the level broadening due to the coupling between left (L) and right (R) electrodes and the central scattering region, $\sum_{L,R}(E)$ are the retarded self-

energies associated with this coupling and $G^R = (ES - H - \sum_L - \sum_R)^{-1}$ is the retarded Green's function.

The electrical conductance is then calculated using the Landauer formula $G(E_F,T) = G_0 L_0$, $L_n = \int_{-\infty}^{+\infty} dE\ (E - E_F)^n\ T(E)(-\partial f(E,T,E_F)/\partial E)$ and $f = (e^{(E-E_F)/k_B T} + 1)^{-1}$ is the Fermi-Dirac probability distribution function, T is the temperature, E_F is the Fermi energy, $G_0 = 2e^2/h$ is the conductance quantum, e is electron charge and h is the Planck's constant.

Data analysis and theoretical conductance histograms:

First, we form a series of junctions with different contacting configuration to electrodes and calculate the electrical conductance G for a range of electrodes Fermi energies. Next, we create the conductance histograms using the calculated conductance for each junction and for a wide range of EF between the HOMO-LUMO gap. To ensure including conductance values in co-tunnelling regime, the range of EF is chosen such that they do not include HOMO and LUMO resonances. The peaks in conductance histograms are fitted with a log-normal distribution and their centre is defined as the most probable conductance.

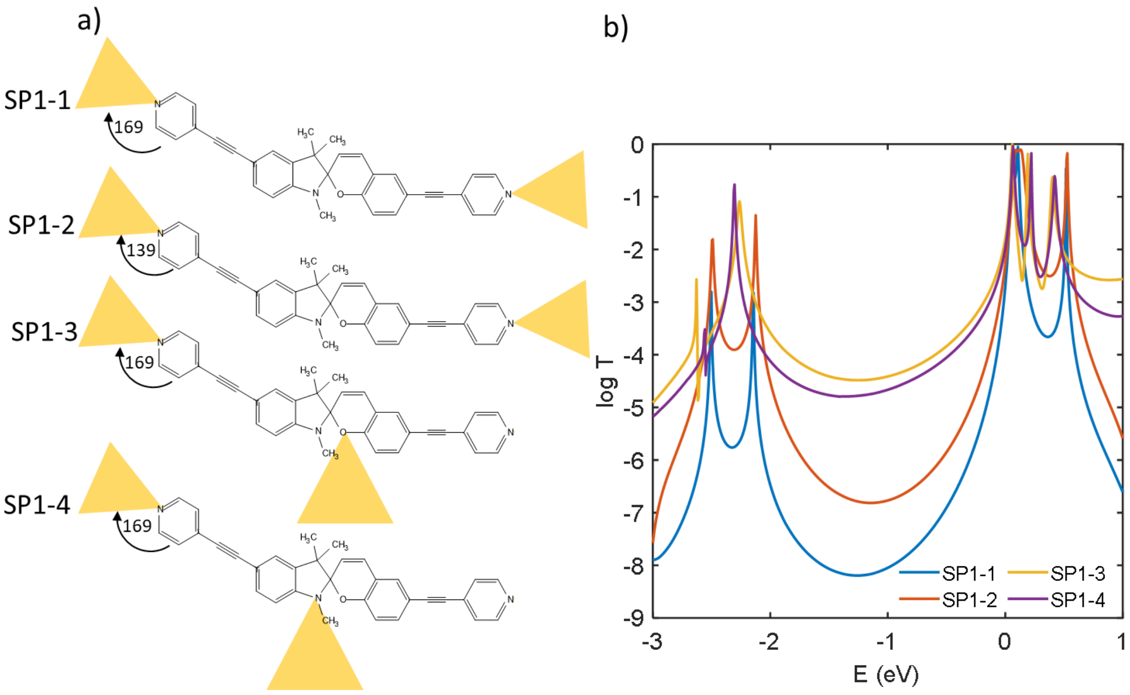

Figure S119: Transport properties of molecular junctions. **(a)** Structures of the molecular wires first generation spiropyran system terminated with 4-pyridyl (**SP1**) contact groups. **(b)** Transmission coefficient *T*(*E*) for structures of molecules **(a).** *E* = 0 eV shows DFT Fermi energy (E_F).

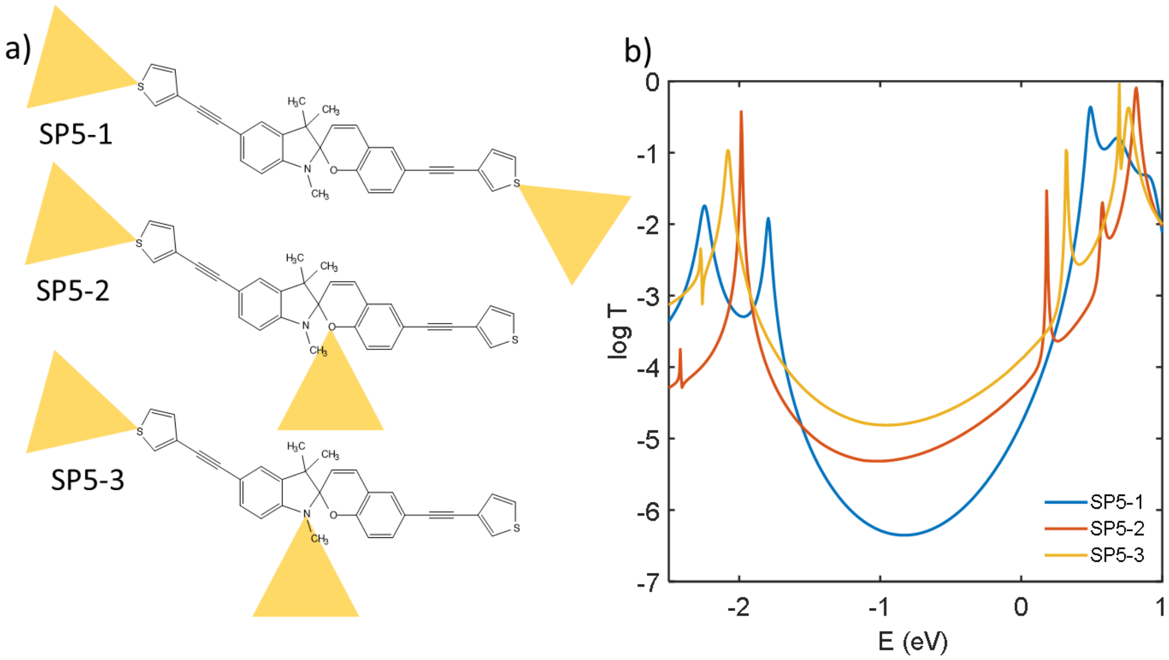

Figure S120: Transport properties of molecular junctions. **(a)** Structures of the molecular wires first generation spiropyran system terminated with thienyl (**SP5**) contact groups. **(b)** Transmission coefficient *T*(*E*) for structures of molecules (a). *E* = 0 eV shows DFT Fermi energy (E_F).

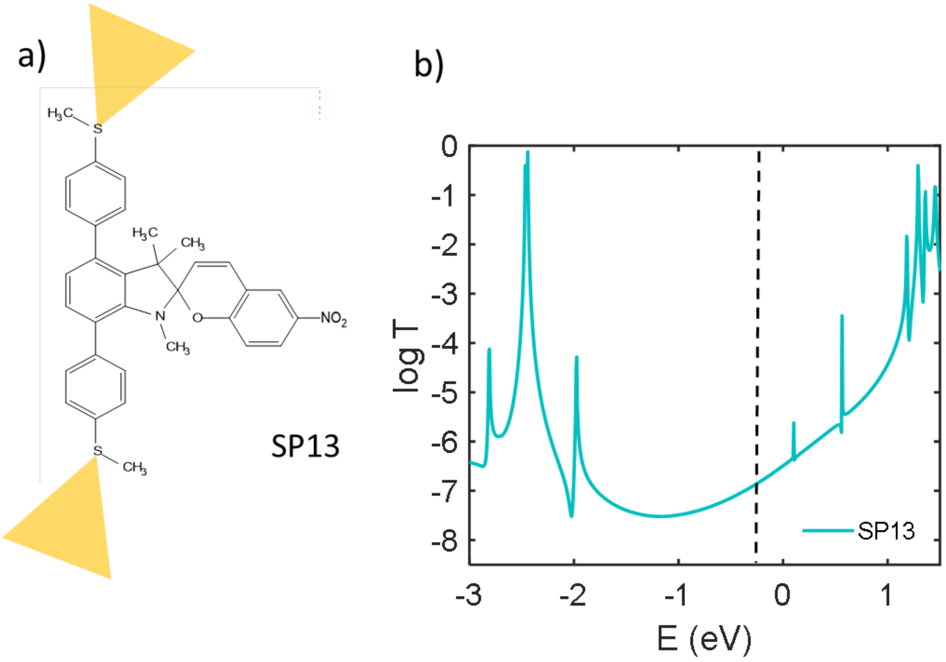

Figure S121: Transport properties of molecular junctions. **(a)** Structures of the molecular wires third generation spiropyran system terminated with 4-thioanisolyl (**SP13**) contact groups. **(b)** Transmission coefficient *T*(*E*) for structures of molecules **(a).** *E* = 0 eV shows DFT Fermi energy (E_F).

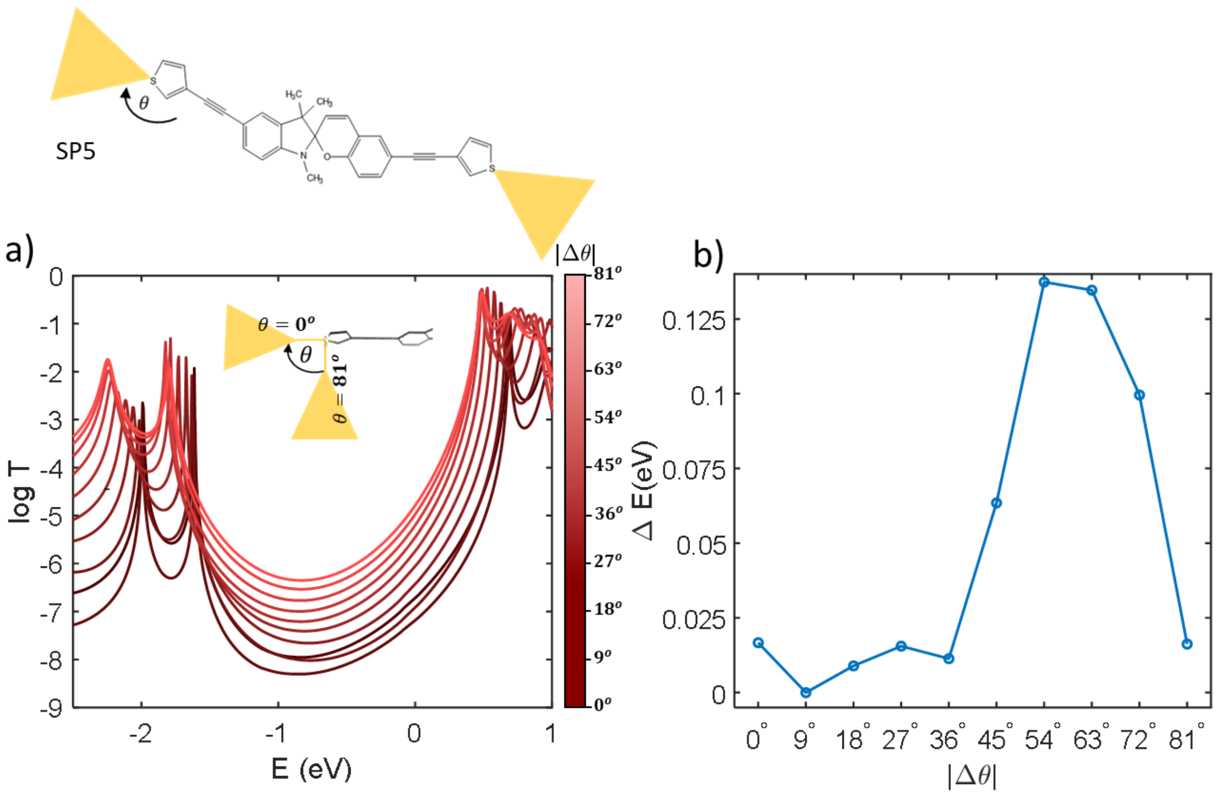

Figure S122: Transport properties of molecular junctions. **(a)** Transmission coefficient T(E) for structures of **SP5** for a range of configurations with different dihedral angles ($|\Delta\theta| = 9^o$). **(b)** Total energy differences for configurations **(a).** *E* = 0 eV shows DFT Fermi energy (E_F).

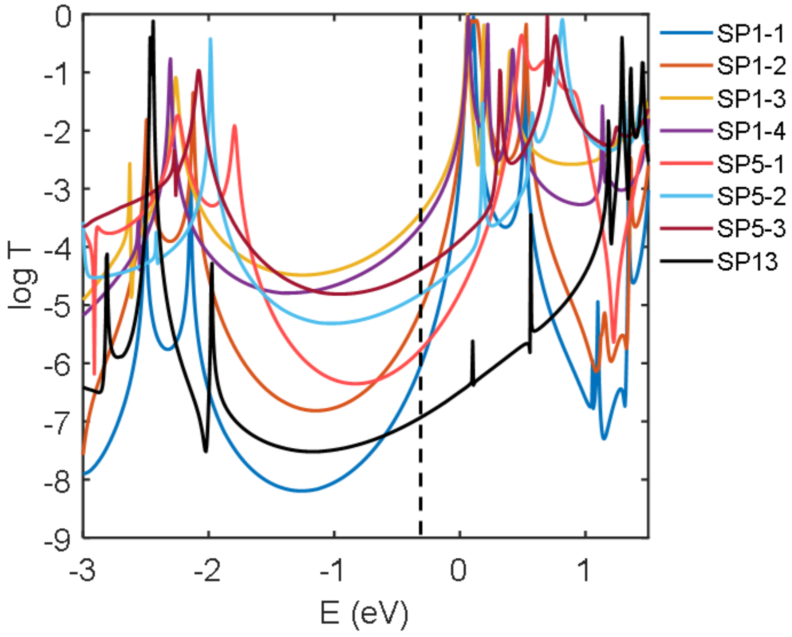

Figure S123: Transmission coefficient T(E) for different structures of **SP1**,**SP5** and **SP13**. *E* = 0 eV shows DFT Fermi energy (E_F).

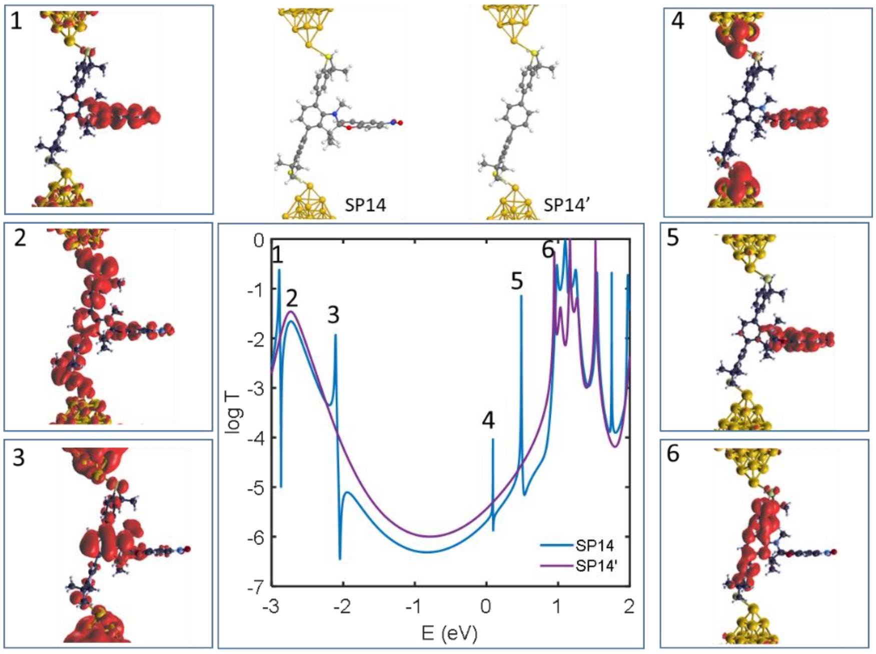

Figure S124: DFT transmission coefficient for **SP14** and **SP14**′ between gold electrodes, where SP14′ is backbone of **SP14** (no side group). In each subfigure, the top panel shows the local density of state around resonance (numbers shown in transmission plots). *E* = 0 eV shows DFT Fermi energy (E_F).

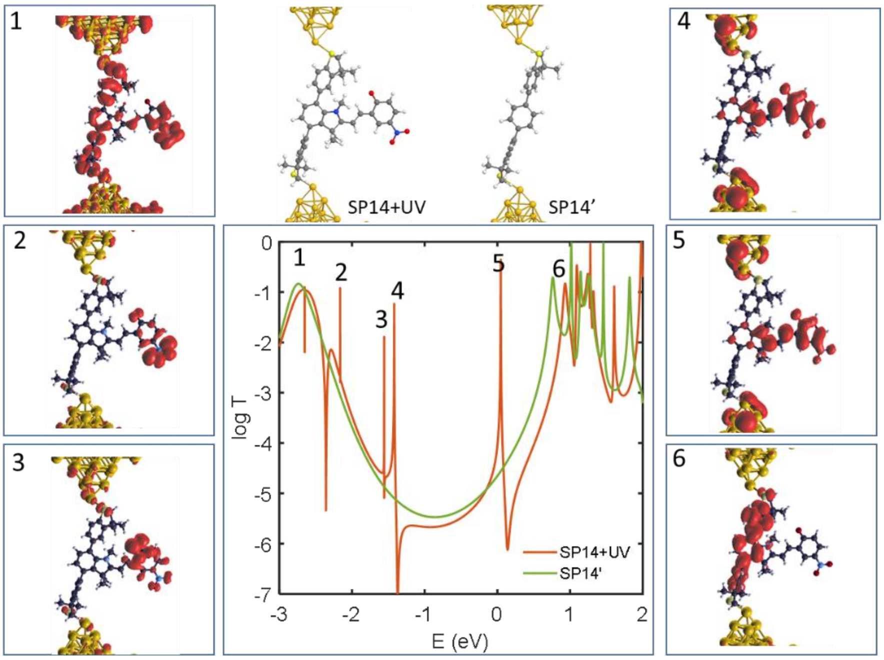

Figure S125: DFT transmission coefficient for **SP14**+UV and **SP14**′ between gold electrodes, where **SP14**′ is backbone of **SP14**+UV (no side group). In each subfigure, the top panel shows the local density of state around resonance (numbers shown in transmission plots). *E* = 0 eV shows DFT Fermi energy (E_F).

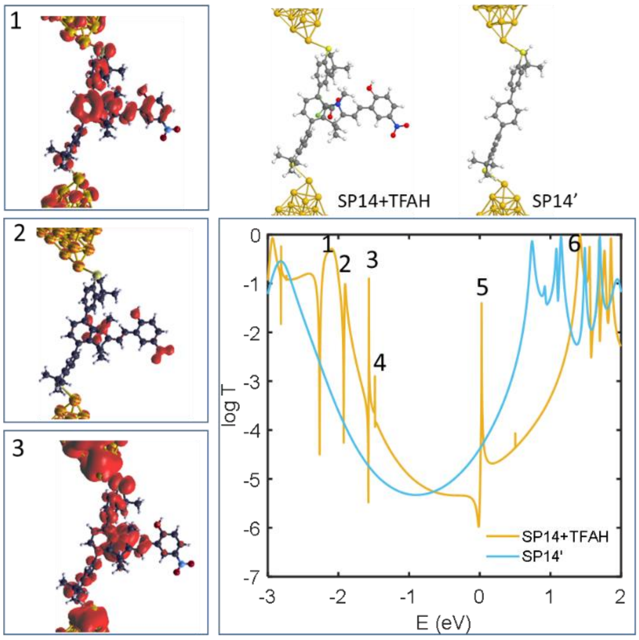

Figure S126: DFT transmission coefficient for **SP14**+TFAH and **SP14**’ between gold electrodes, where **SP14**’ is backbone of TFAH (no side group). In each subfigure, the top panel shows the local density of state around resonance (numbers shown in transmission plots). E = 0 eV shows DFT Fermi energy (E_F).

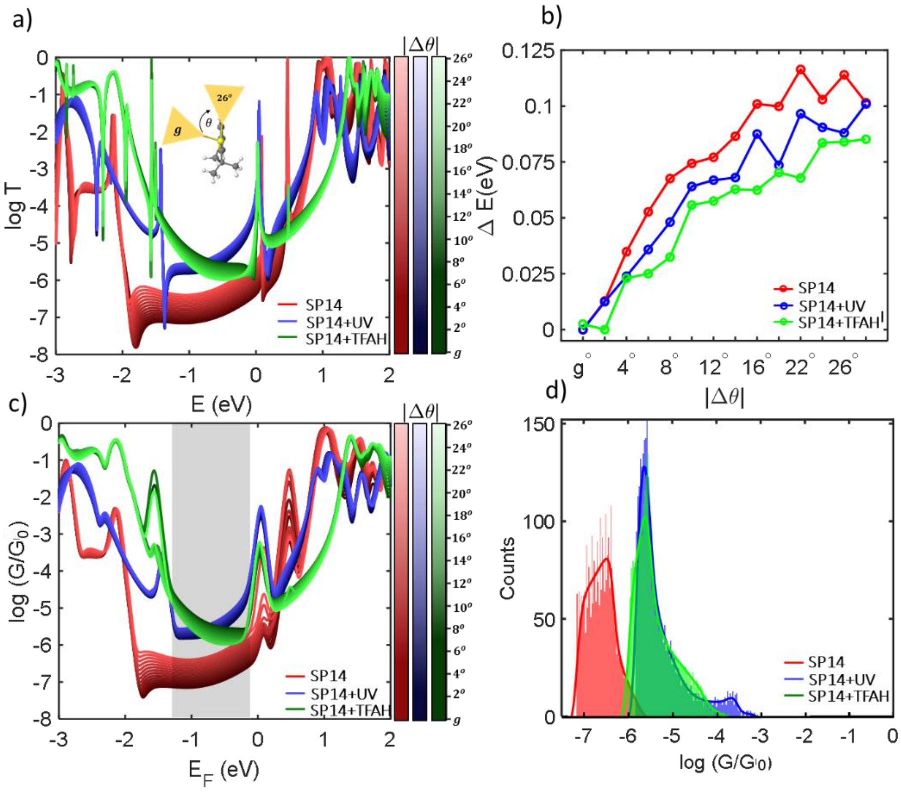

Figure S127: (a). Transmission coefficients for **SP14, SP14+UV, SP14+TFAH. (b)** Total energy differences for range of configurations with different dihedral angles ($|\Delta\theta| = 2^o$). **(c)** Electrical conductance for a range of configurations with different dihedral angles**. (d)** Calculated room

temperature conductance histograms over a range of molecule-electrode dihedral angles. E = 0 eV shows DFT Fermi energy (E_F).

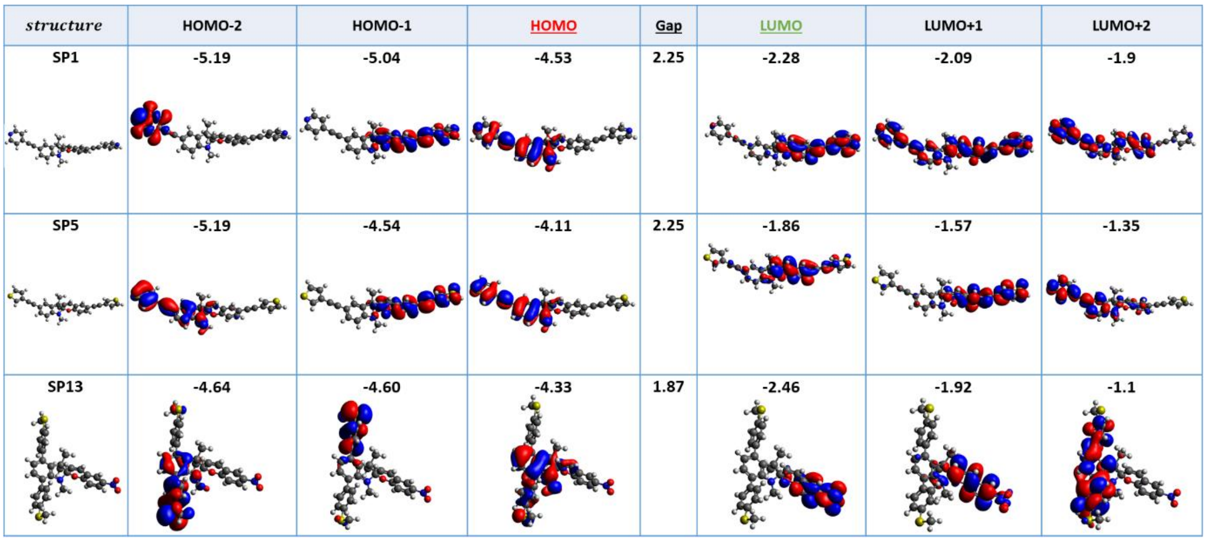

Figure S128: Frontier orbital table for the gas-phase structures

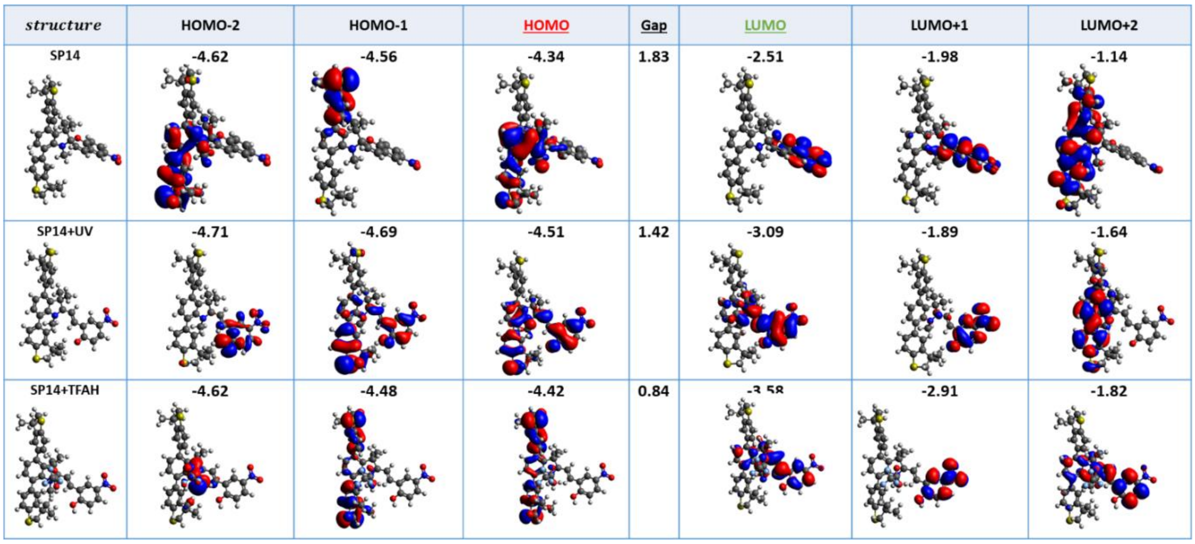

Figure S129: Frontier orbital table for the gas-phase structures

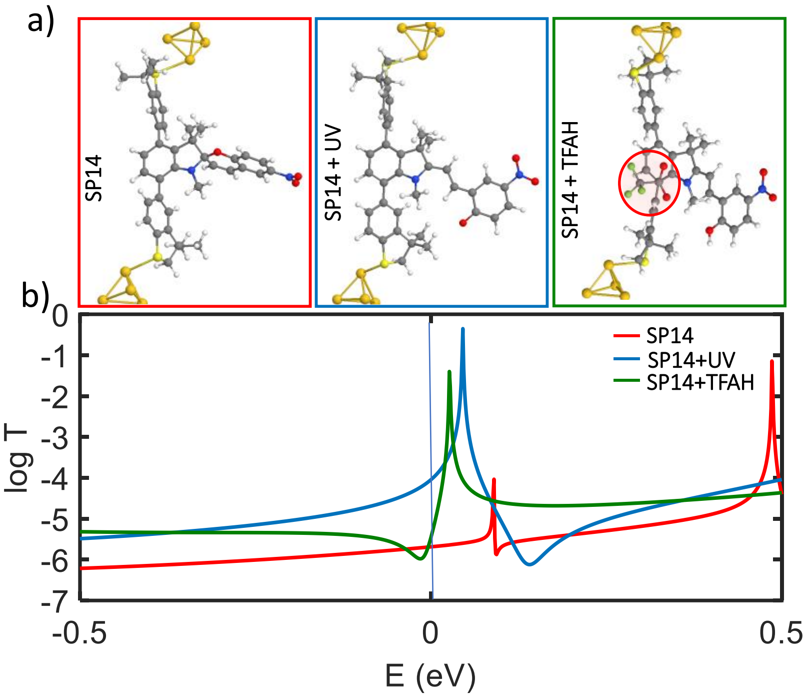

Figure S130: (a) Relaxed structure of SP14, SP14+UV and SP14+TFAH junctions. **(b)** Transmission coefficient for the corresponding junctions.

References

[1] (a) T. A. Su, M. Neupane, M. L. Steigerwald, L. Venkataraman, C. Nuckolls, *Nat. Rev. Mater.* **2016**, *1*, 16002; (b) N. Xin, J. Guan, C. Zhou, X. Chen, C. Gu, Y. Li, M. A. Ratner, A. Nitzan, J. F. Stoddart, X. Guo, *Nat. Rev. Phys.* **2019**, *1*, 211-230.

[2] E. J. Dell, B. Capozzi, J. Xia, L. Venkataraman, L. M. Campos, *Nat. Chem.* **2015**, *7*, 209-214.

[3] (a) V. Kaliginedi, A. V. Rudnev, P. Moreno-García, M. Baghernejad, C. Huang, W. Hong, T. Wandlowski, *Phys. Chem. Chem. Phys.* **2014**, *16*, 23529-23539; (b) J. Liu, X. Zhao, Q. Al-Galiby, X. Huang, J. Zheng, R. Li, C. Huang, Y. Yang, J. Shi, D. Z. Manrique, C. J. Lambert, M. R. Bryce, W. Hong, *Angew. Chem. Int. Ed.* **2017**, *56*, 13061-13065; (c) P. Moreno-García, M. Gulcur, D. Z. Manrique, T. Pope, W. Hong, V. Kaliginedi, C. Huang, A. S. Batsanov, M. R. Bryce, C. Lambert, T. Wandlowski, *J. Am. Chem. Soc.* **2013**, *135*, 12228-12240; (d) H. Ozawa, M. Baghernejad, O. A. Al-Owaedi, V. Kaliginedi, T. Nagashima, J. Ferrer, T. Wandlowski, V. M. García-Suárez, P. Broekmann, C. J. Lambert, M. Haga, *Chem. Eur. J.* **2016**, *22*, 12732-12740; (e) Y. Li, M. Baghernejad, Q. Al-Galiby, D. Z. Manrique, G. Zhang, J. Hamill, Y. Fu, P. Broekmann, W. Hong, T. Wandlowski, D. Zhang, C. Lambert, *Angew. Chem. Int. Ed.* **2015**, *54*, 13586-13589; (f) F. Yan, F. Chen, X.-H. Wu, J. Luo, X.-S. Zhou, J. R. Horsley, A. D. Abell, J. Yu, S. Jin, B.-W. Mao, *J. Phys. Chem. C* **2020**, *124*, 8496-8503.

[4] (a) M. Naher, D. C. Milan, O. A. Al-Owaedi, I. J. Planje, S. Bock, J. Hurtado-Gallego, P. Bastante, Z. M. Abd Dawood, L. Rincón-García, G. Rubio-Bollinger, S. J. Higgins, N. Agraït, C. J. Lambert, R. J. Nichols, P. J. Low, *J. Am. Chem. Soc.* **2021**, *143*, 3817-3829; (b) M. Naher, S. Bock, Z. M. Langtry, K. M. O'Malley, A. N. Sobolev, B. W. Skelton, M. Korb, P. J. Low, *Organometallics* **2020**, *39*, 4667-4687.

[5] W. Hong, D. Z. Manrique, P. Moreno-García, M. Gulcur, A. Mishchenko, C. J. Lambert, M. R. Bryce, T. Wandlowski, *J. Am. Chem. Soc.* **2012**, *134*, 2292-2304.

[6] (a) C. K. Lee, D. A. Davis, S. R. White, J. S. Moore, N. R. Sottos, P. V. Braun, *J. Am. Chem. Soc.* **2010**, *132*, 16107-16111; (b) M. C. Walkey, L. T. Byrne, M. J. Piggott, P. J. Low, G. A. Koutsantonis, *Dalton Trans.* **2015**, *44*, 8812-8815; (c) M. C. Walkey, C. R. Peiris, S. Ciampi, A. C. Aragonès, R. B. Domínguez-Espíndola, D. Jago, T. Pulbrook, B. W. Skelton, A. N. Sobolev, I. Díez Pérez, M. J. Piggott, G. A. Koutsantonis, N. Darwish, *ACS Appl. Mater. Interfaces* **2019**, *11*, 36886-36894.

[7] S. Bhattacharyya, M. Maity, A. Chowdhury, M. L. Saha, S. K. Panja, P. J. Stang, P. S. Mukherjee, *Inorg. Chem.* **2020**, *59*, 2083-2091.

[8] K. Wagner, M. Zanoni, A. B. S. Elliott, P. Wagner, R. Byrne, L. E. Florea, D. Diamond, K. C. Gordon, G. G. Wallace, D. L. Officer, *J. Mater. Chem. C* **2013**, *1*, 3913-3916.

[9] J. S. Meisner, D. F. Sedbrook, M. Krikorian, J. Chen, A. Sattler, M. E. Carnes, C. B. Murray, M. Steigerwald, C. Nuckolls, *Chem. Sci.* **2012**, *3*, 1007-1014.

[10] H. Ma, Z. Bao, L. Bai, W. Cao, *Int. J. Org. Chem.* **2012**, *2*, 21-25.

[11] (a) R. L. Starr, T. Fu, E. A. Doud, I. Stone, X. Roy, L. Venkataraman, *J. Am. Chem. Soc.* **2020**, *142*, 7128-7133; (b) E. A. Doud, M. S. Inkpen, G. Lovat, E. Montes, D. W. Paley, M. L. Steigerwald, H. Vázquez, L. Venkataraman, X. Roy, *J. Am. Chem. Soc.* **2018**, *140*, 8944-8949.

[12] H. J. Yoon, J. Kuwabara, J.-H. Kim, C. A. Mirkin, *Science* **2010**, *330*, 66.

[13] B. S. Lukyanov, M. B. Lukyanova, *Chem. Heterocycl. Compd.* **2005**, *41*, 281-311.

[14] (a) D. E. Williams, C. R. Martin, E. A. Dolgopolova, A. Swifton, D. C. Godfrey, O. A. Ejegbavwo, P. J. Pellechia, M. D. Smith, N. B. Shustova, *J. Am. Chem. Soc.* **2018**, *140*, 7611-7622; (b) A. C. Benniston, A. Harriman, S. L. Howell, P. Y. Li, D. P. Lydon, *J. Org. Chem.* **2007**, *72*, 888-897; (c) F. Maya, J. M. Tour, *Tetrahedron* **2004**, *60*, 81-92.

[15] K. H. Chang, C. C. Huang, Y. H. Liu, Y. H. Hu, P. T. Chou, Y. C. Lin, *Dalton Trans.* **2004**, 1731-1738.

[16] H. J. Yin, B. C. Zhang, H. Z. Yu, L. Zhu, Y. Feng, M. Z. Zhu, Q. X. Guo, X. M. Meng, *J. Org. Chem.* **2015**, *80*, 4306-4312.

[17] C. Ma, A. Lo, A. Abdolmaleki, M. J. MacLachlan, *Org. Lett.* **2004**, *6*, 3841-3844.

[18] M. V. Reddington, *Bioconjug. Chem.* **2007**, *18*, 2178-2190.

[19] M. Schulz-Senft, P. J. Gates, F. D. Sönnichsen, A. Staubitz, *Dyes Pigm.* **2017**, *136*, 292-301.

[20] É. R. Zakhs, L. A. Zvenigorodskaya, N. G. Leshenyuk, V. P. Martynova, *Chem. Heterocycl. Compd.* **1977**, *13*, 1055-1061.

[21] H. Yin, B. Zhang, H. Yu, L. Zhu, Y. Feng, M. Zhu, Q. Guo, X. Meng, *J. Org. Chem.* **2015**, *80*, 4306-4312.

[22] B. N. Boden, A. Abdolmaleki, C. T. Z. Ma, M. J. MacLachlan, *Can. J. Chem.* **2008**, *86*, 50-64.

[23] M. Acelas, A. F. Sierra, C. A. Sierra, *Synth. Commun.* **2020**, *50*, 1335-1352.

[24] J. Sturala, R. Cibulka, *Eur. J. Org. Chem.* **2012**, *2012*, 7066-7074.

[25] M. A. V. R. da Silva, A. I. M. C. L. Ferreira, J. R. B. Gomes, *Bull. Chem. Soc. Jpn.* **2006**, *79*, 1852-1859.

[26] O. V. Dolomanov, L. J. Bourhis, R. J. Gildea, J. A. Howard, H. Puschmann, *J. Appl. Crystallogr.* **2009**, *42*, 339-341.

[27] (a) G. M. Sheldrick, *Acta Crystallogr. A* **2008**, *64*, 112-122; (b) G. M. Sheldrick, *Acta Crystallogr. A* **2015**, *71*, 3-8; (c) G. M. Sheldrick, *Acta. Crystallogr. C* **2015**, *71*, 3-8.

[28] (a) I. Zouev, M. Kaftory, *Acta. Crystallogr. C* **2008**, *64*, o372-o375; (b) W. Clegg, N. C. Norman, T. Flood, L. Sallans, W. S. Kwak, P. L. Kwiatkowski, J. G. Lasch, *Acta. Crystallogr. C* **1991**, *47*, 817-824.

[29] (a) A. D. Pugachev, I. V. Ozhogin, M. B. Lukyanova, B. S. Lukyanov, A. S. Kozlenko, I. A. Rostovtseva, N. I. Makarova, V. V. Tkachev, S. M. Aldoshin, A. V. Metelitsa, *J. Mol. Struct.* **2021**, *1229*, 129615; (b) F. M. Raymo, S. Giordani, A. J. P. White, D. J. Williams, *J. Org. Chem.* **2003**, *68*, 4158-4169.

[30] (a) J. O. S. Beckett, M. M. Olmstead, J. C. Fettinger, D. A. Gray, S. Manabe, M. Mascal, *Acta. Crystallogr. E* **2016**, *72*, 1659-1662; (b) A. D. Pugachev, V. V. Tkachev, S. M. Aldoshin, N. I. Makarova, I. A. Rostovtseva, A. V. Metelitsa, N. V. Stankevich, G. V. Shilov, B. S. Lukyanov, *Russ. J. Gen. Chem.* **2021**, *91*, 1297-1304; (c) S. Raić-Malić, L. Tomašković, D. Mrvoš-Sermek, B. Prugovečki, M. Cetina, M. Grdiša, K. Pavelić, A. Mannschreck, J. Balzarini, E. De Clercq, M. Mintas, *Biorg. Med. Chem.* **2004**, *12*, 1037-1045.

[31] X. Meng, G. Y. Qi, C. Zhang, K. Wang, B. Zou, Y. G. Ma, *Chem. Commun.* **2015**, *51*, 9320-9323.

[32] V. K. Seiler, N. Tumanov, K. Robeyns, J. Wouters, B. Champagne, T. Leyssens, *Crystals* **2017**, *7*.

[33] (a) E. Artacho, E. Anglada, O. Dieguez, J. D. Gale, A. Garcia, J. Junquera, R. M. Martin, P. Ordejon, J. M. Pruneda, D. Sanchez-Portal, J. M. Soler, *J. Phys. Condens. Matter* **2008**, *20*; (b) J. M. Soler, E. Artacho, J. D. Gale, A. Garcia, J. Junquera, P. Ordejon, D. Sanchez-Portal, *J. Phys. Condens. Matter* **2002**, *14*, 2745-2779.

[34] J. Ferrer, C. J. Lambert, V. M. Garcia-Suarez, D. Z. Manrique, D. Visontai, L. Oroszlany, R. Rodriguez-Ferradas, I. Grace, S. W. D. Bailey, K. Gillemot, H. Sadeghi, L. A. Algharagholy, *New J. Phys.* **2014**, *16*.
